# Supplementary material for: Postoperative Analgesic Effectiveness of Peripheral Nerve Blocks in Cesarean Delivery: A Systematic Review and Network Meta-Analysis
Source: J Pers Med. 2022 Apr 14;12(4):634. doi: 10.3390/jpm12040634 (PMC9033028; doi:10.3390/jpm12040634)
Supplement: Supplementary file 1 [file jpm-12-00634-s001.zip › jpm-1607529 - Supplementary Figures and Tables - Conversion.pdf]

Journal of
Personalized Medicine

MDPI

Supplementary Materials

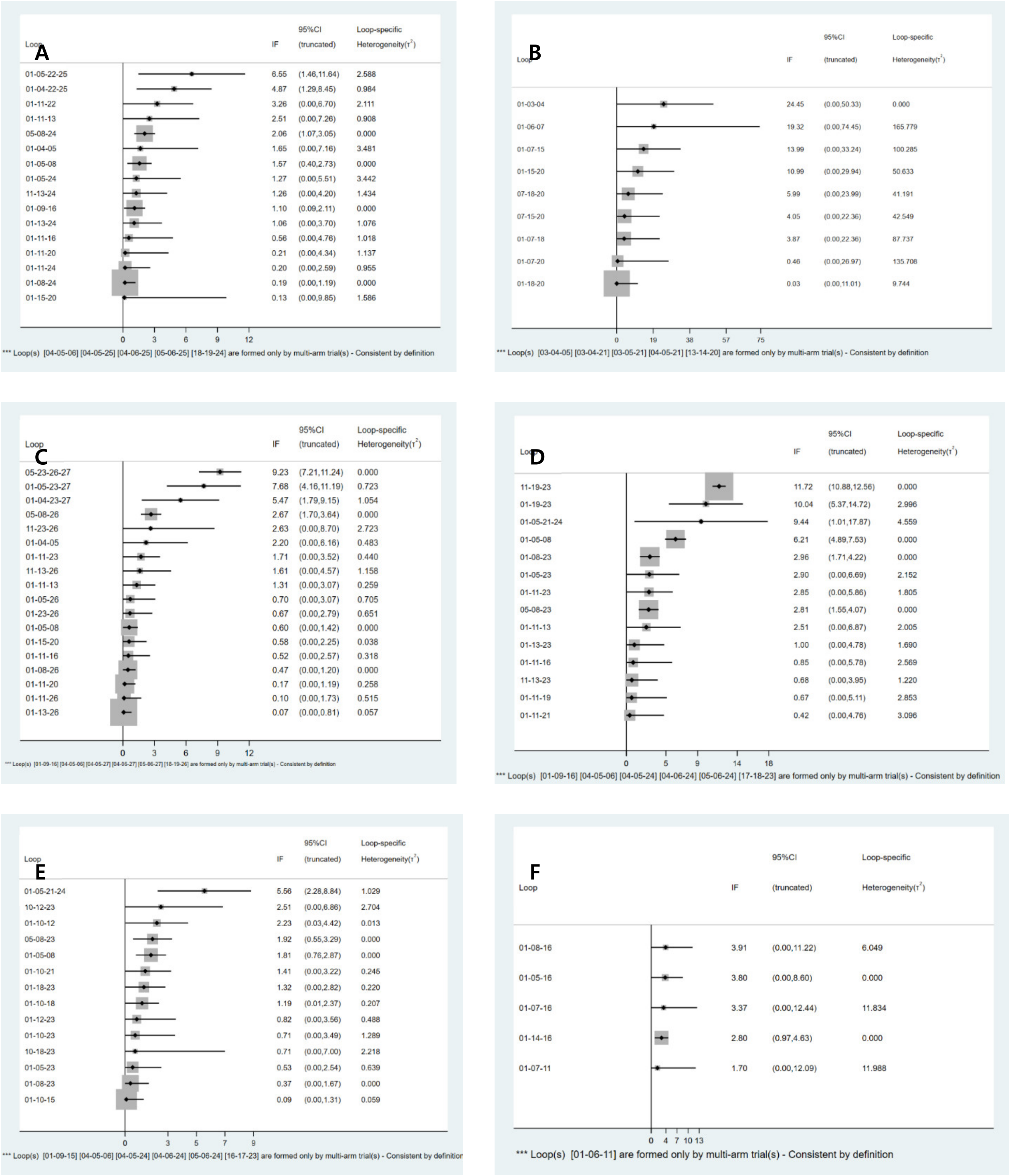

Figure S1. Inconsistency plots of direct and indirect comparisons for the entire network. Triangular loops are formed by three interventions all compared with each other and quadratic loops are formed by four interventions that each one is compared exactly with two other interventions in the loop. Inconsistency factor (IF) means the absolute difference between direct and indirect estimates for each paired comparison in the loop. (**A**). Pain at rest 6 h after surgery, (**B**). Postoperative cumulative 24 h morphine equivalent consumption, (**C**). Pain at rest 24 h after surgery, (**D**). Dynamic pain at 6 h after surgery, (**E**). Dynamic pain at 24 h after surgery, (**F**). The time to first analgesic request.

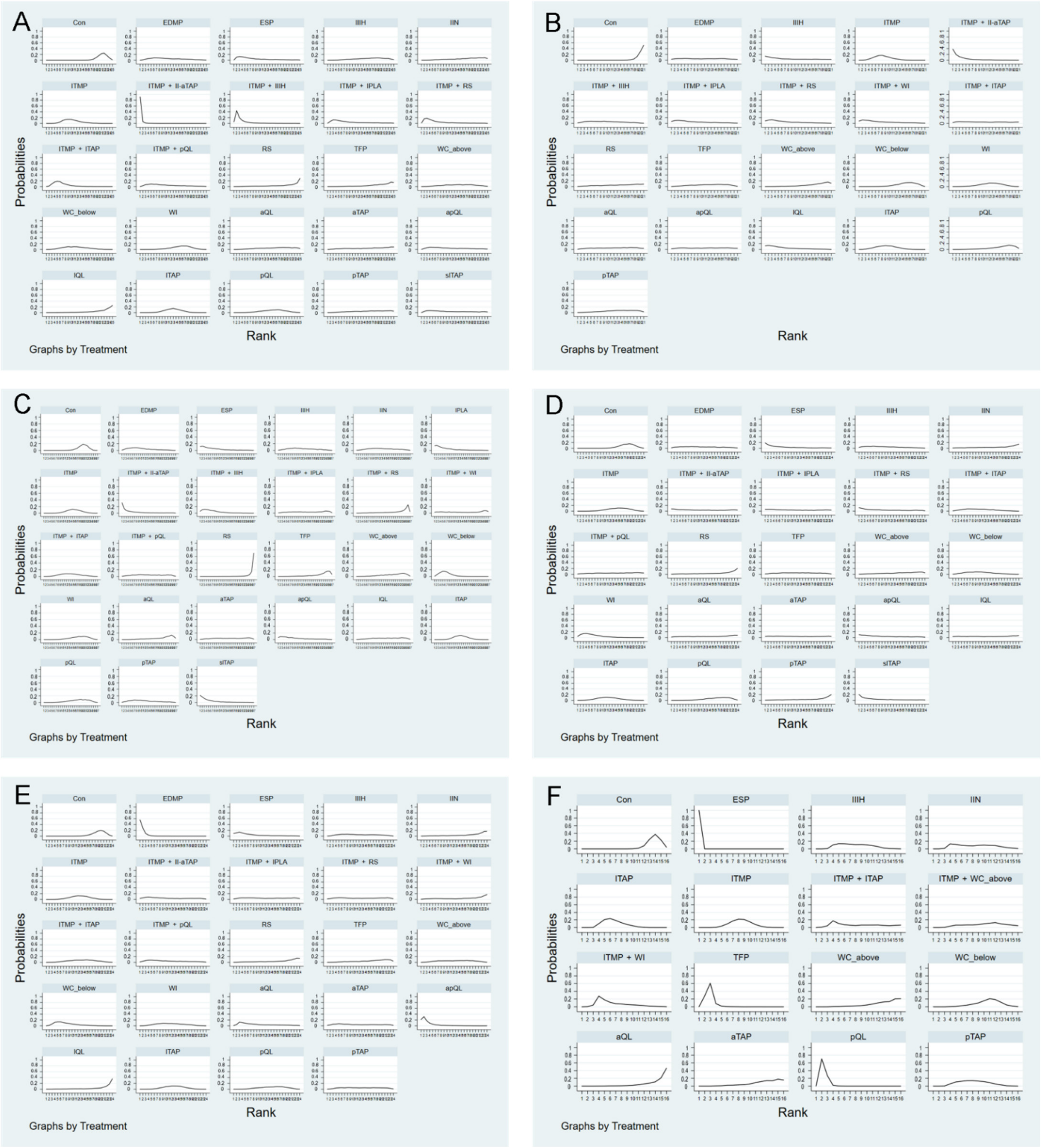

Figure S2. The rankograms of all outcomes. A rankogram for each specific intervention is a plot of the probability of assuming each of the possible rank. Each number on the X−axis means the possible rank among the total number of interventions in each network. (**A**). Pain at rest 6 h after surgery, (**B**). Postoperative cumulative 24 h morphine equivalent consumption, (**C**). Pain at rest 24 h after surgery, (**D**). Dynamic pain at 6 h after surgery, (**E**). Dynamic pain at 24 h after surgery, (**F**). The time to first analgesic request.

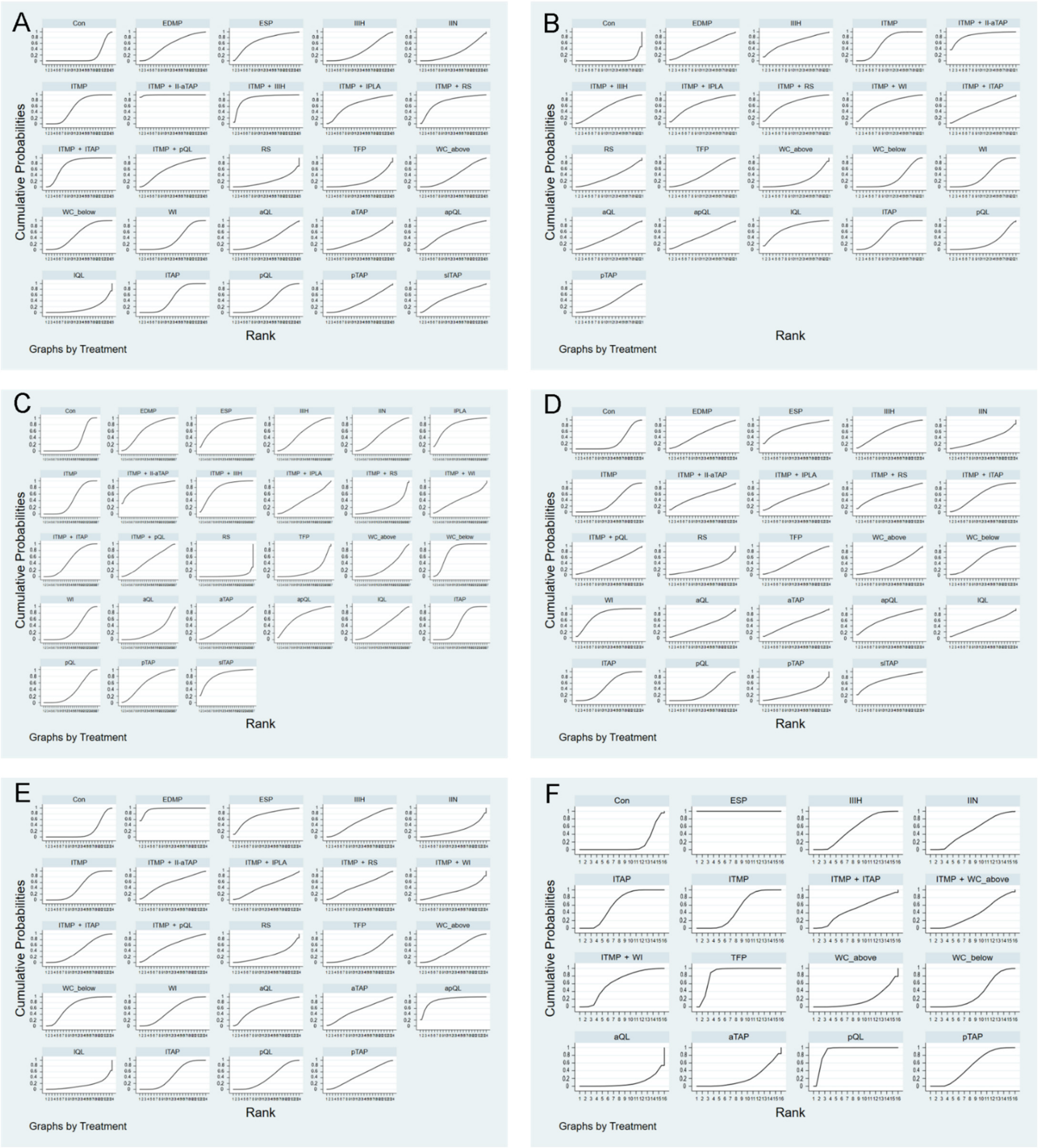

Figure S3. The cumulative ranking curves of all outcomes. Each one present the cumulative ranking probability. The numbers on the X−axis are the possible rank of each intervention and the numbers on the Y−axis mean the cumulative probability for each intervention to be the best option, among the two options, three options, and so on. (**A**). Pain at rest 6 h after surgery, (**B**). Postoperative cumulative 24 h morphine equivalent consumption, (**C**). Pain at rest 24 h after surgery, (**D**). Dynamic pain at 6 h after surgery, (**E**). Dynamic pain at 24 h after surgery, (**F**). The time to first analgesic request.

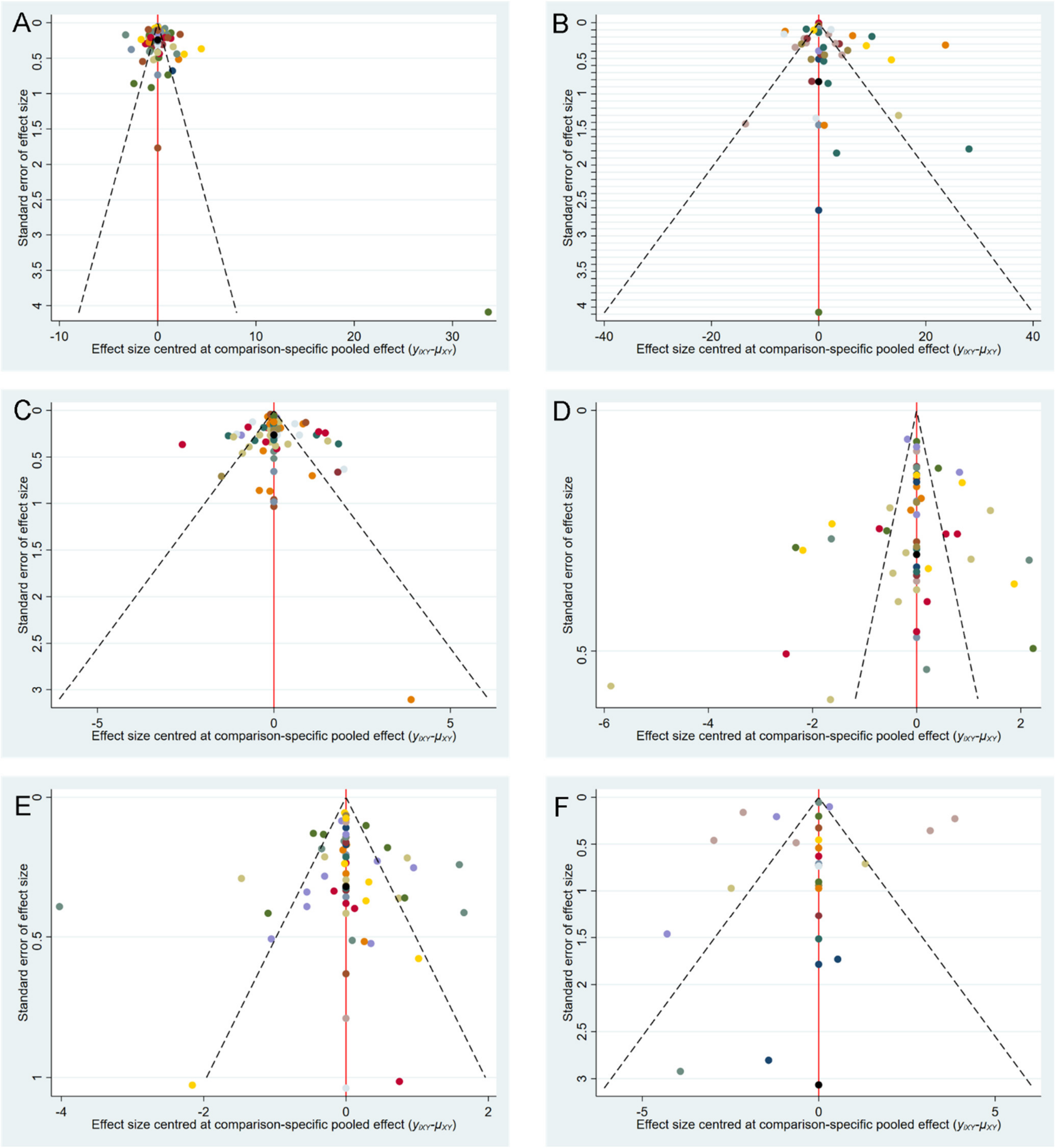

Figure S4. Comparison−adjusted funnel plots. The red line means the null hypothesis that the study−specific effect sizes are not different with the respective comparison−specific pooled effect estimates. In the presence of small study effects, the plot is asymmetric around the red line. (**A**). Pain at rest 6 h after surgery, (**B**). Postoperative cumulative 24 h morphine equivalent consumption, (**C**). Pain at rest 24 h after surgery, (**D**). Dynamic pain at 6 h after surgery, (**E**). Dynamic pain at 24 h after surgery, (**F**). The time to first analgesic request.

(**A**) Trace plot and density plot in fixed effect model.

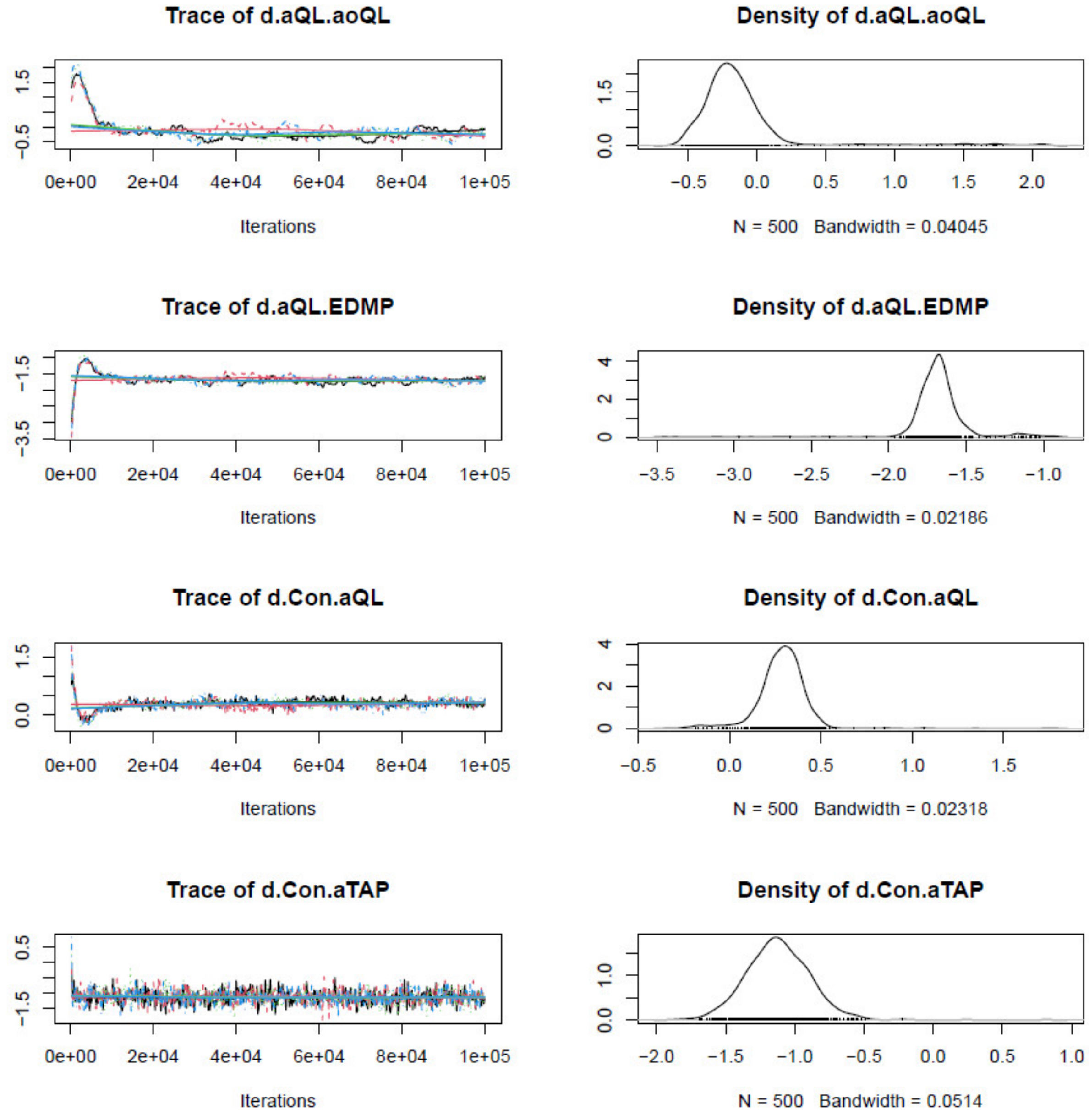

Figure S5. *Cont.*

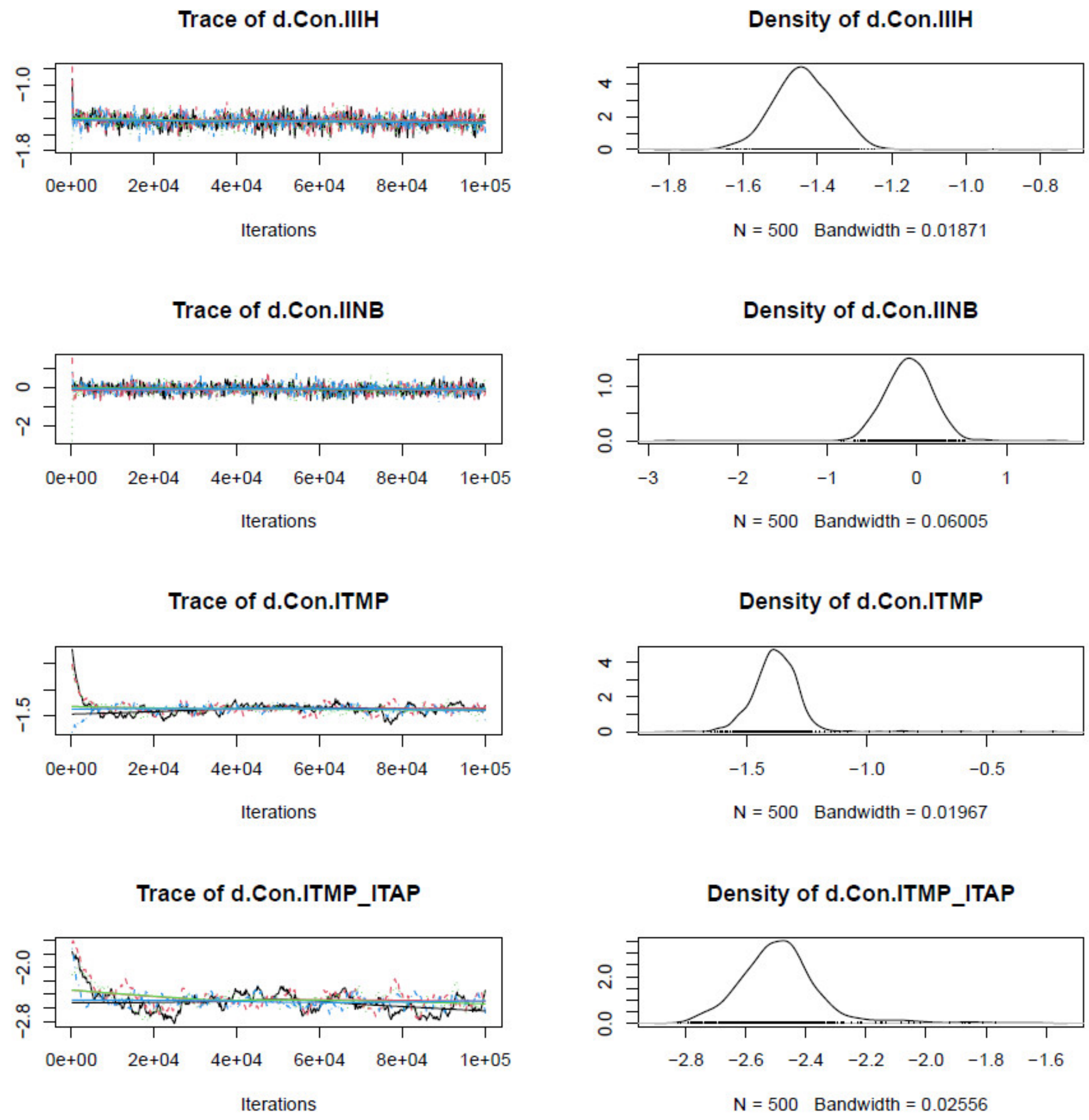

Figure S5. *Cont.*

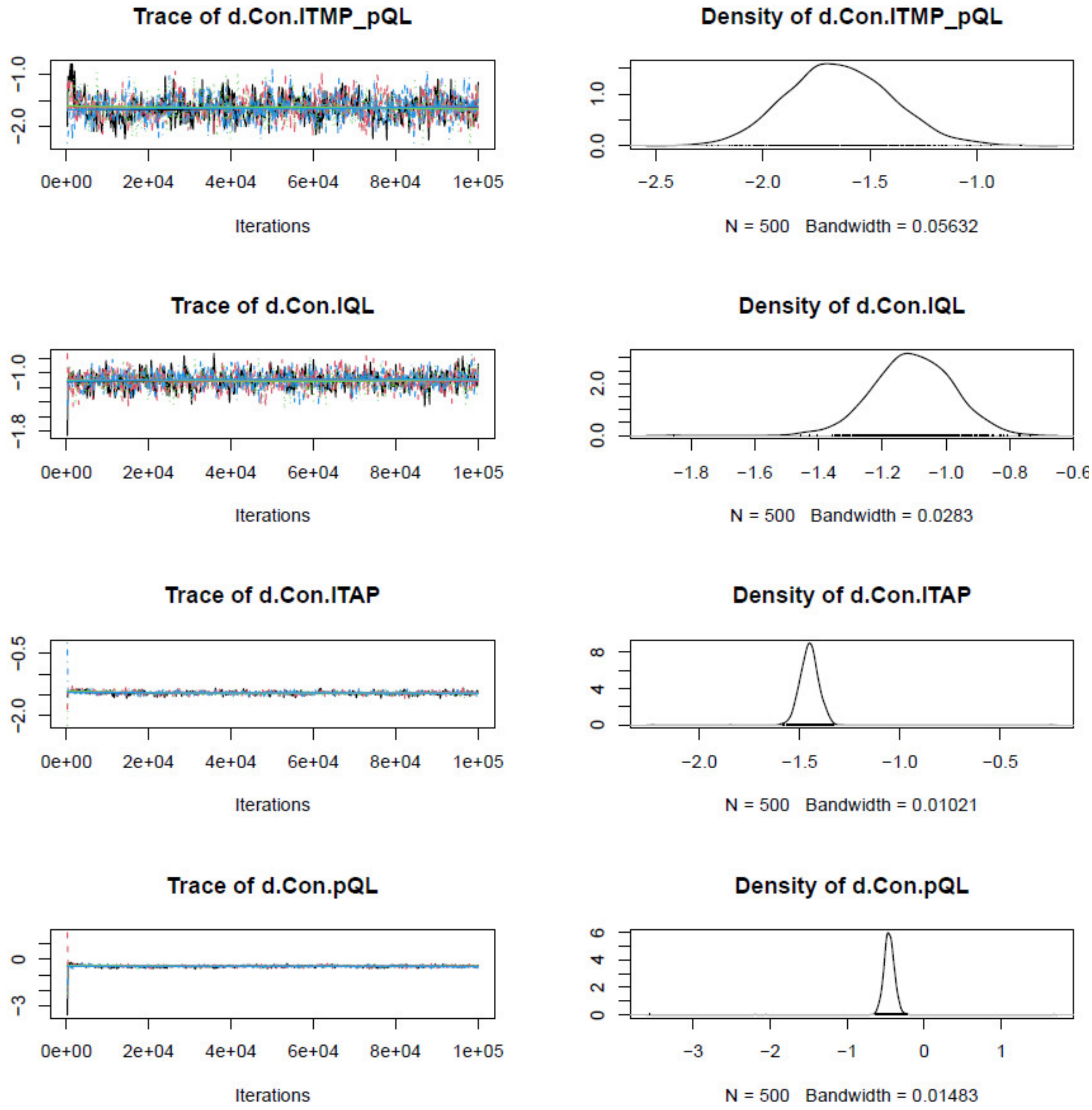

Figure S5. *Cont.*

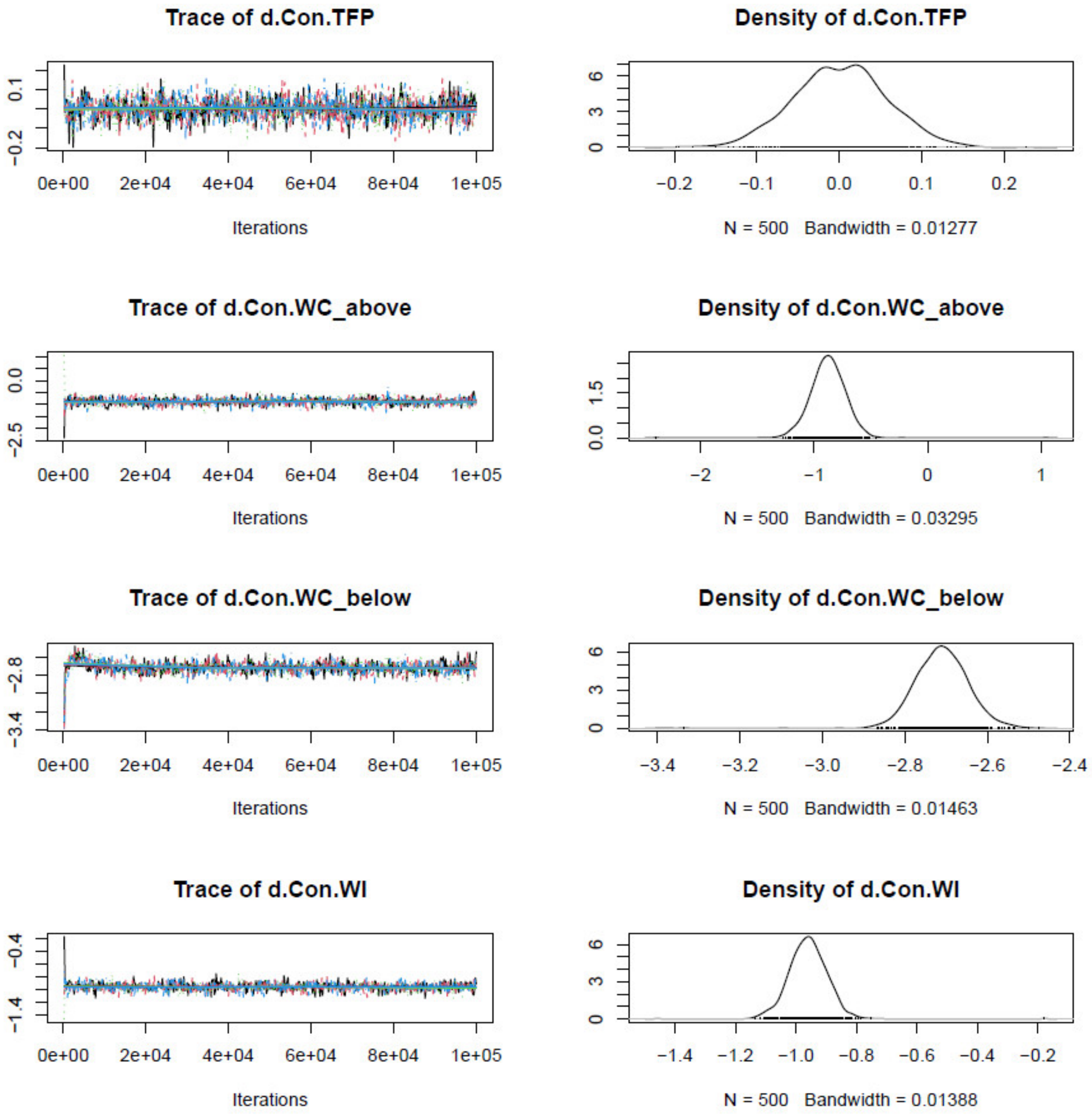

Figure S5. *Cont.*

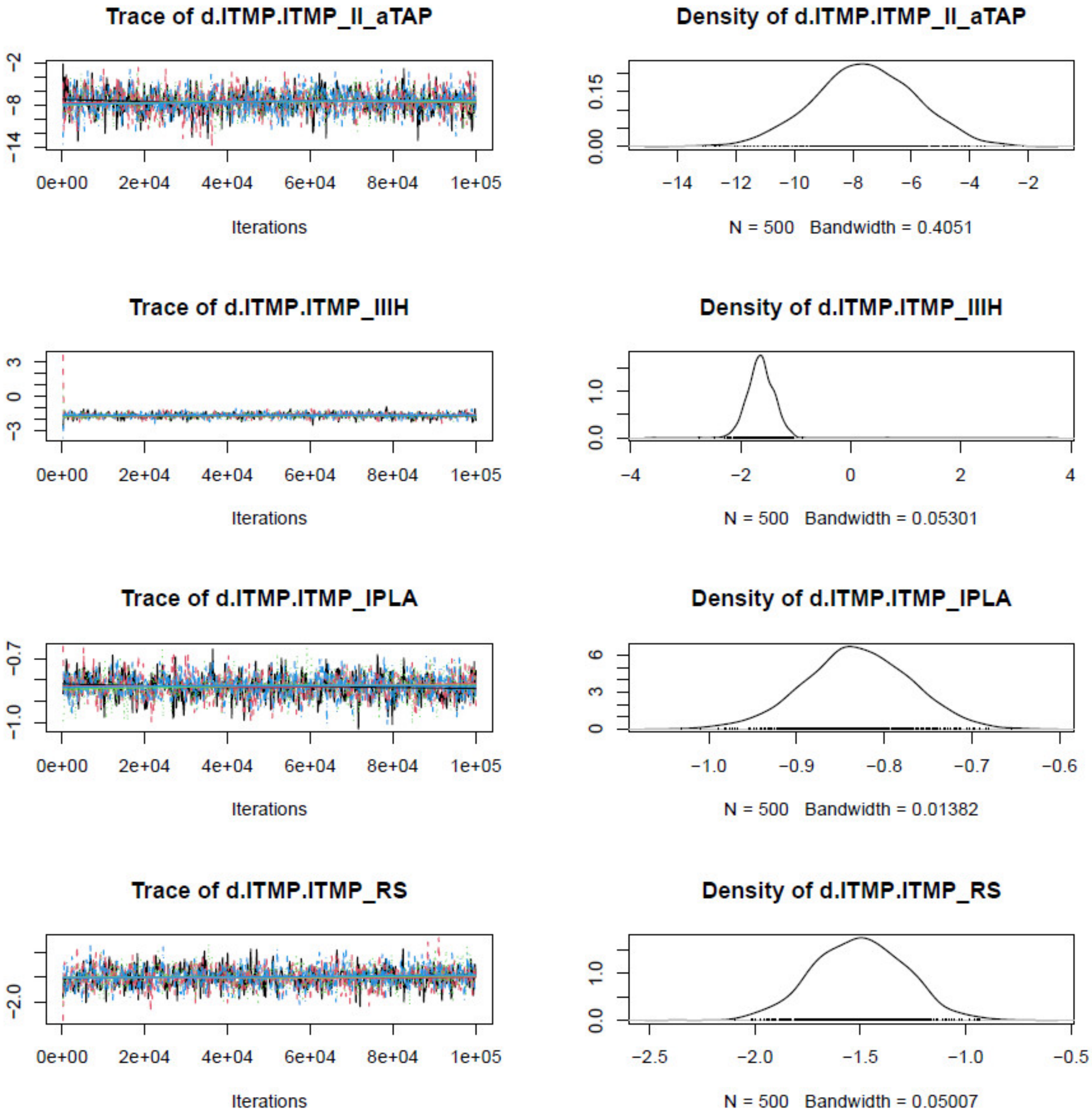

Figure S5. *Cont.*

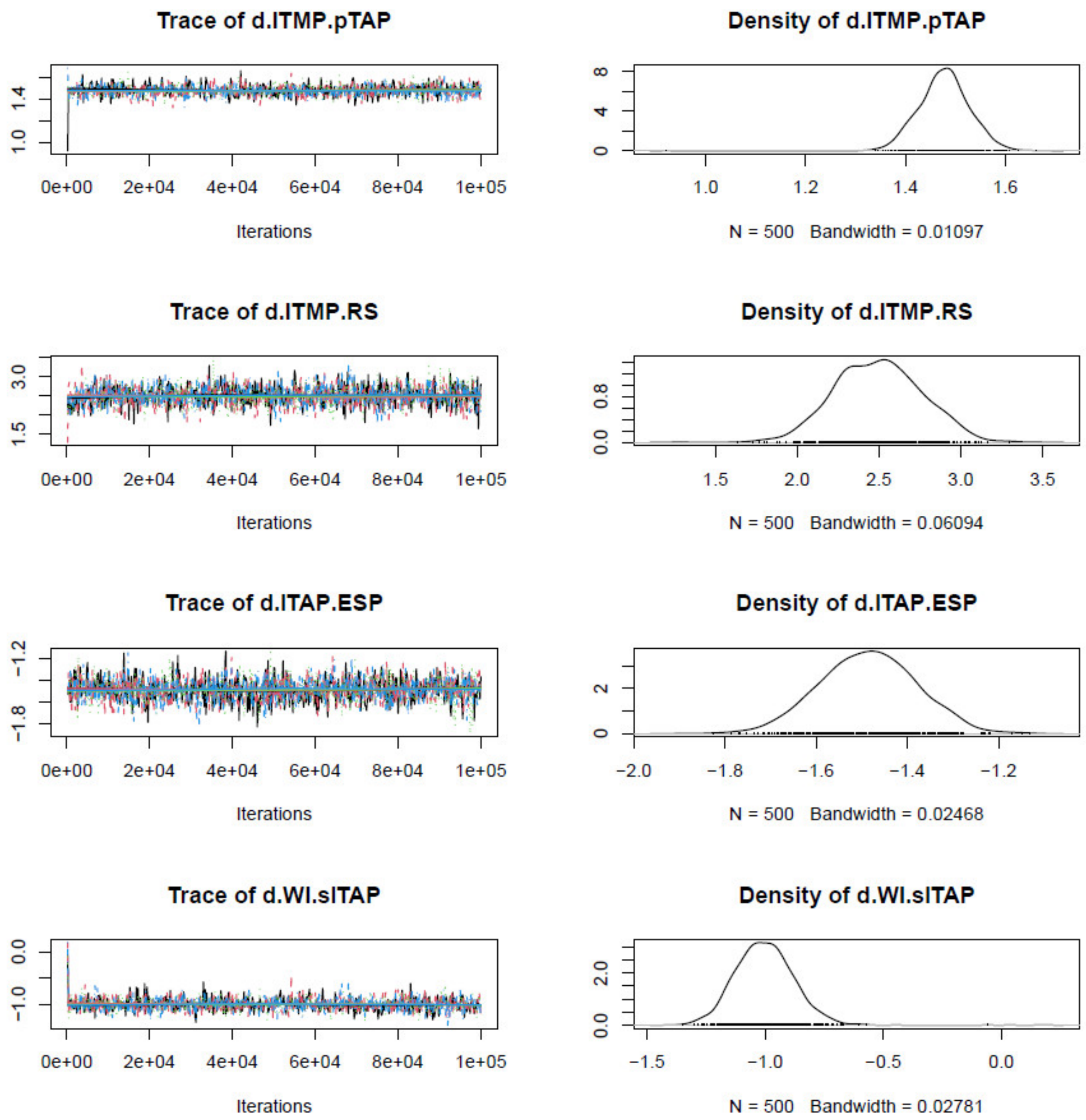

Figure S5. *Cont.*

(**B**) Trace plot and density plot in random effect model.

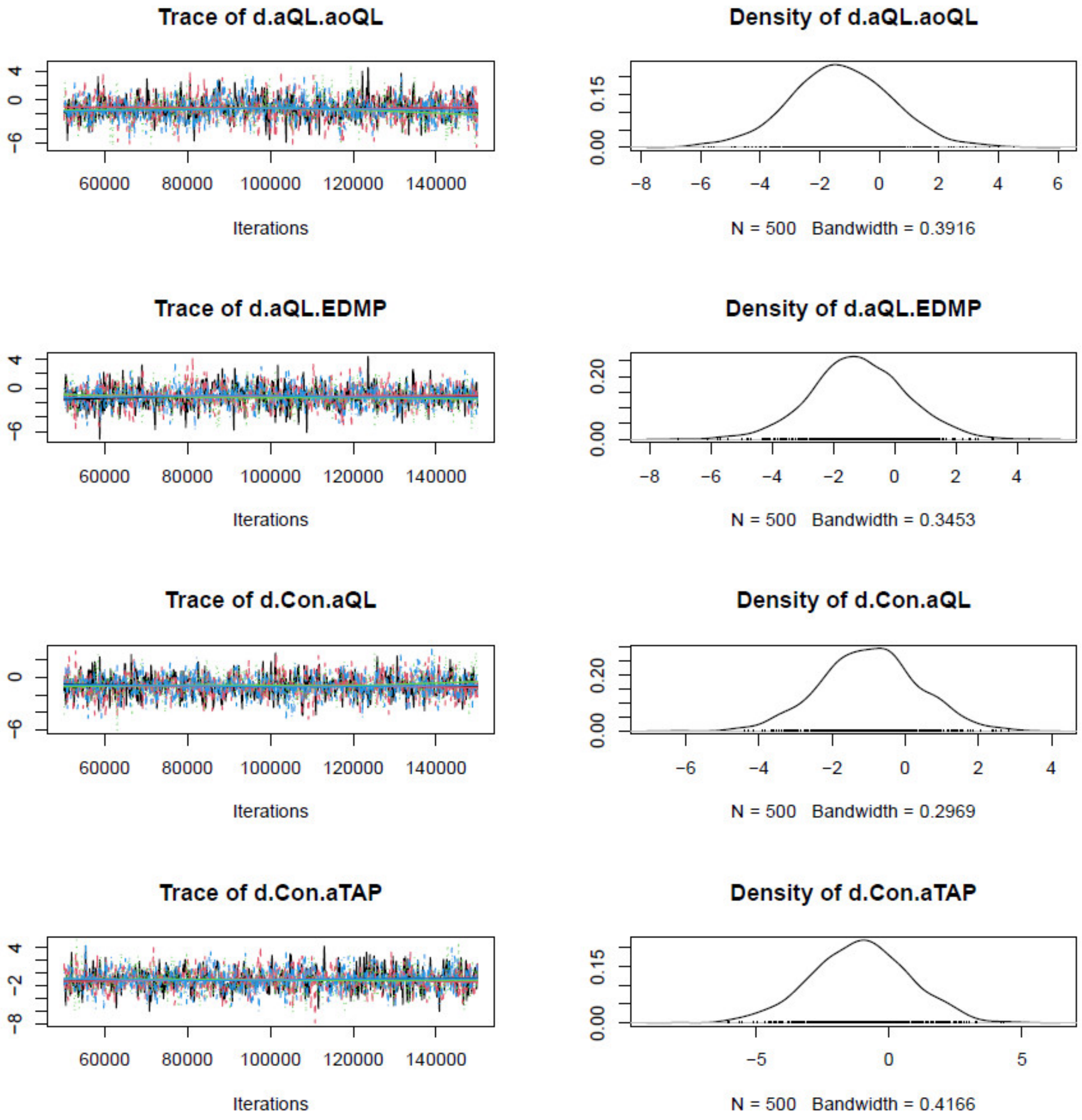

Figure S5. *Cont.*

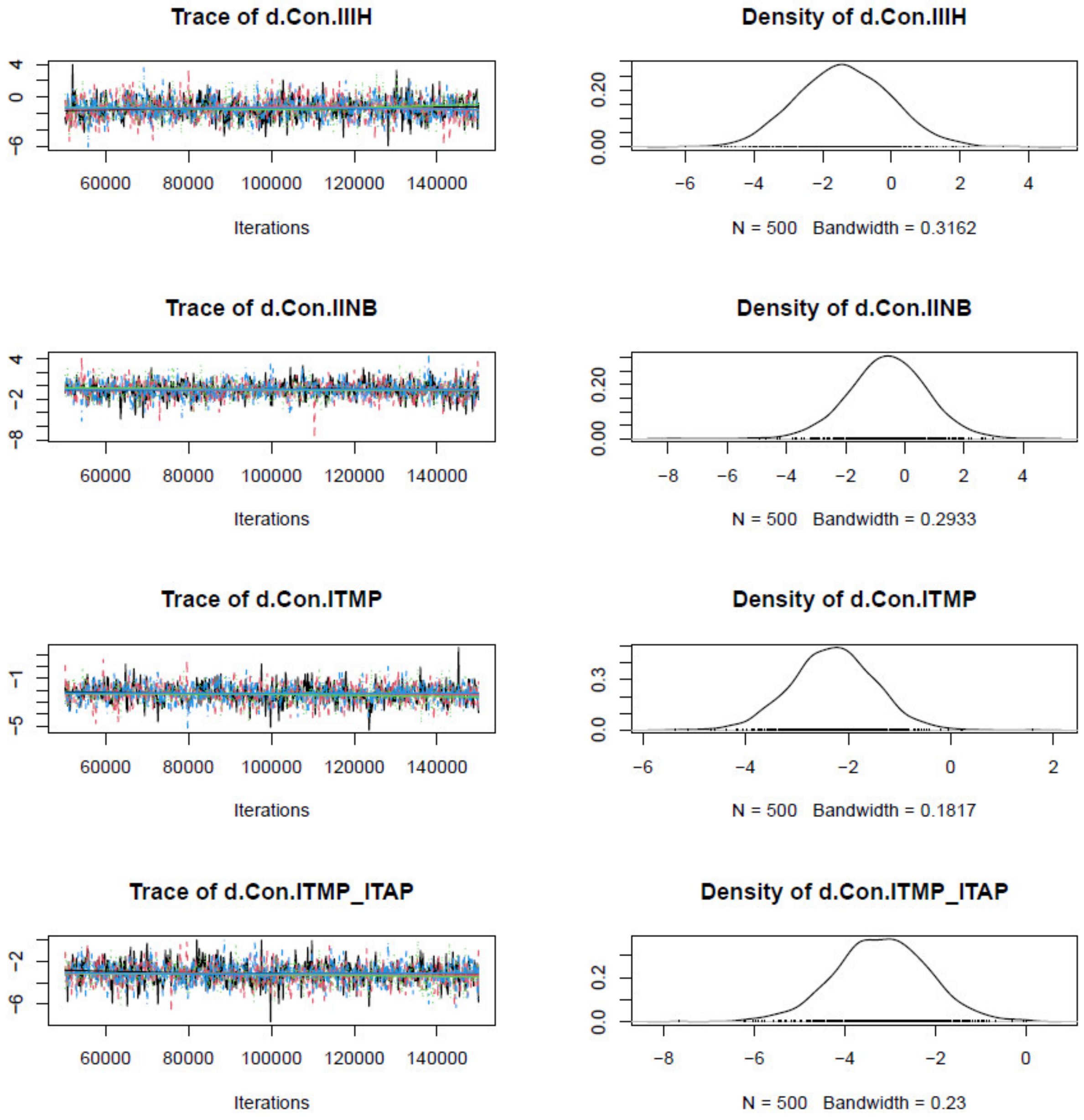

Figure S5. *Cont.*

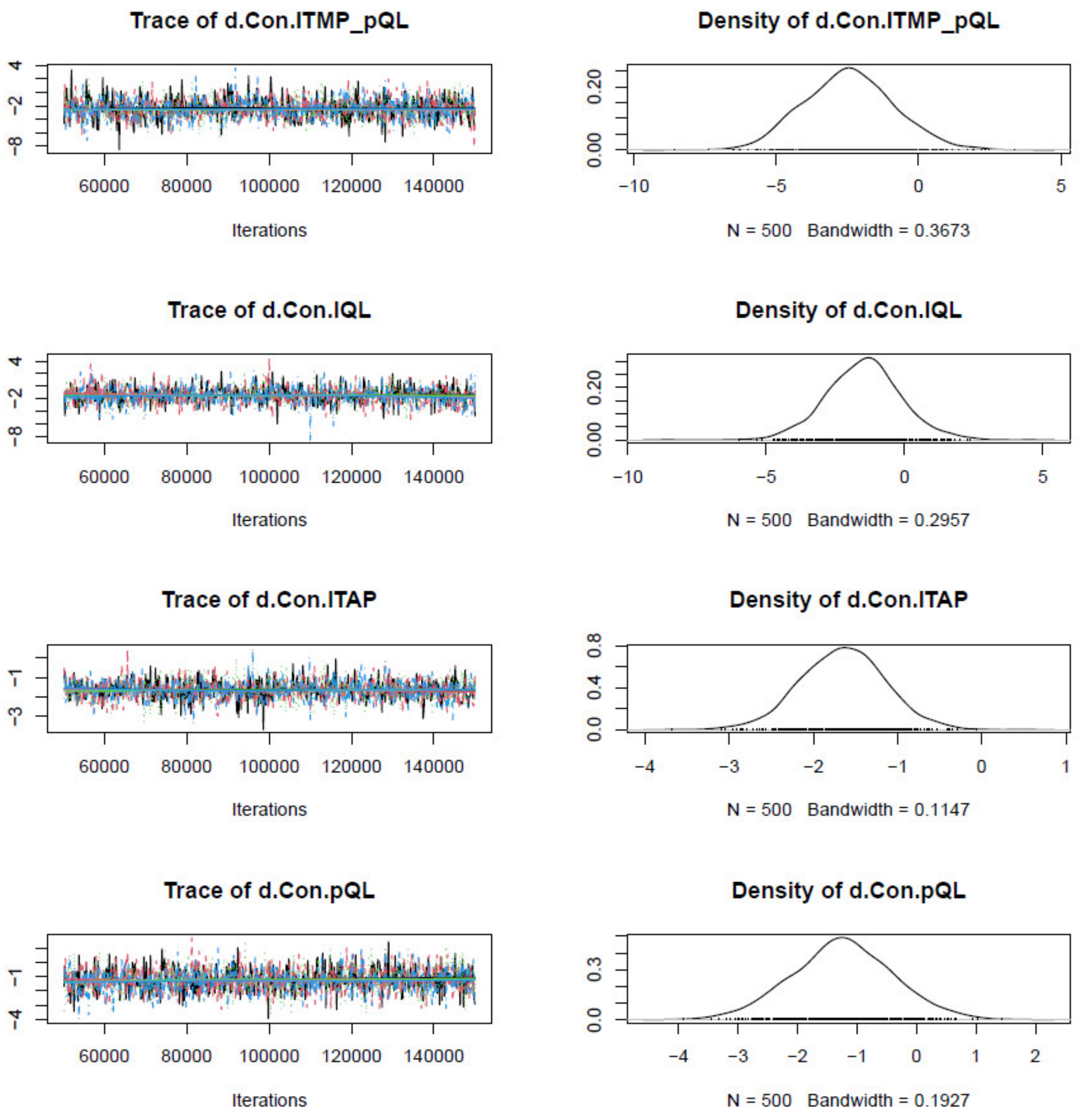

Figure S5. *Cont.*

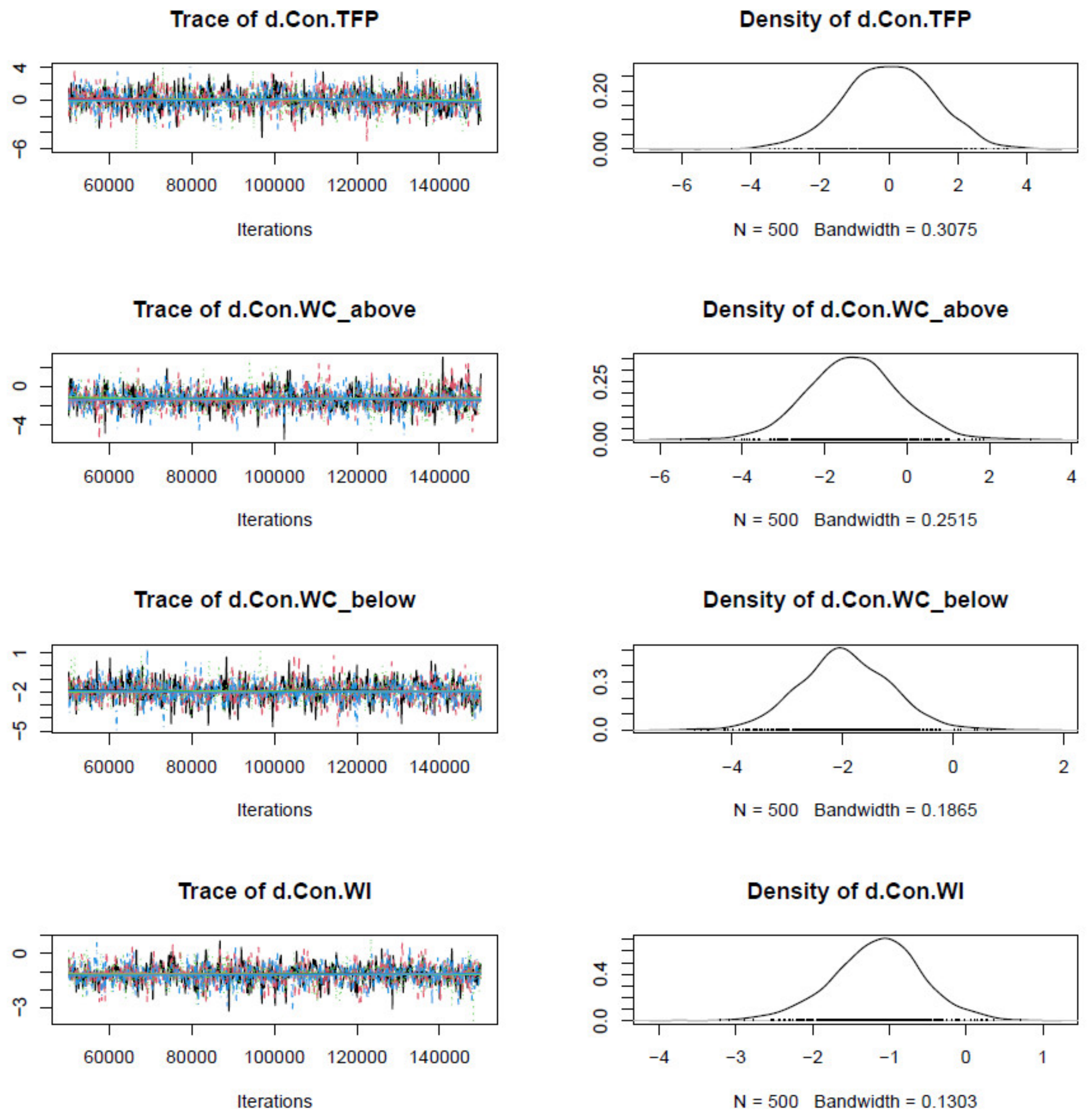

Figure S5. *Cont.*

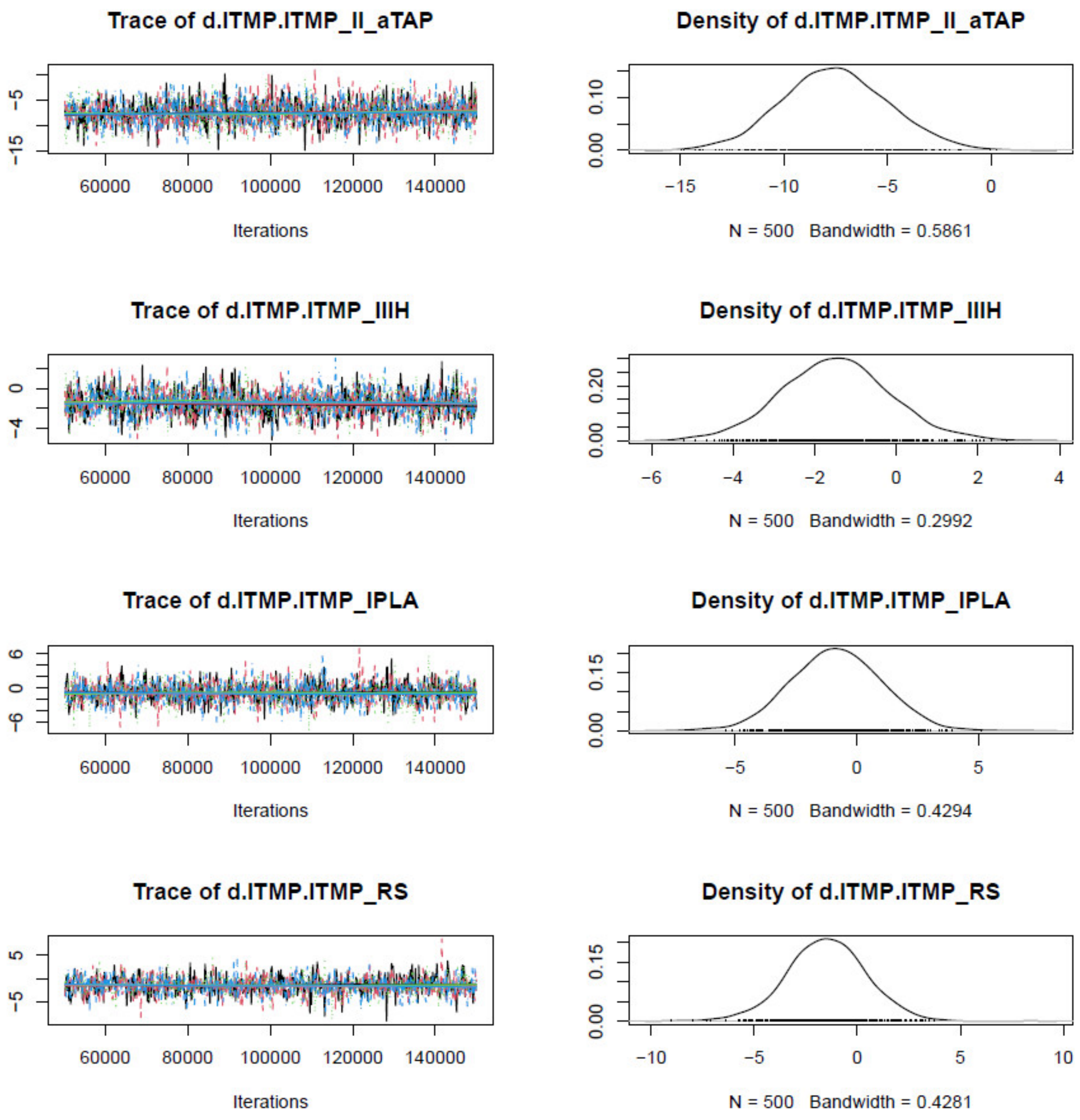

Figure S5. *Cont.*

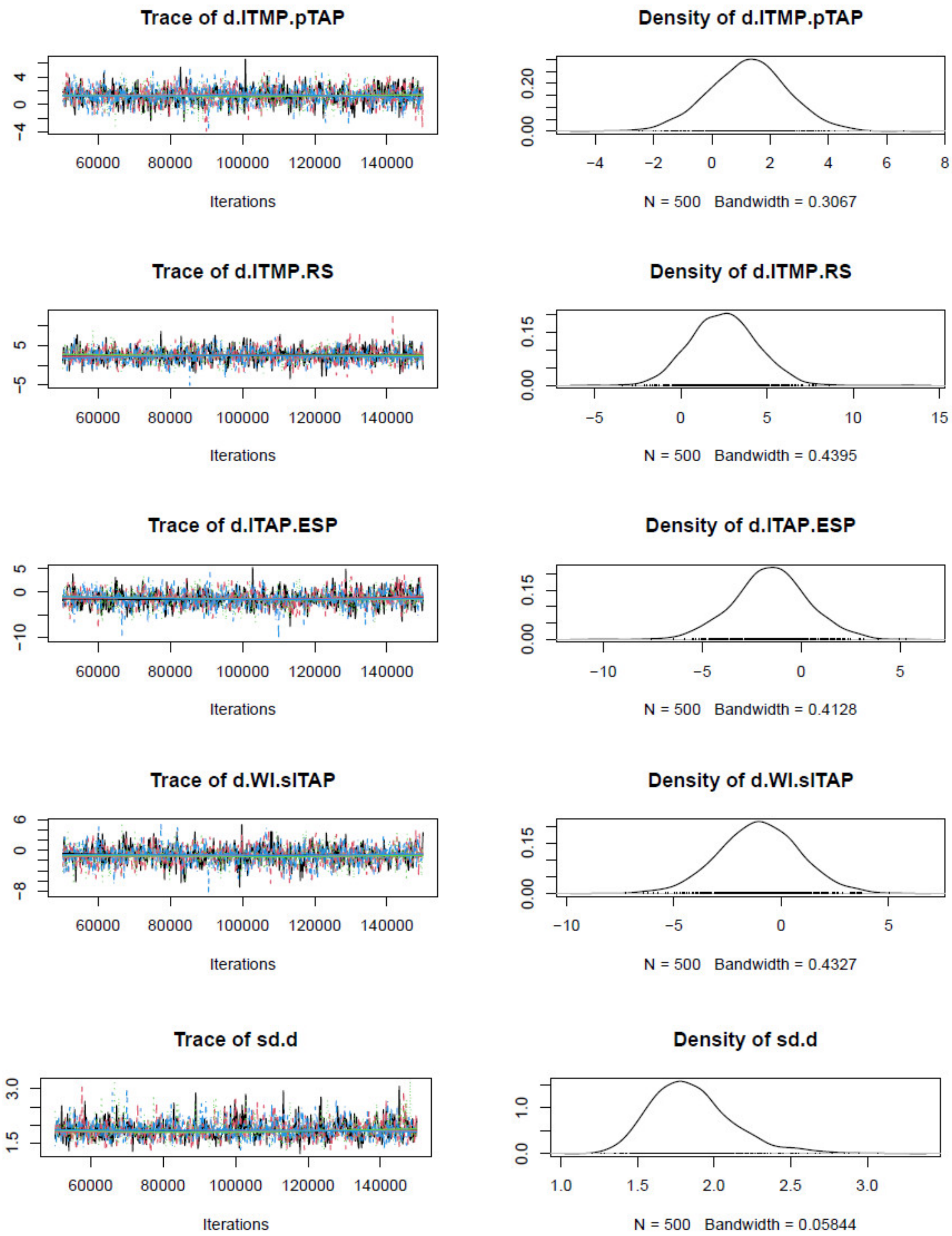

Figure S5. *Cont.*

(**C**) Gelman-Rubin-Brooks plot in fixed effect model.

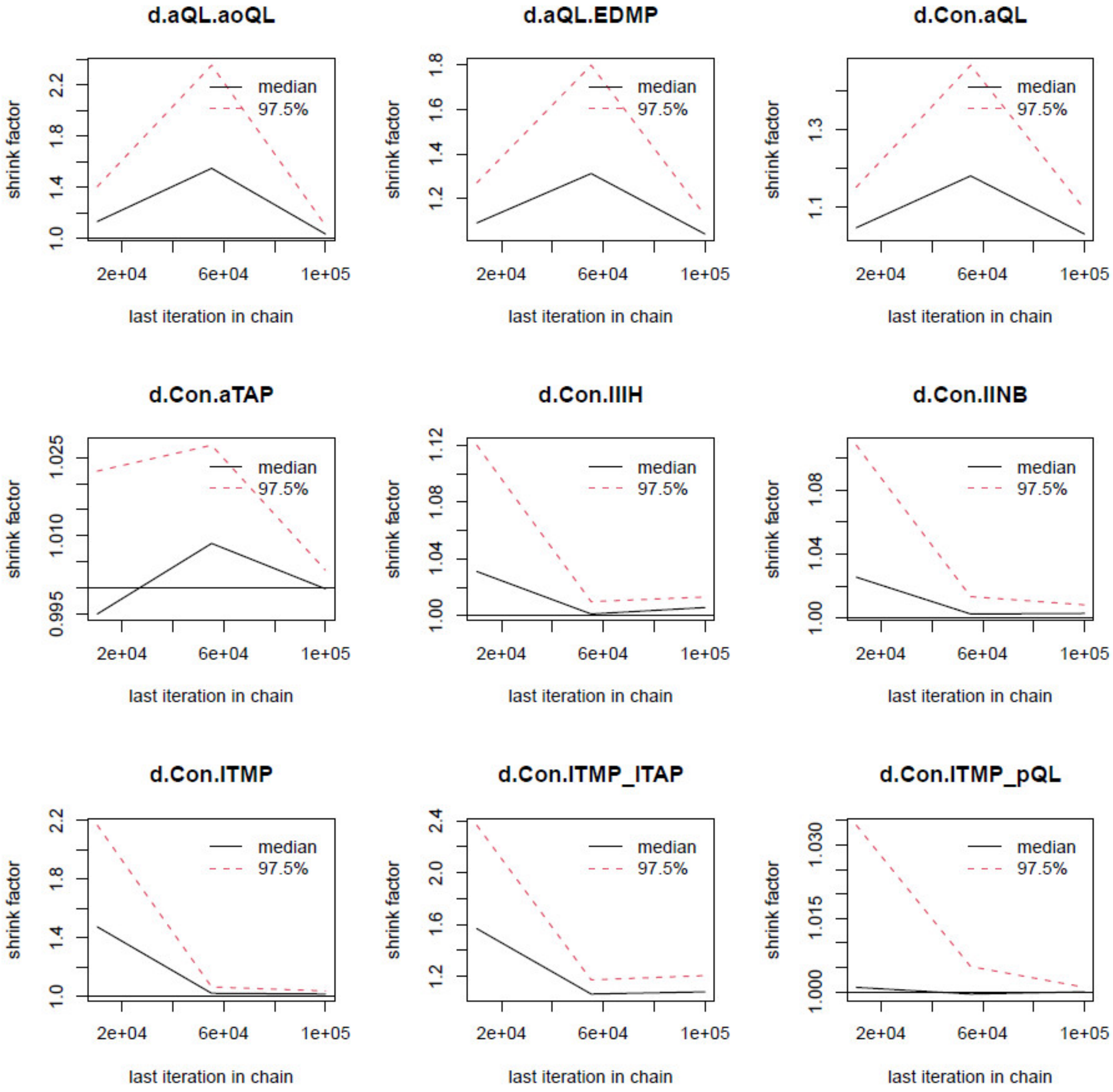

Figure S5. *Cont.*

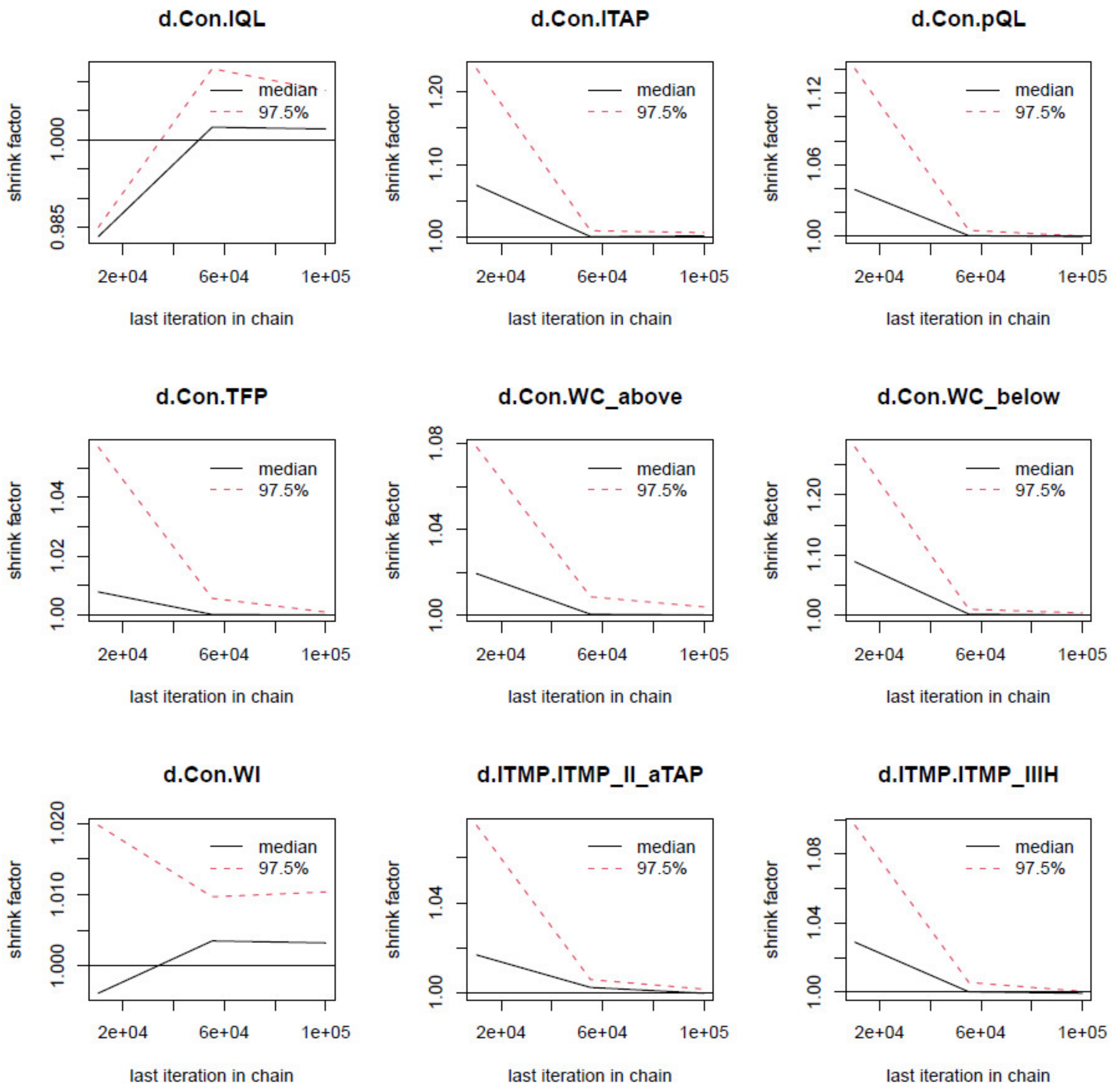

Figure S5. *Cont.*

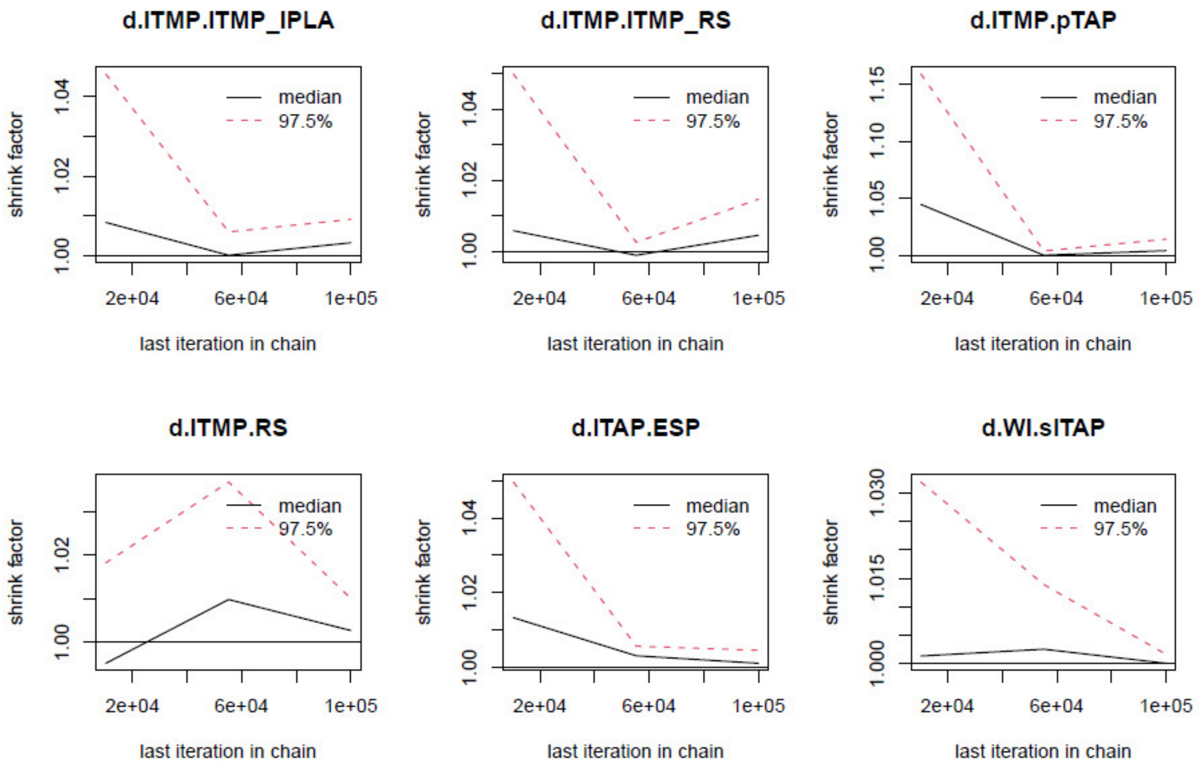

Figure S5. *Cont.*

(**D**) Gelman-Rubin-Brooks plot in random effect model.

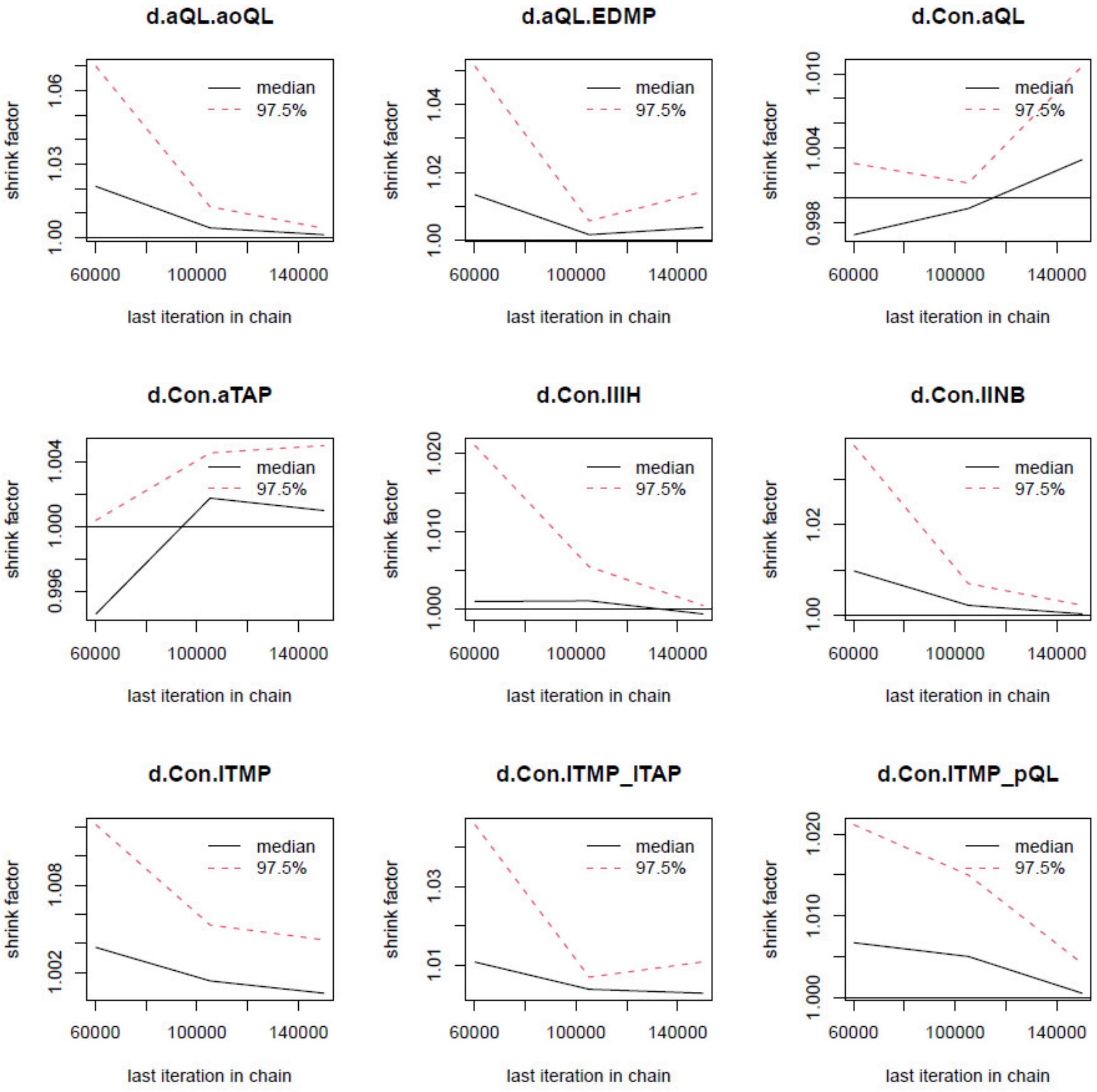

Figure S5. *Cont.*

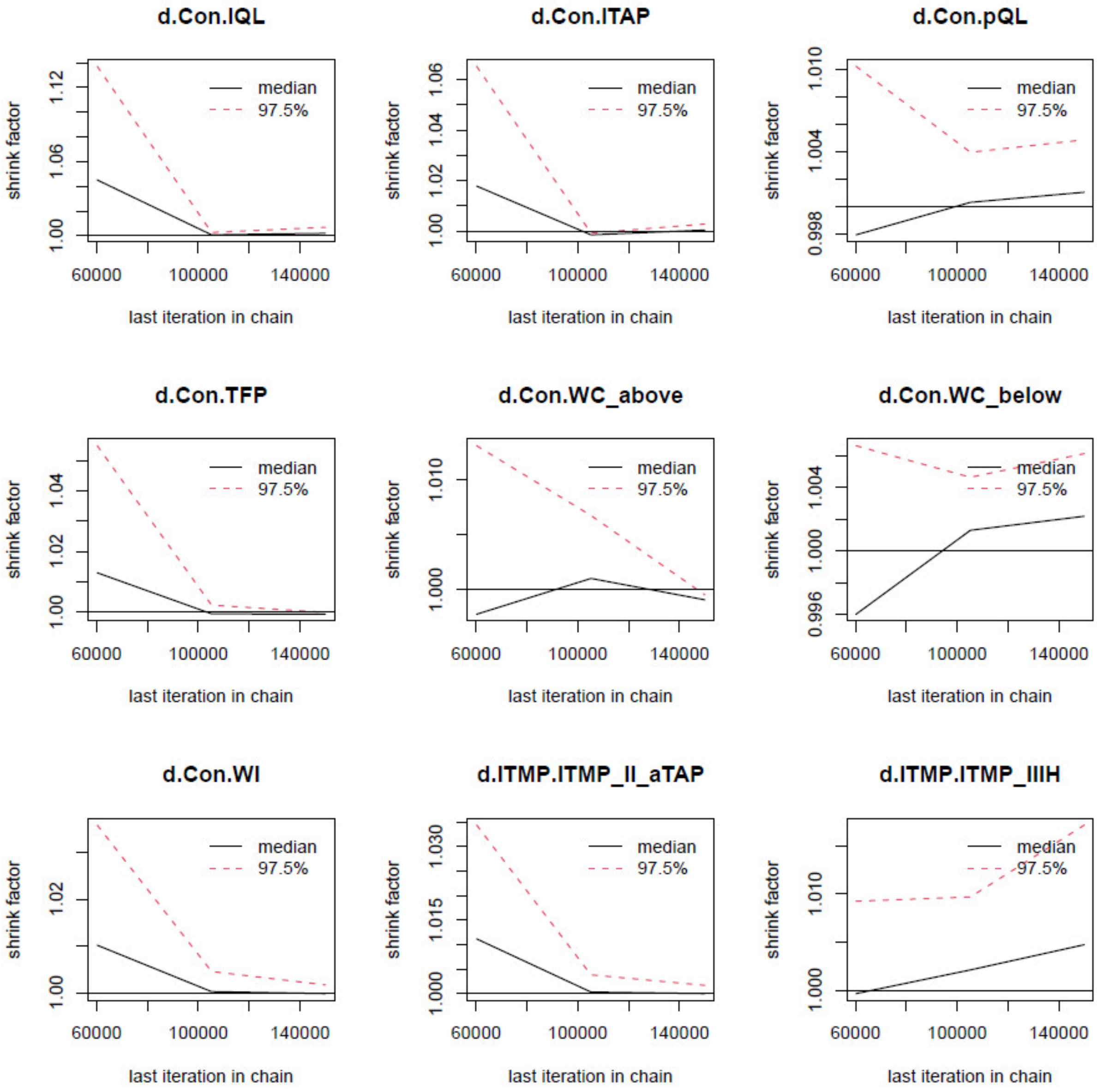

Figure S5. *Cont.*

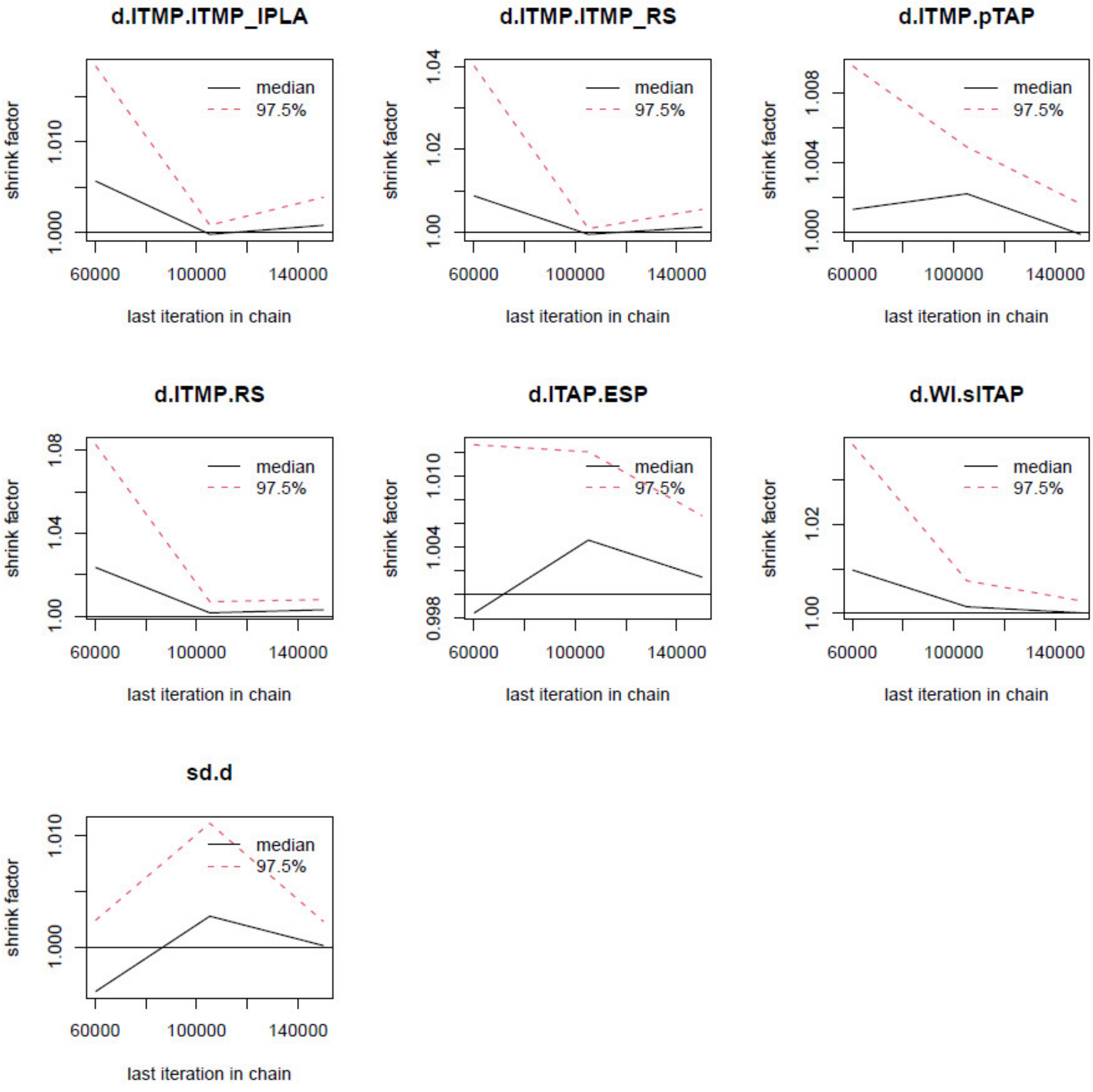

Figure S5. *Cont.*

(**E**) Forest plot comparing with control in random effect model.

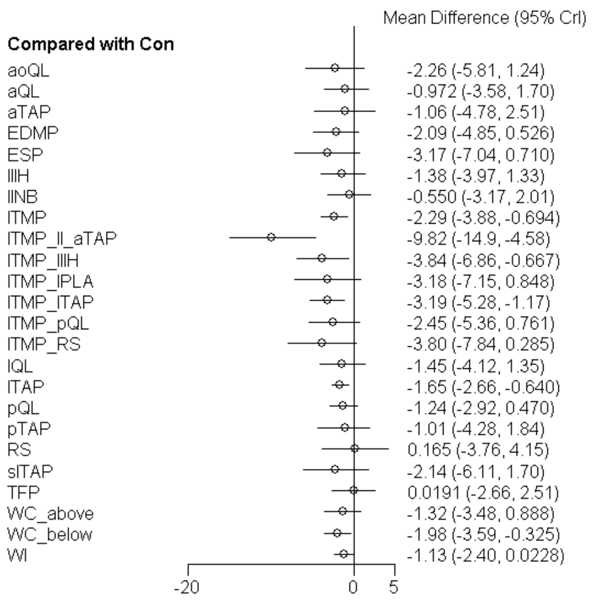

(**F**) Node splitting plot in random effect model.

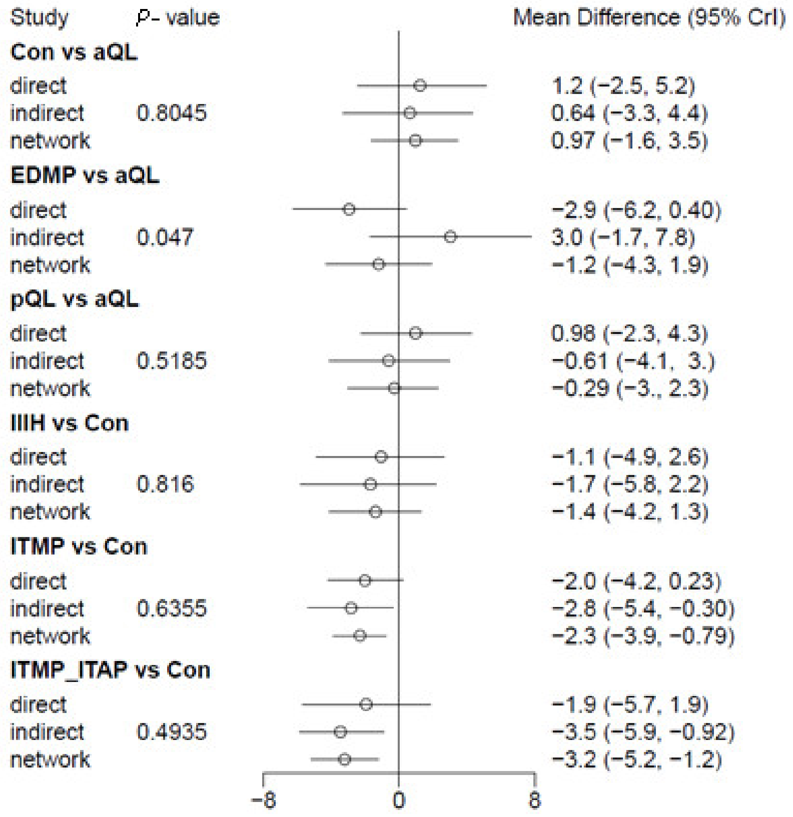

Figure S5. *Cont.*

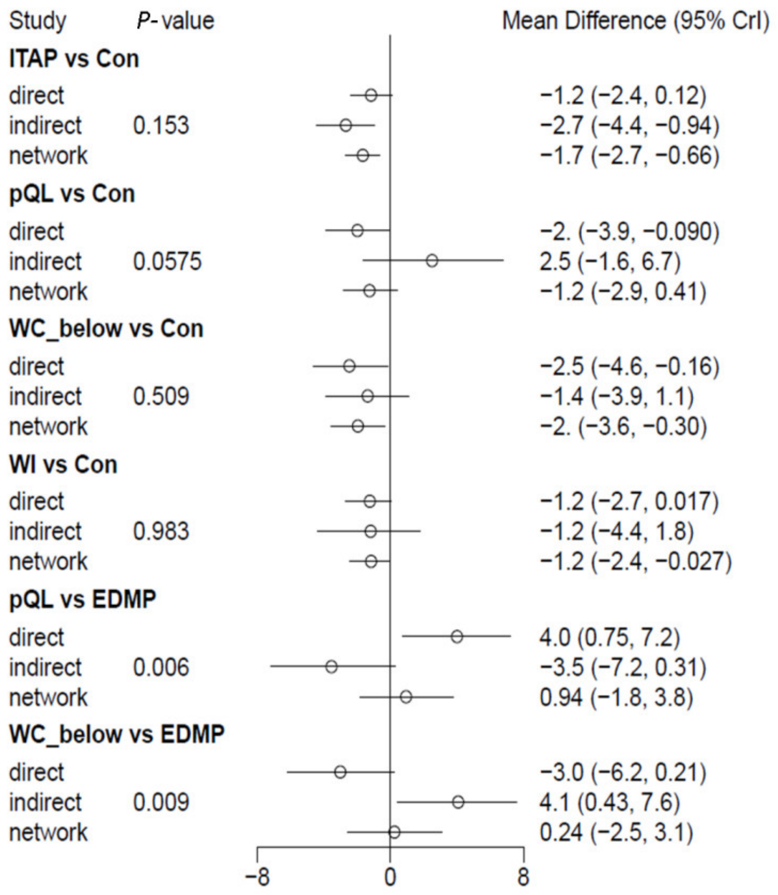

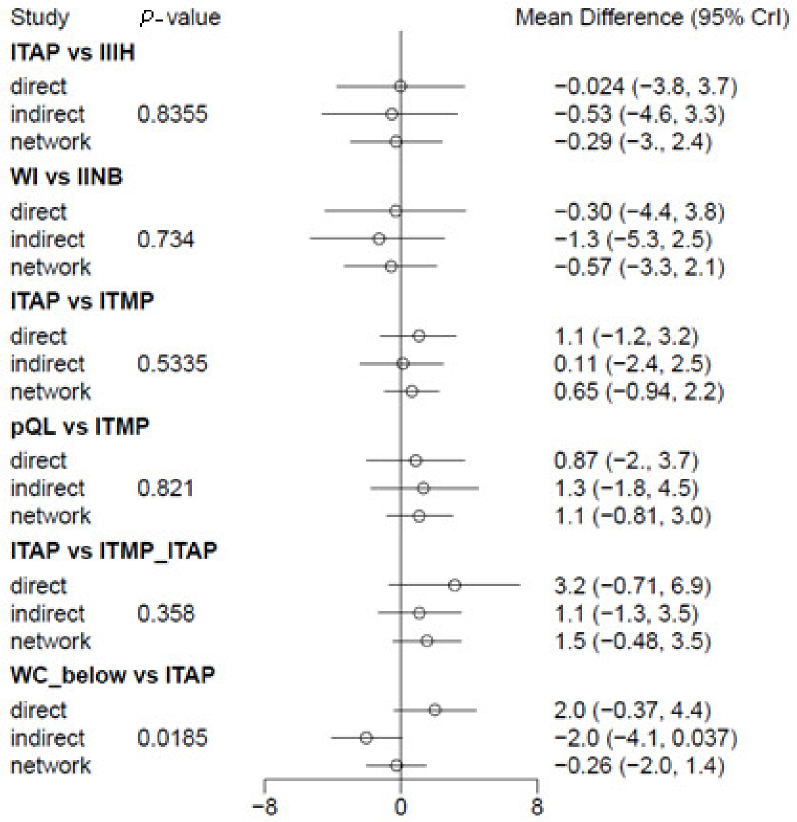

Figure S5. *Cont.*

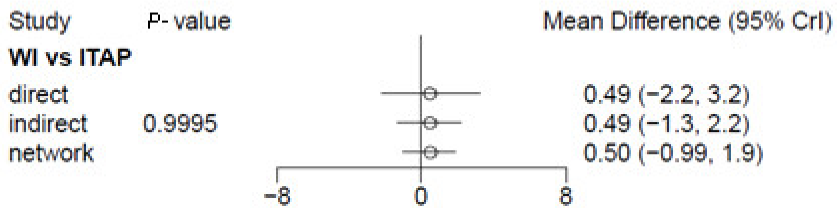

(**G**) Rankogram in random effect model.

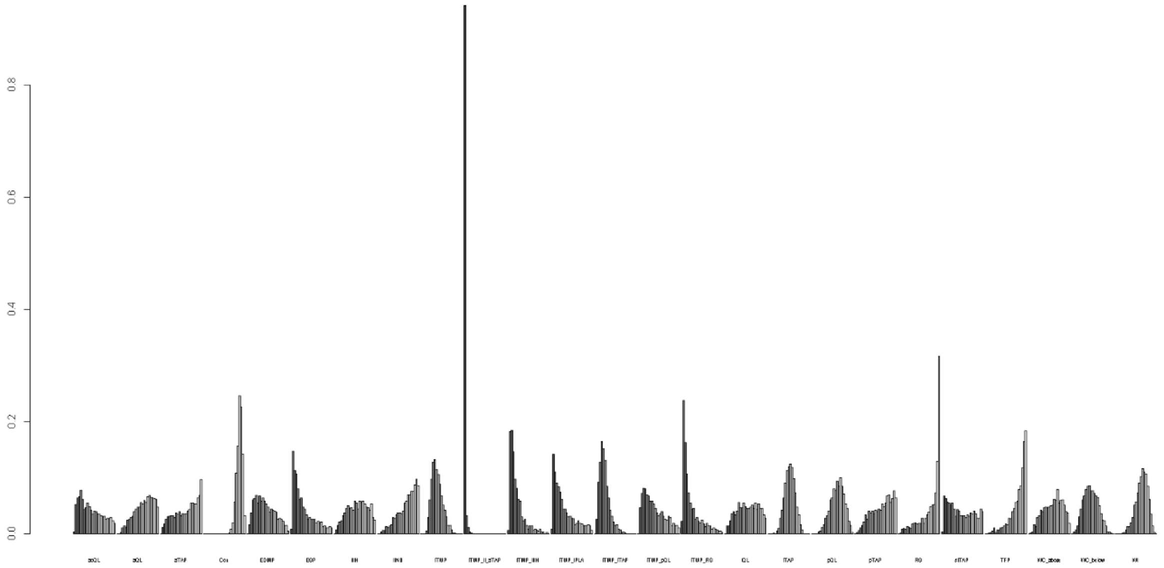

Figure S5. Pain at rest 6 h after surgery. (**A**) Trace plot and density plot in fixed effect model; (**B**) Trace plot and density plot in random effect model; (**C**) Gelman-Rubin-Brooks plot in fixed effect model; (**D**) Gelman-Rubin-Brooks plot in random effect model; (**E**) Forest plot comparing with control in random effect model; (**F**) Node splitting plot in random effect model; (**G**) Rankogram in random effect model.

(**A**) Trace plot and density plot in fixed effect model.

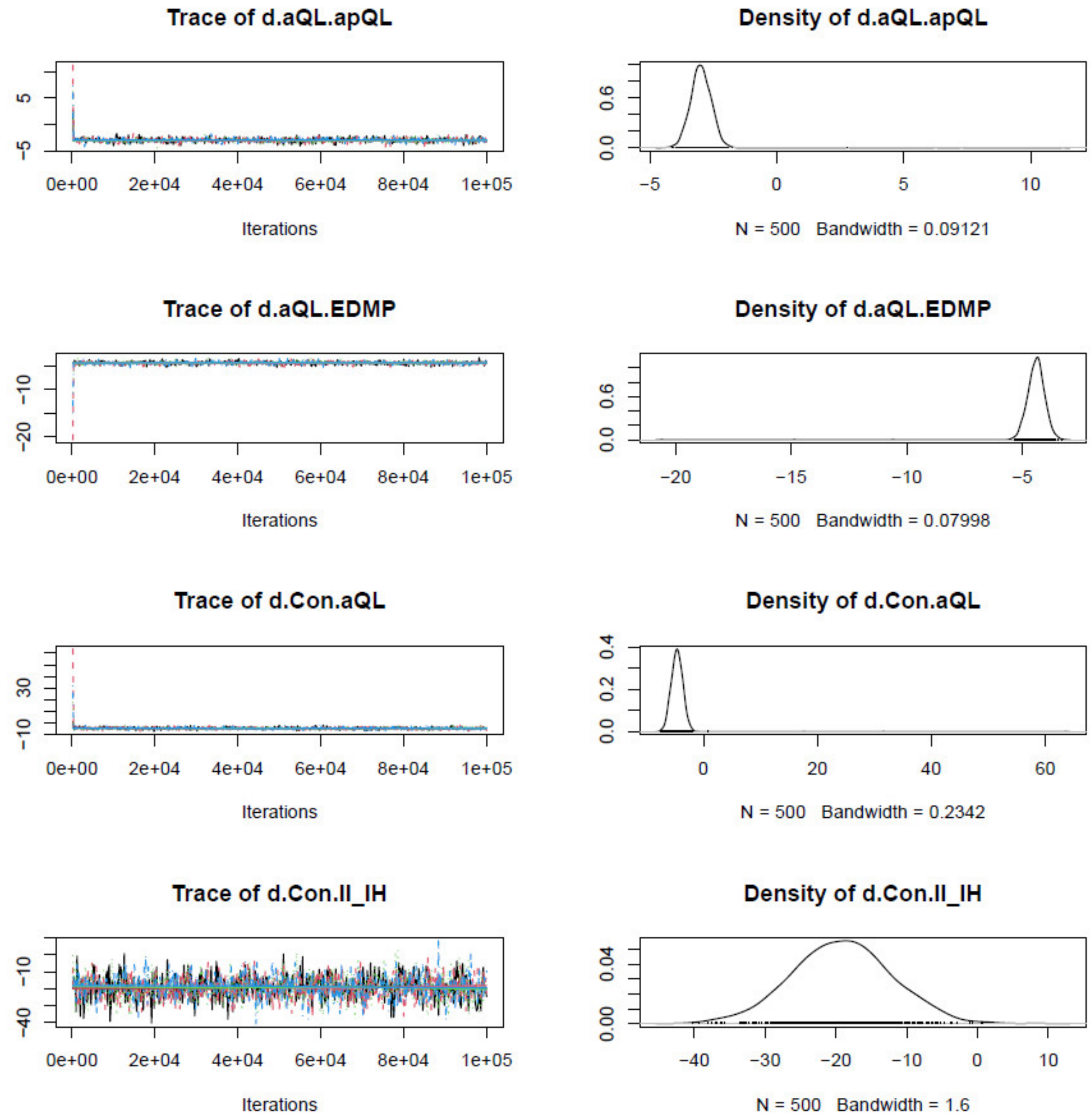

Figure S6. *Cont.*

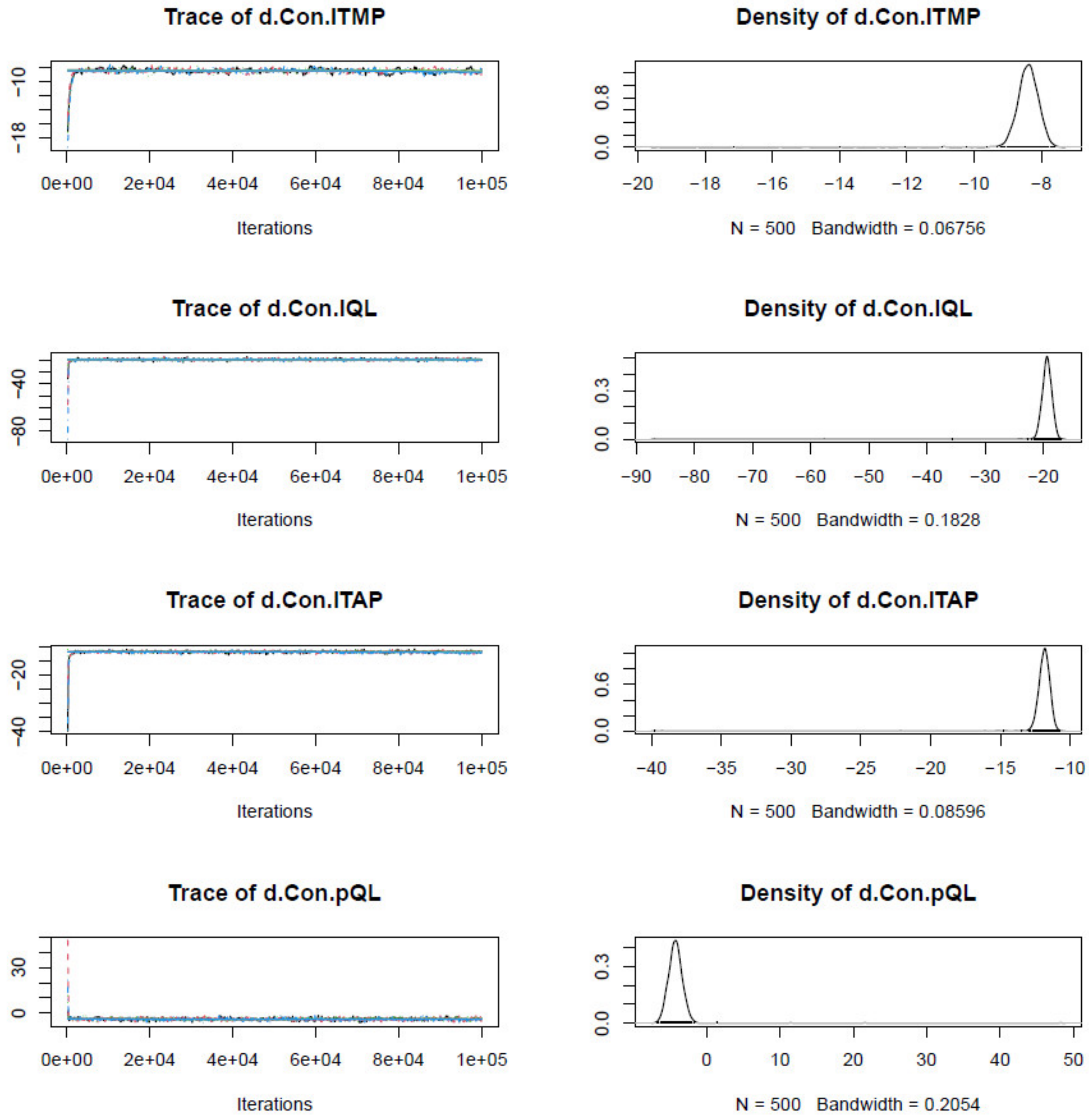

Figure S6. *Cont.*

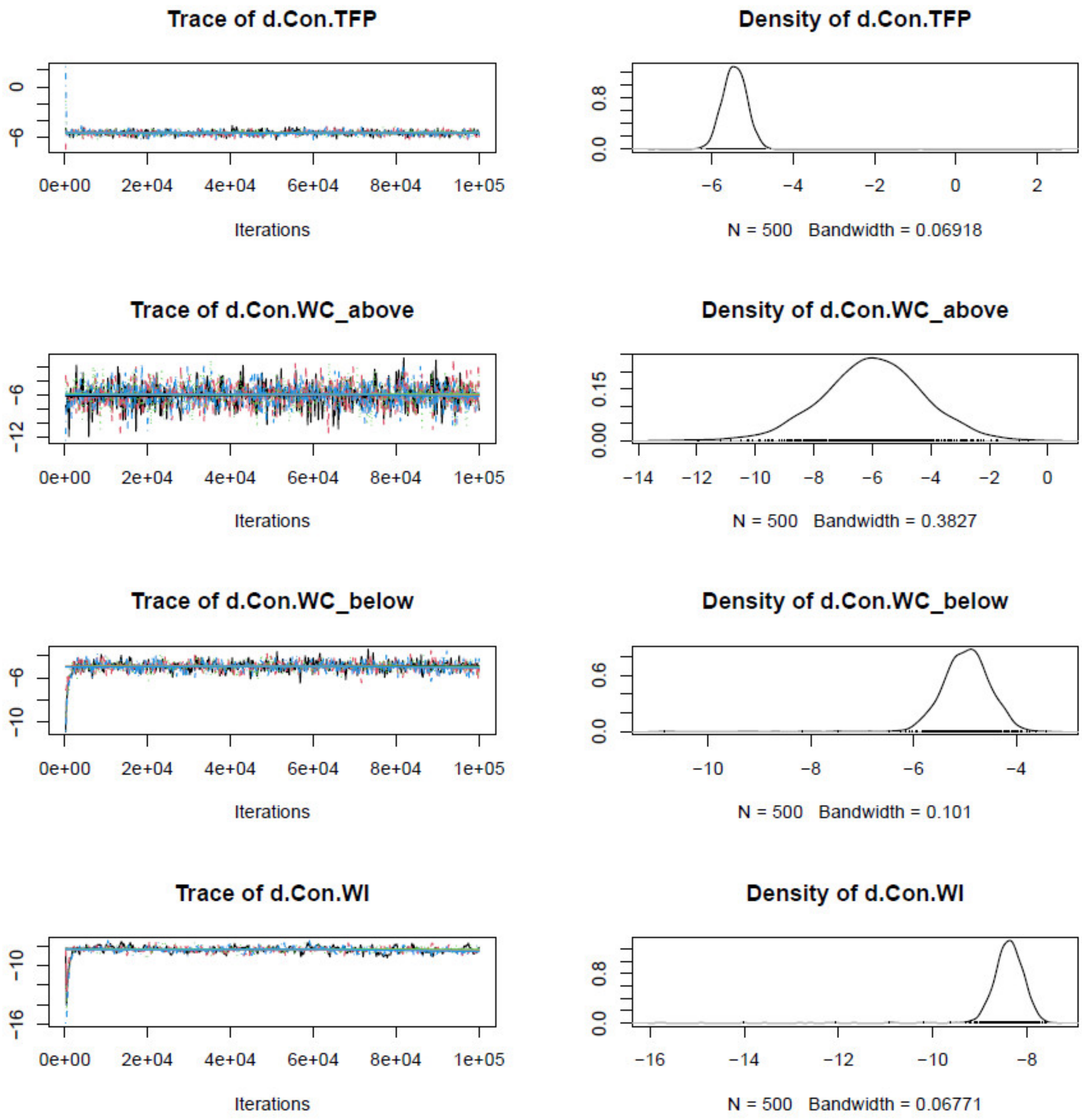

Figure S6. *Cont.*

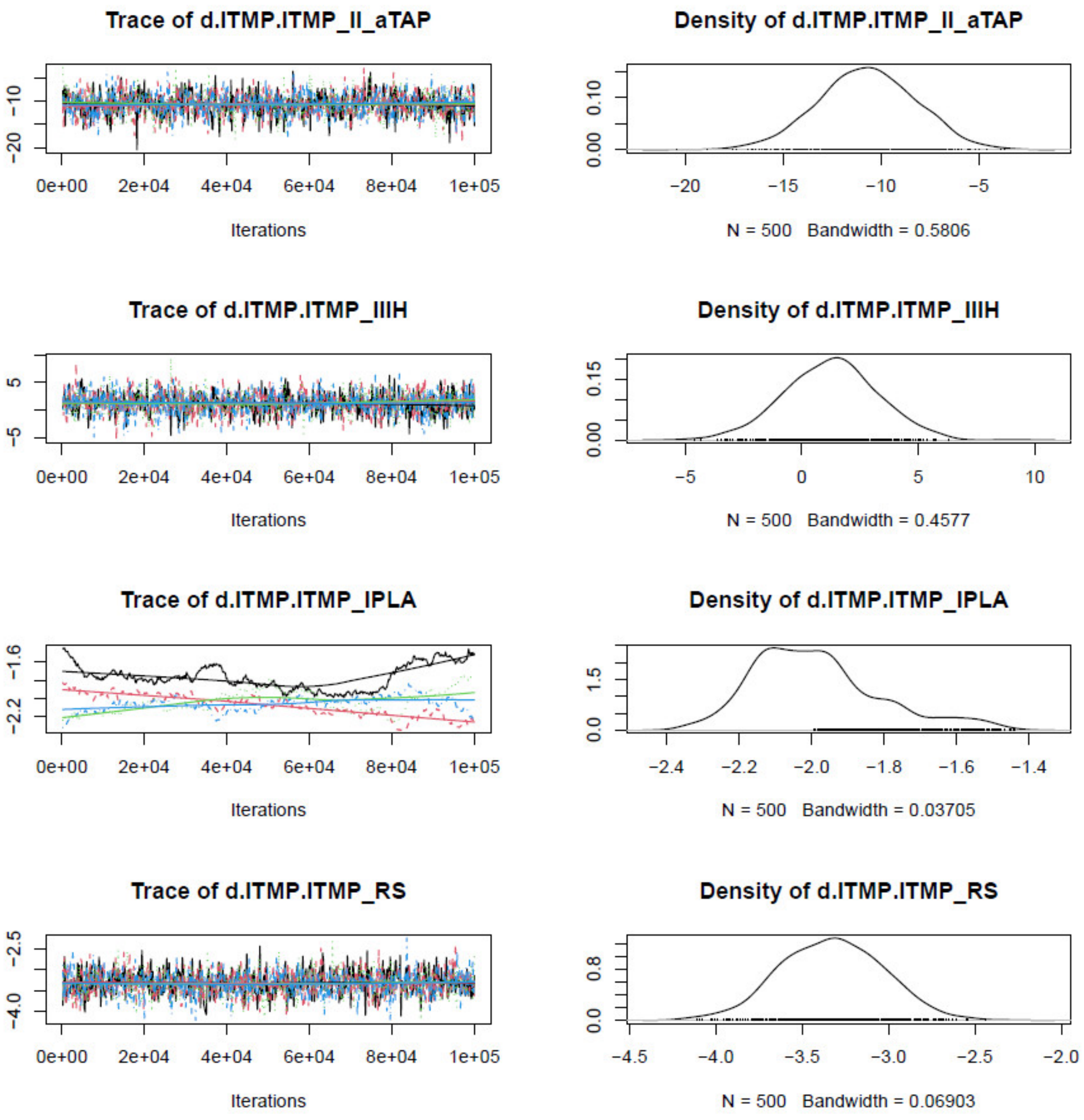

Figure S6. *Cont.*

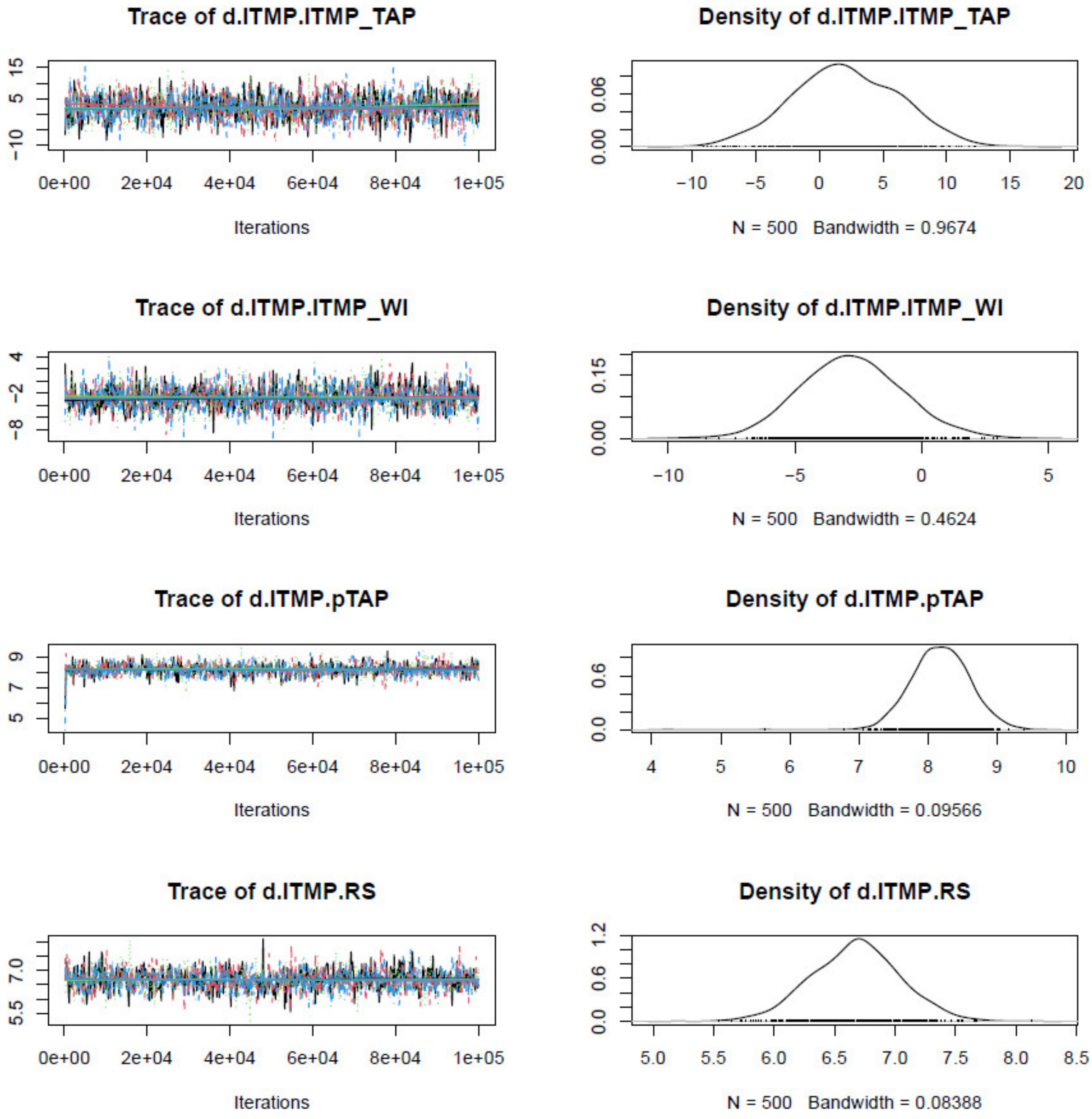

Figure S6. *Cont.*

(**B**) Trace plot and density plot in random effect model.

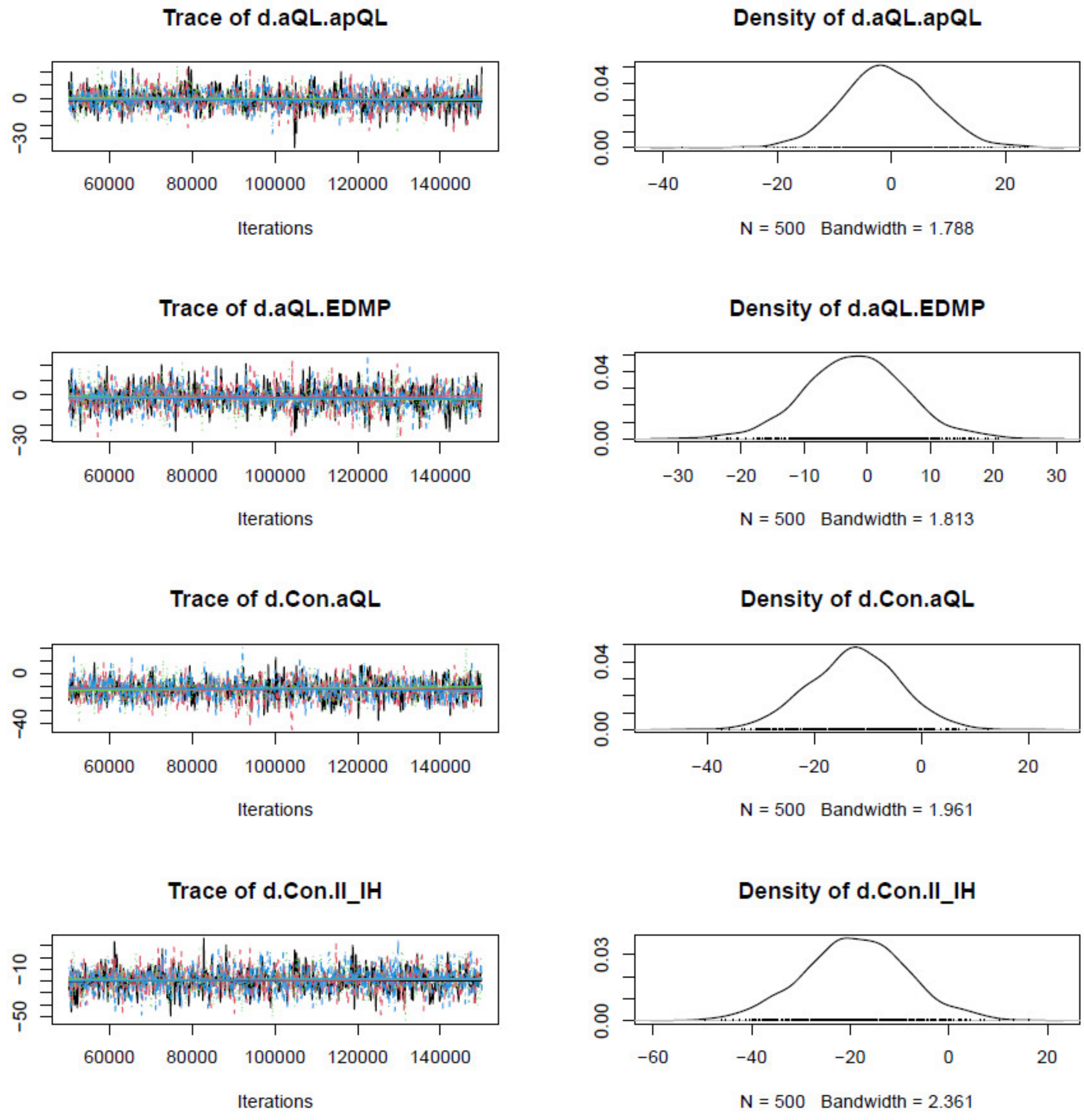

Figure S6. *Cont.*

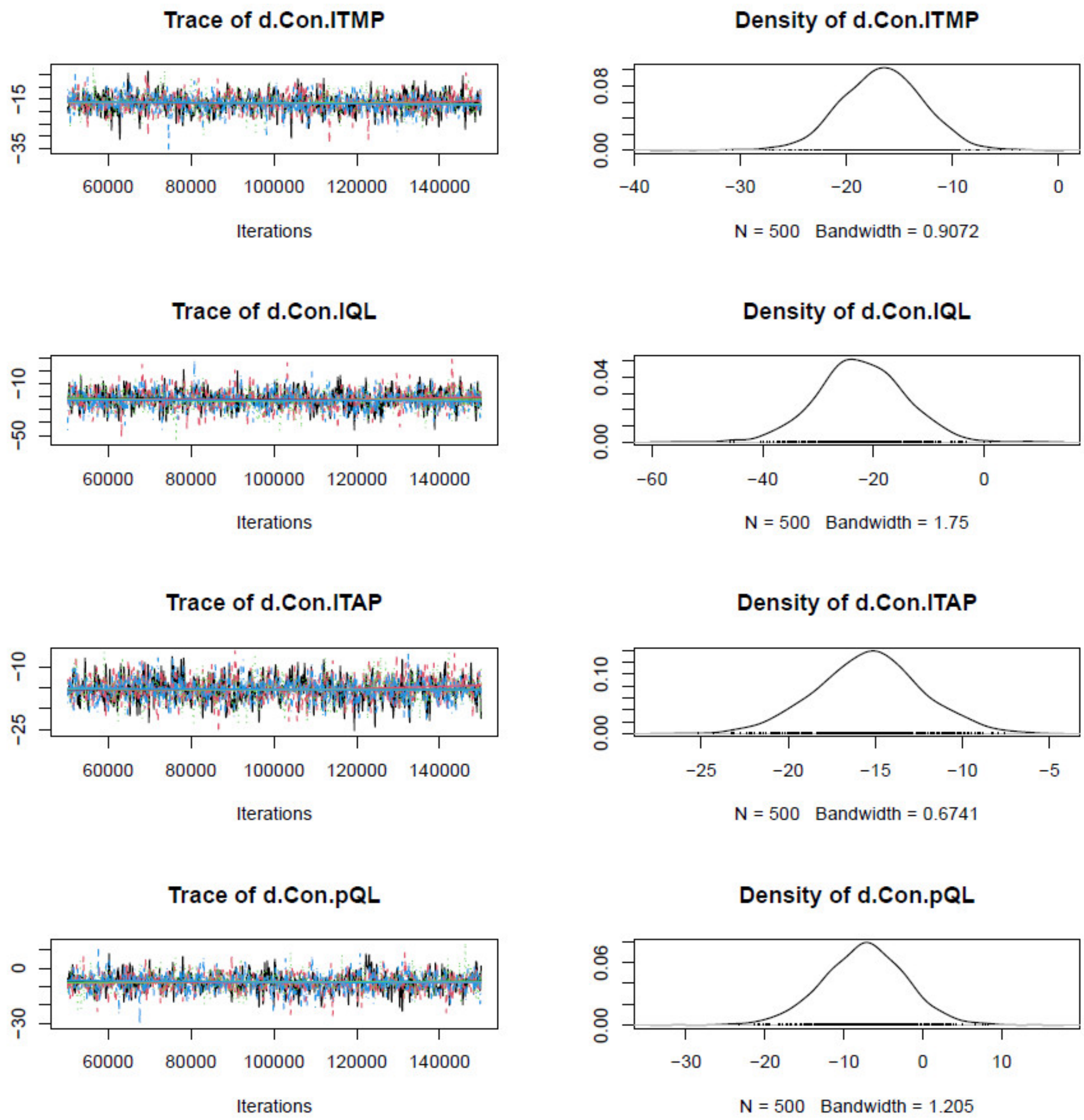

Figure S6. *Cont.*

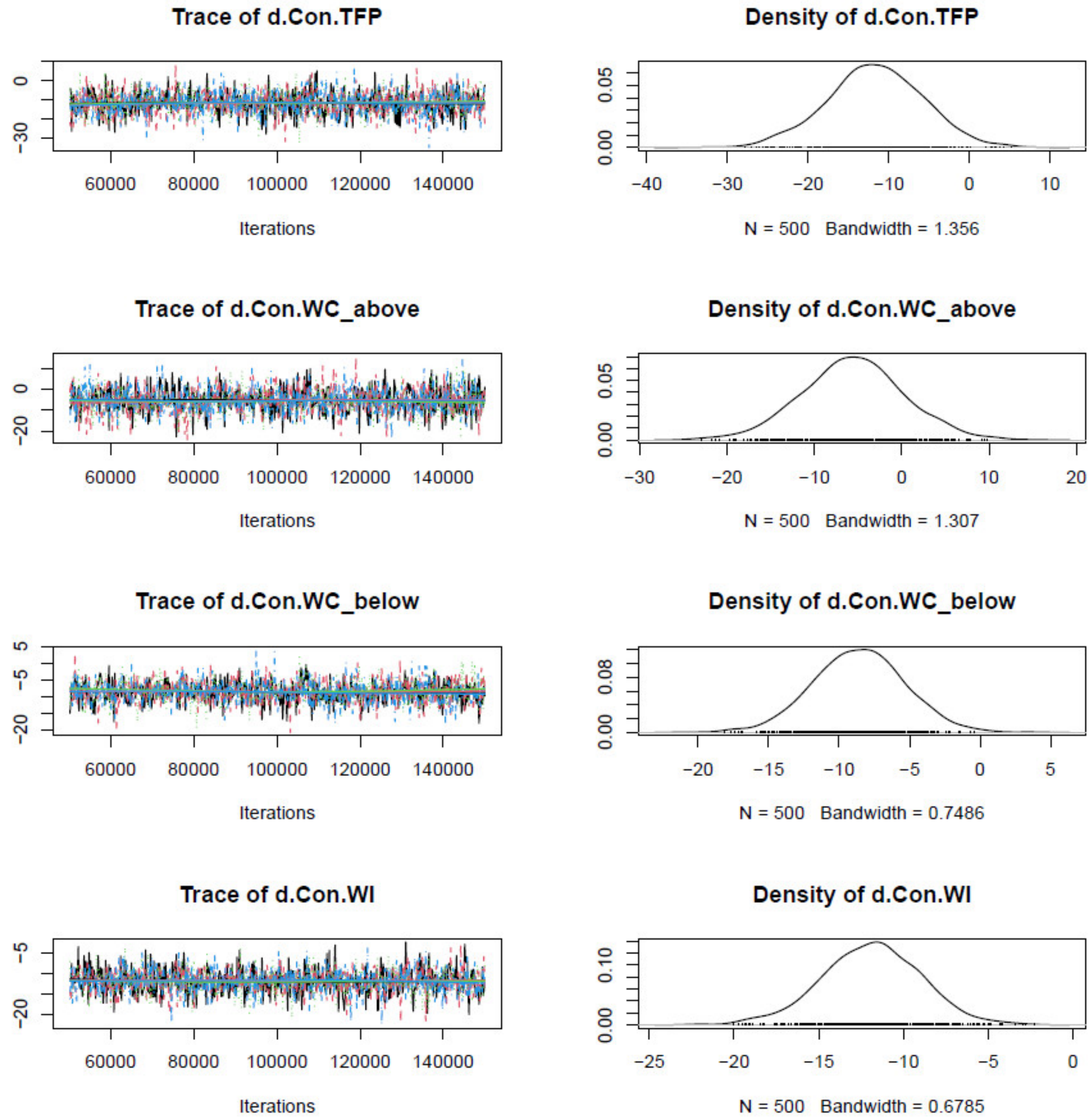

Figure S6. *Cont.*

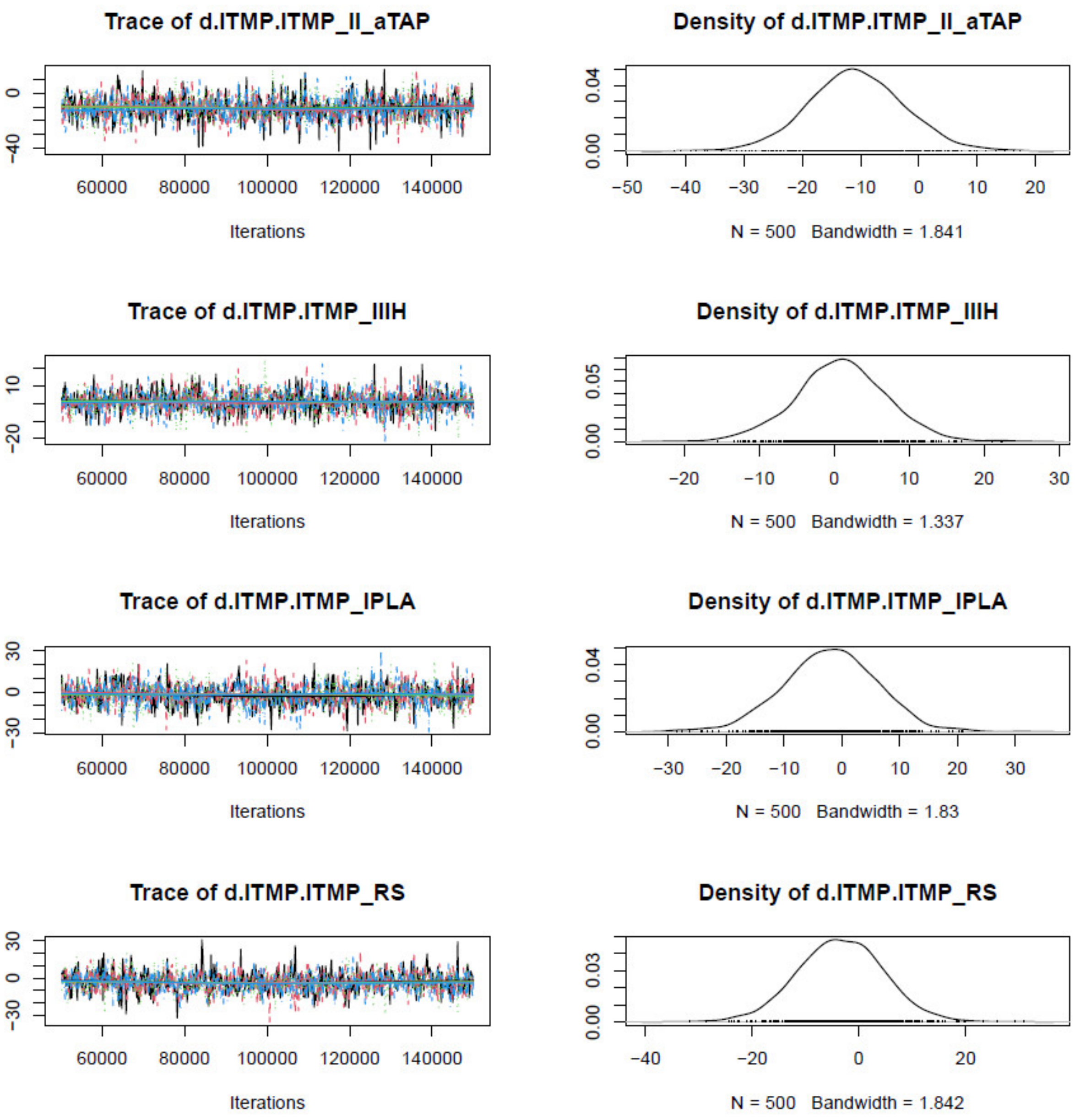

Figure S6. *Cont.*

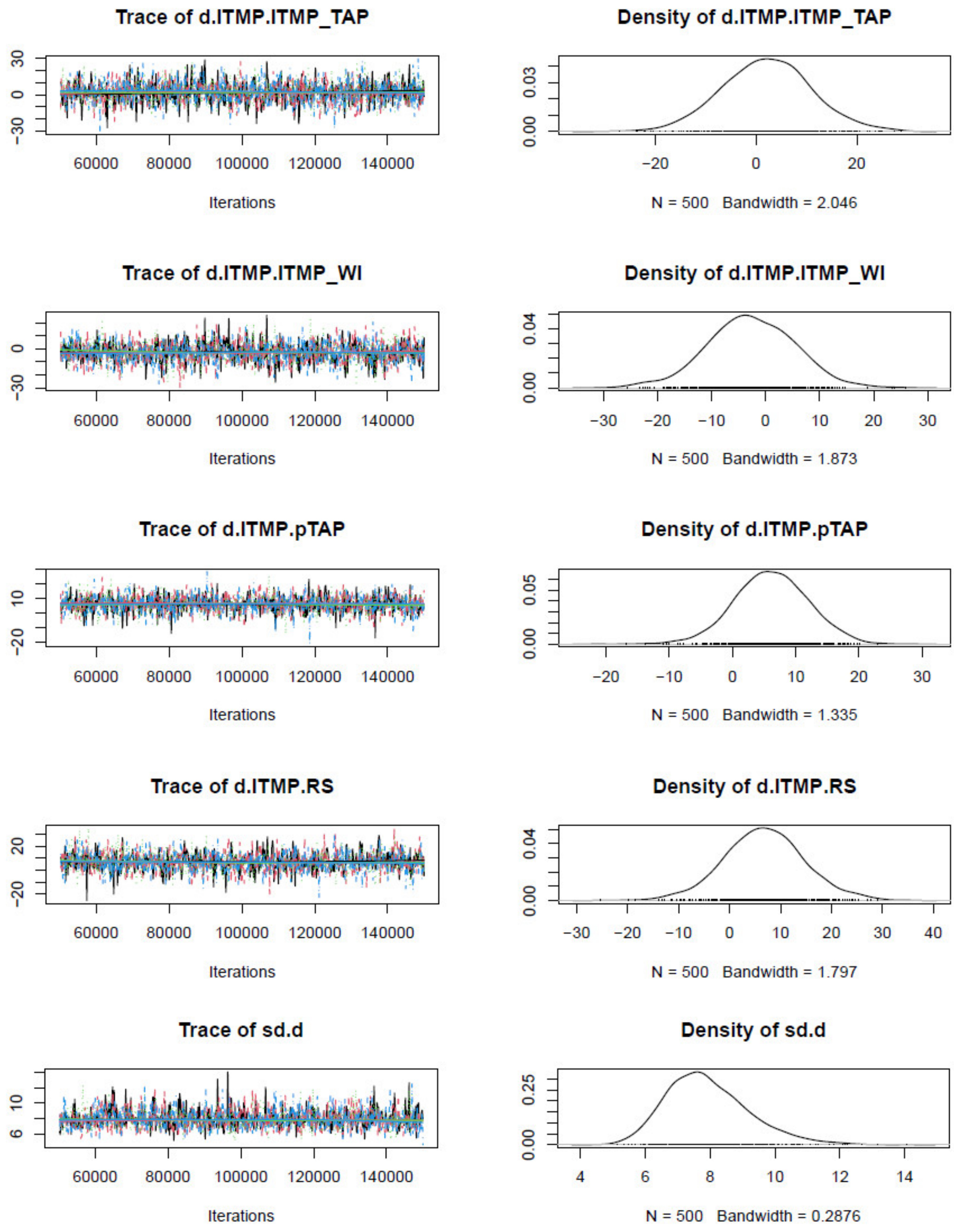

Figure S6. *Cont.*

(**C**) Gelman-Rubin-Brooks plot in fixed effect model.

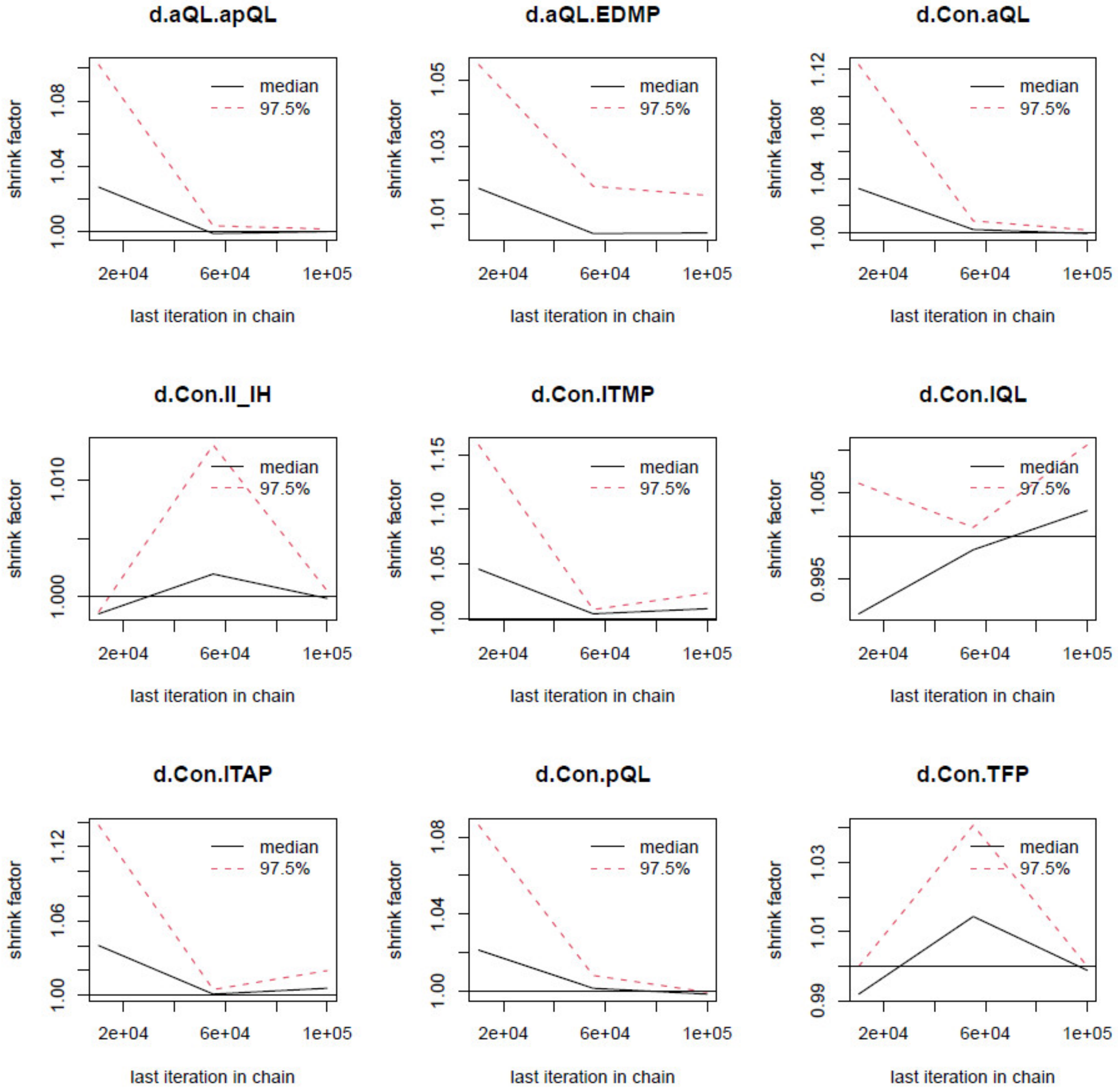

Figure S6. *Cont.*

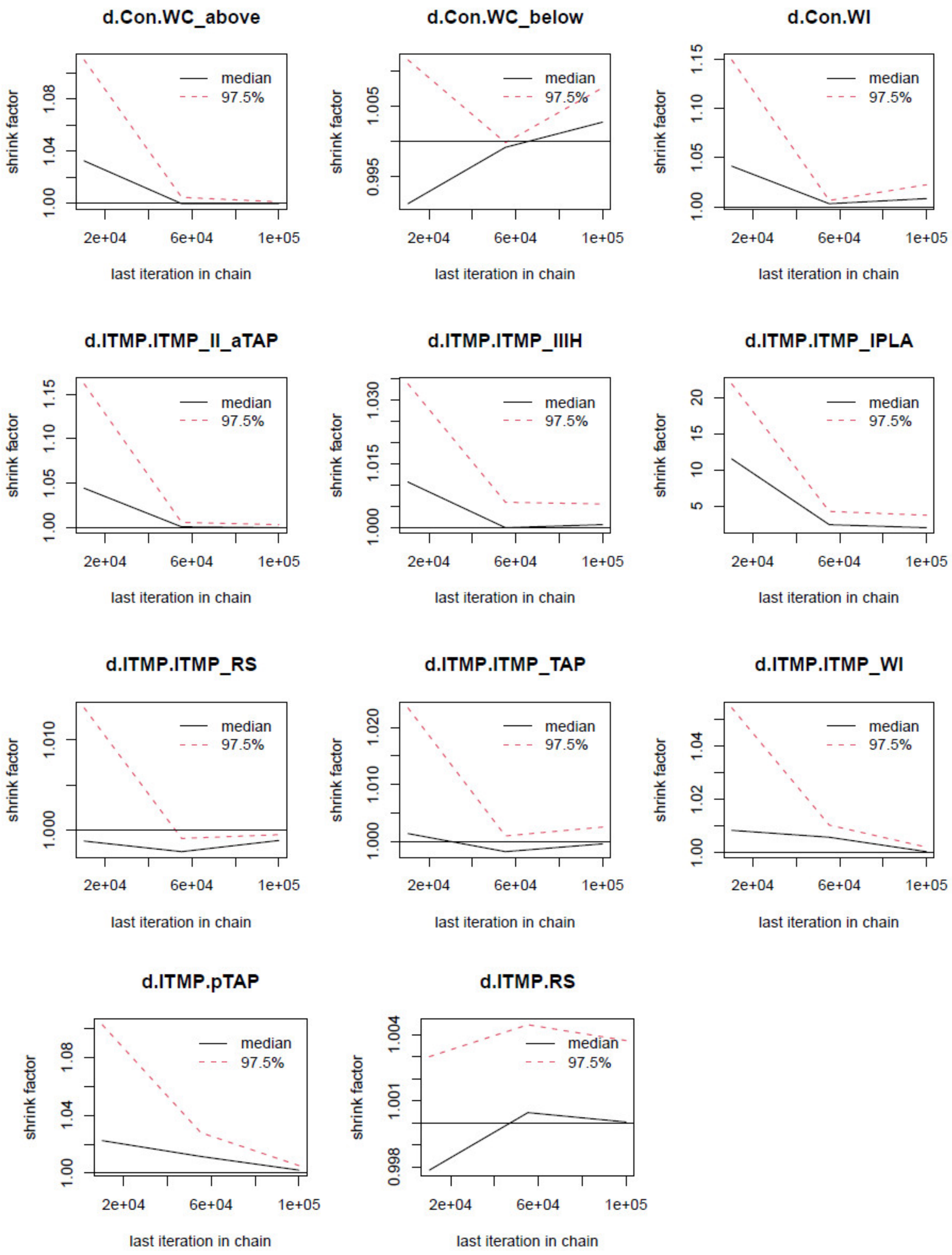

Figure S6. *Cont.*

(**D**) Gelman-Rubin-Brooks plot in random effect model.

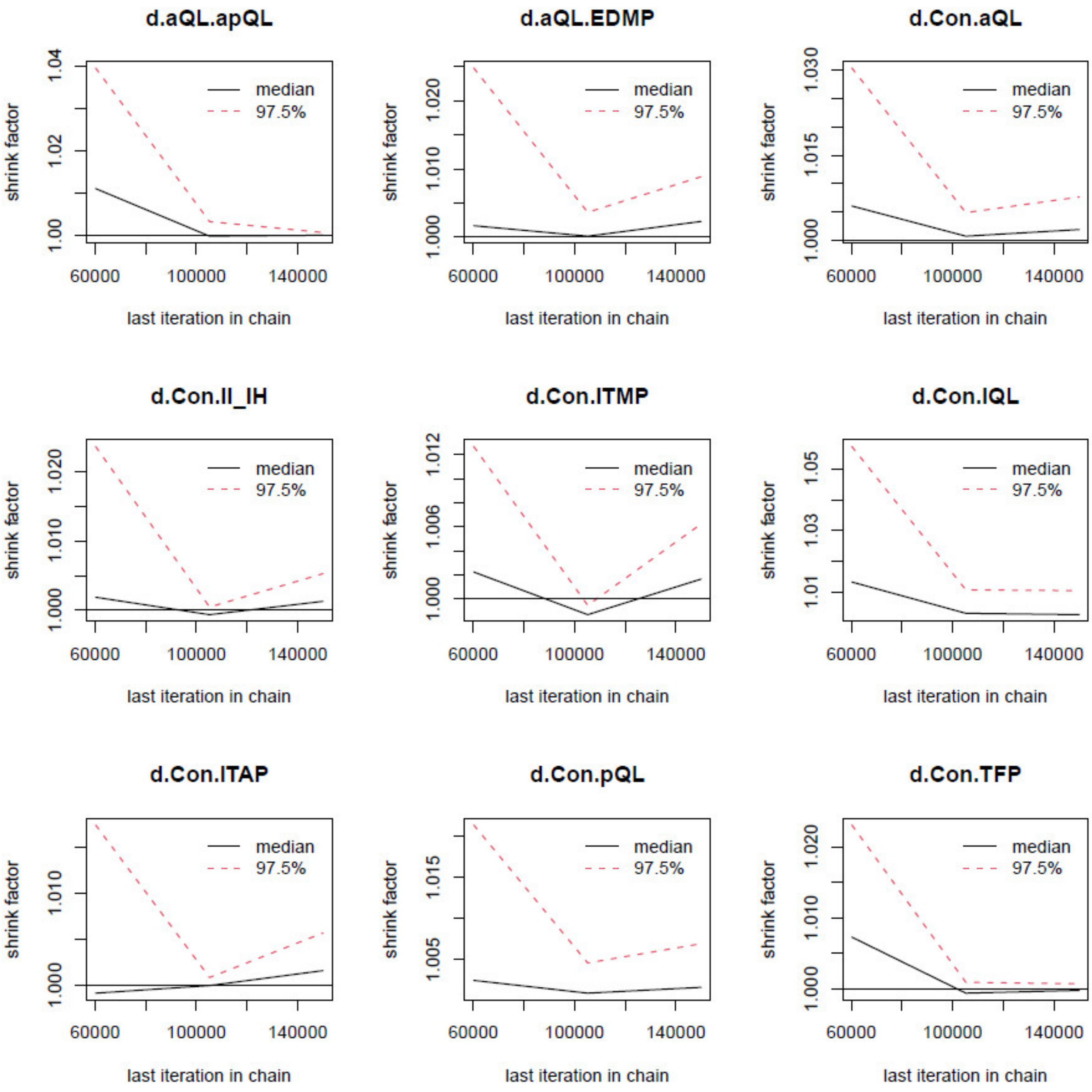

Figure S6. *Cont.*

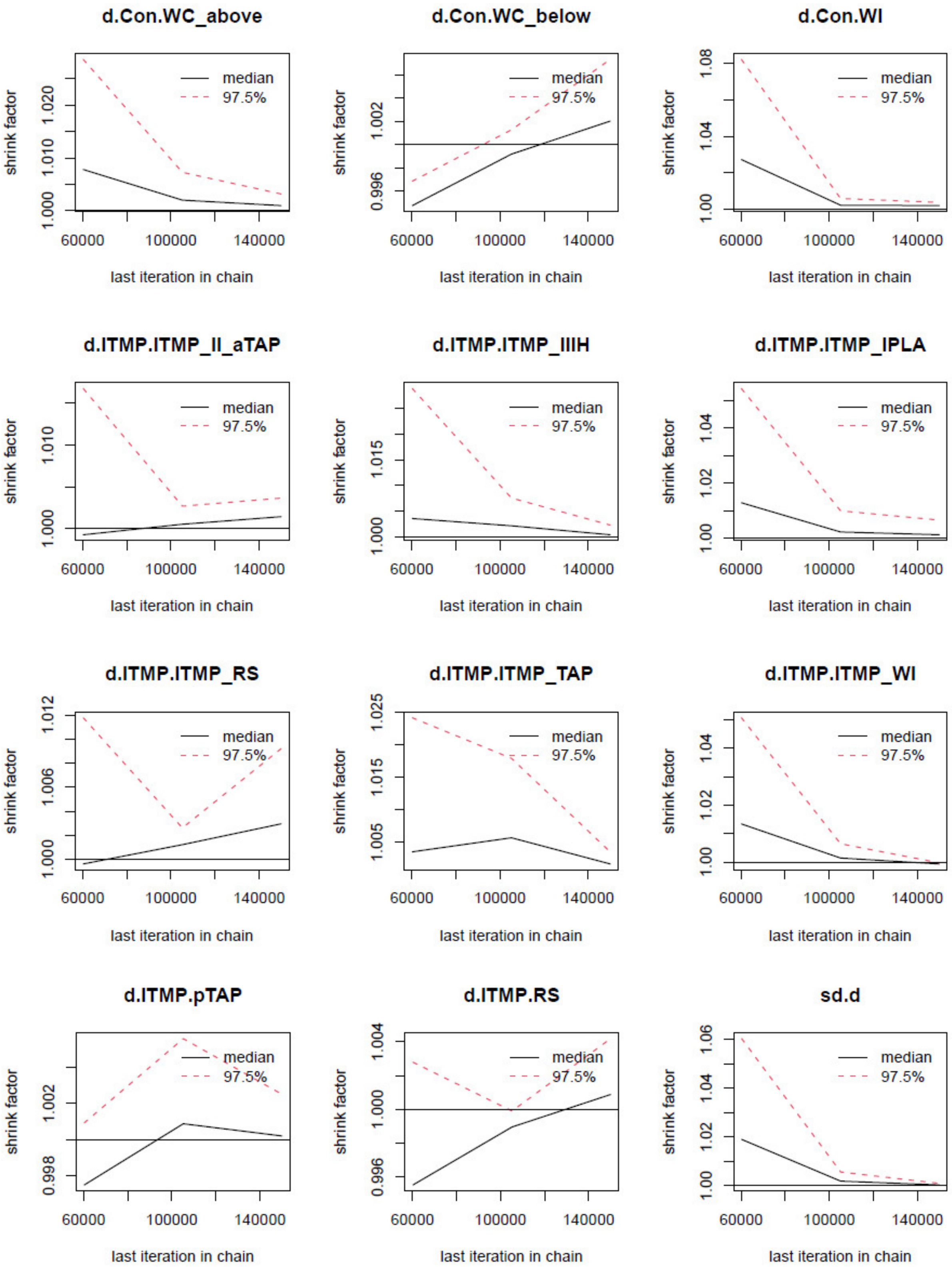

Figure S6. *Cont.*

(**E**) Forest plot comparing with control in random effect model.

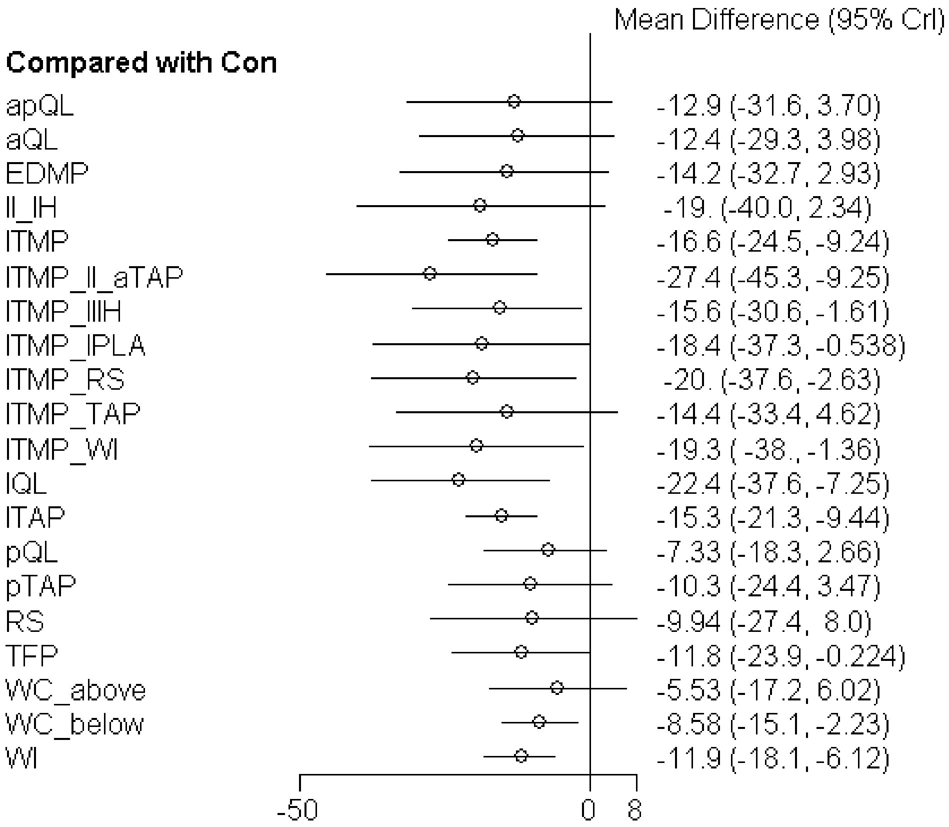

Figure S6. *Cont.*

(**F**) Node splitting plot in random effect model.

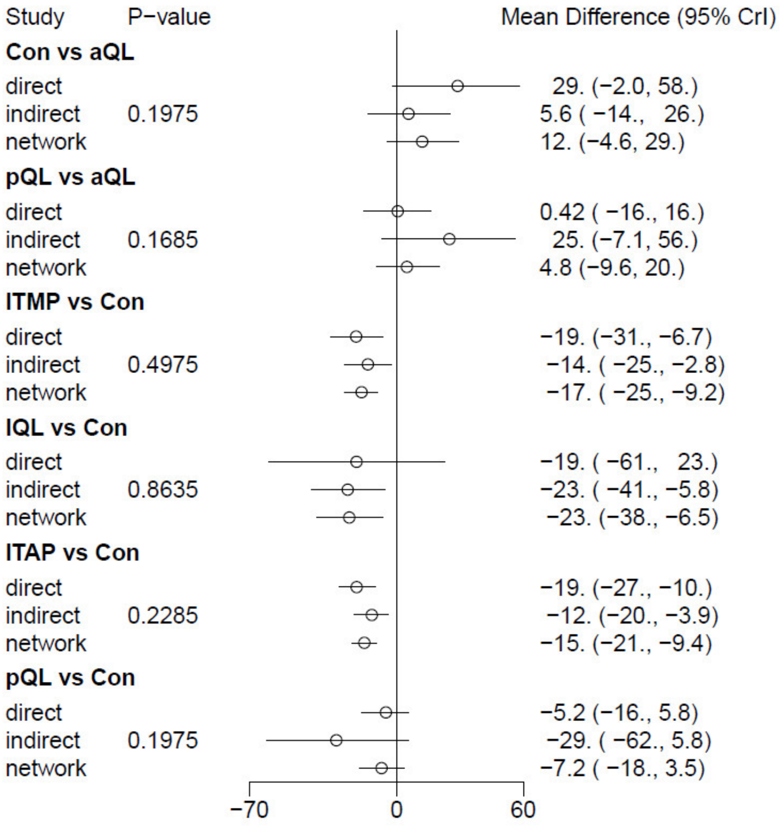

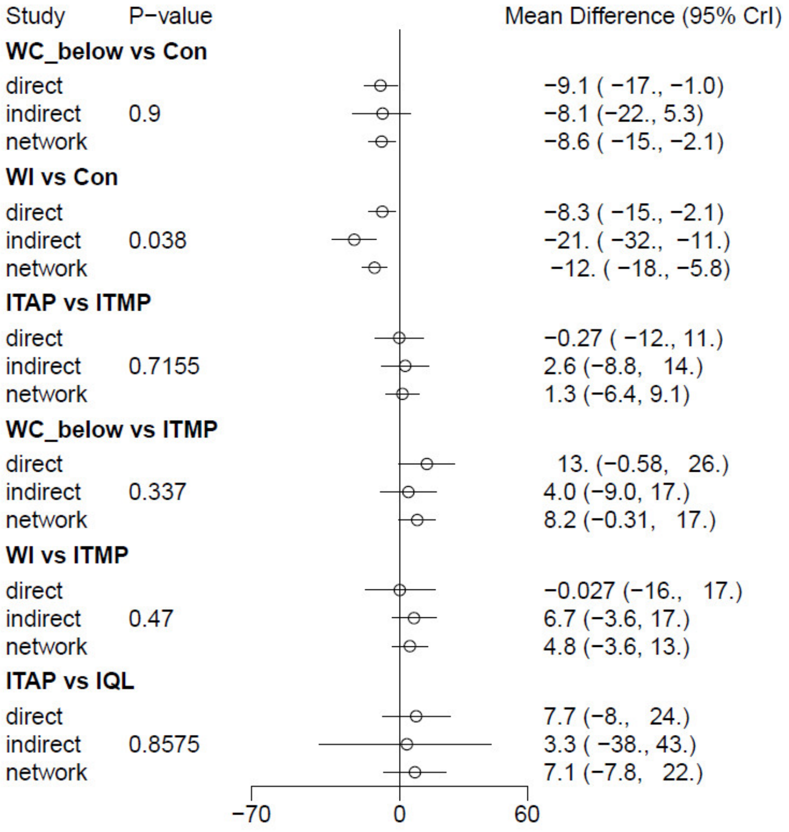

Figure S6. *Cont.*

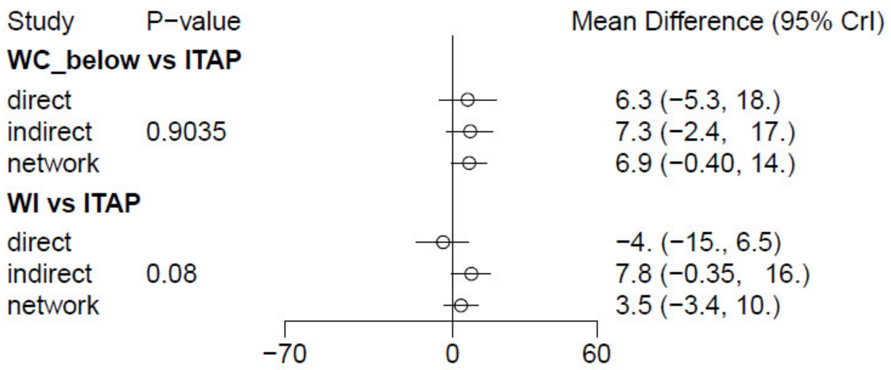

(**G**) Rankogram in random effect model.

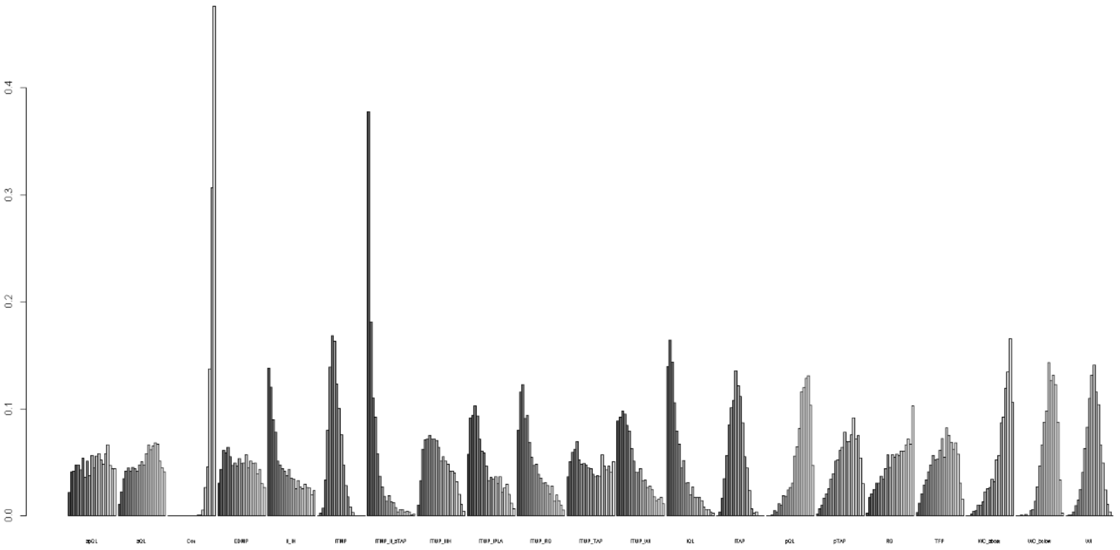

Figure S6. Postoperative cumulative 24 h morphine equivalent consumption. (**A**) Trace plot and density plot in fixed effect model; (**B**) Trace plot and density plot in random effect model; (**C**) Gelman-Rubin-Brooks plot in fixed effect model; (**D**) Gelman-Rubin-Brooks plot in random effect model; (**E**) Forest plot comparing with control in random effect model; (**F**) Node splitting plot in random effect model; (**G**) Rankogram in random effect model.

(**A**) Trace plot and density plot in fixed effect model.

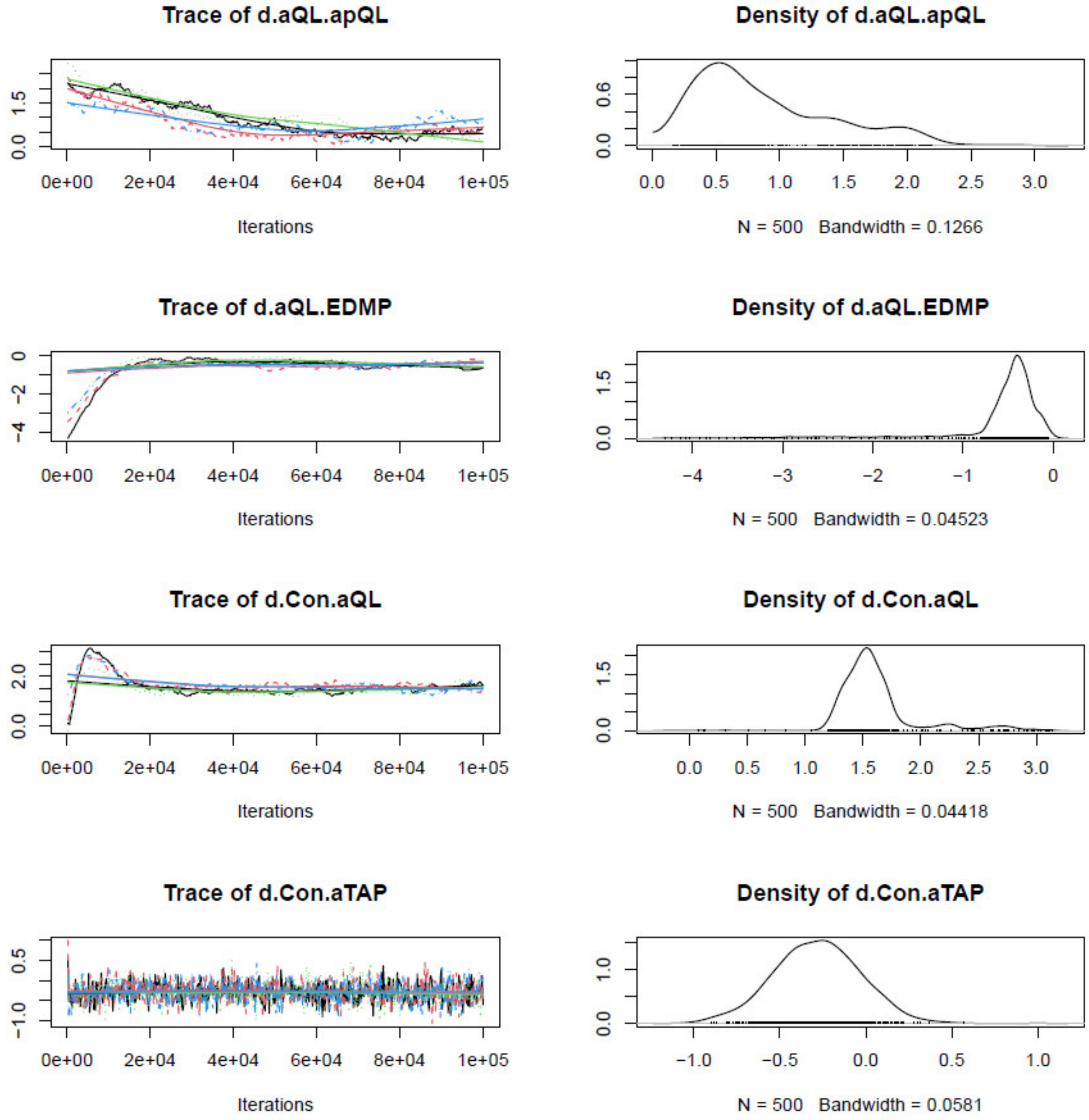

Figure S7. *Cont.*

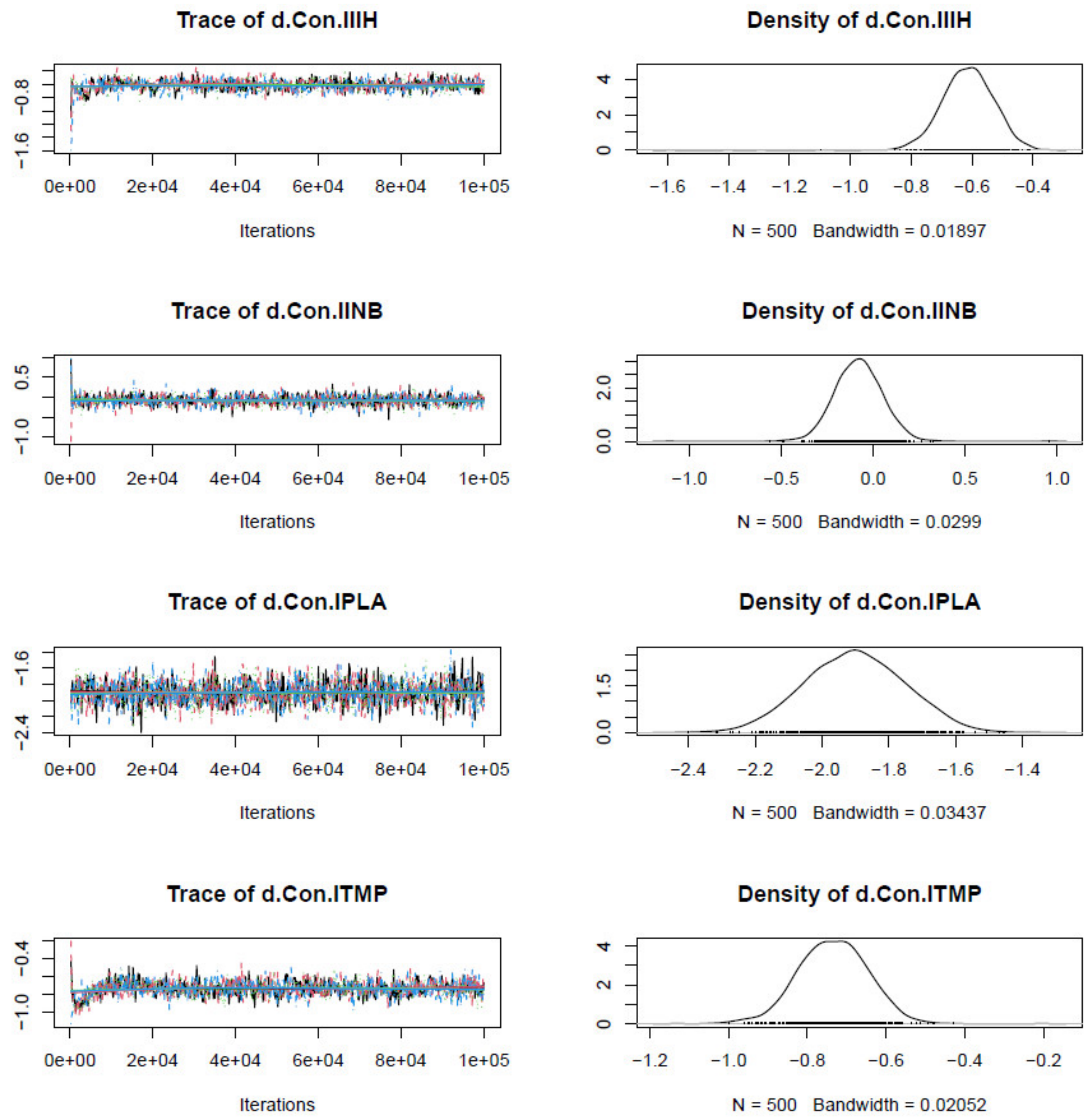

Figure S7. *Cont.*

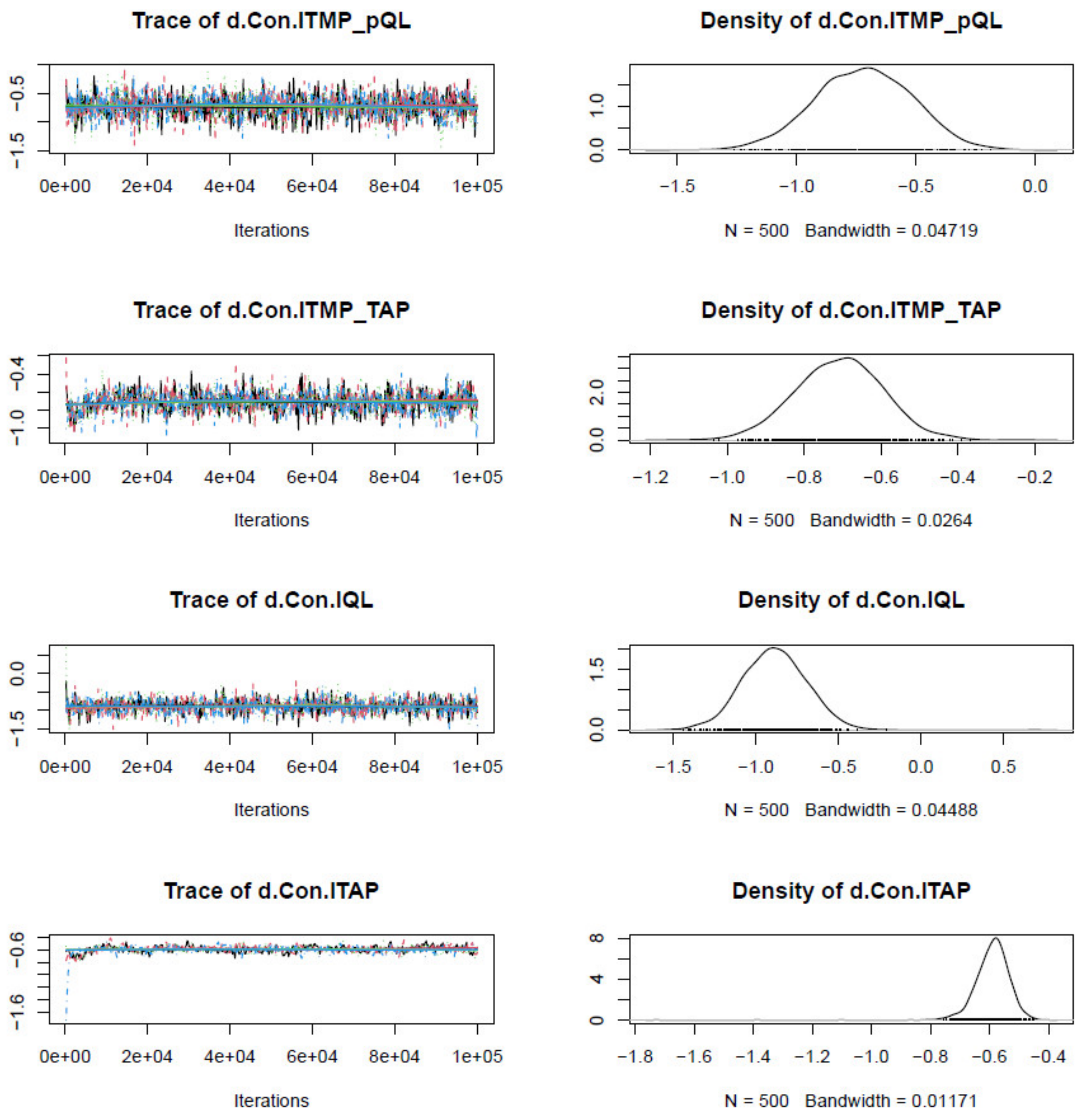

Figure S7. *Cont.*

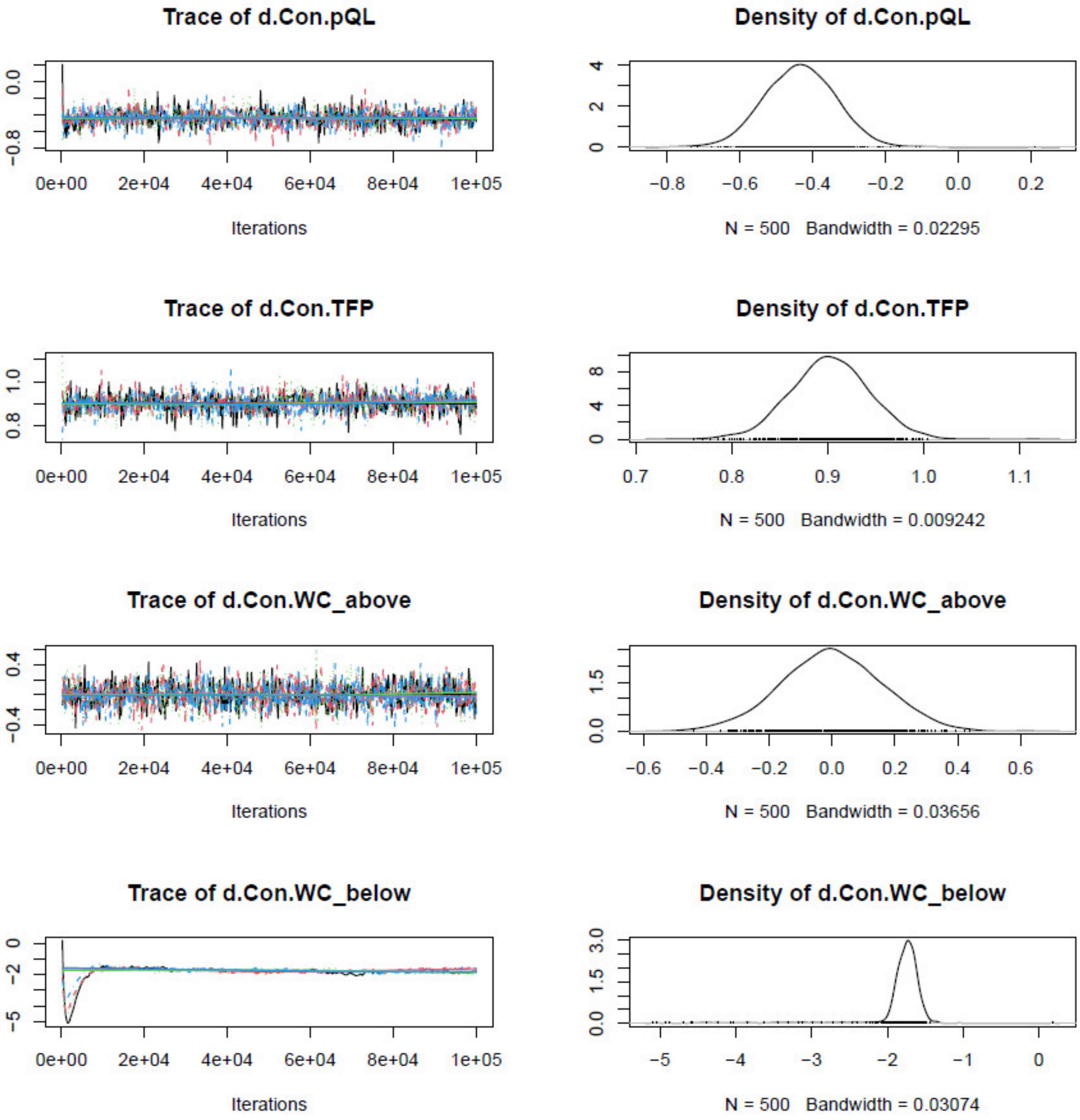

Figure S7. *Cont.*

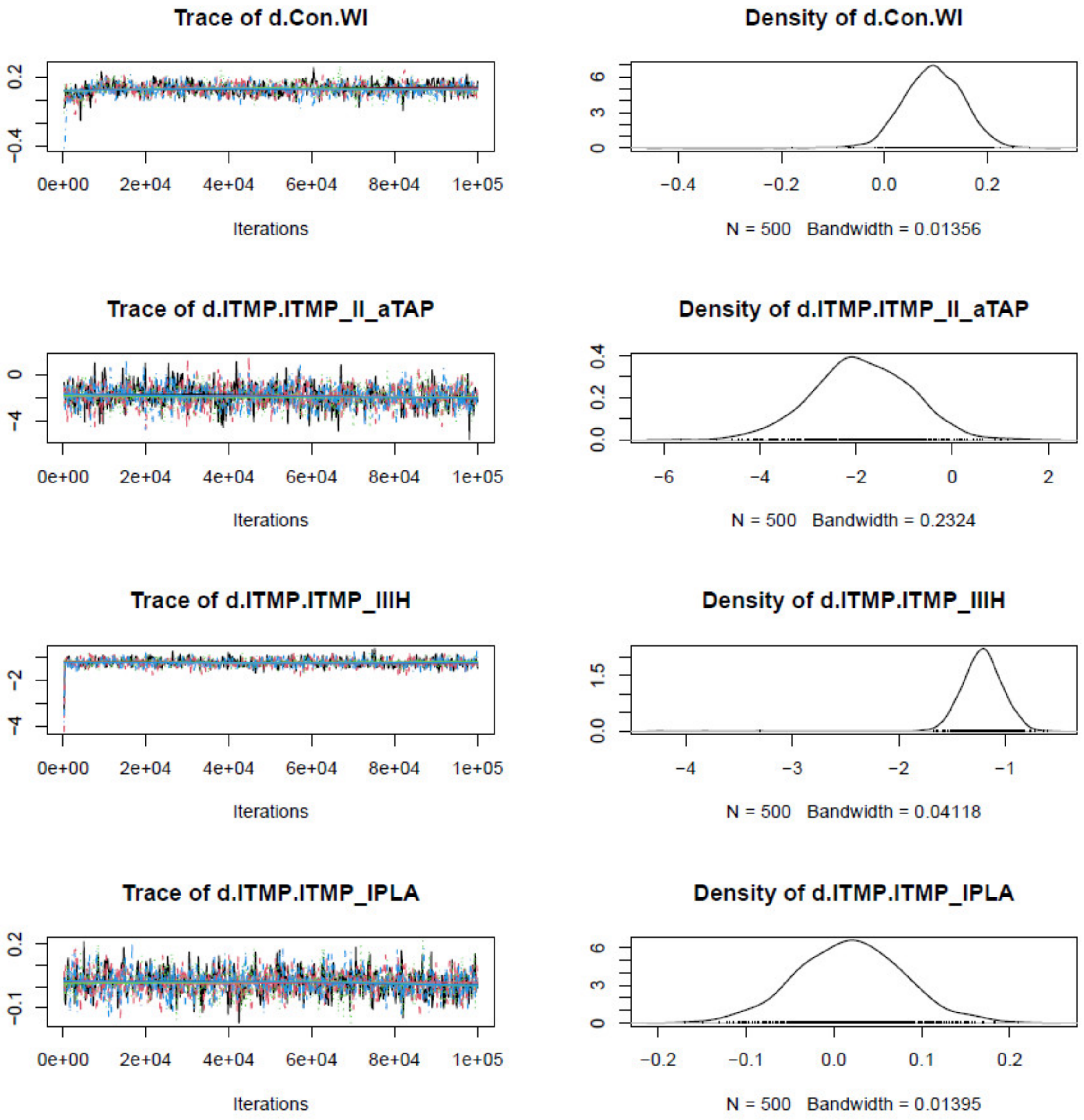

Figure S7. *Cont.*

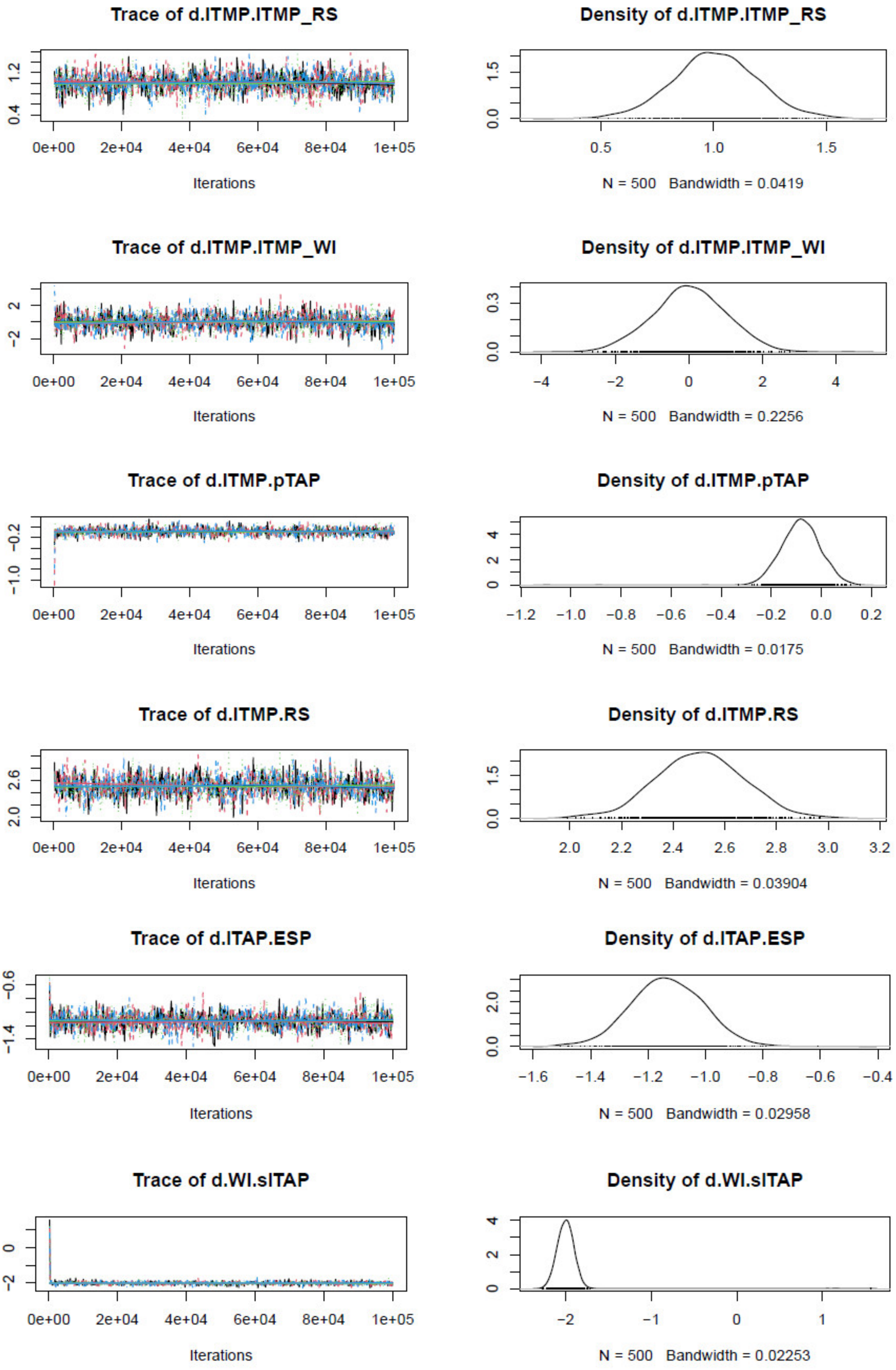

Figure S7. *Cont.*

(**B**) Trace plot and density plot in random effect model.

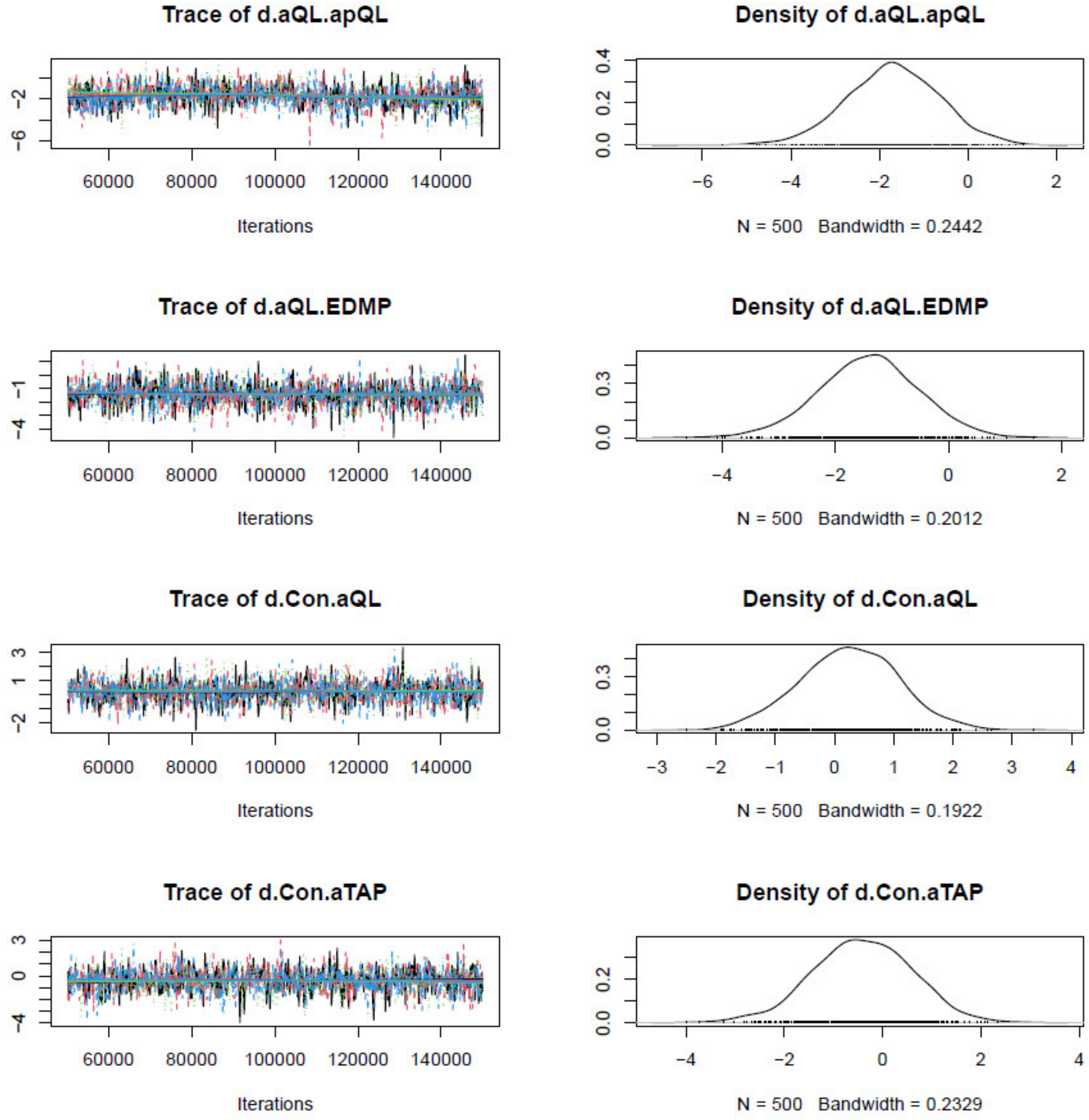

Figure S7. *Cont.*

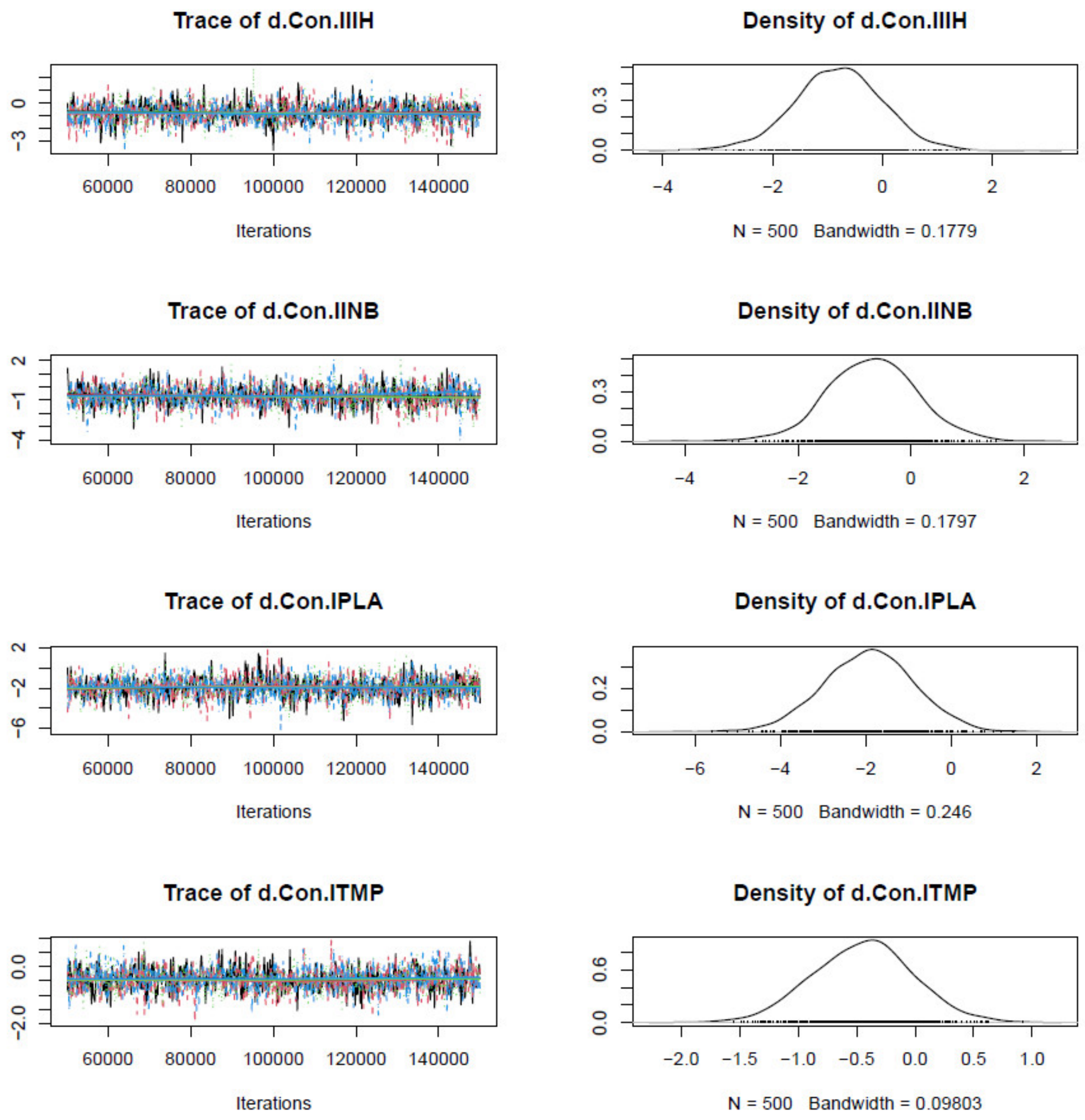

Figure S7. *Cont.*

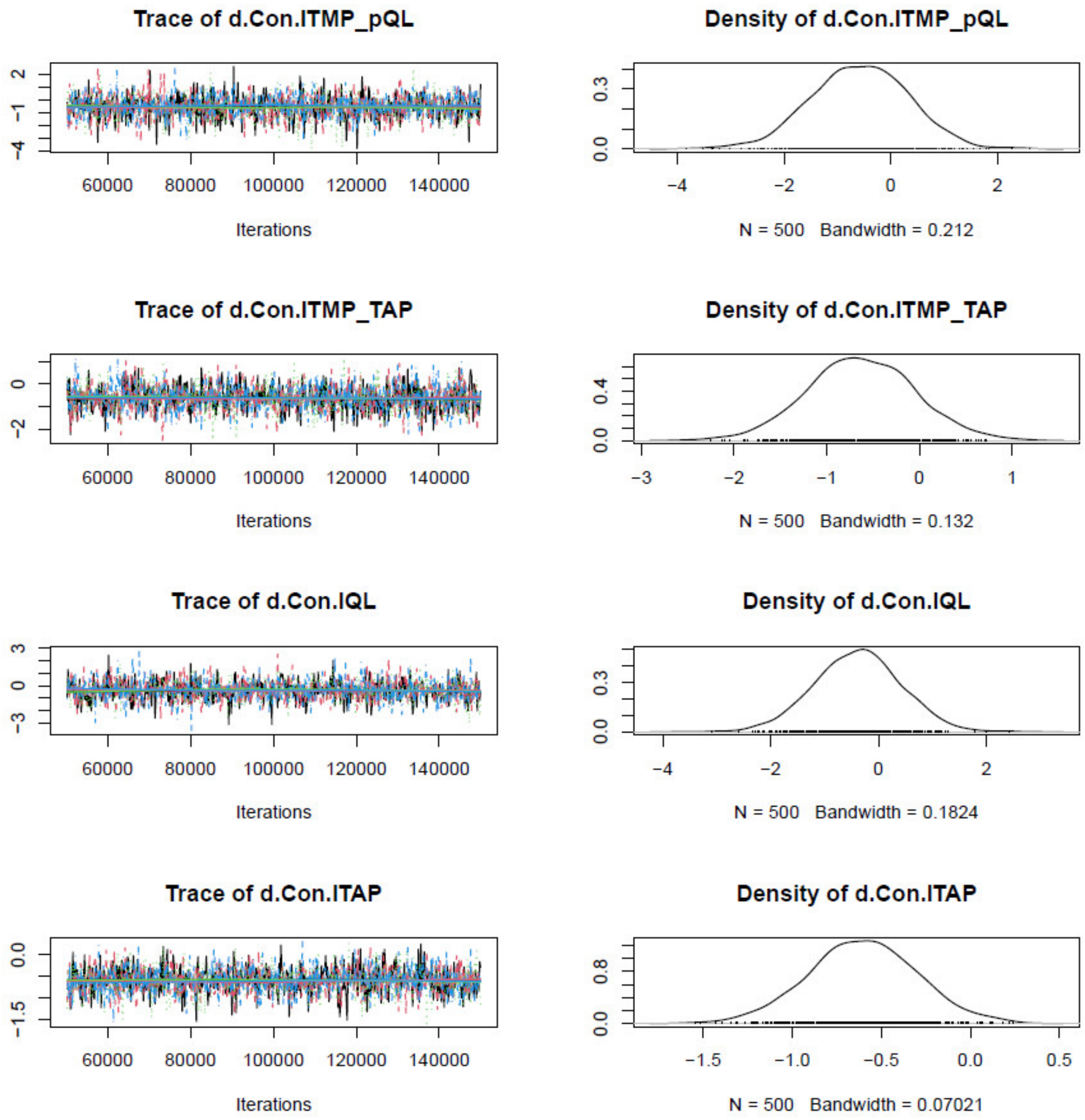

Figure S7. *Cont.*

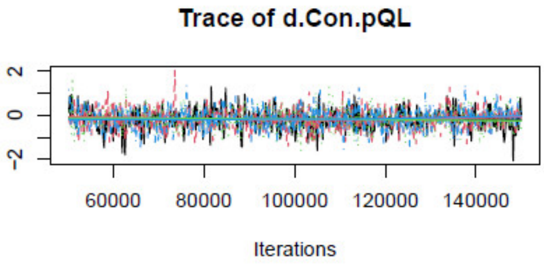
Trace of d.Con.pQL
2
0
−2
60000
80000
100000
120000
140000
Iterations

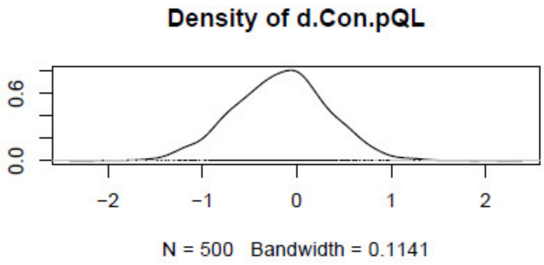
Density of d.Con.pQL
0.6
0.0
−2
−1
0
1
2
N = 500 Bandwidth = 0.1141

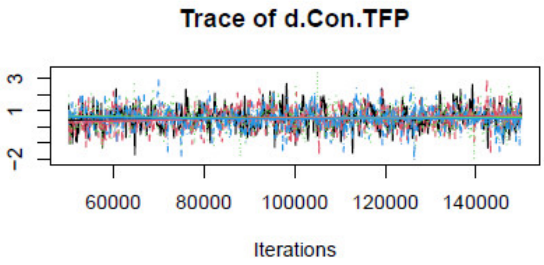
Trace of d.Con.TFP
3
1
−2
60000
80000
100000
120000
140000
Iterations

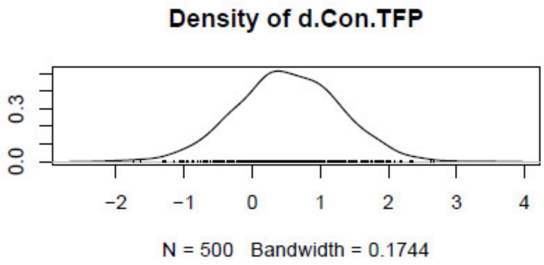
Density of d.Con.TFP
0.3
0.0
−2
−1
0
1
2
3
4
N = 500 Bandwidth = 0.1744

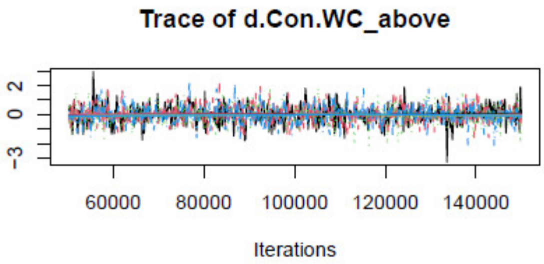
Trace of d.Con.WC_above
2
0
−3
60000
80000
100000
120000
140000
Iterations

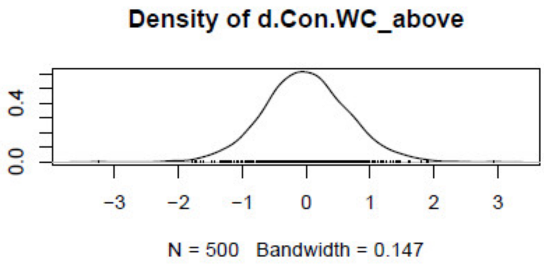
Density of d.Con.WC_above
0.4
0.0
−3
−2
−1
0
1
2
3
N = 500 Bandwidth = 0.147

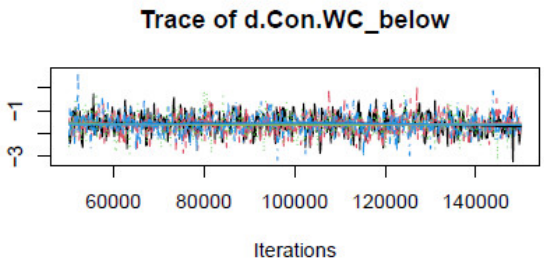
Trace of d.Con.WC_below
−1
−3
60000
80000
100000
120000
140000
Iterations

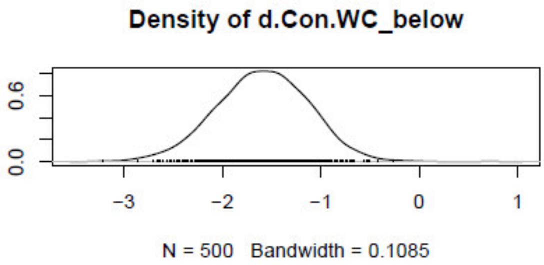
Density of d.Con.WC_below
0.6
0.0
−3
−2
−1
0
1
N = 500 Bandwidth = 0.1085

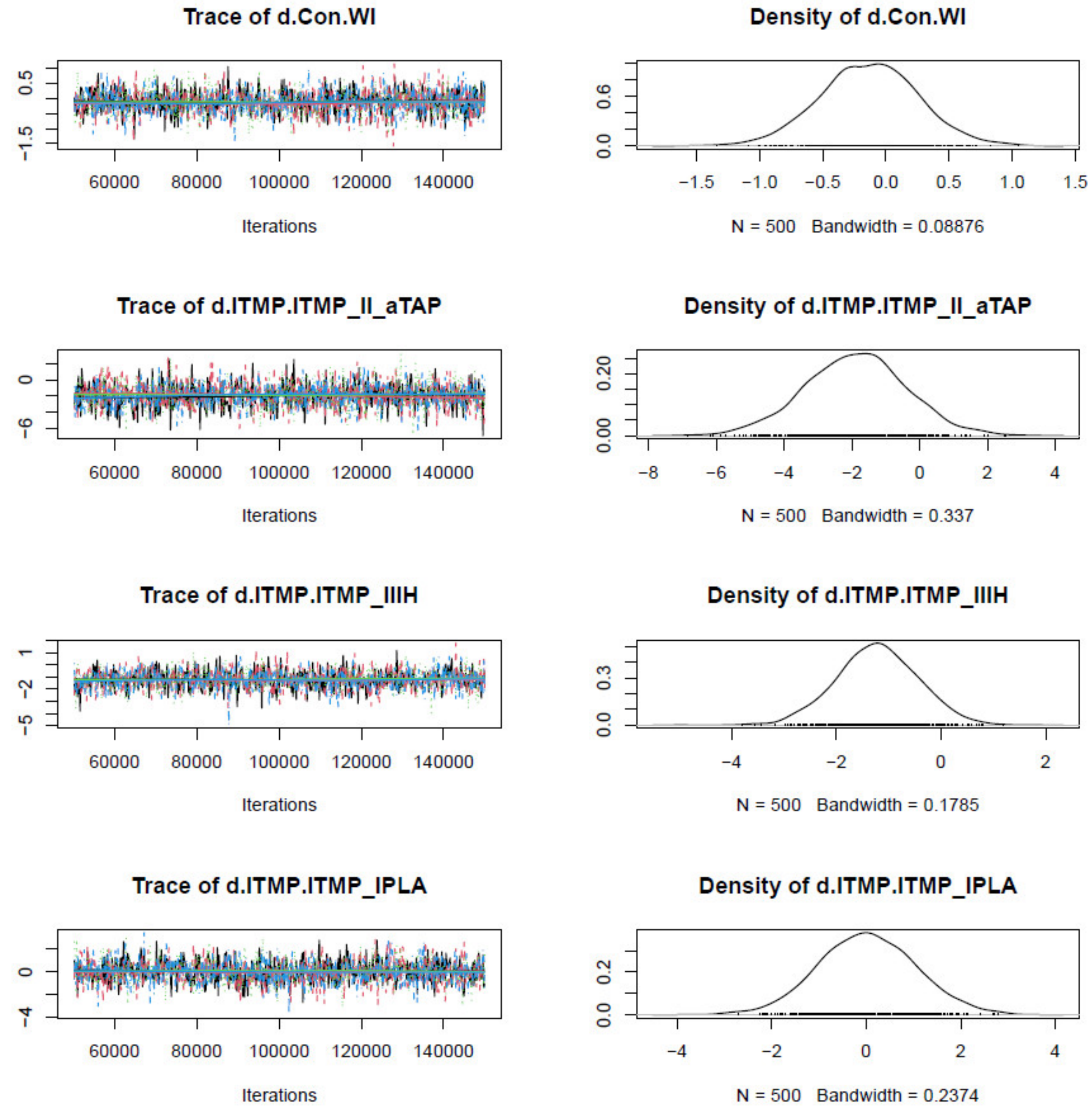

Figure S7. *Cont.*

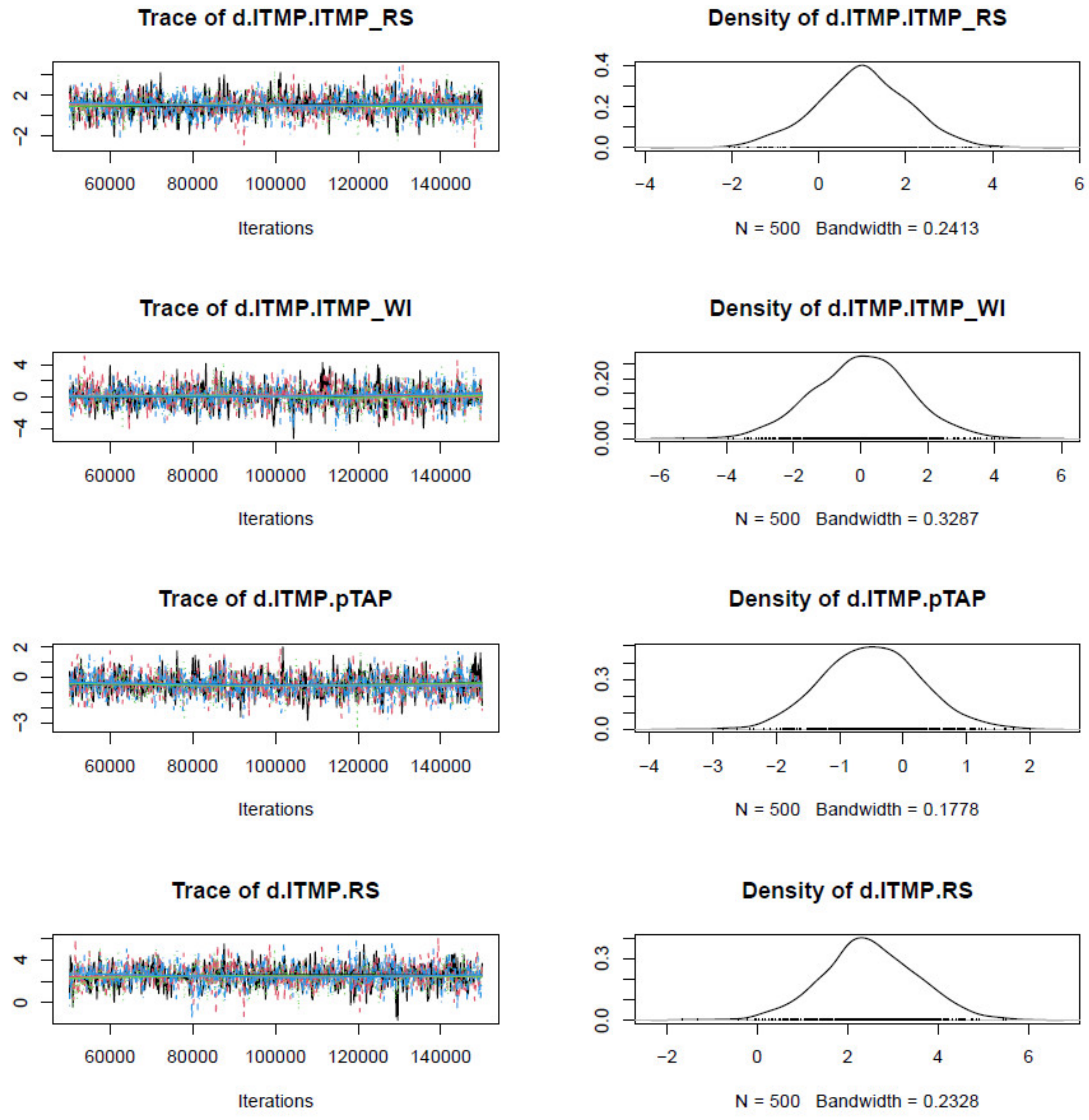

Figure S7. *Cont.*

(**C**) Gelman-Rubin-Brooks plot in fixed effect model.

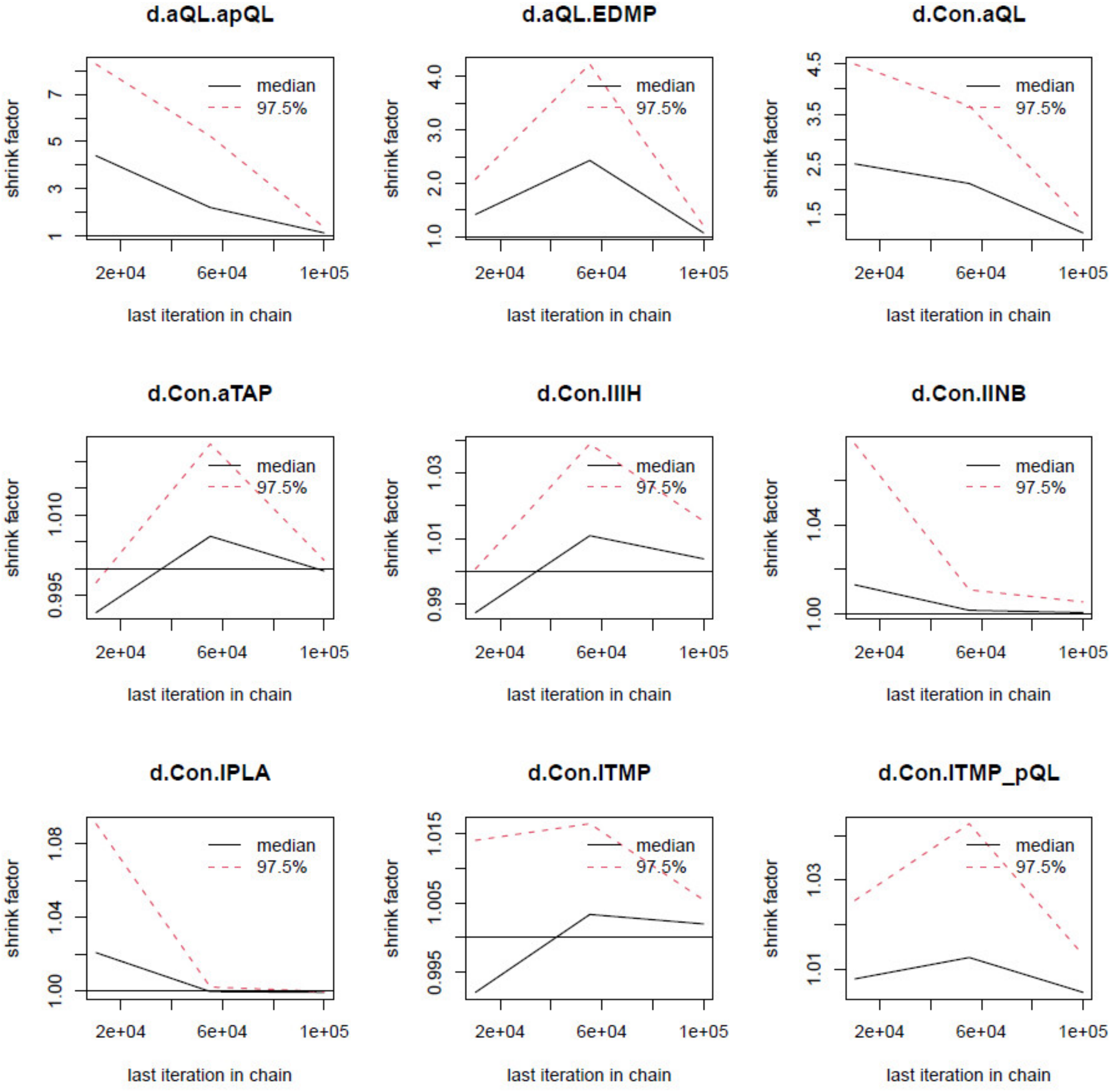

Figure S7. *Cont.*

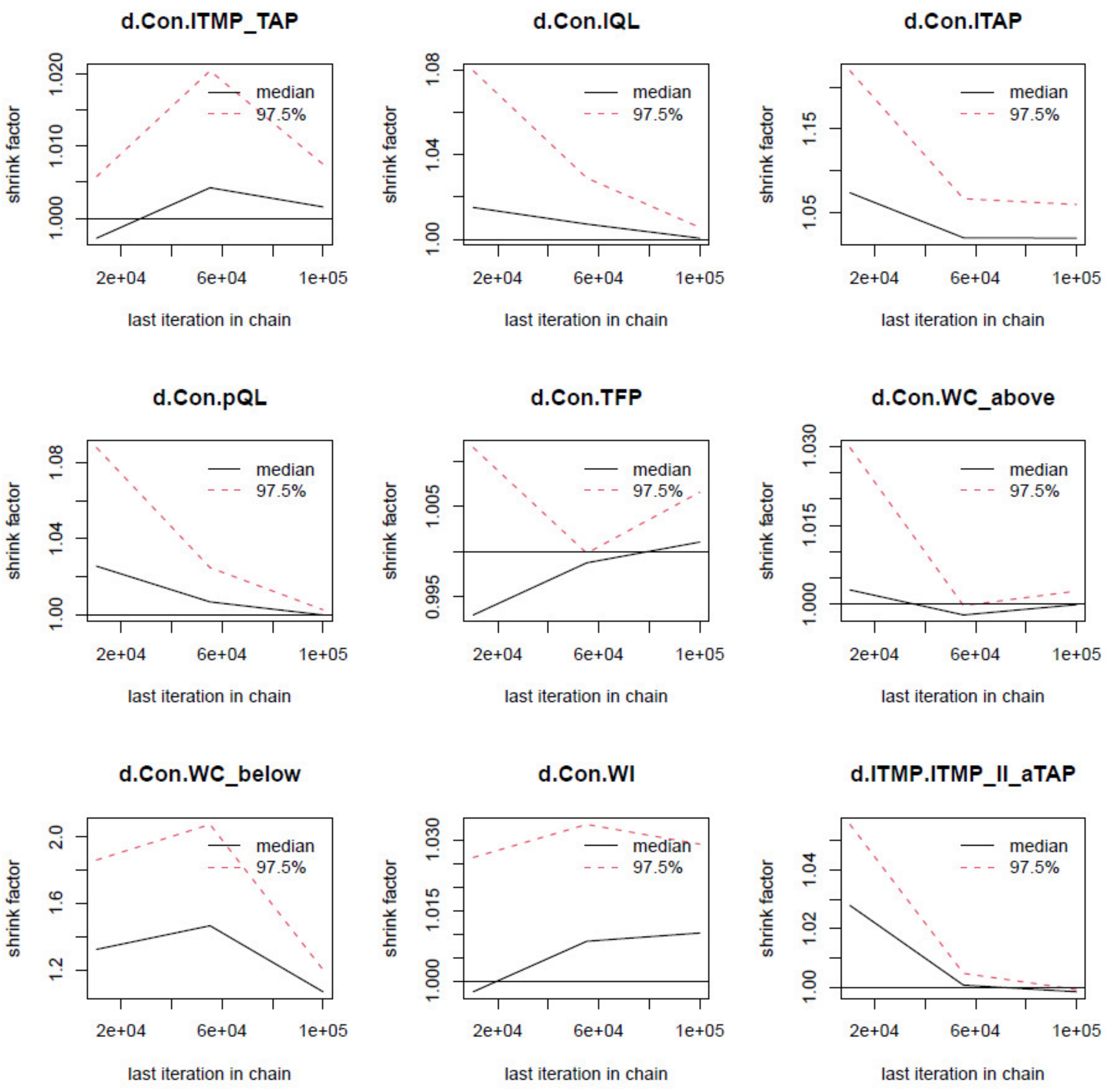
d.Con.ITMP_TAP
d.Con.IQL
d.Con.ITAP
d.Con.pQL
d.Con.TFP
d.Con.WC_above
d.Con.WC_below
d.Con.WI
d.ITMP.ITMP_II_aTAP
shrink factor
last iteration in chain
median
97.5%
2e+04
6e+04
1e+05

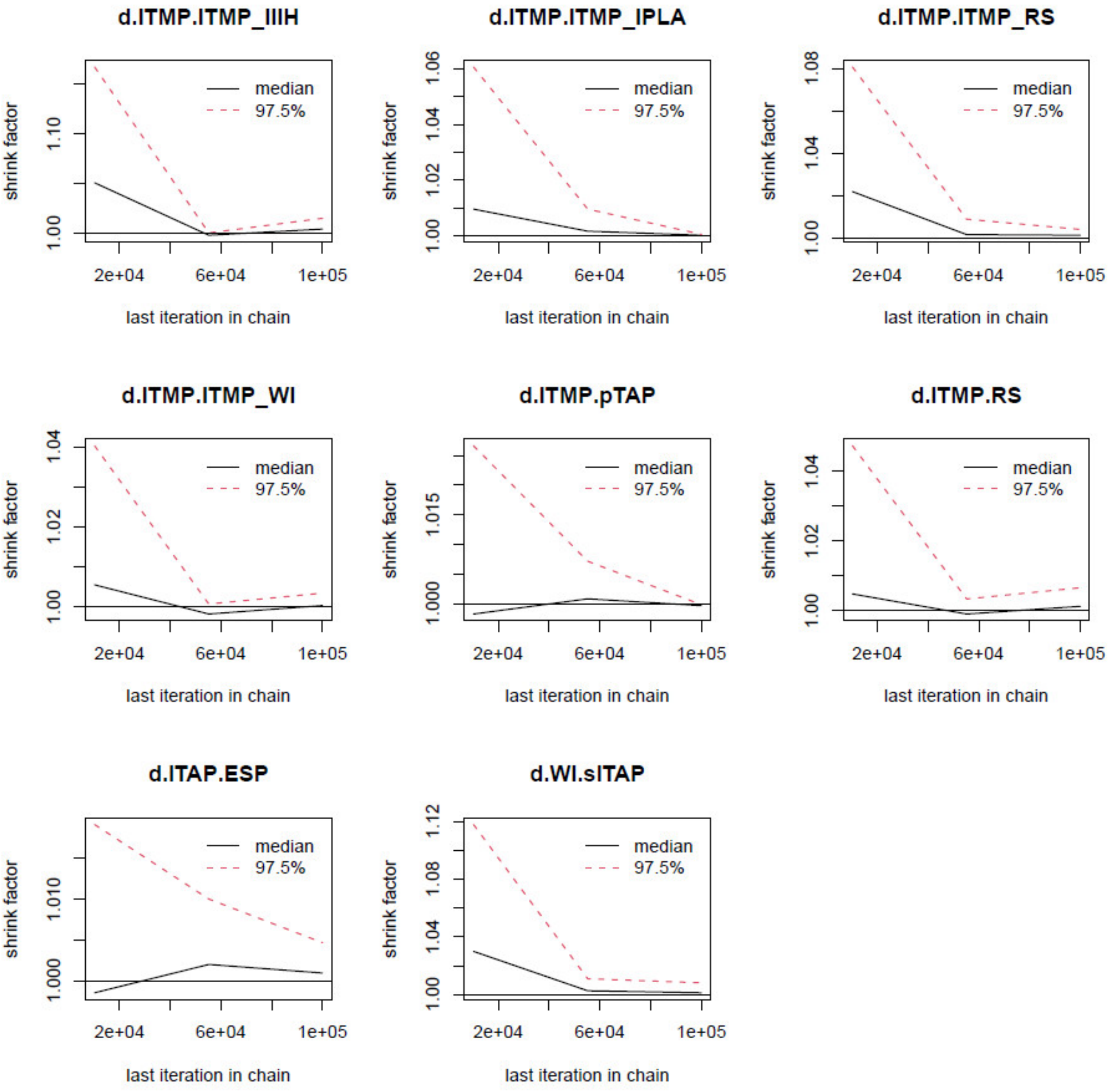

Figure S7. *Cont.*

(**D**) Gelman-Rubin-Brooks plot in random effect model.

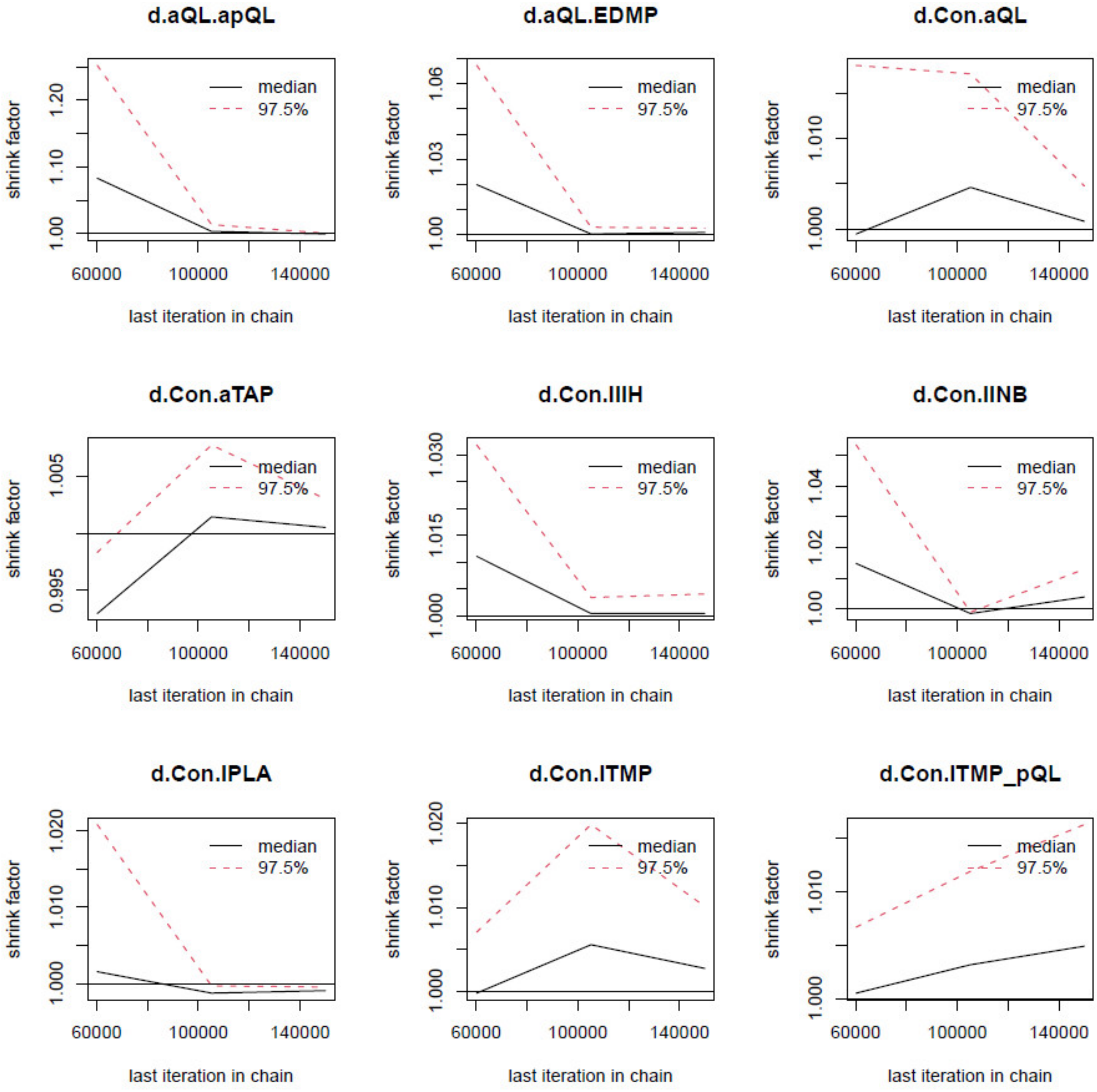

Figure S7. *Cont.*

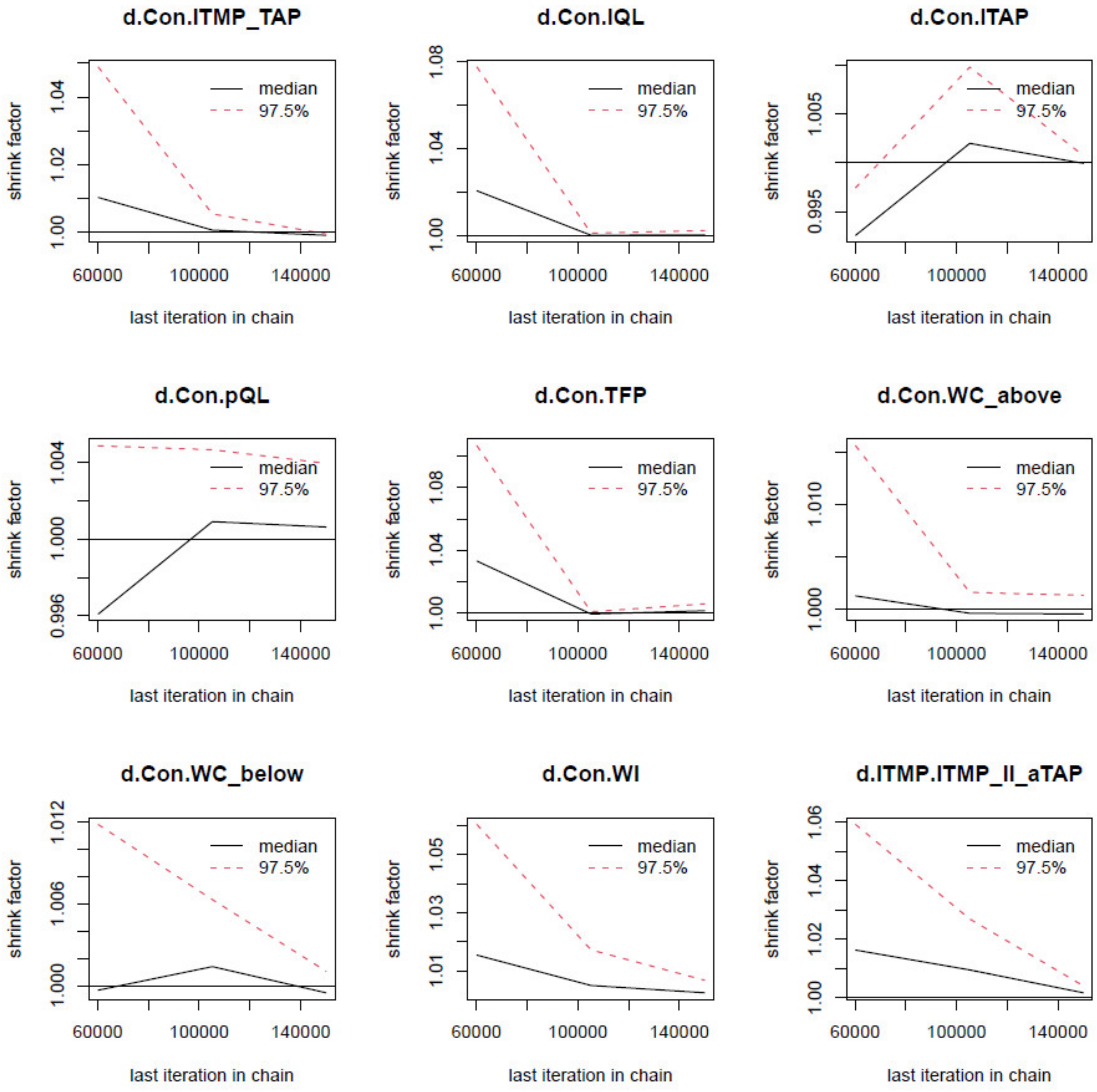

Figure S7. *Cont.*

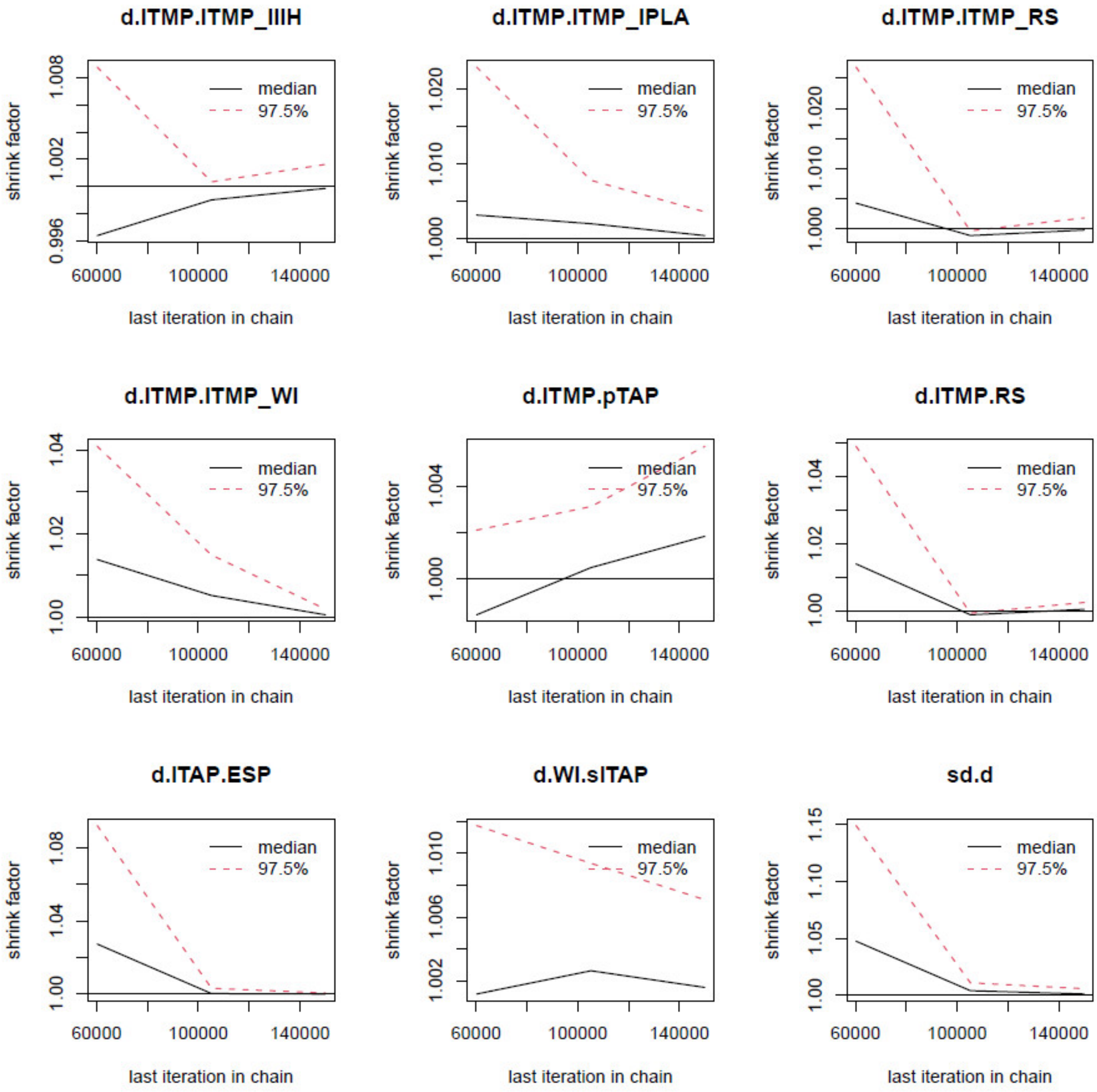

Figure S7. *Cont.*

(**E**) Forest plot comparing with control in random effect model.

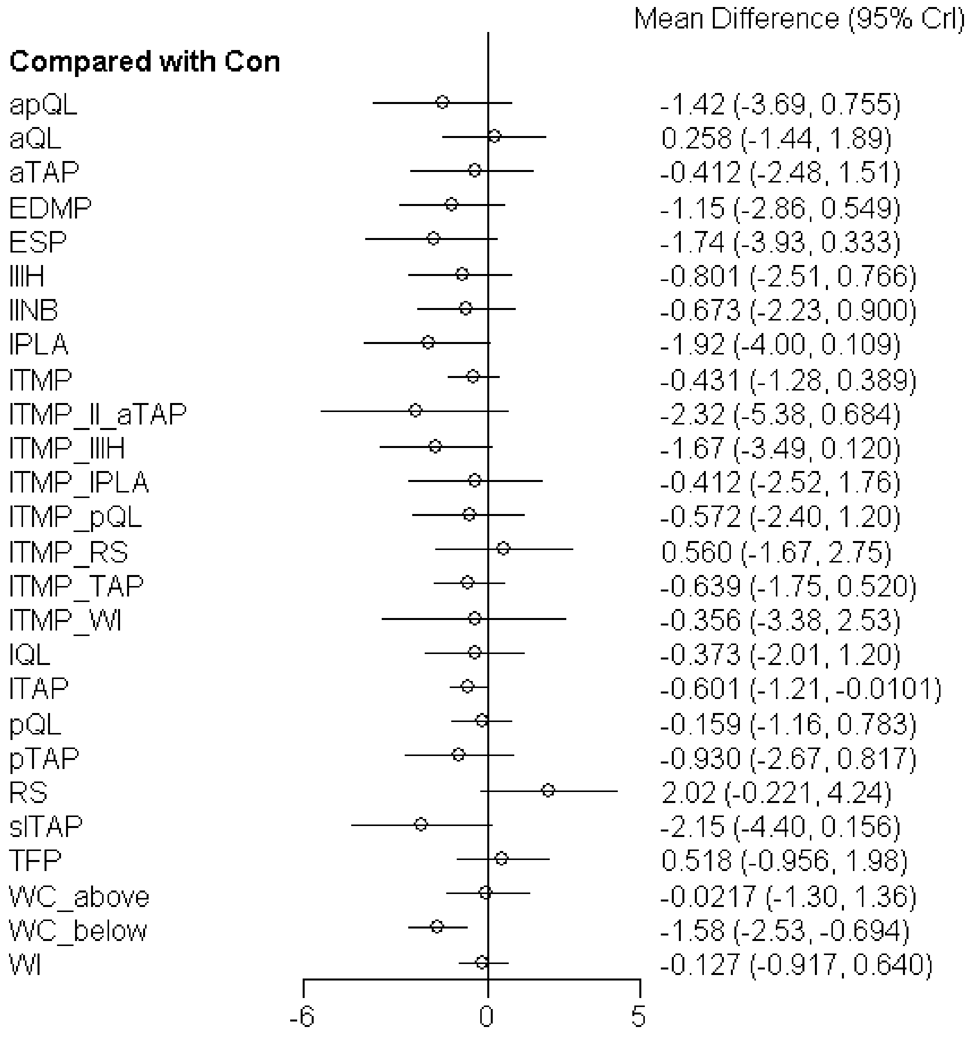

Figure S7. *Cont.*

(**F**) Node splitting plot in random effect model.

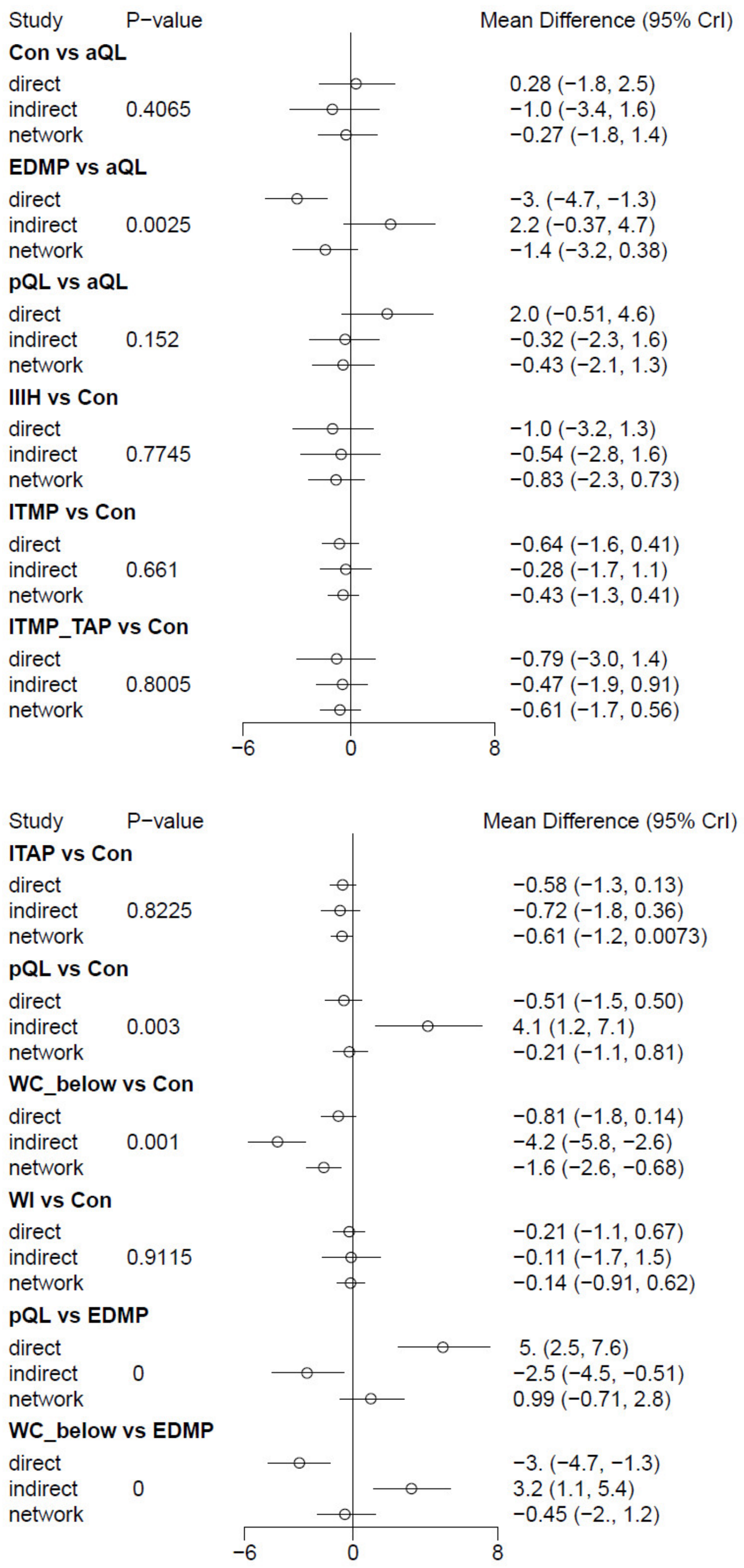

Figure S7. *Cont.*

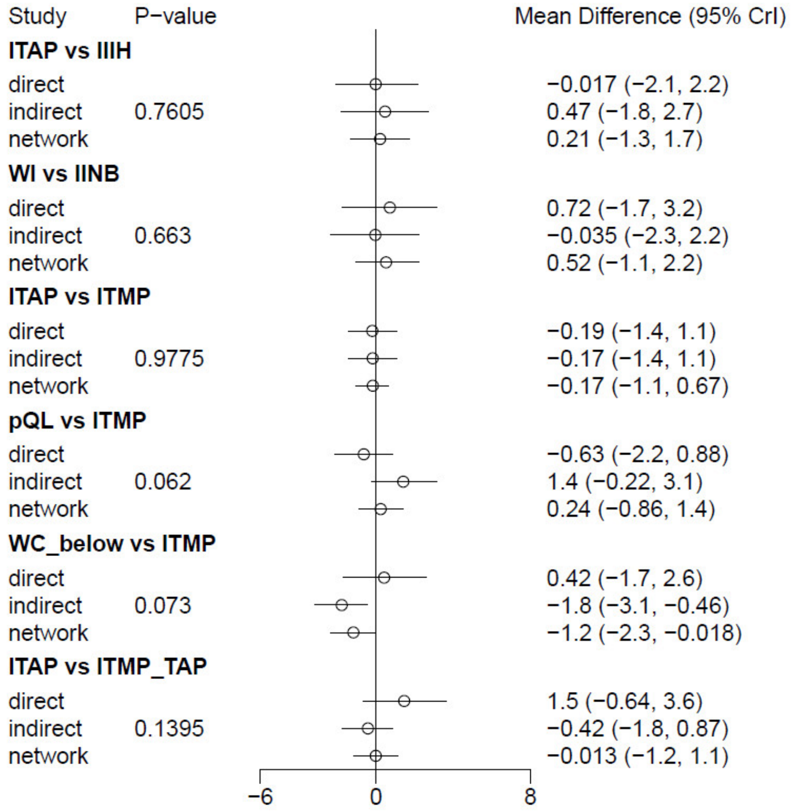

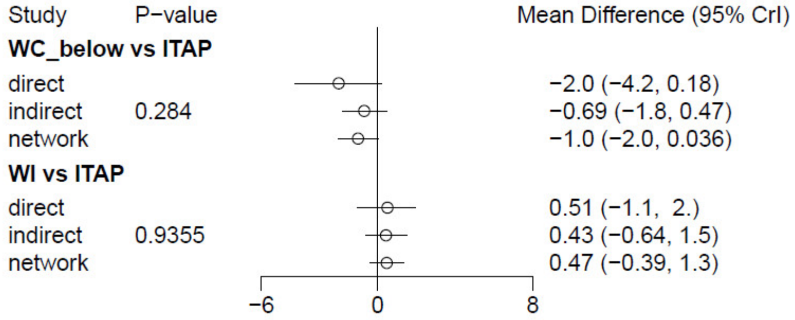

Figure S7. *Cont.*

(**G**) Rankogram in random effect model.

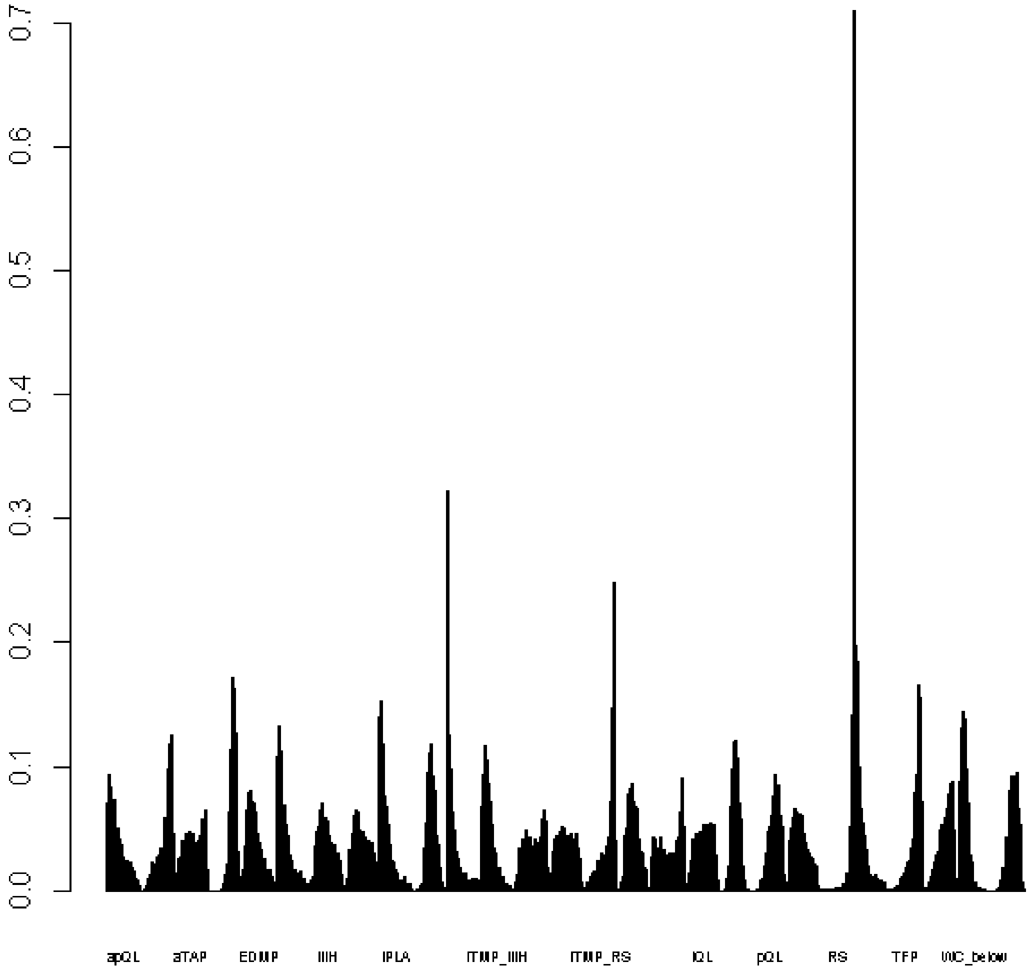

Figure S7. Pain at rest 24 h after surgery. (**A**) Trace plot and density plot in fixed effect model; (**B**) Trace plot and density plot in random effect model; (**C**) Gelman-Rubin-Brooks plot in fixed effect model, (**D**) Gelman-Rubin-Brooks plot in random effect model; (**E**) Forest plot comparing with control in random effect model; (**F**) Node splitting plot in random effect model; (**G**) Rankogram in random effect model.

(**A**) Trace plot and density plot in fixed effect model.

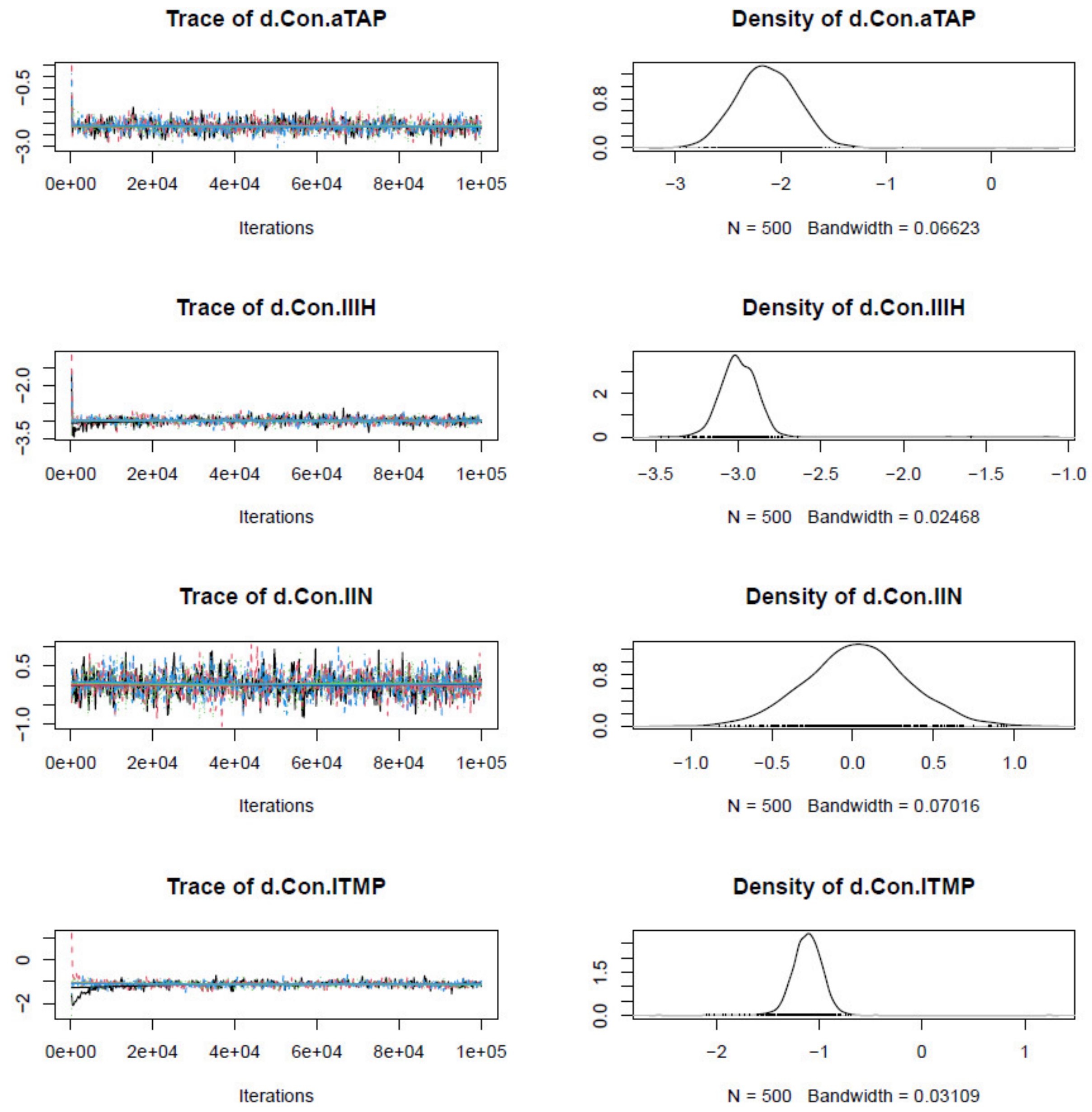

Figure S8. *Cont.*

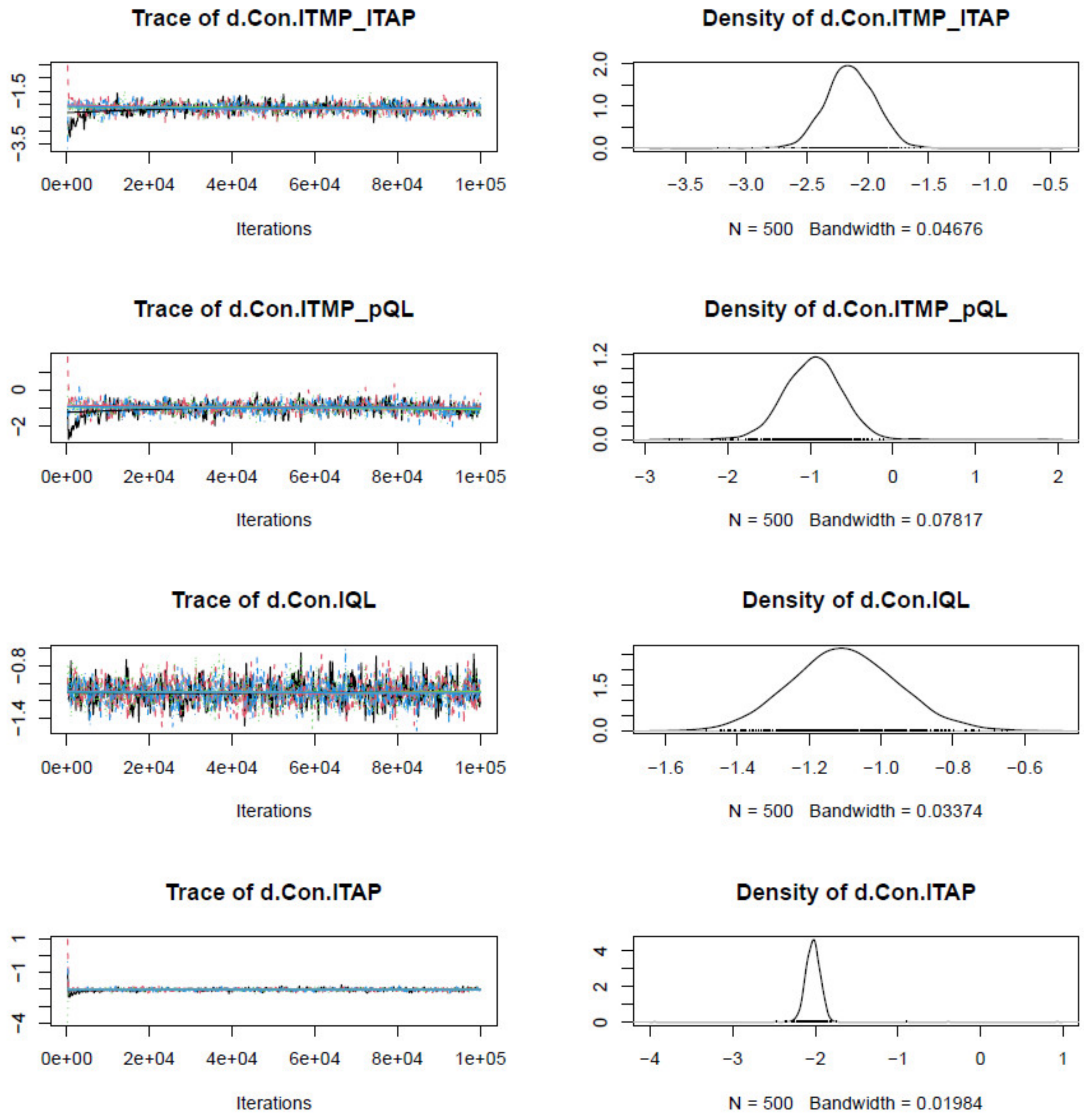

Figure S8. *Cont.*

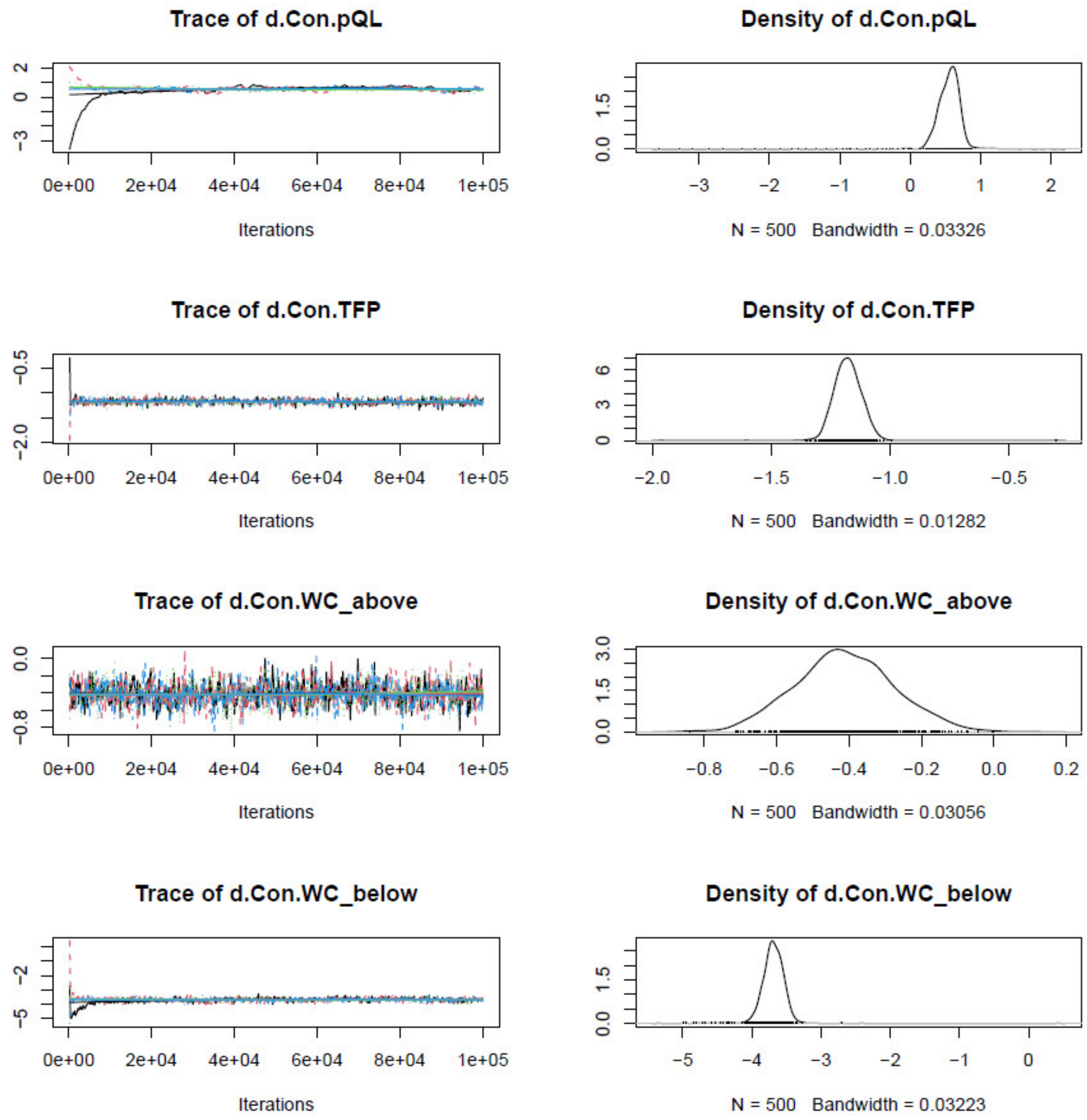

Figure S8. *Cont.*

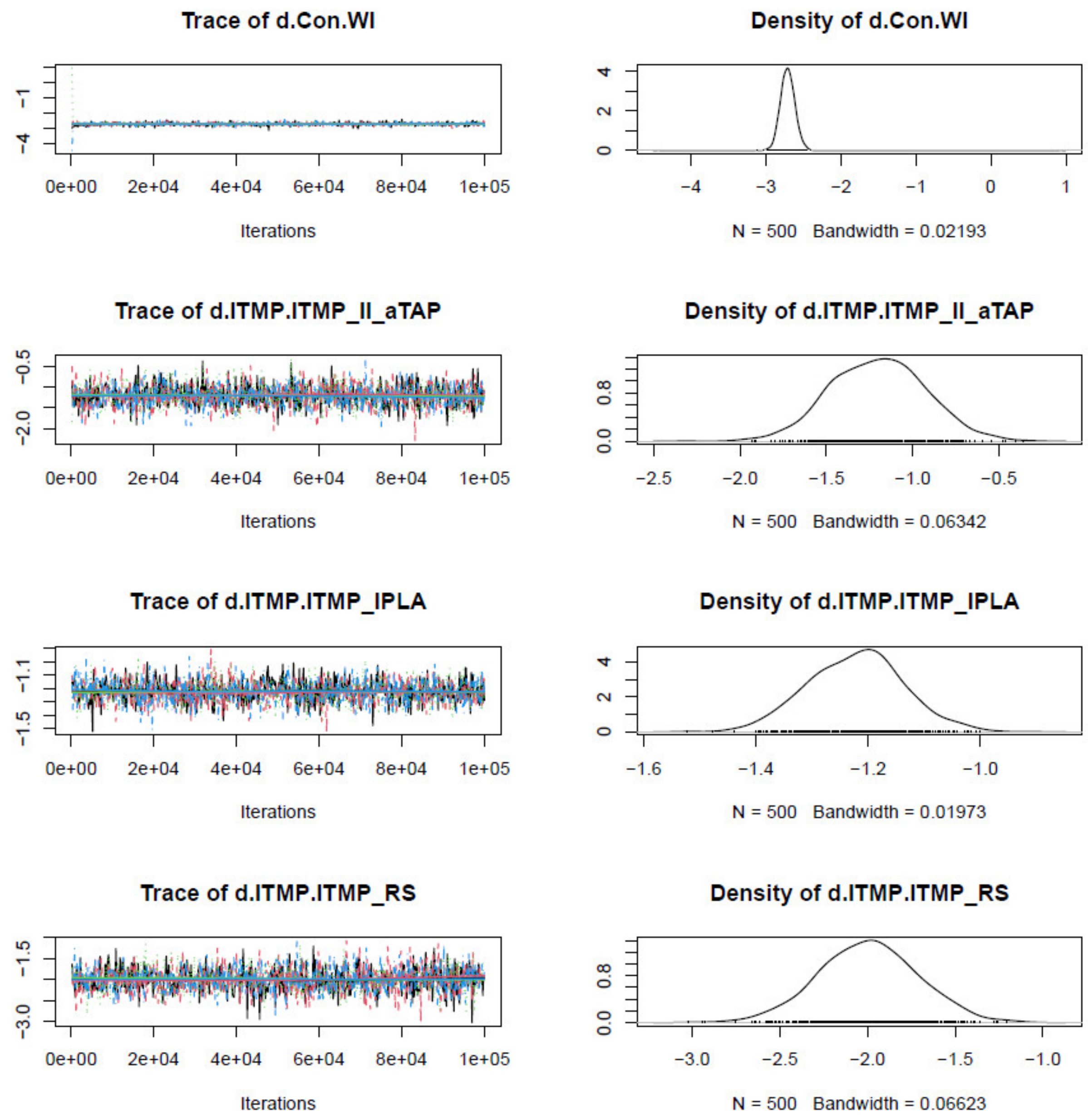

Figure S8. *Cont.*

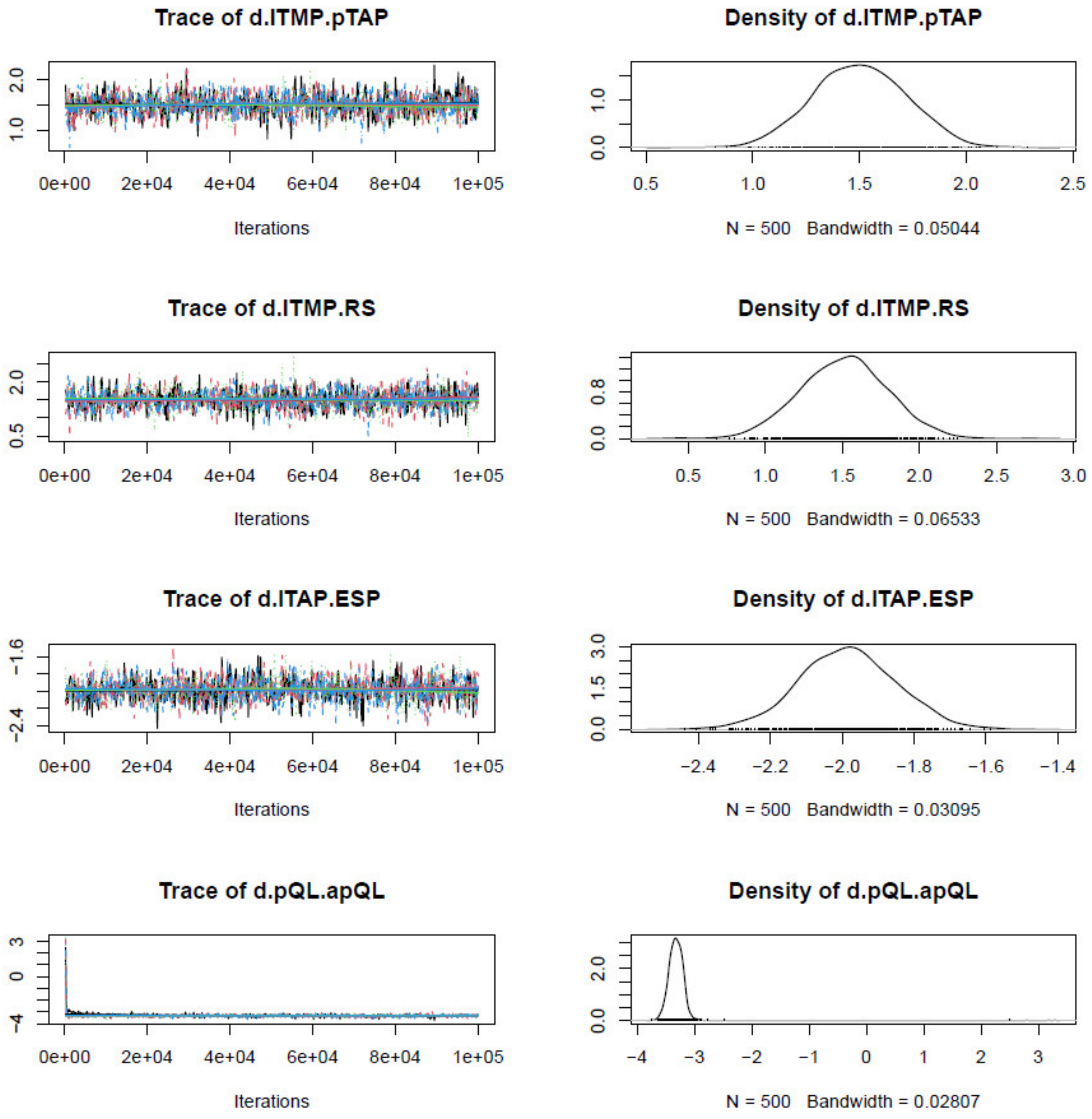

Figure S8. *Cont.*

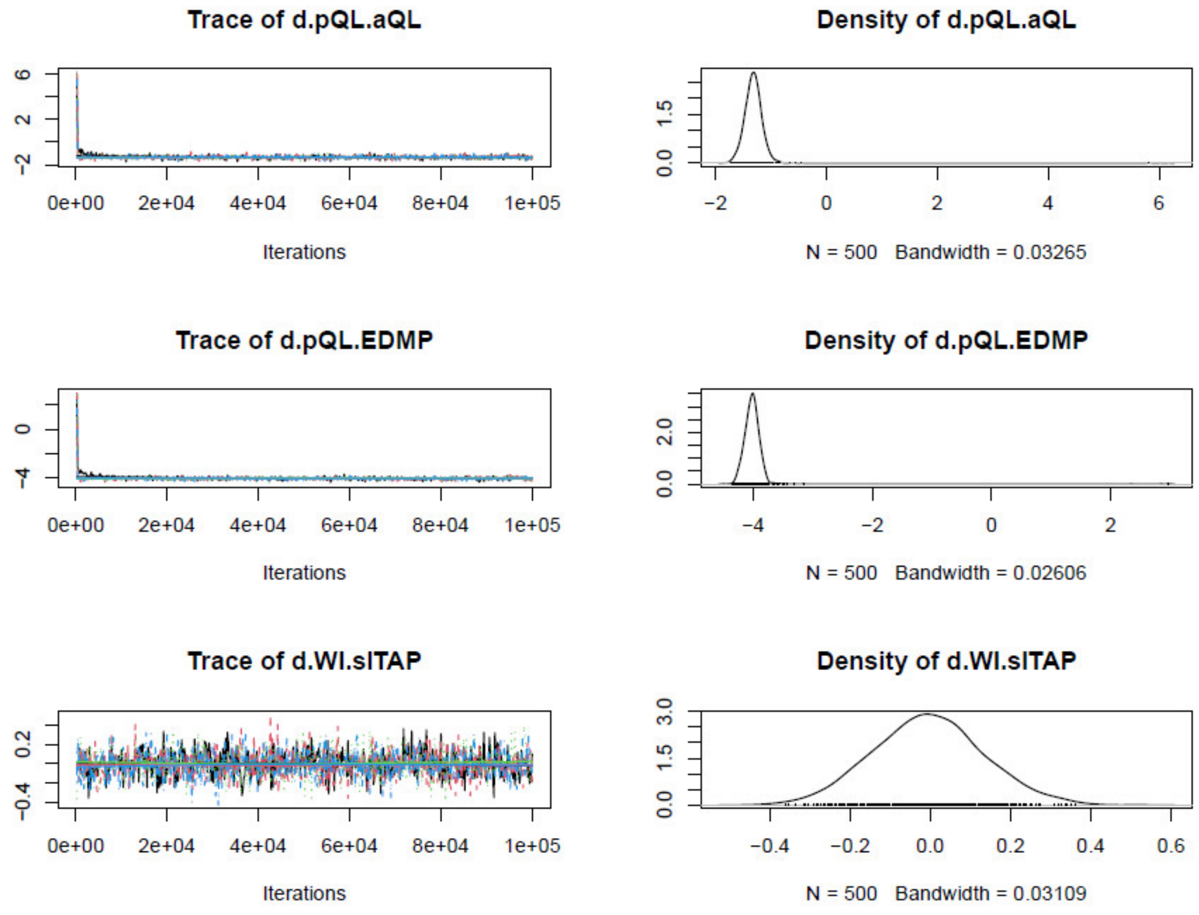

Figure S8. *Cont.*

(**B**) Trace plot and density plot in random effect model.

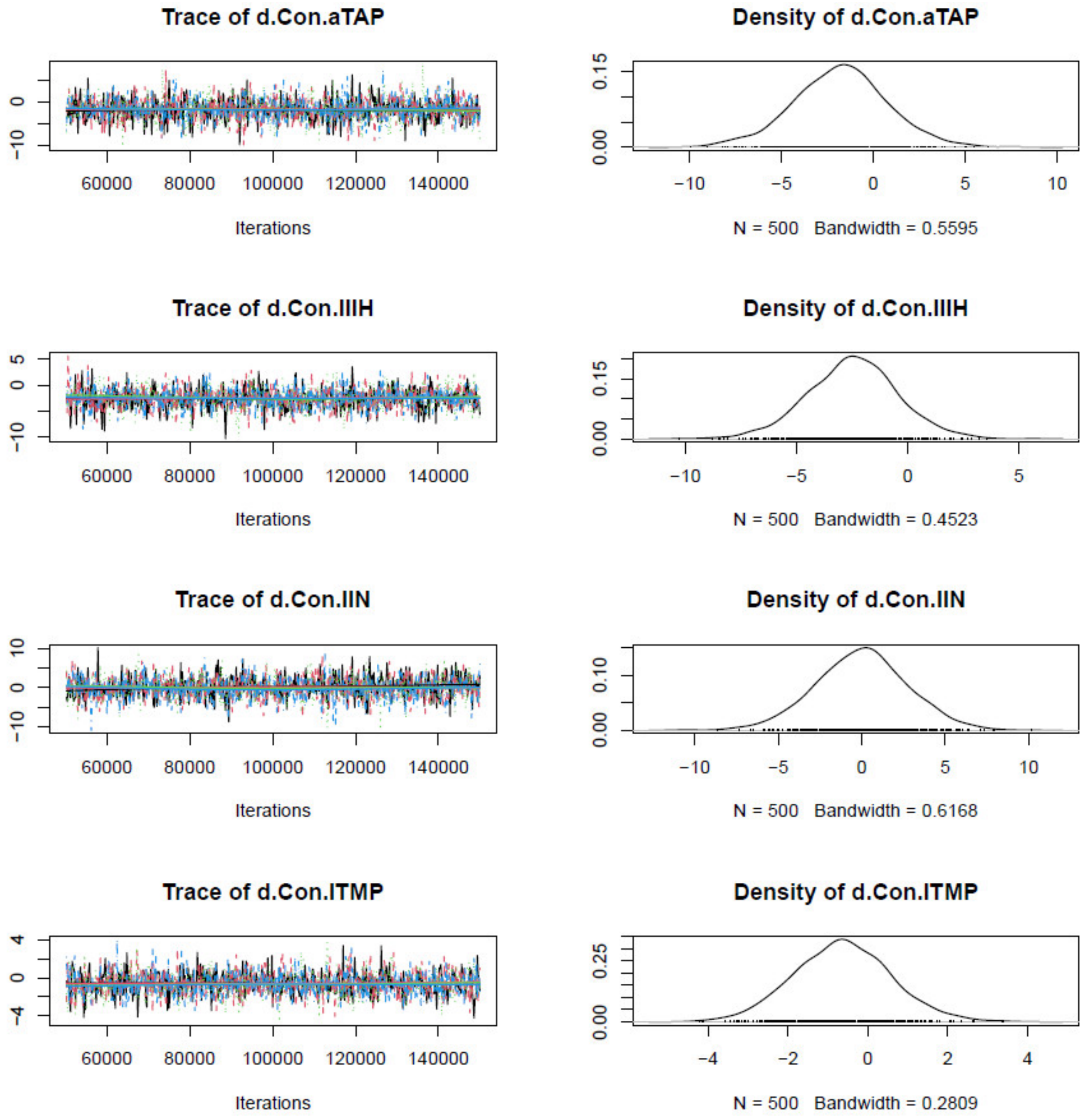

Figure S8. *Cont.*

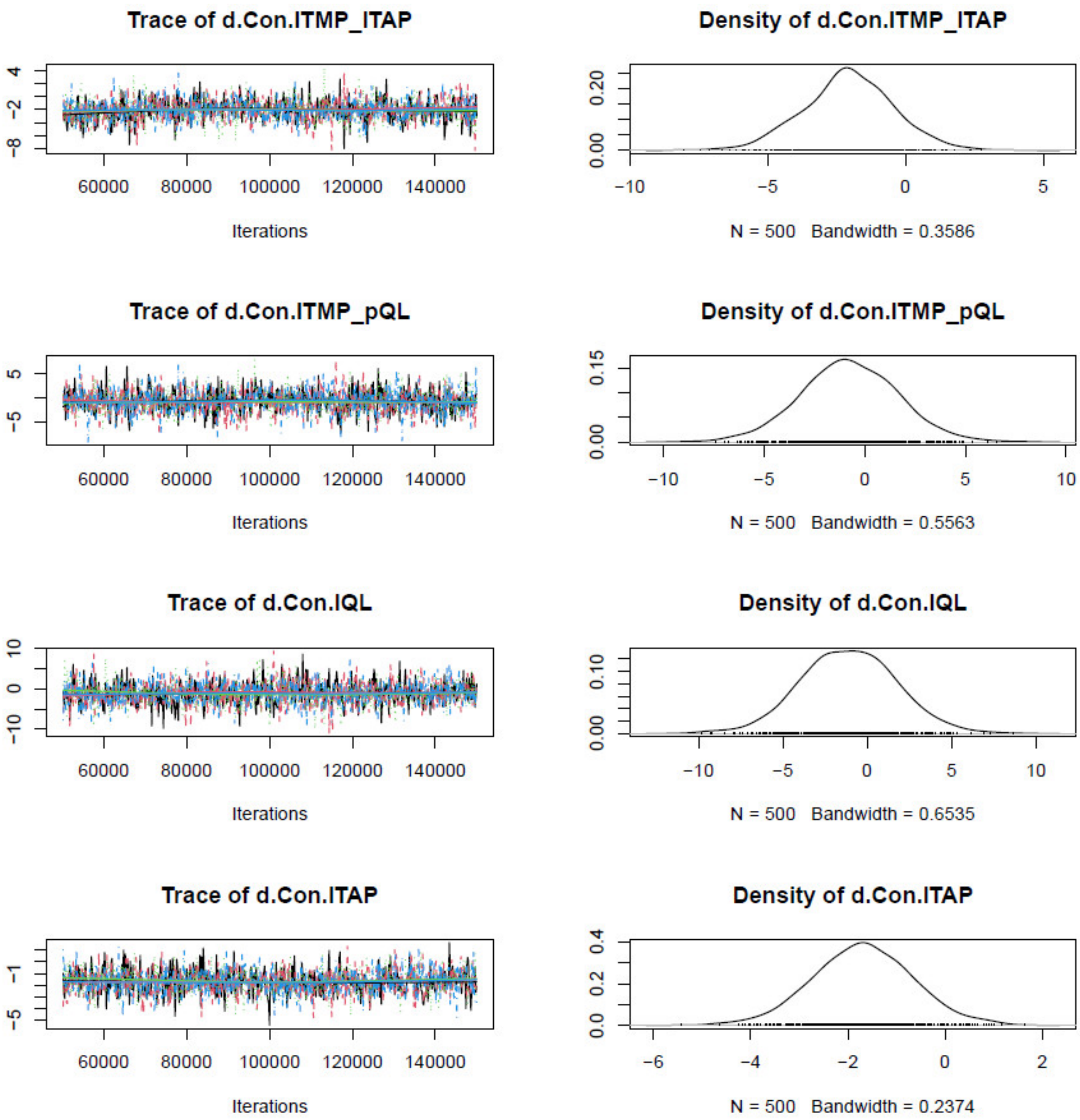

Figure S8. *Cont.*

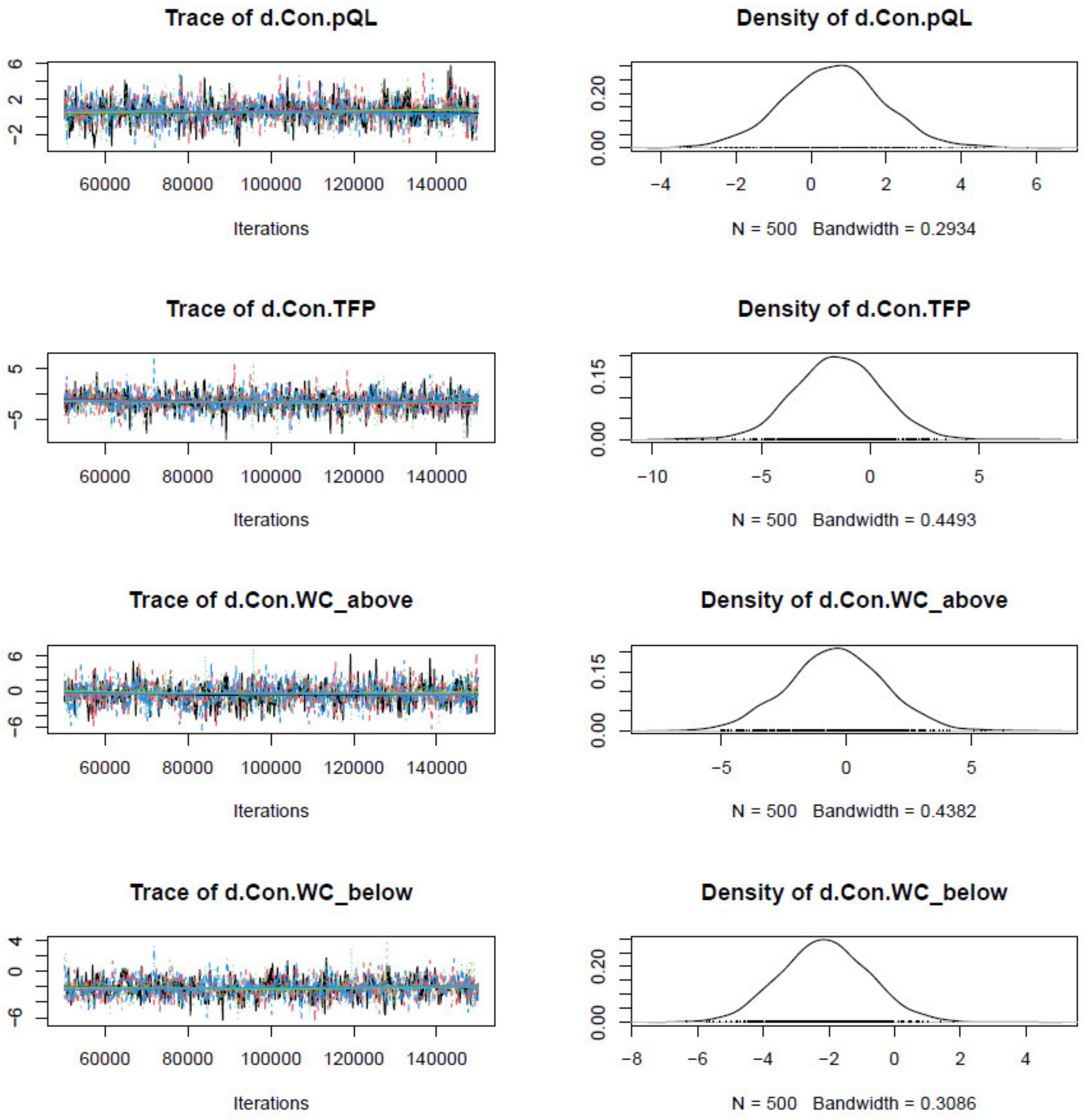

Figure S8. *Cont.*

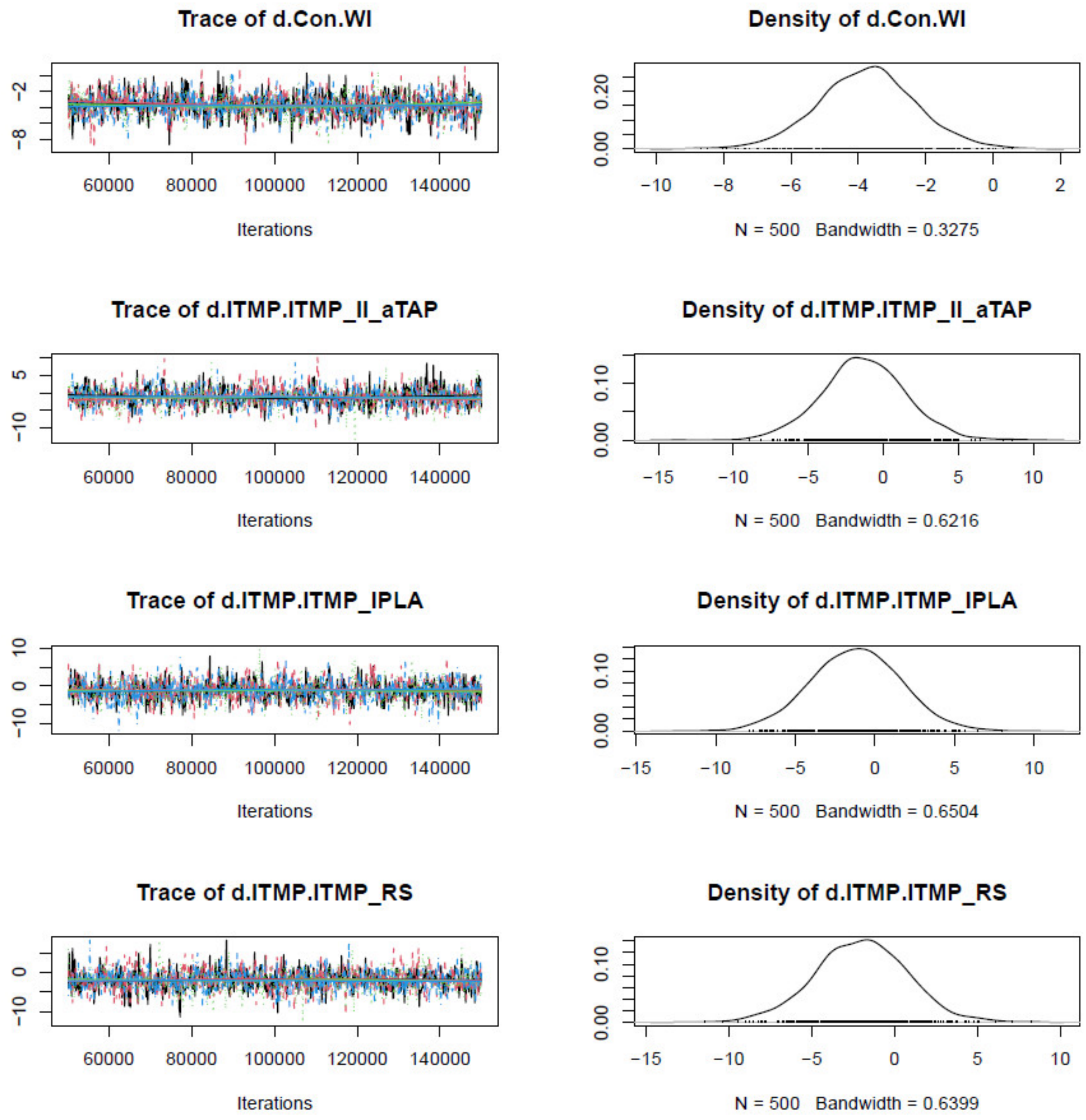

Figure S8. *Cont.*

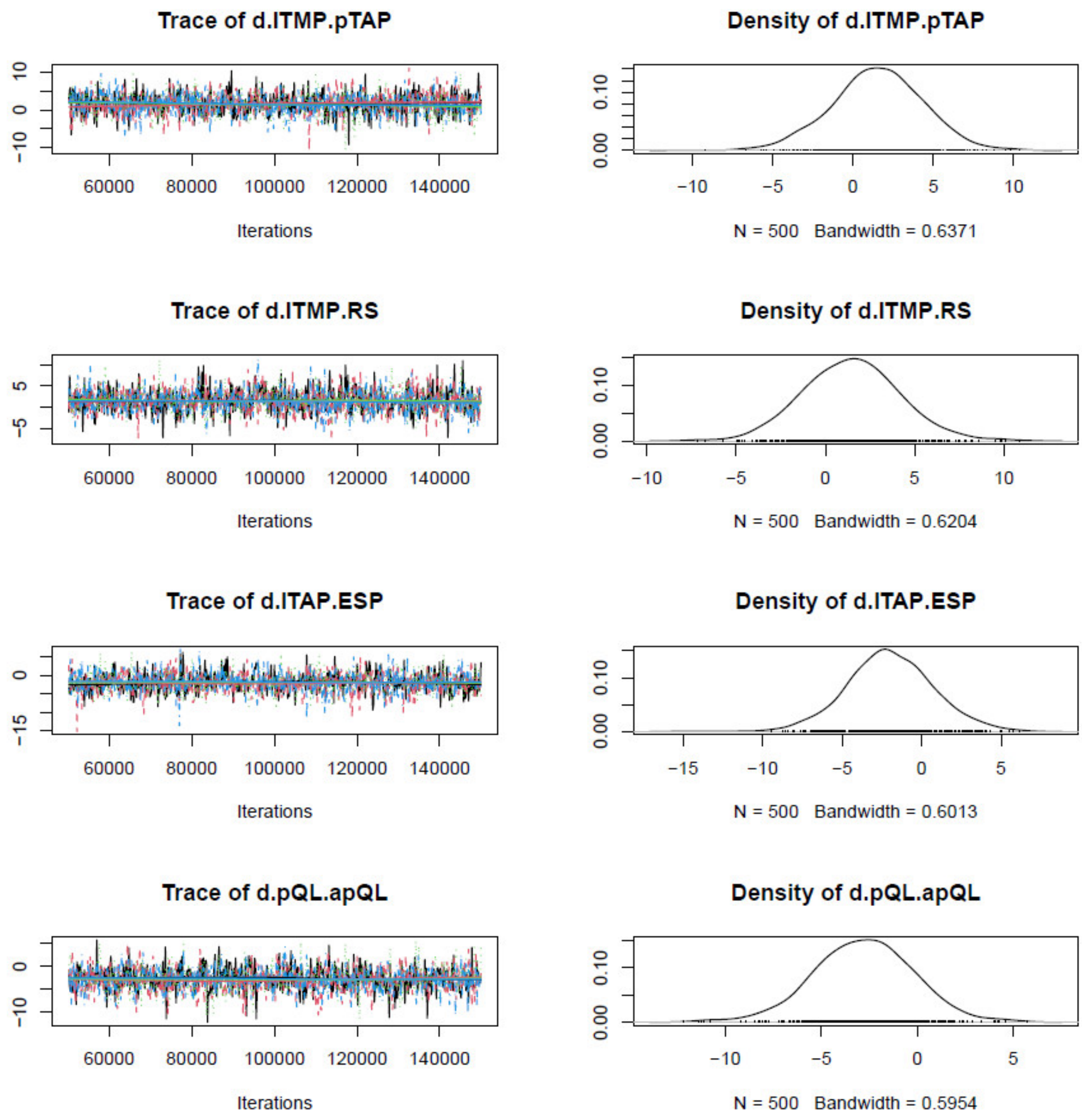

Figure S8. *Cont.*

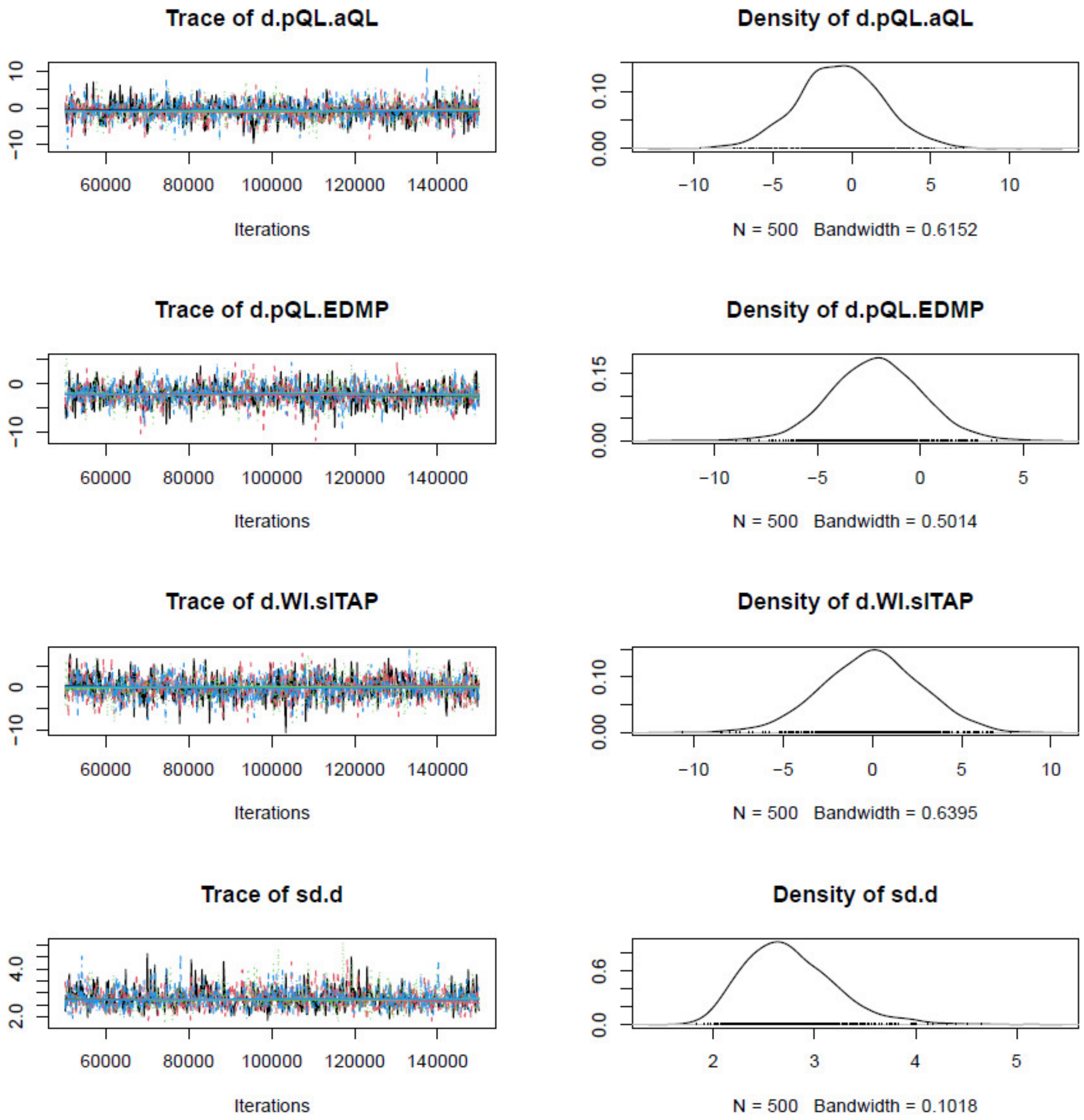

Figure S8. *Cont.*

(**C**) Gelman-Rubin-Brooks plot in fixed effect model.

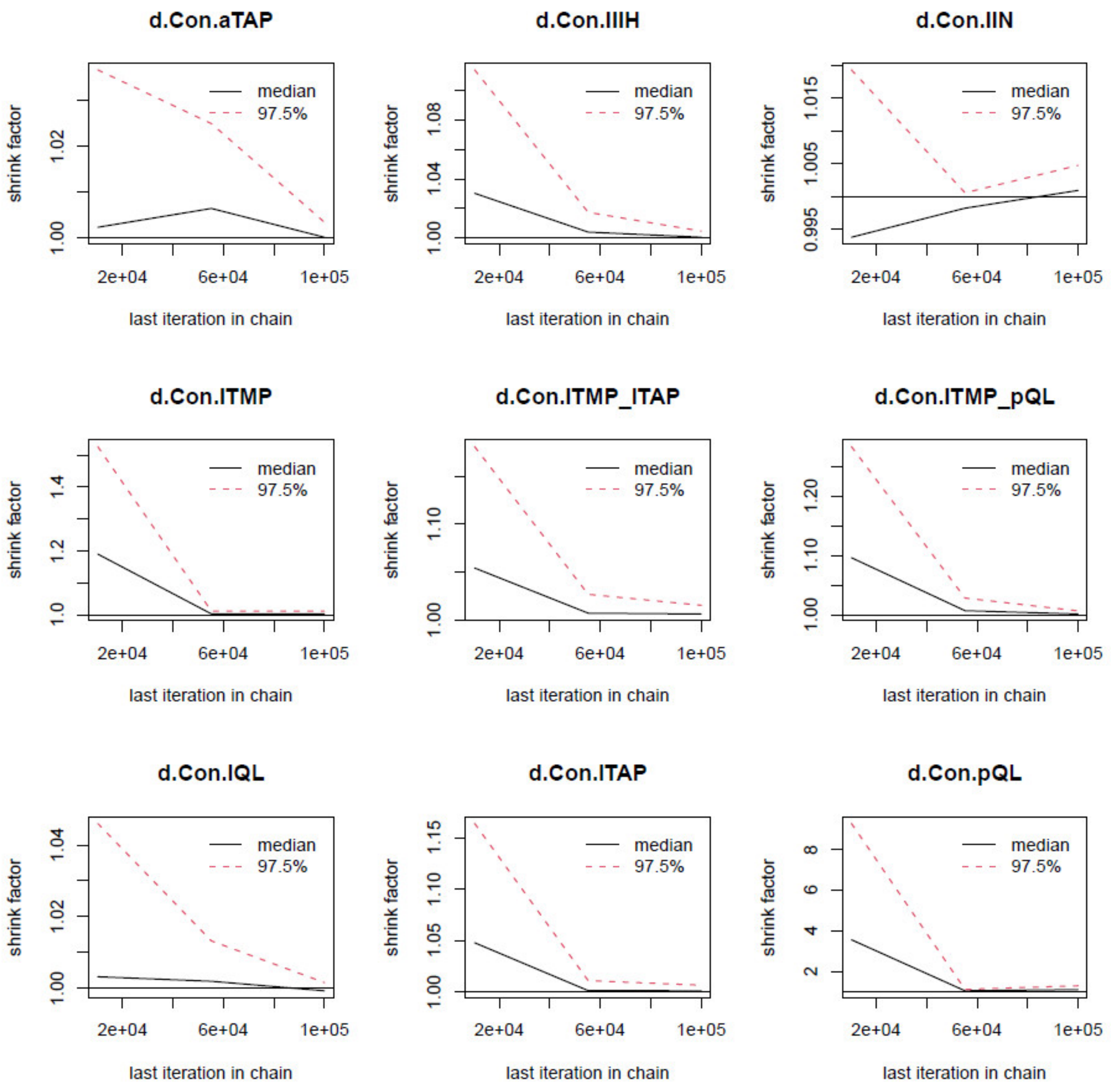

Figure S8. *Cont.*

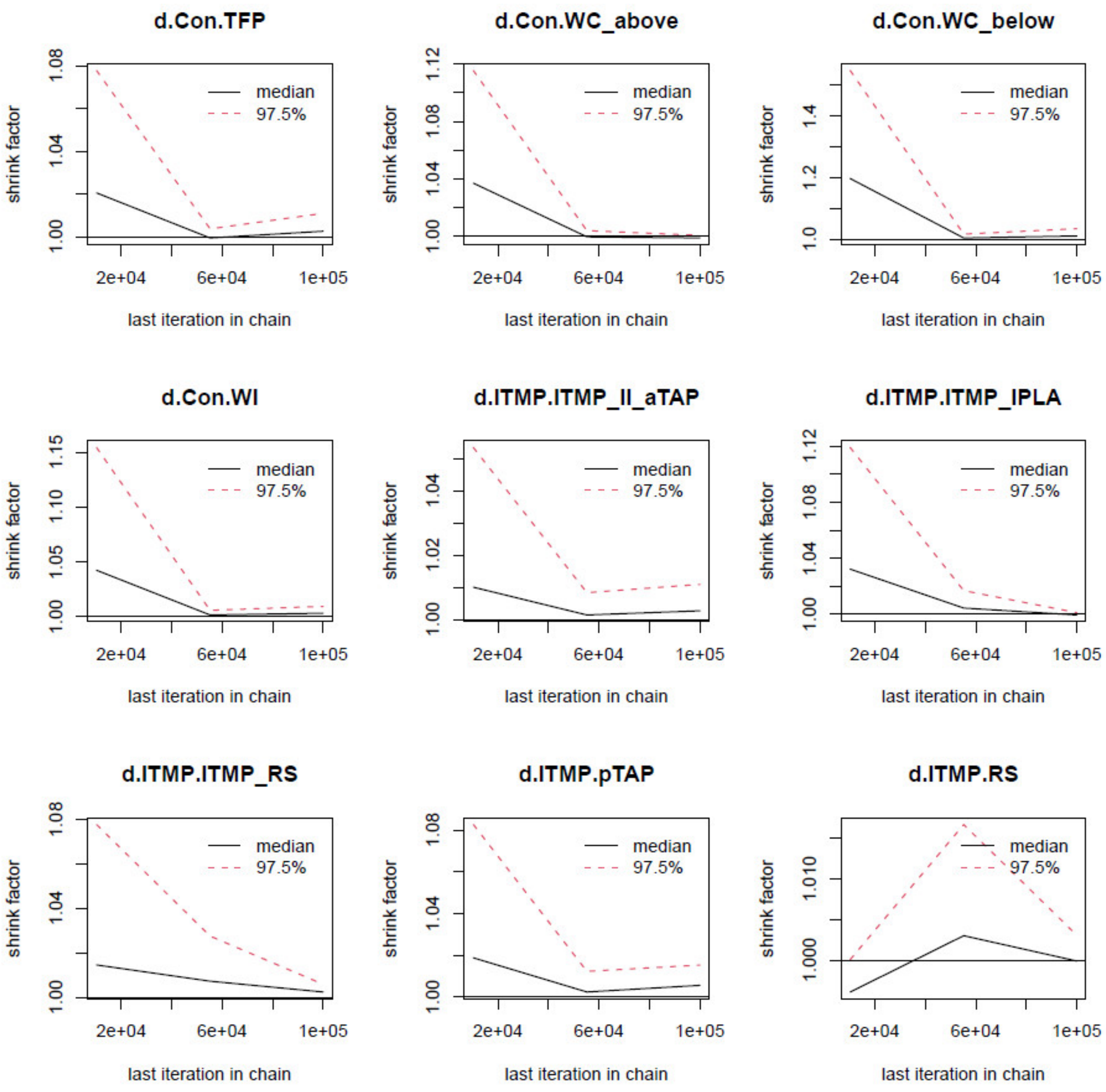

Figure S8. *Cont.*

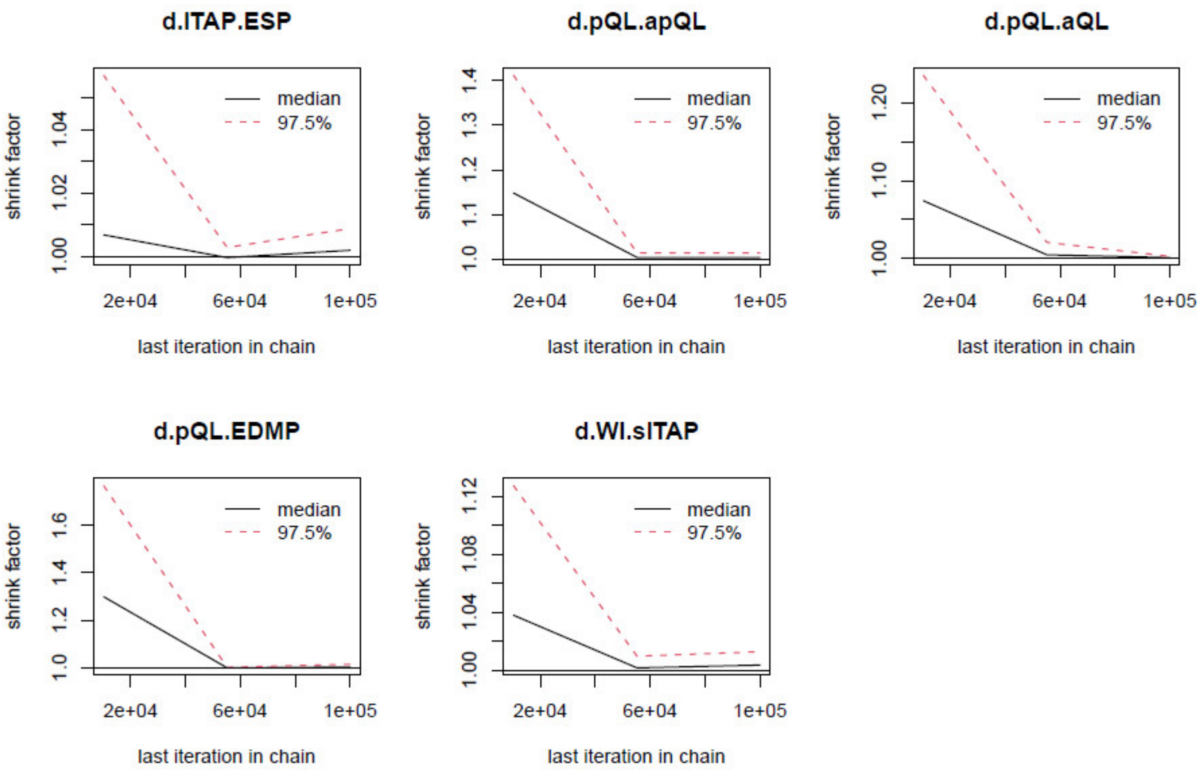

Figure S8. *Cont.*

(**D**) Gelman-Rubin-Brooks plot in random effect model.

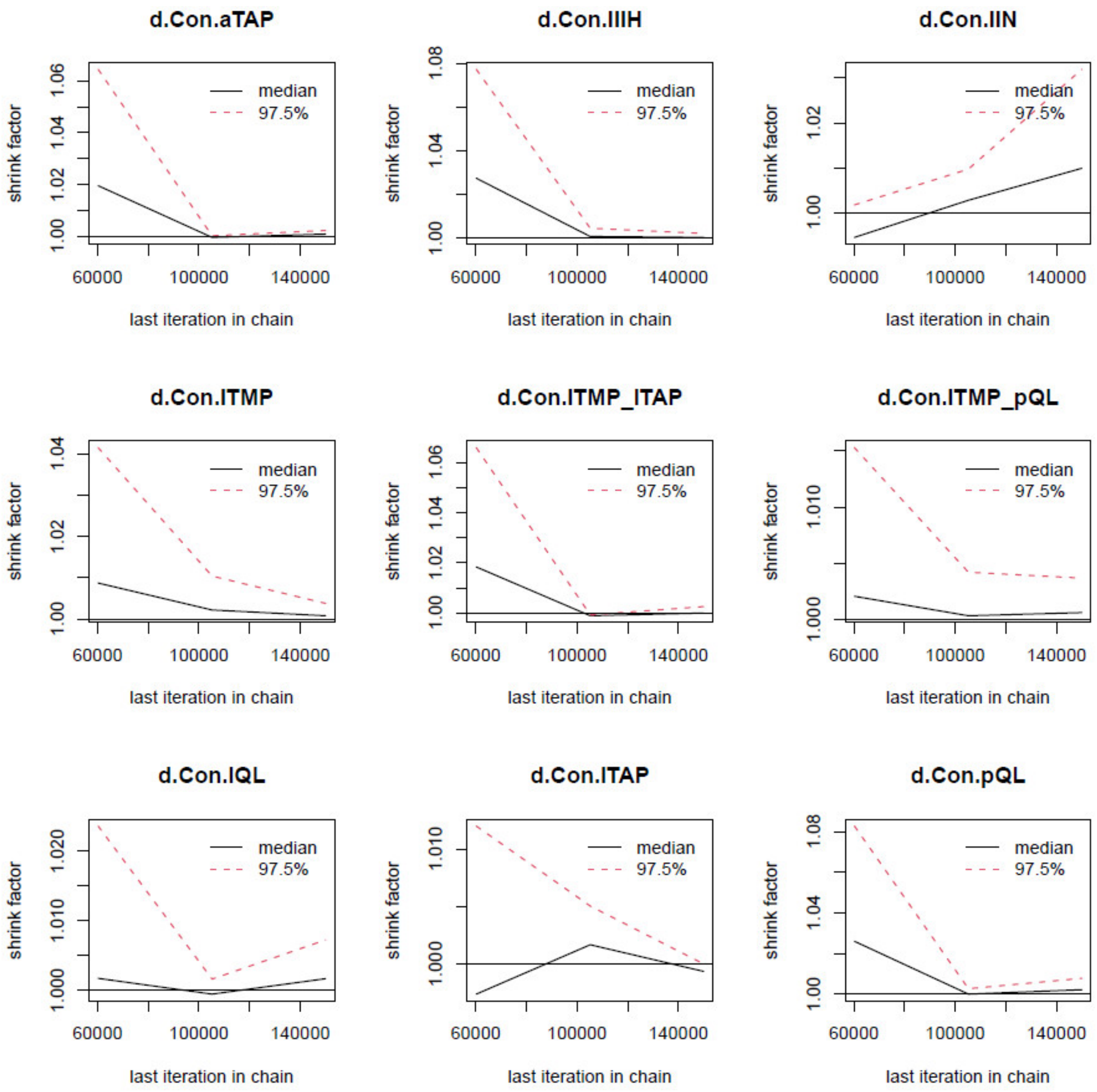

Figure S8. *Cont.*

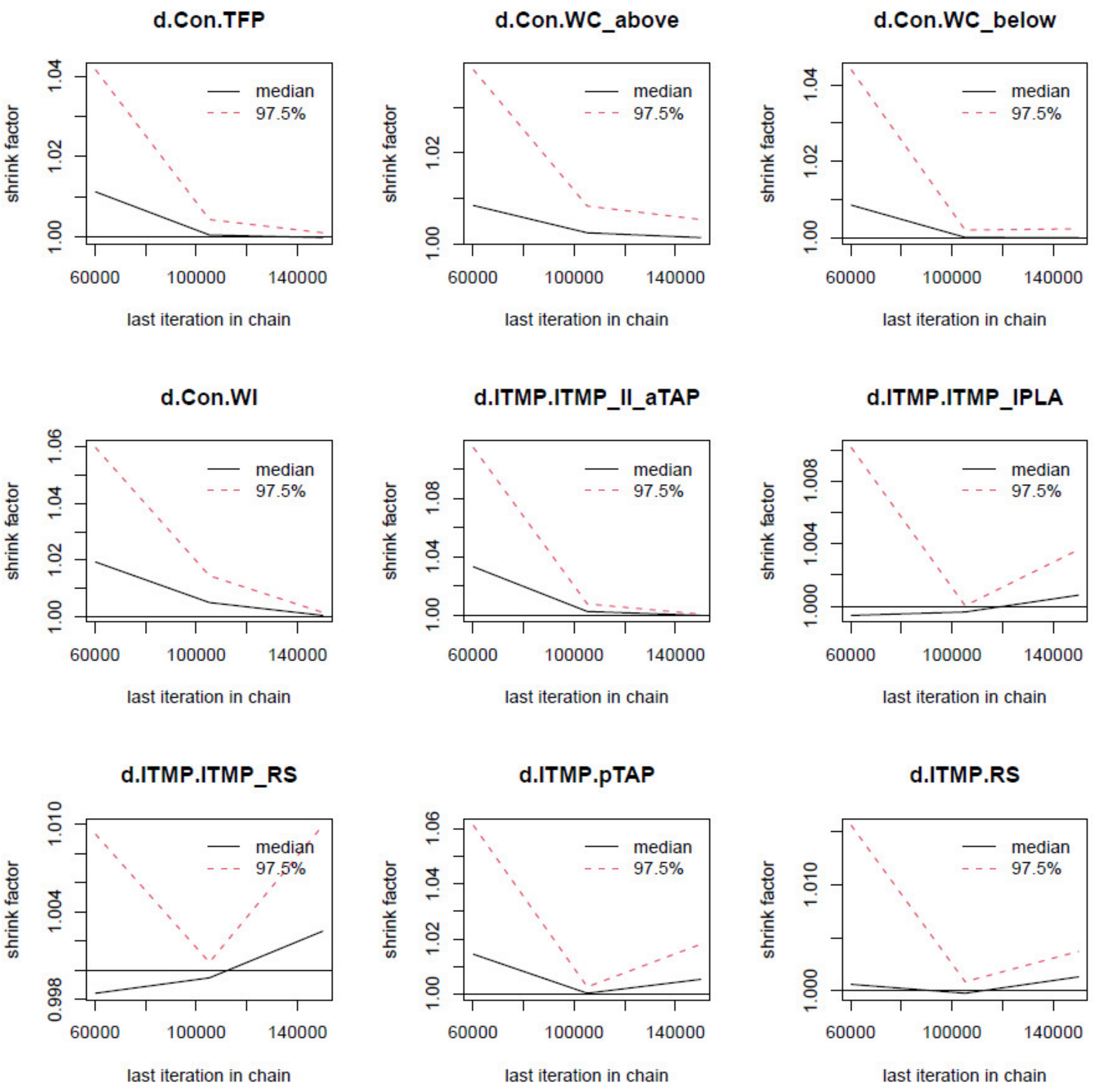

Figure S8. *Cont.*

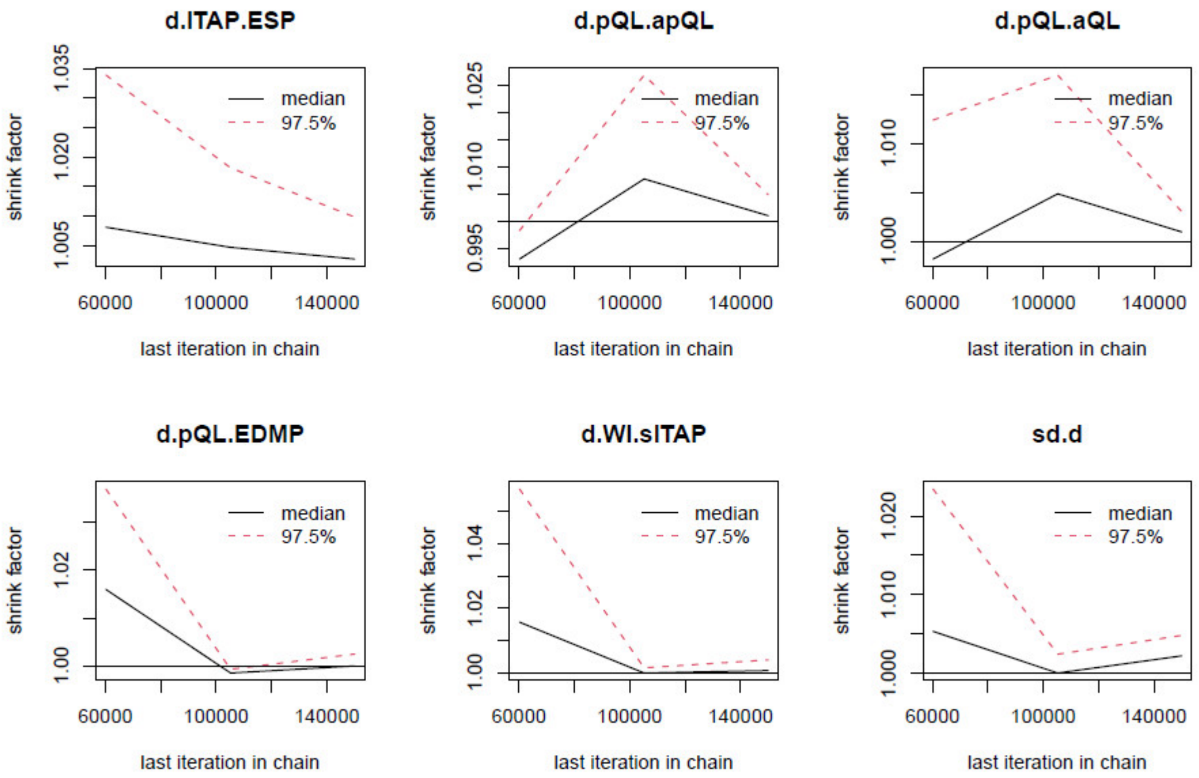

Figure S8. *Cont.*

(**E**) Forest plot comparing with control in random effect model.

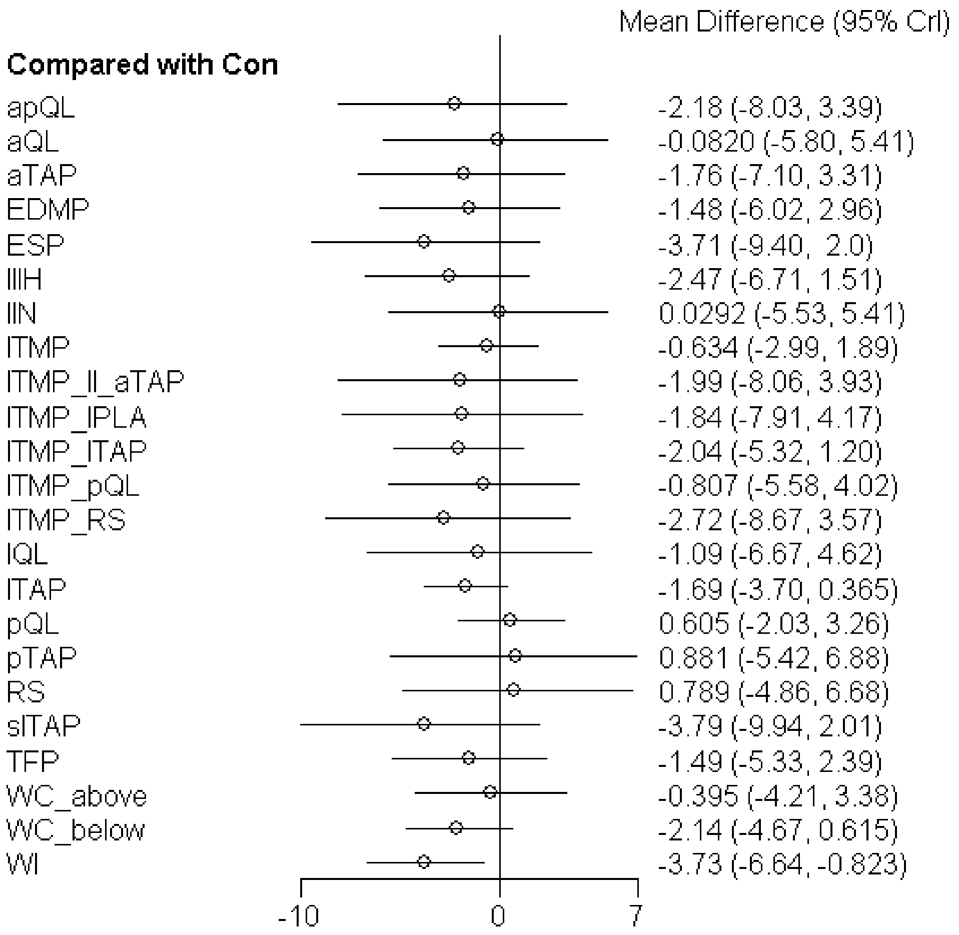

Figure S8. *Cont.*

(**F**) Node splitting plot in random effect model.

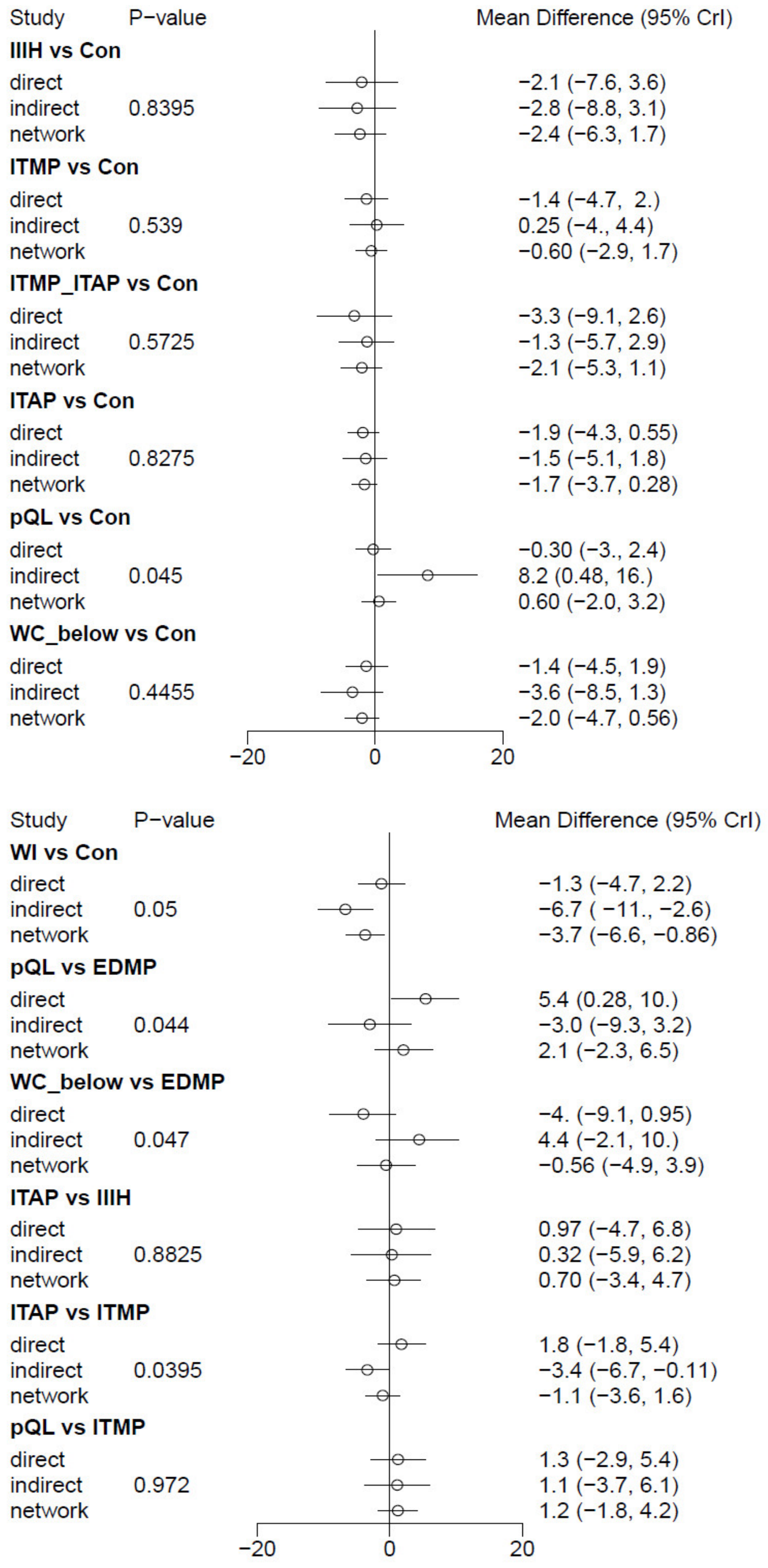

Figure S8. *Cont.*

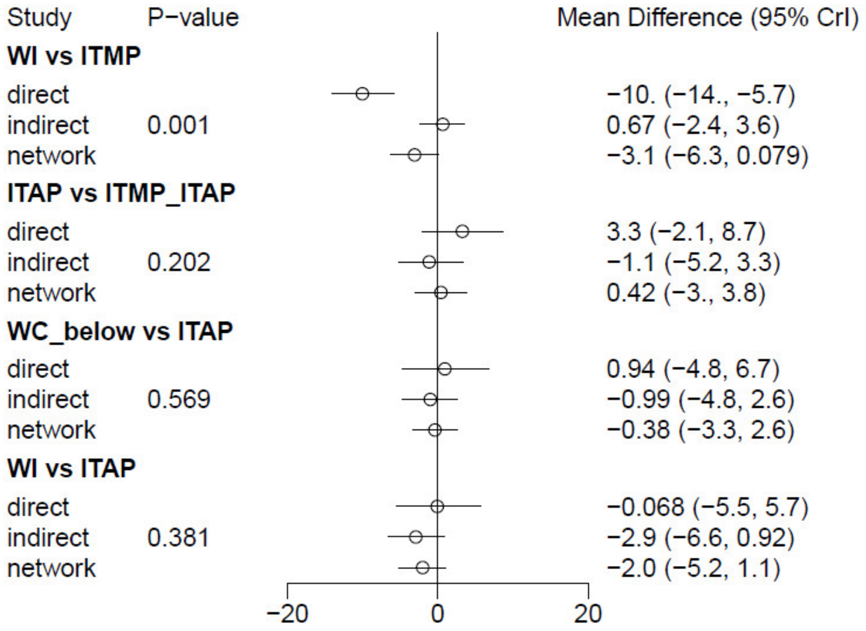

Figure S8. *Cont.*

(**G**) Rankogram in random effect model.

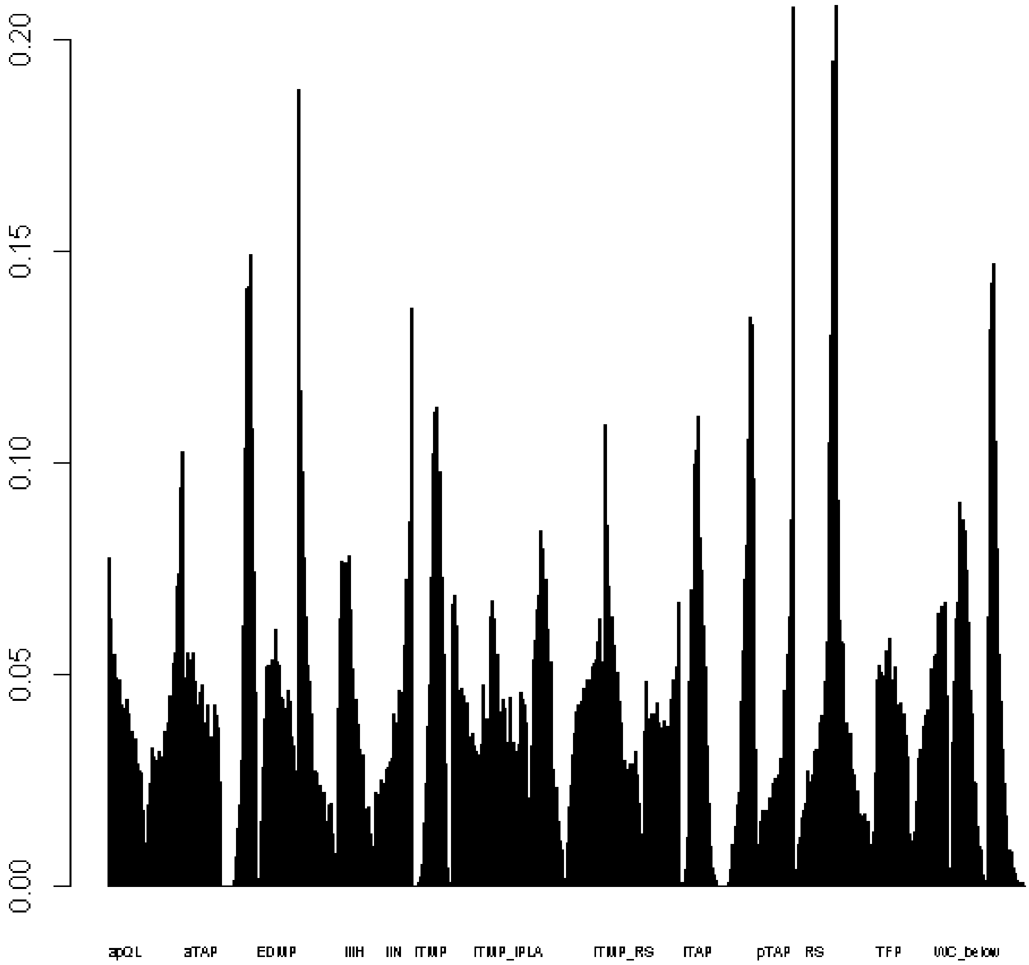

Figure S8. Dynamic pain at 6 h after surgery. (**A**) Trace plot and density plot in fixed effect model; (**B**) Trace plot and density plot in random effect model; (**C**) Gelman-Rubin-Brooks plot in fixed effect model; (**D**) Gelman-Rubin-Brooks plot in random effect model; (**E**) Forest plot comparing with control in random effect model; (**F**) Node splitting plot in random effect model; (**G**) Rankogram in random effect model.

(**A**) Trace plot and density plot in fixed effect model.

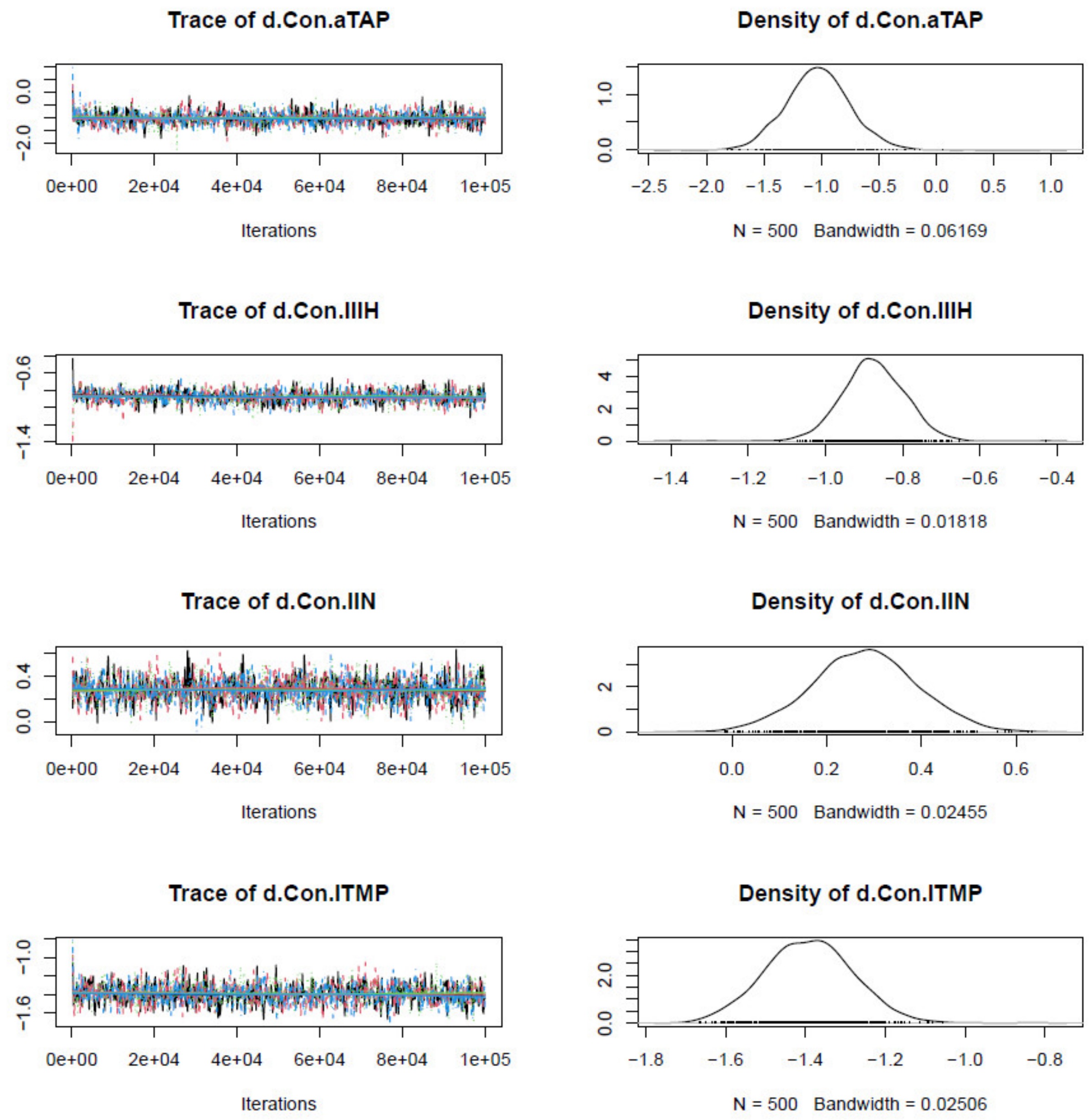

Figure S9. *Cont.*

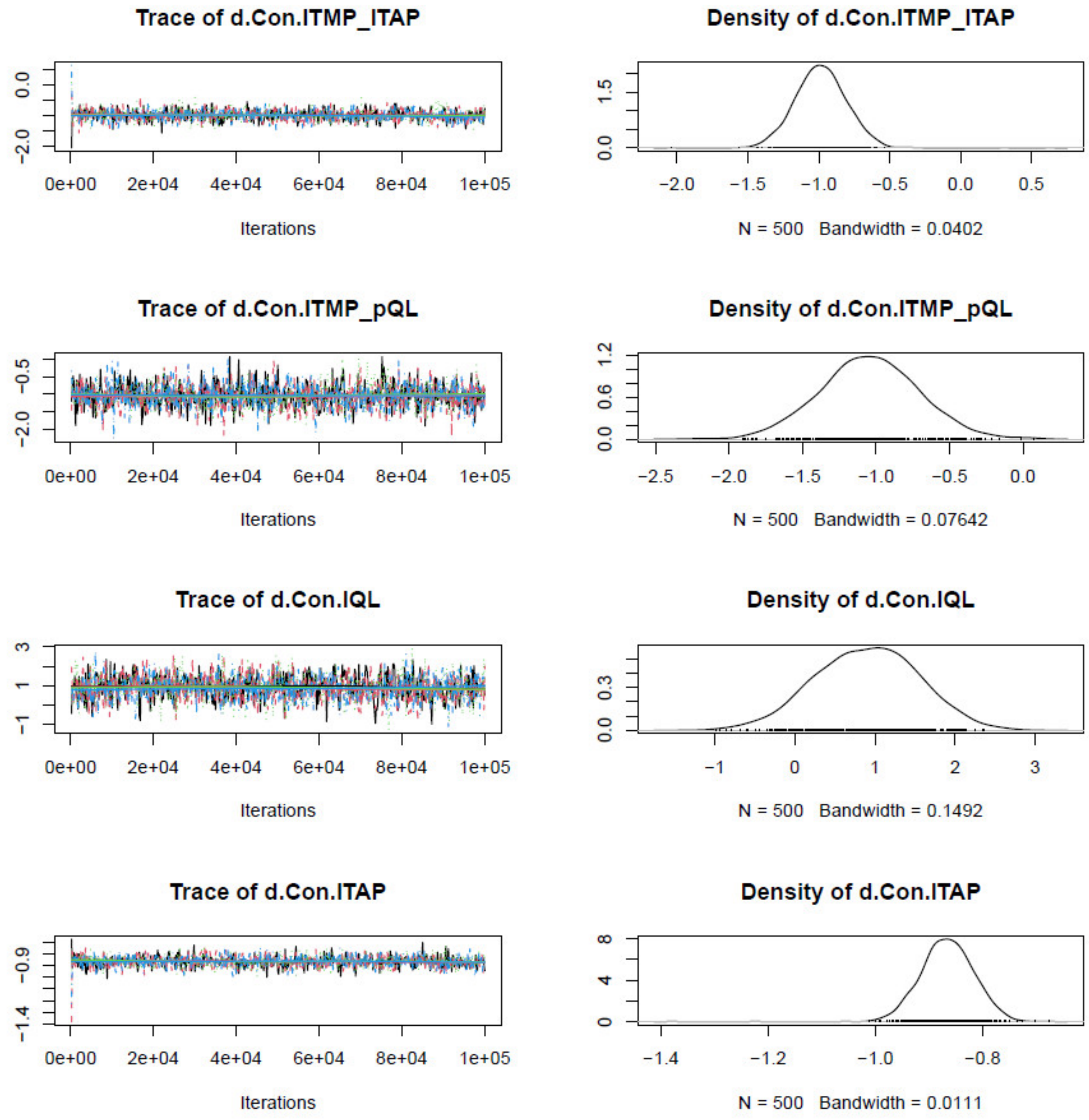

Figure S9. *Cont.*

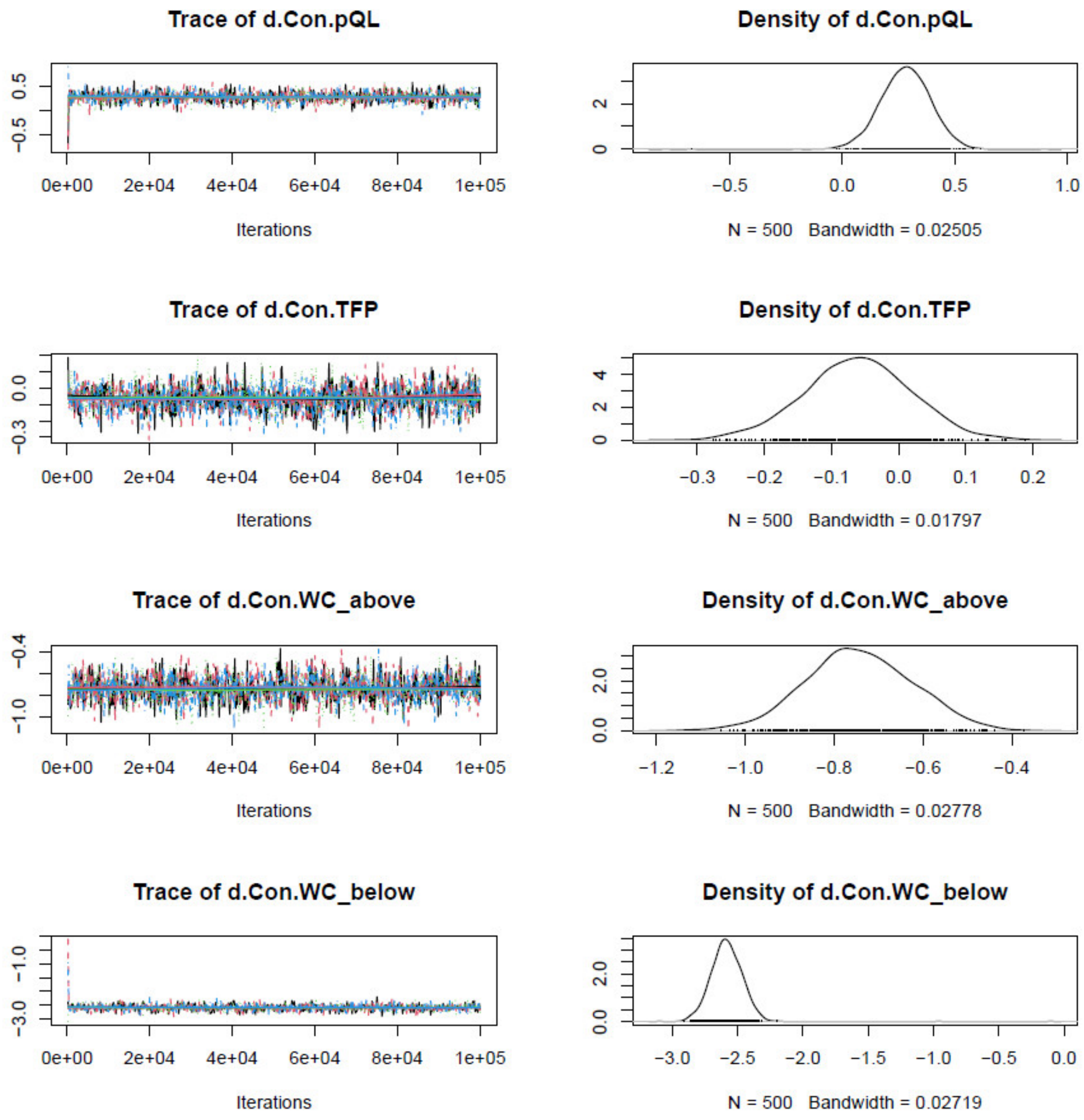

Figure S9. *Cont.*

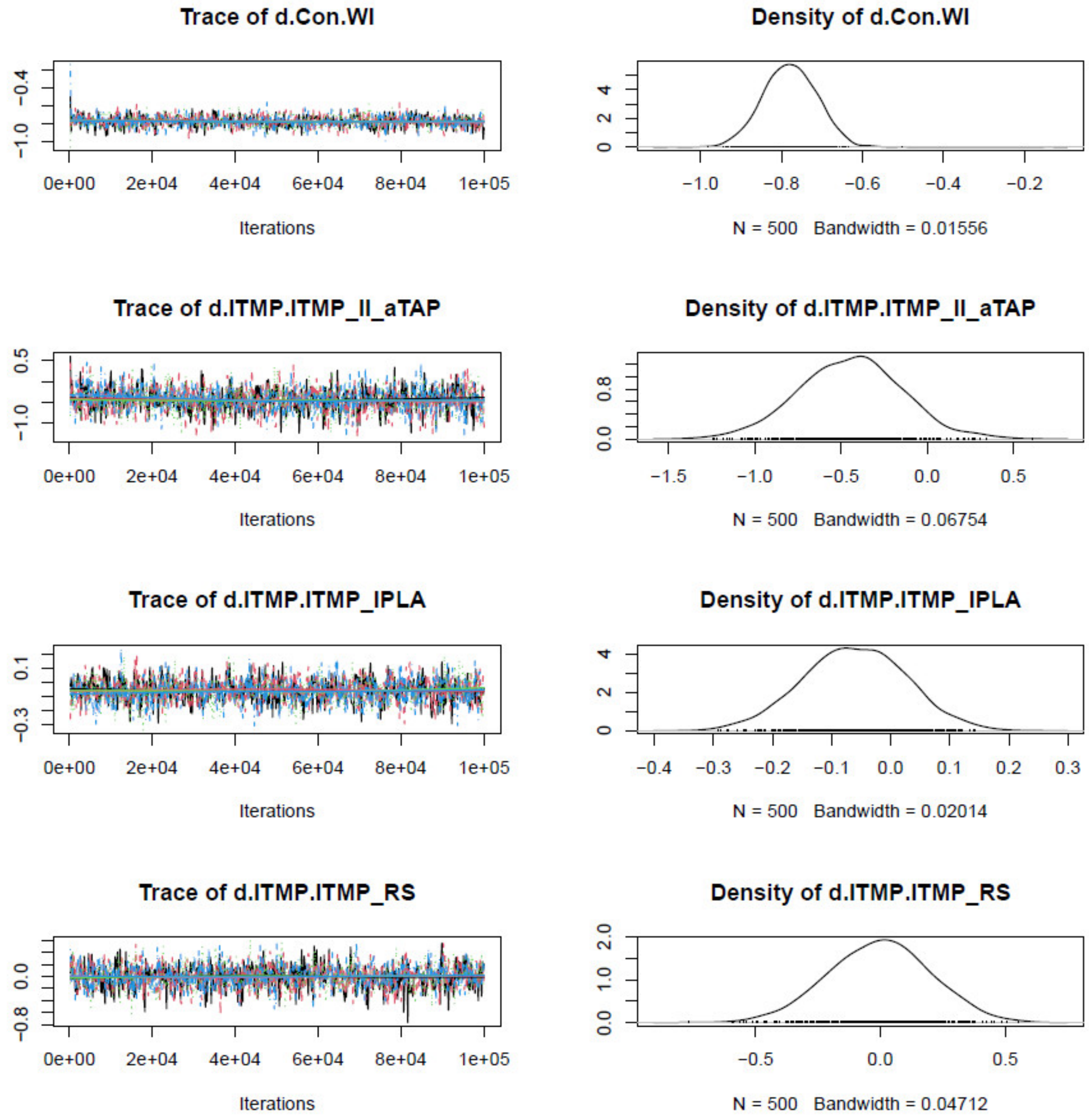

Figure S9. *Cont.*

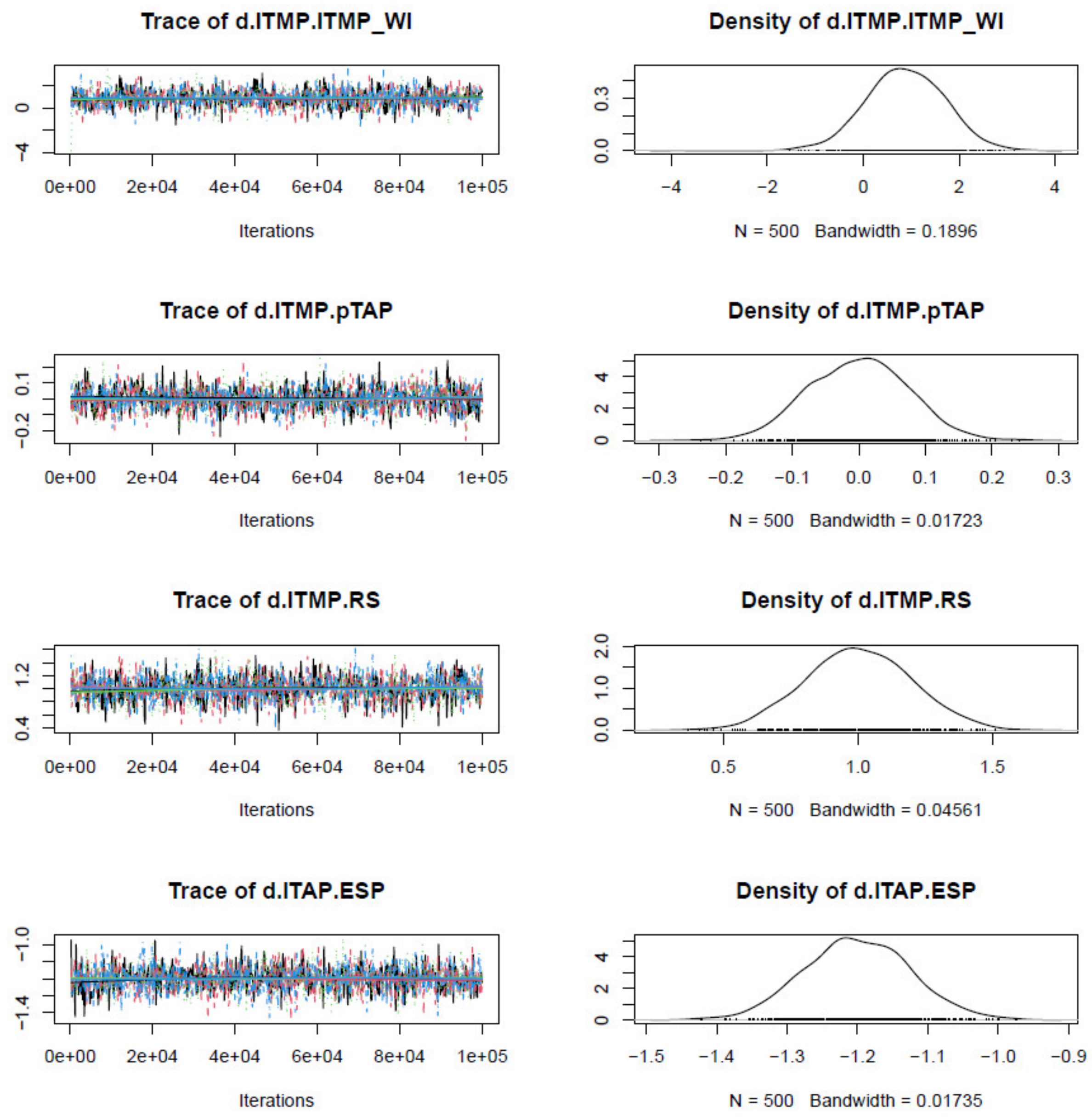

Figure S9. *Cont.*

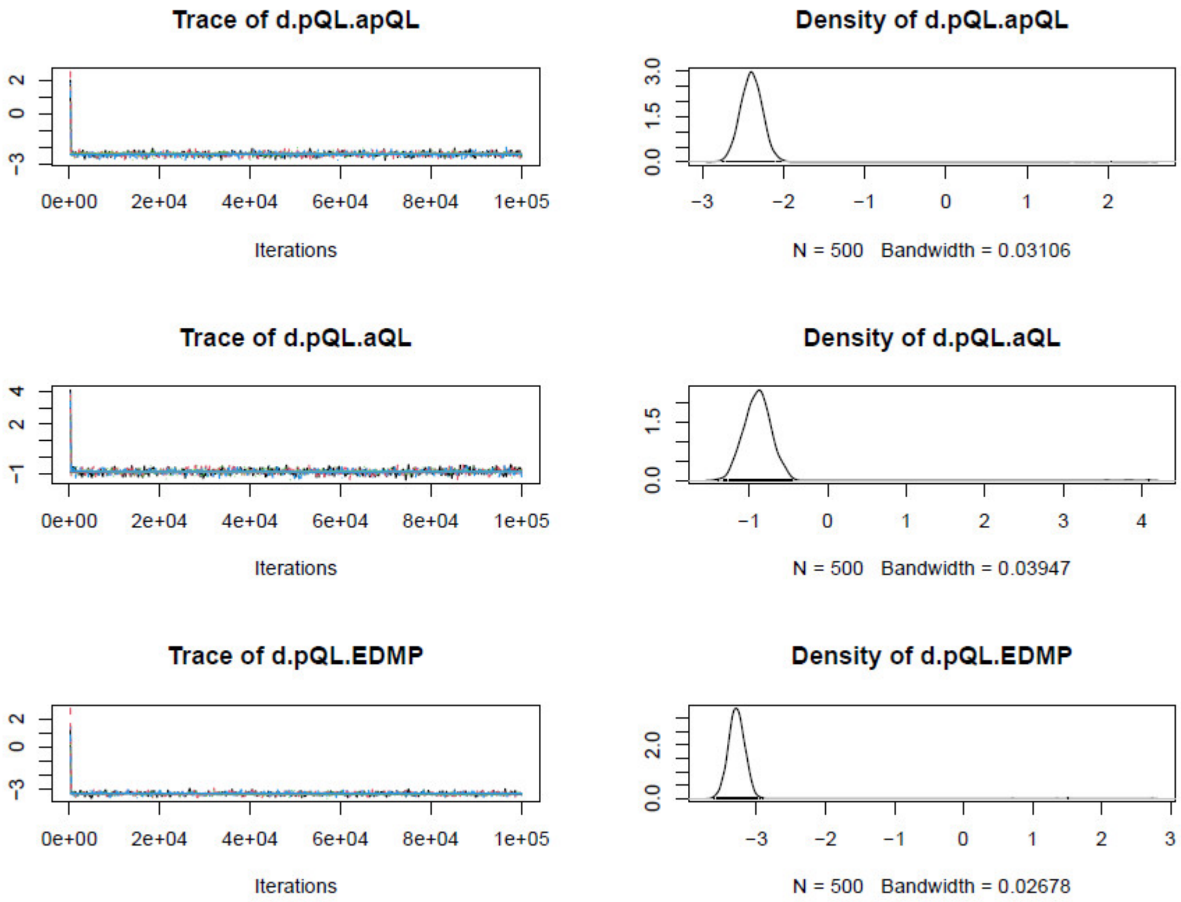

Figure S9. *Cont.*

(**B**) Trace plot and density plot in random effect model.

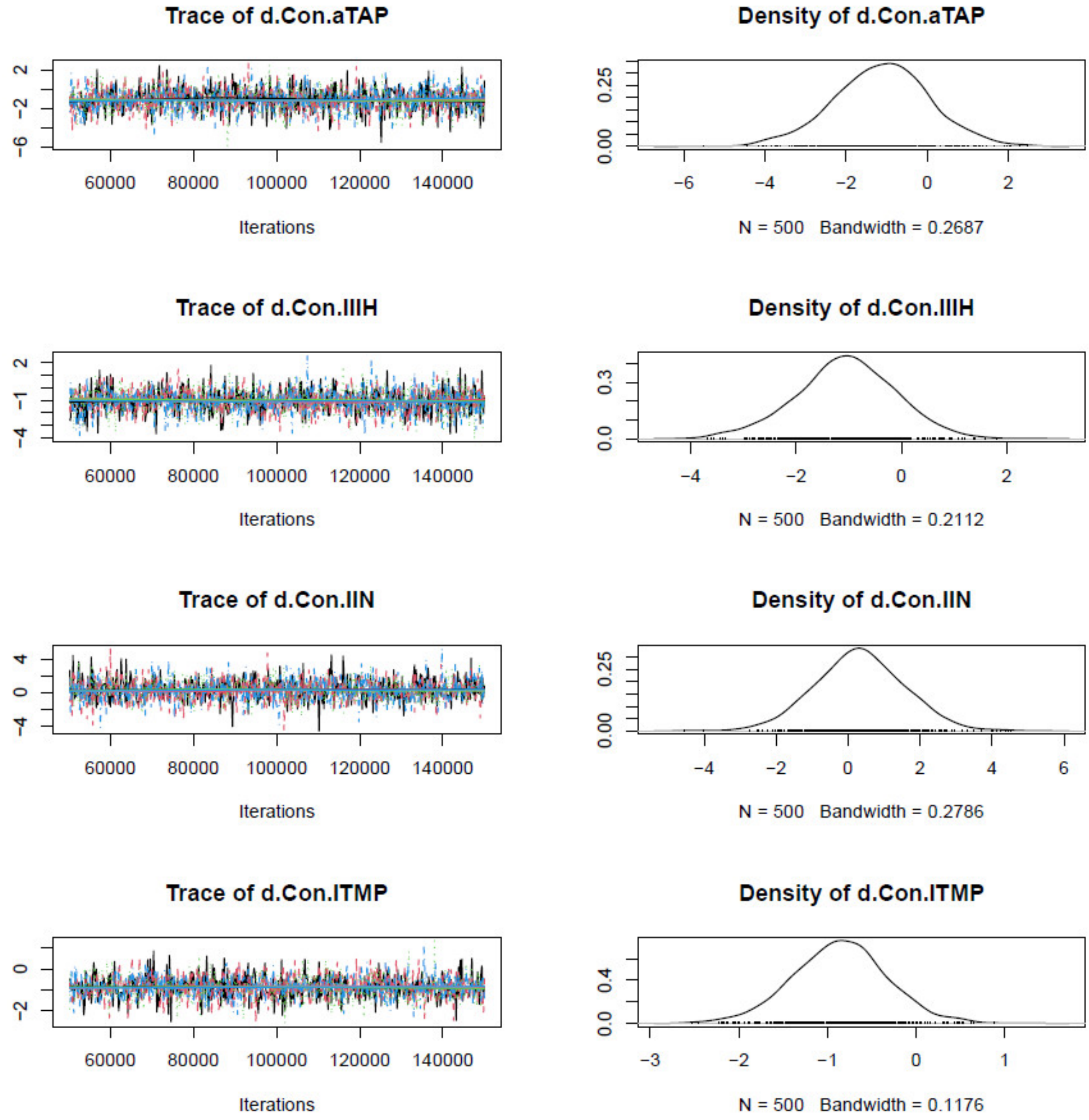

Figure S9. *Cont.*

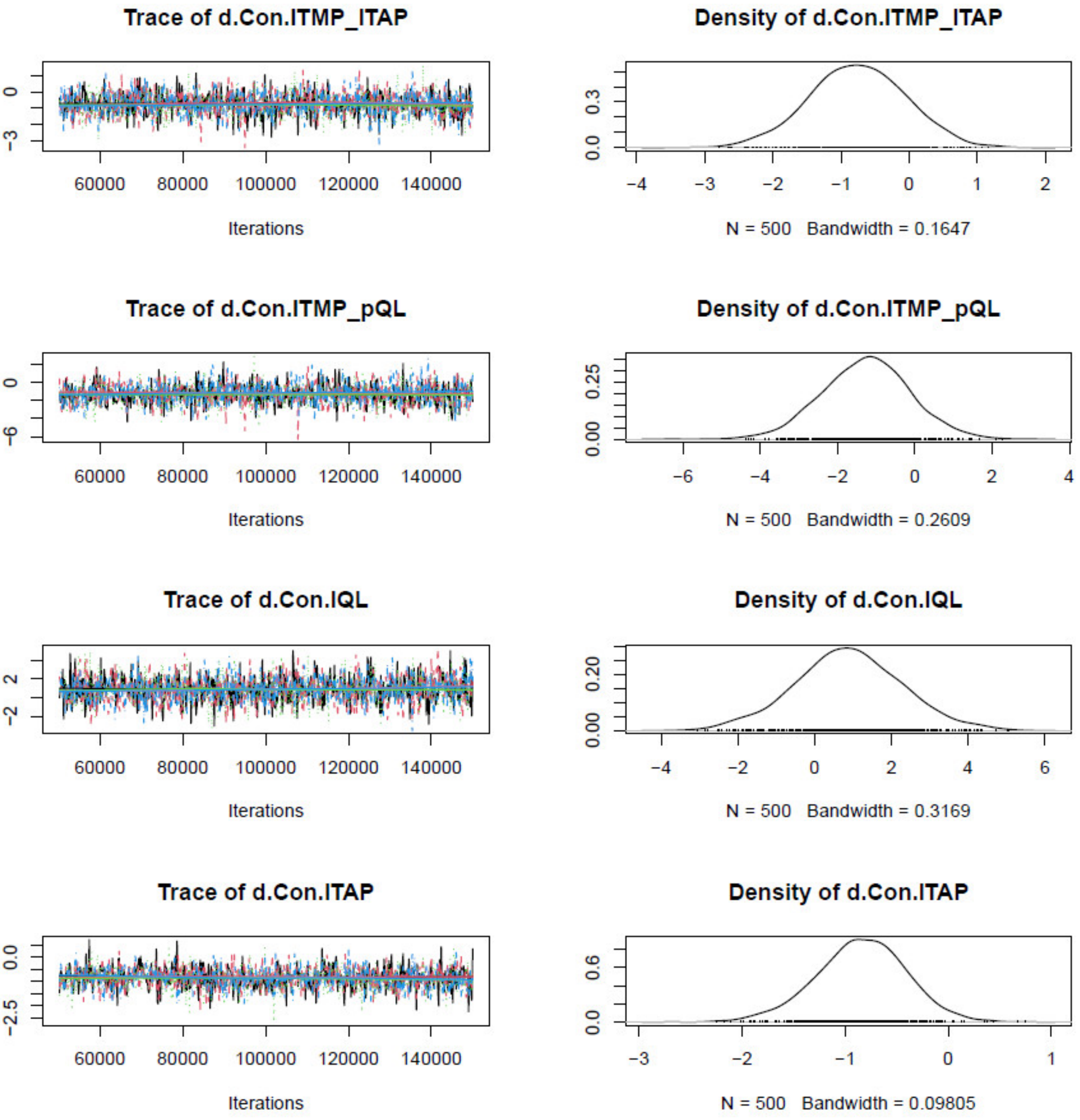

Figure S9. *Cont.*

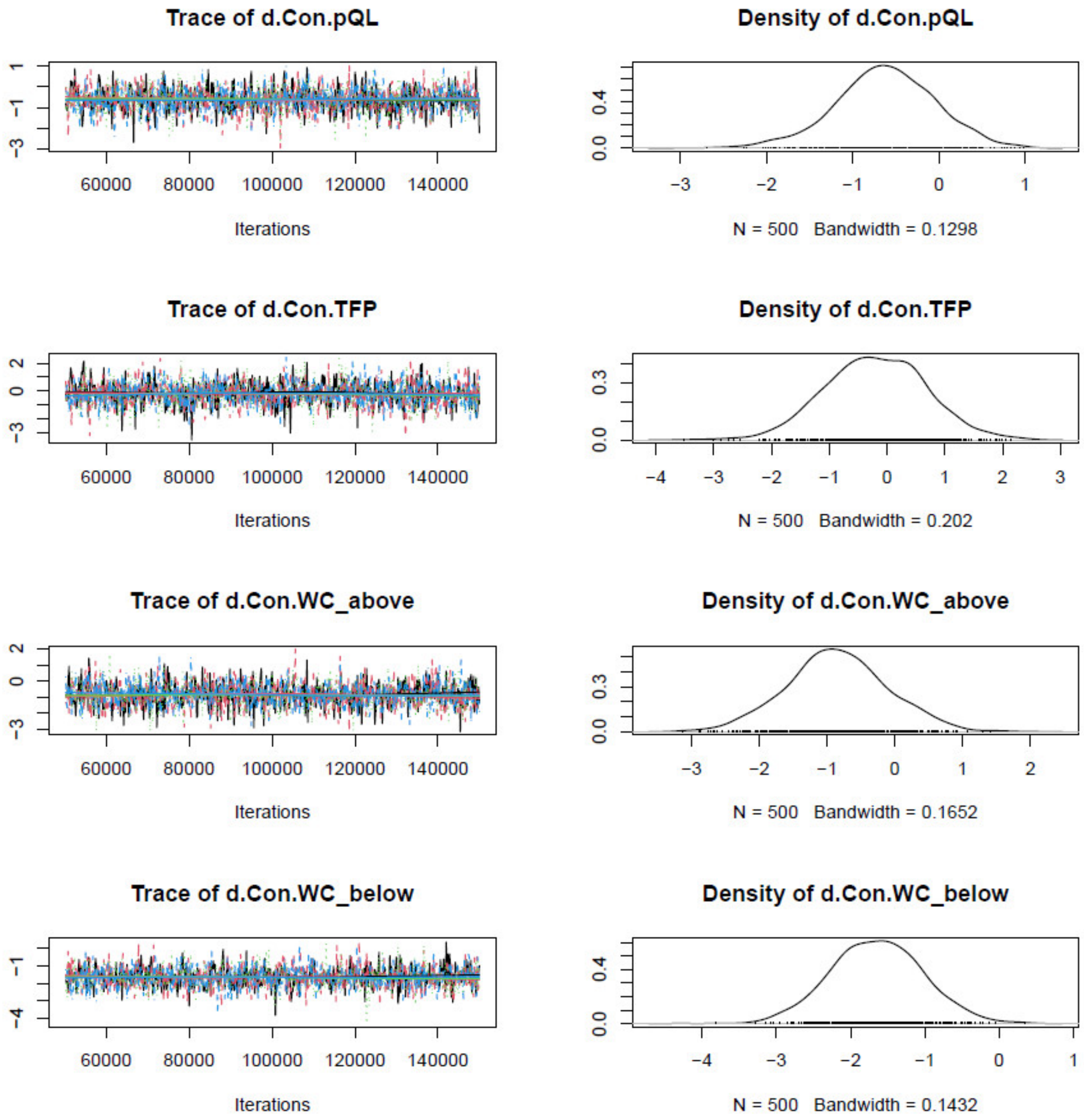

Figure S9. *Cont.*

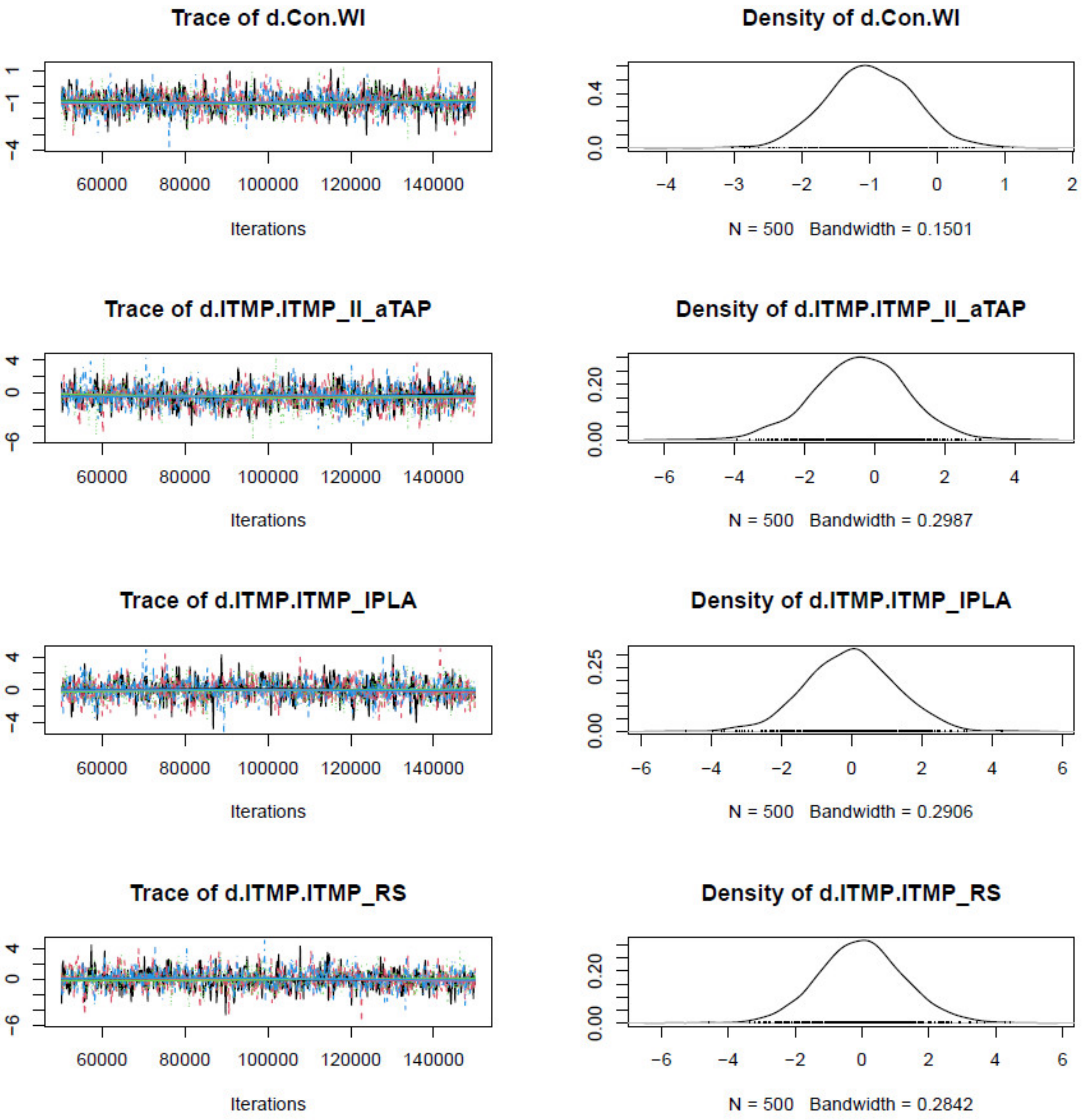

Figure S9. *Cont.*

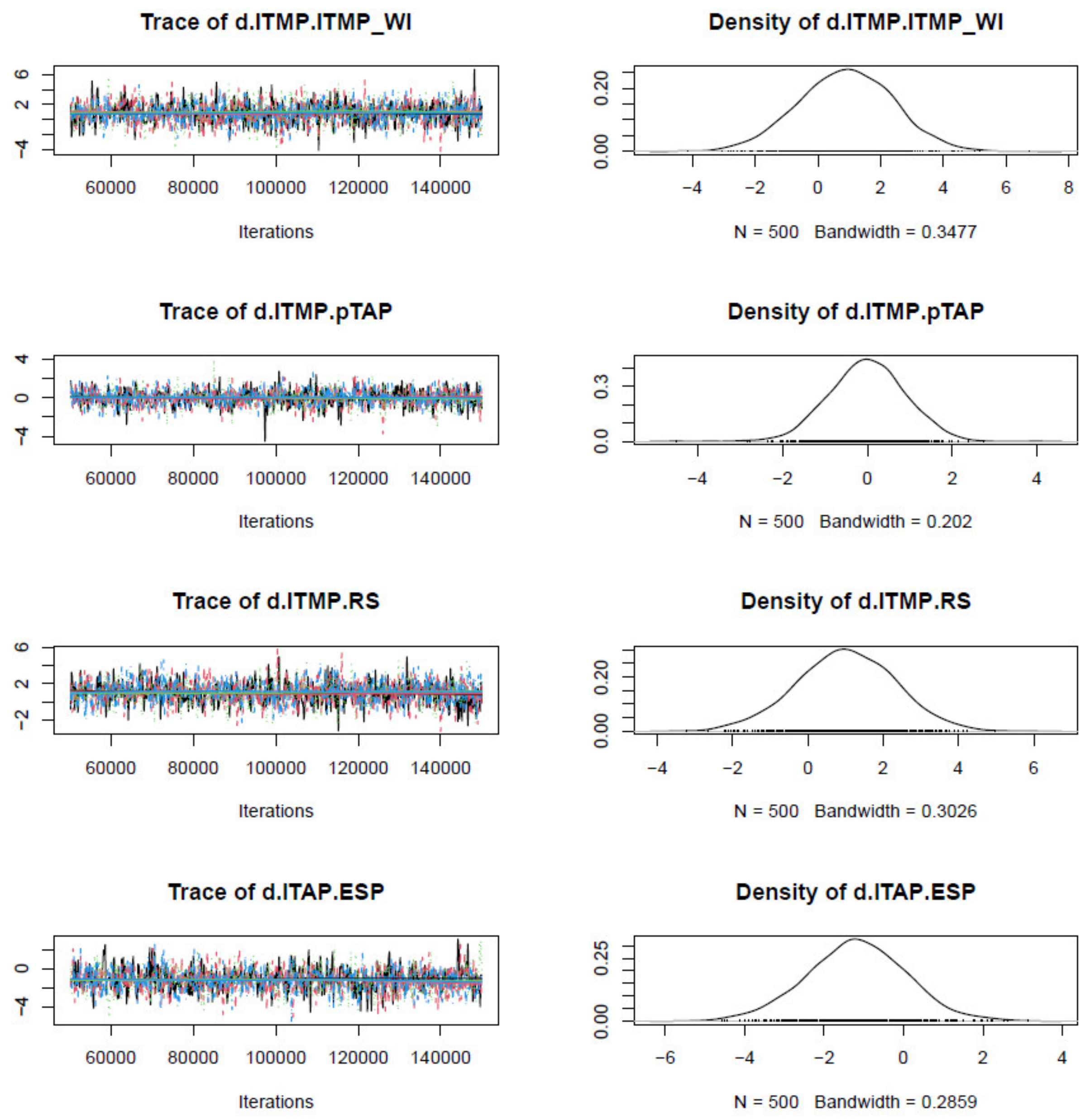

Figure S9. *Cont.*

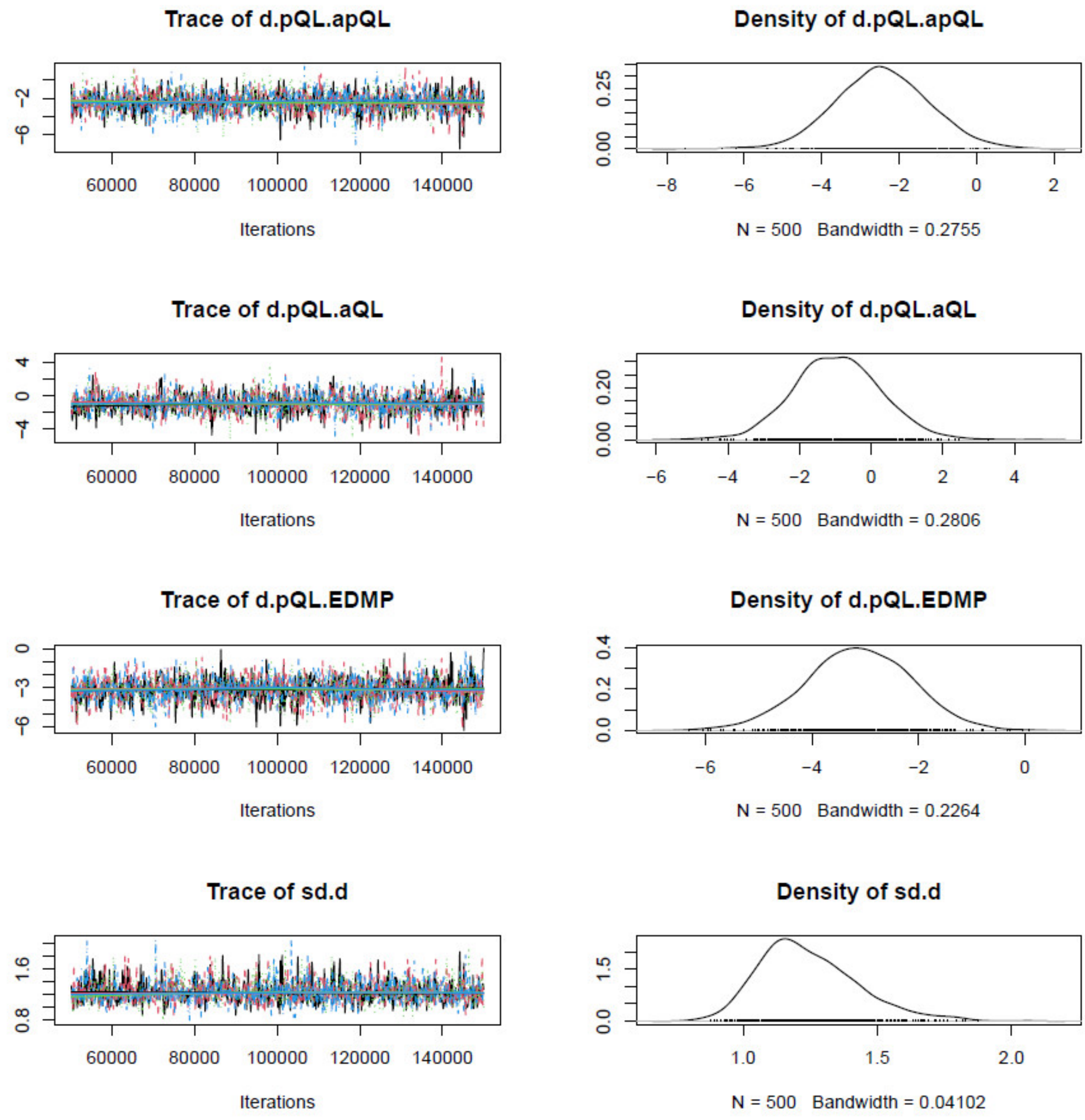

Figure S9. *Cont.*

(**C**) Gelman-Rubin-Brooks plot in fixed effect model.

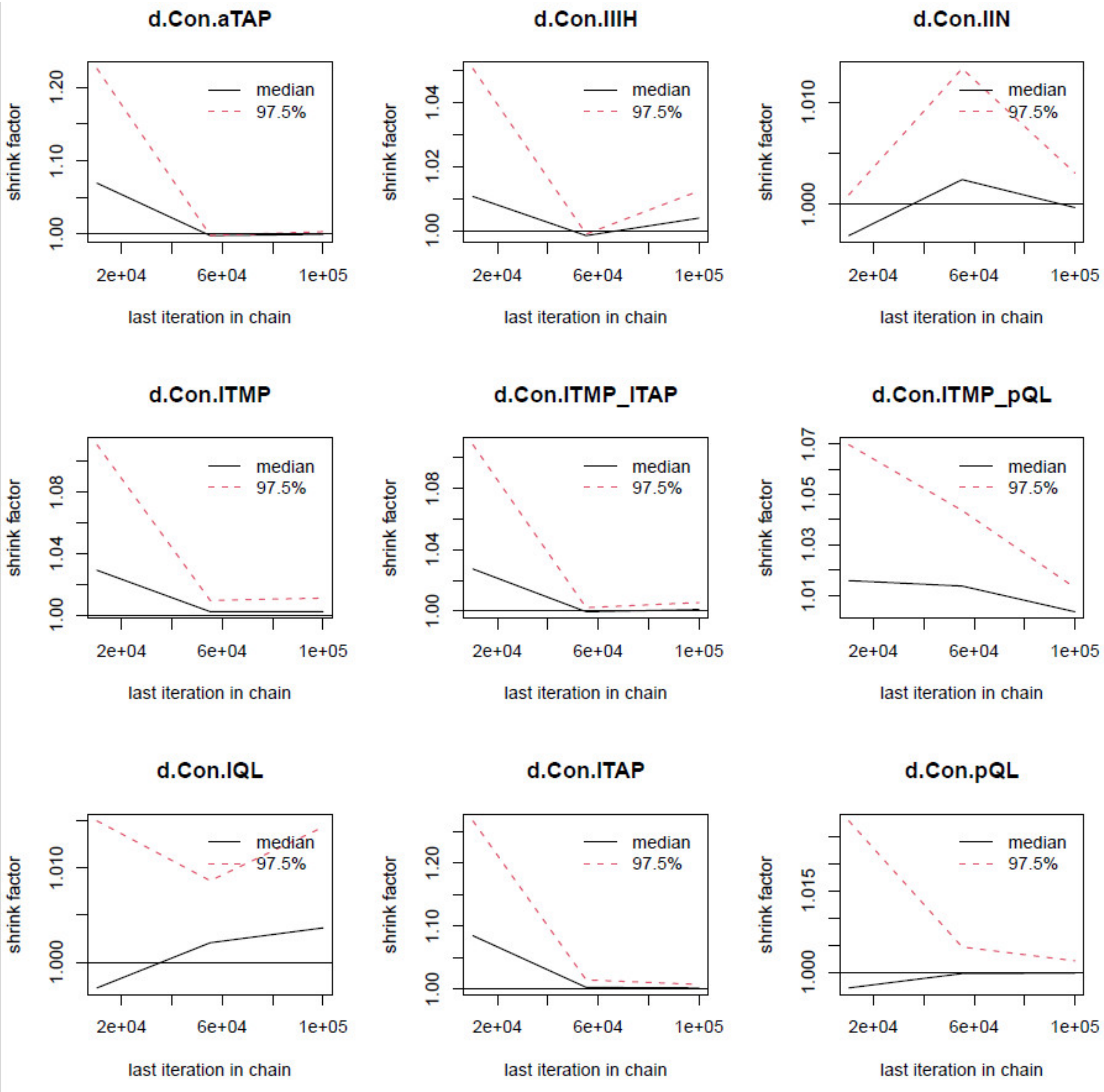

Figure S9. *Cont.*

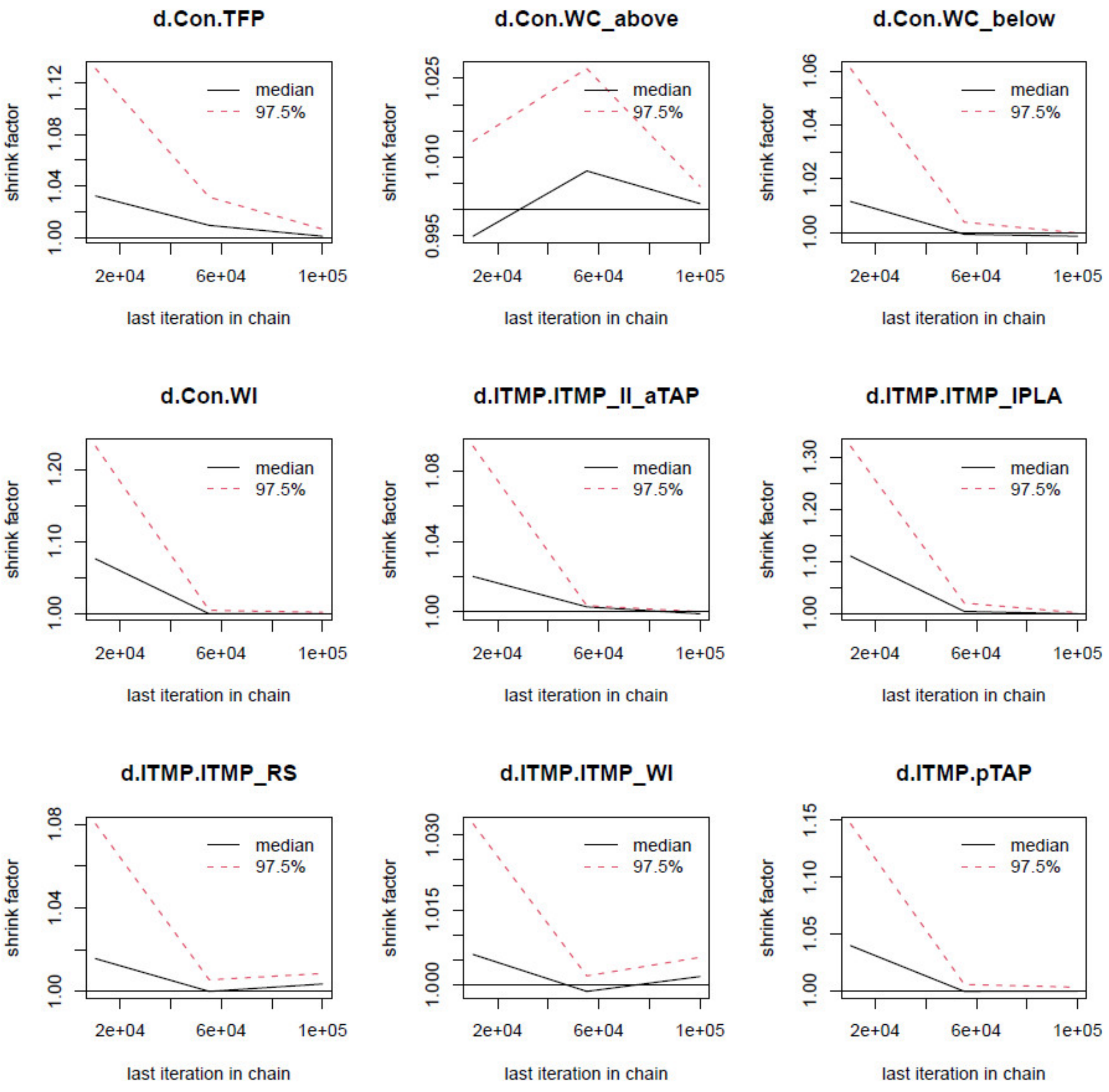

Figure S9. *Cont.*

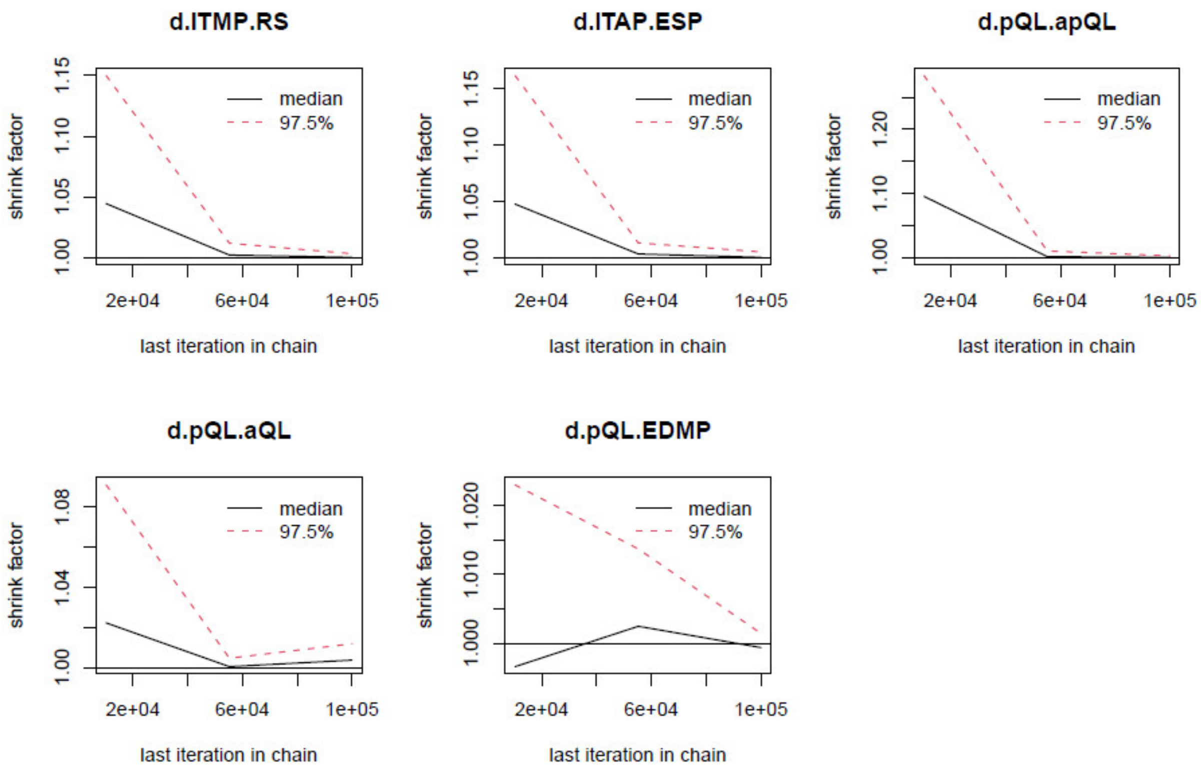

Figure S9. *Cont.*

(**D**) Gelman-Rubin-Brooks plot in random effect model.

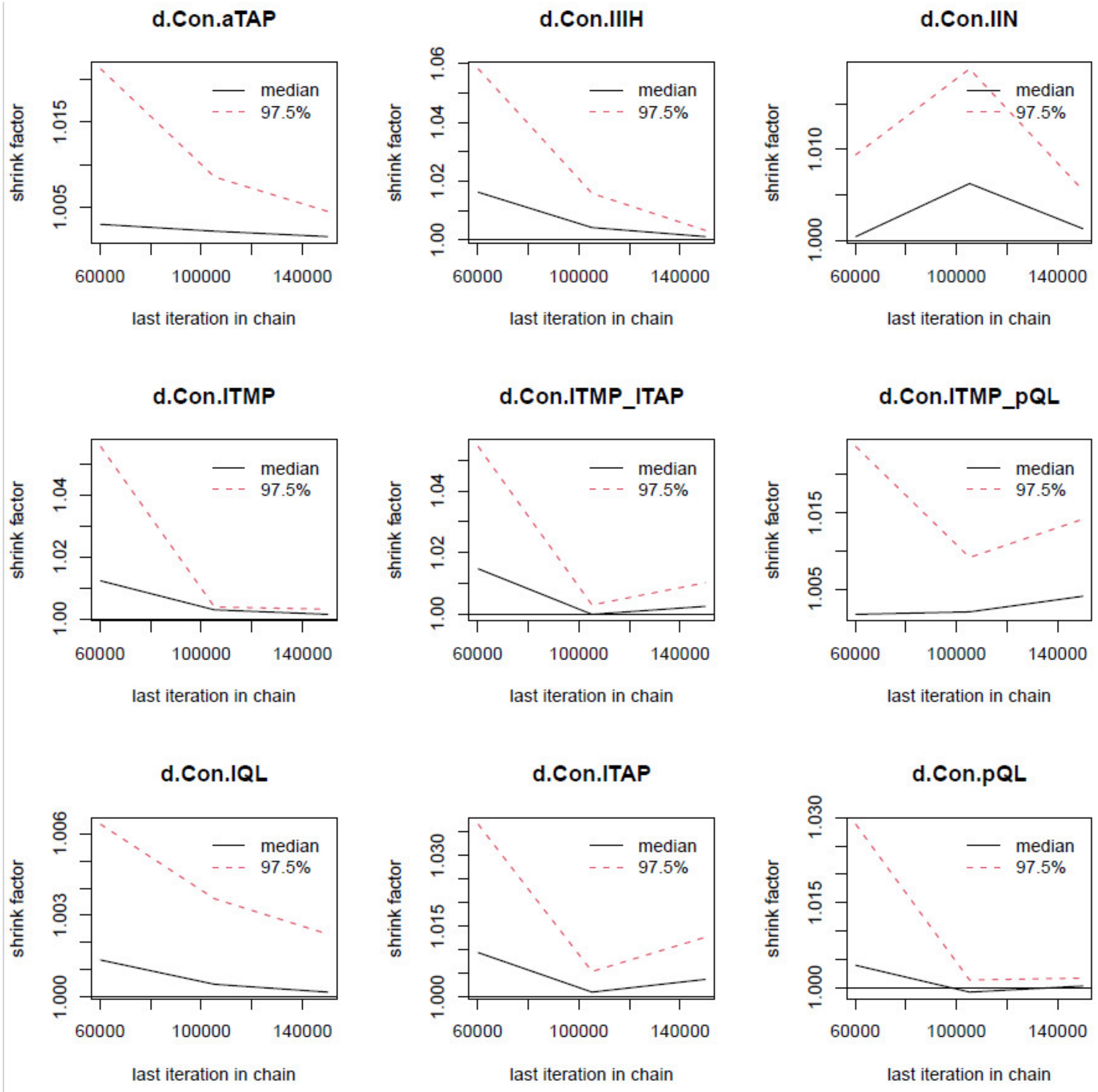

Figure S9. *Cont.*

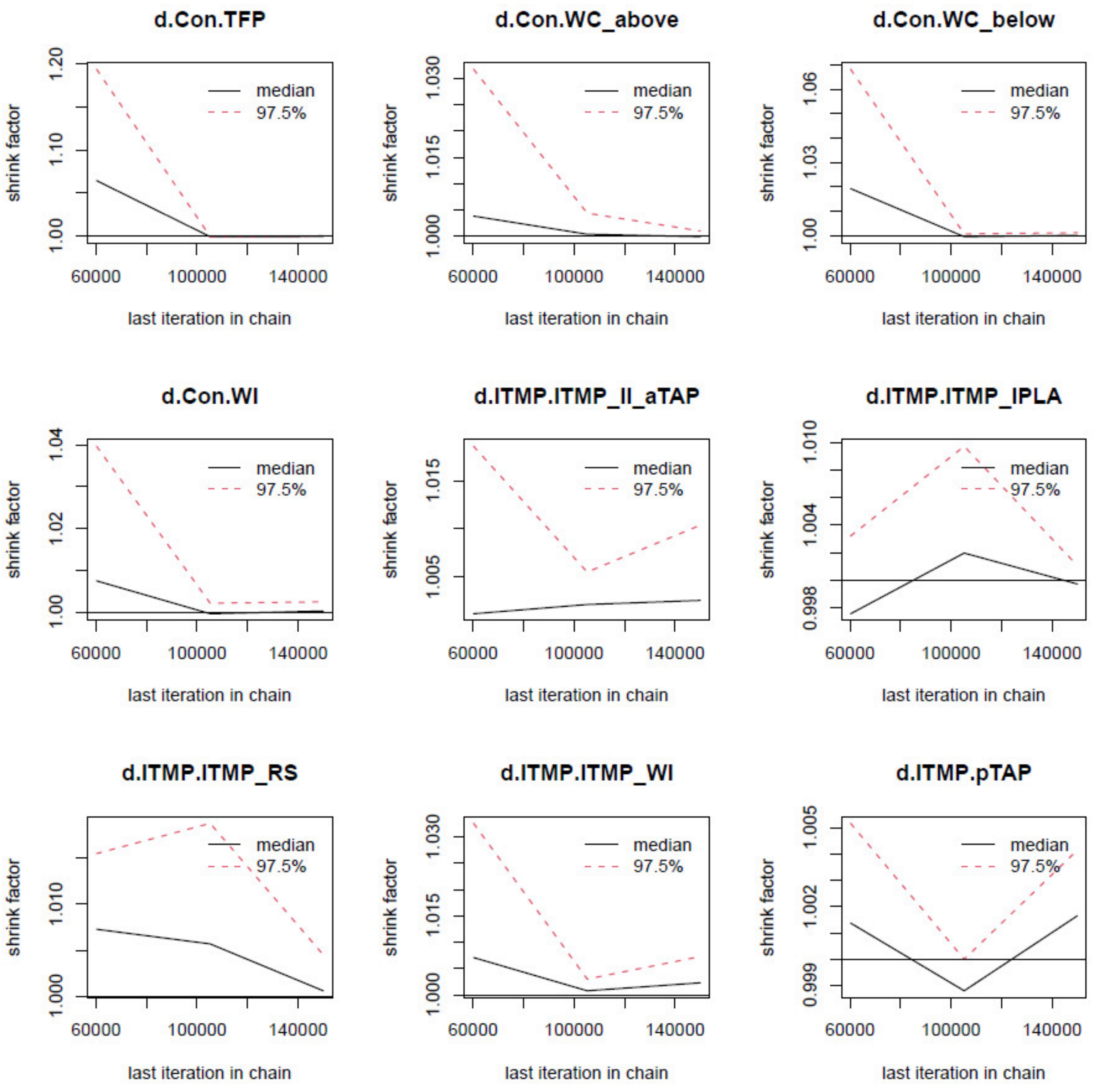

Figure S9. *Cont.*

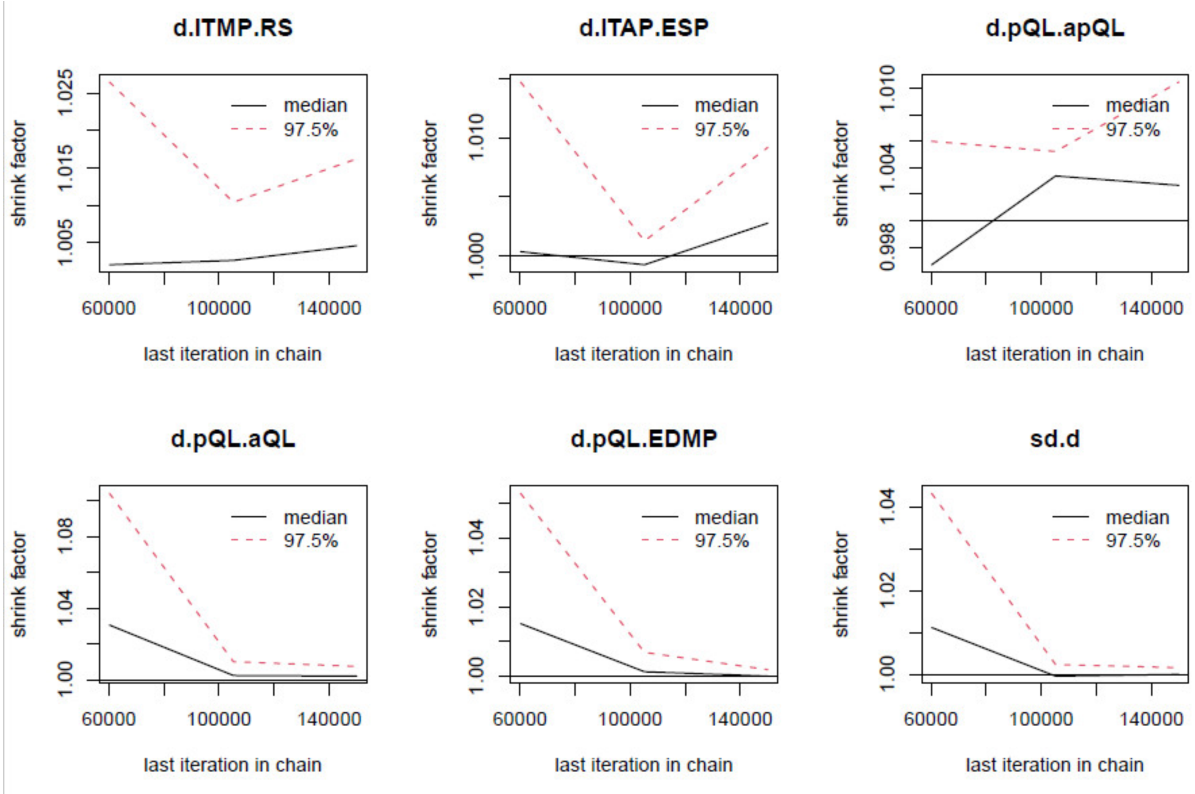

Figure S9. *Cont.*

(E) Forest plot comparing with control in random effect model.

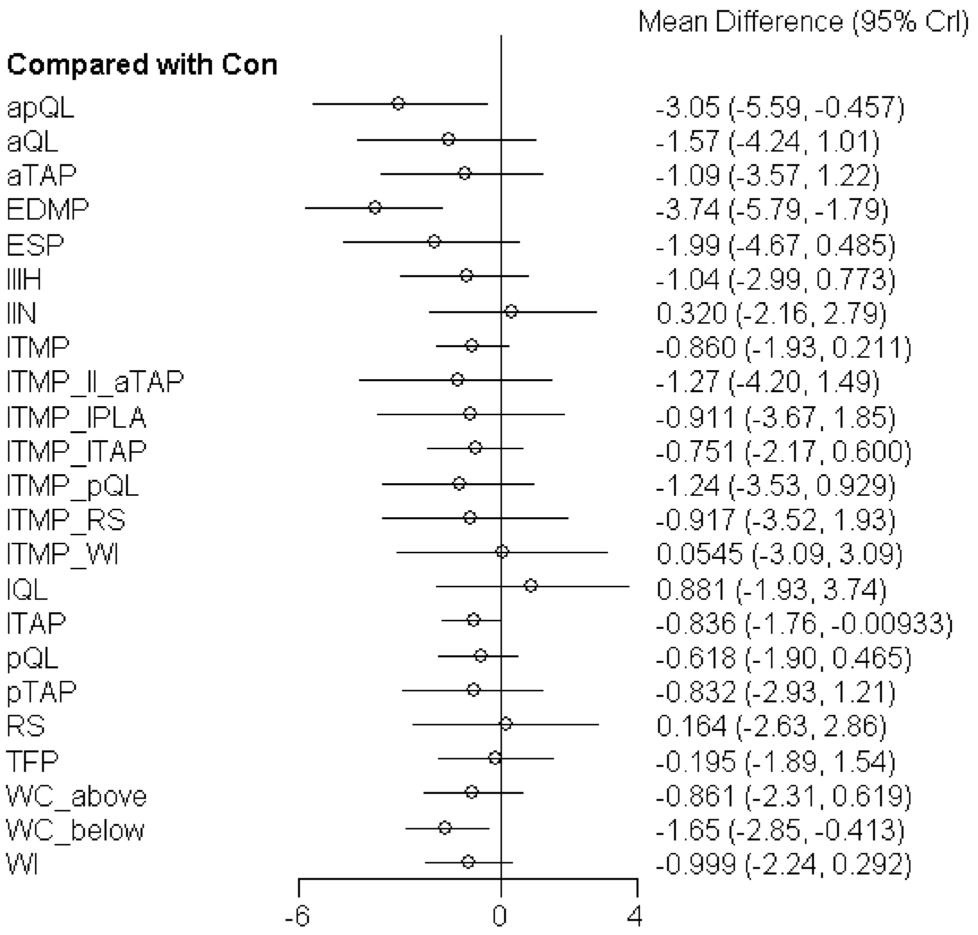

Figure S9. *Cont.*

(**F**) Node splitting plot in random effect model.

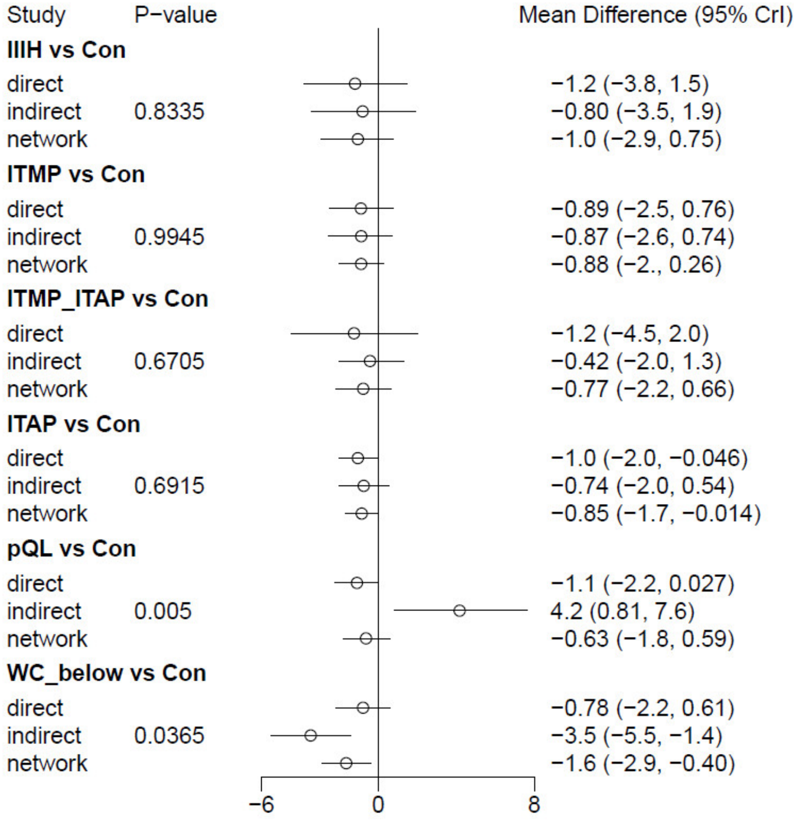

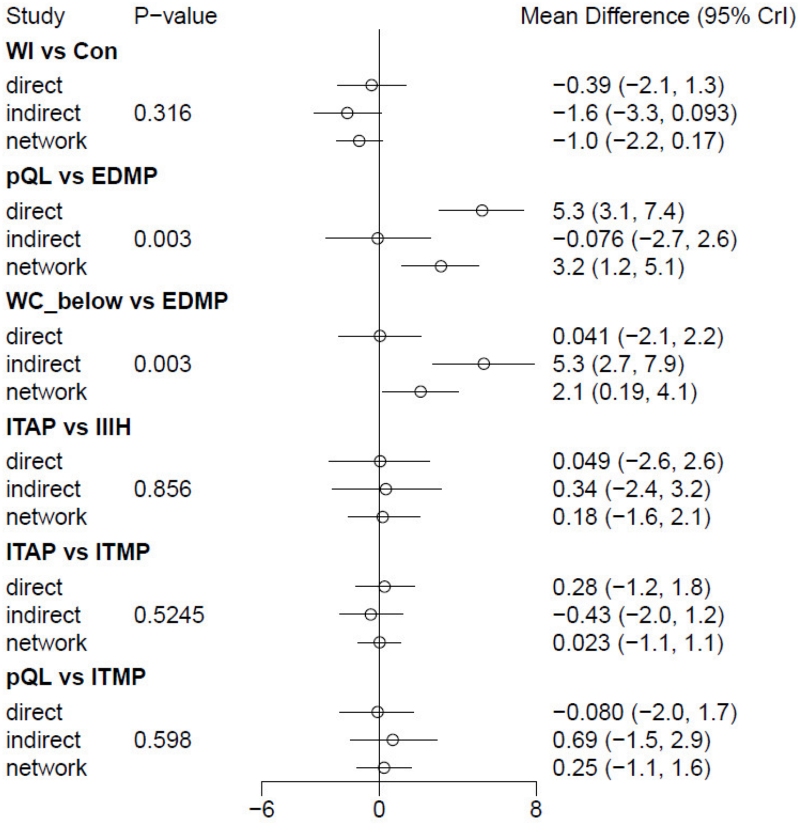

Figure S9. *Cont.*

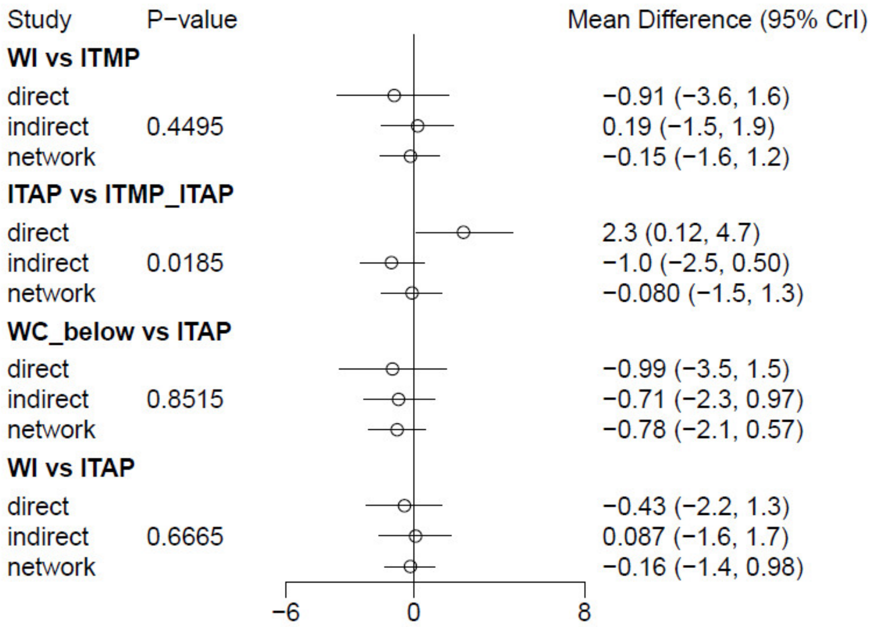

(**G**) Rankogram in random effect model.

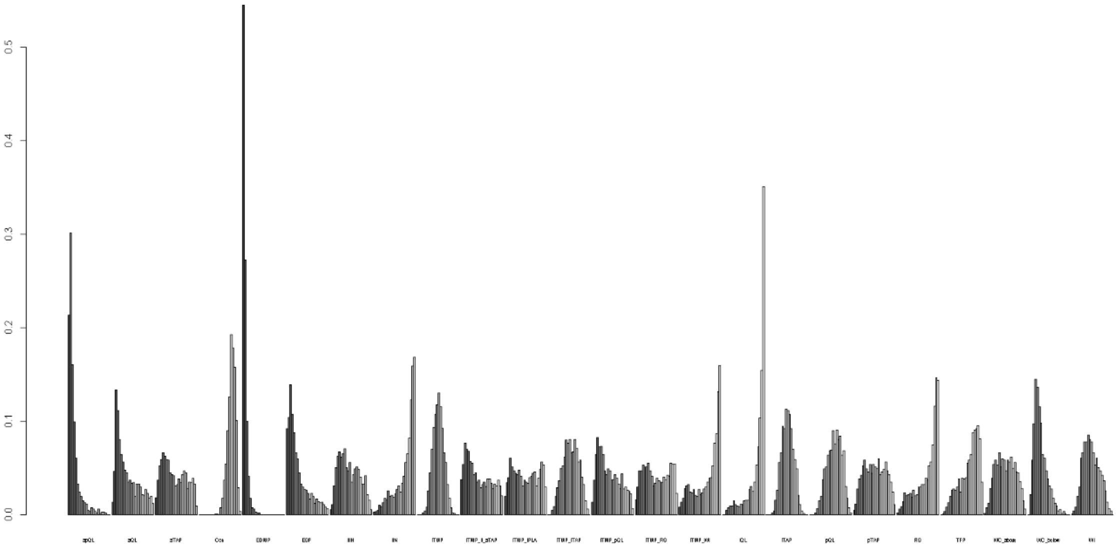

Figure S9. Dynamic pain at 24 h after surgery. (**A**) Trace plot and density plot in fixed effect model; (**B**) Trace plot and density plot in random effect model; (**C**) Gelman-Rubin-Brooks plot in fixed effect model; (**D**) Gelman-Rubin-Brooks plot in random effect model; (**E**) Forest plot comparing with control in random effect model; (**F**) Node splitting plot in random effect model; (**G**) Rankogram in random effect model.

(**A**) Trace plot and density plot in fixed effect model.

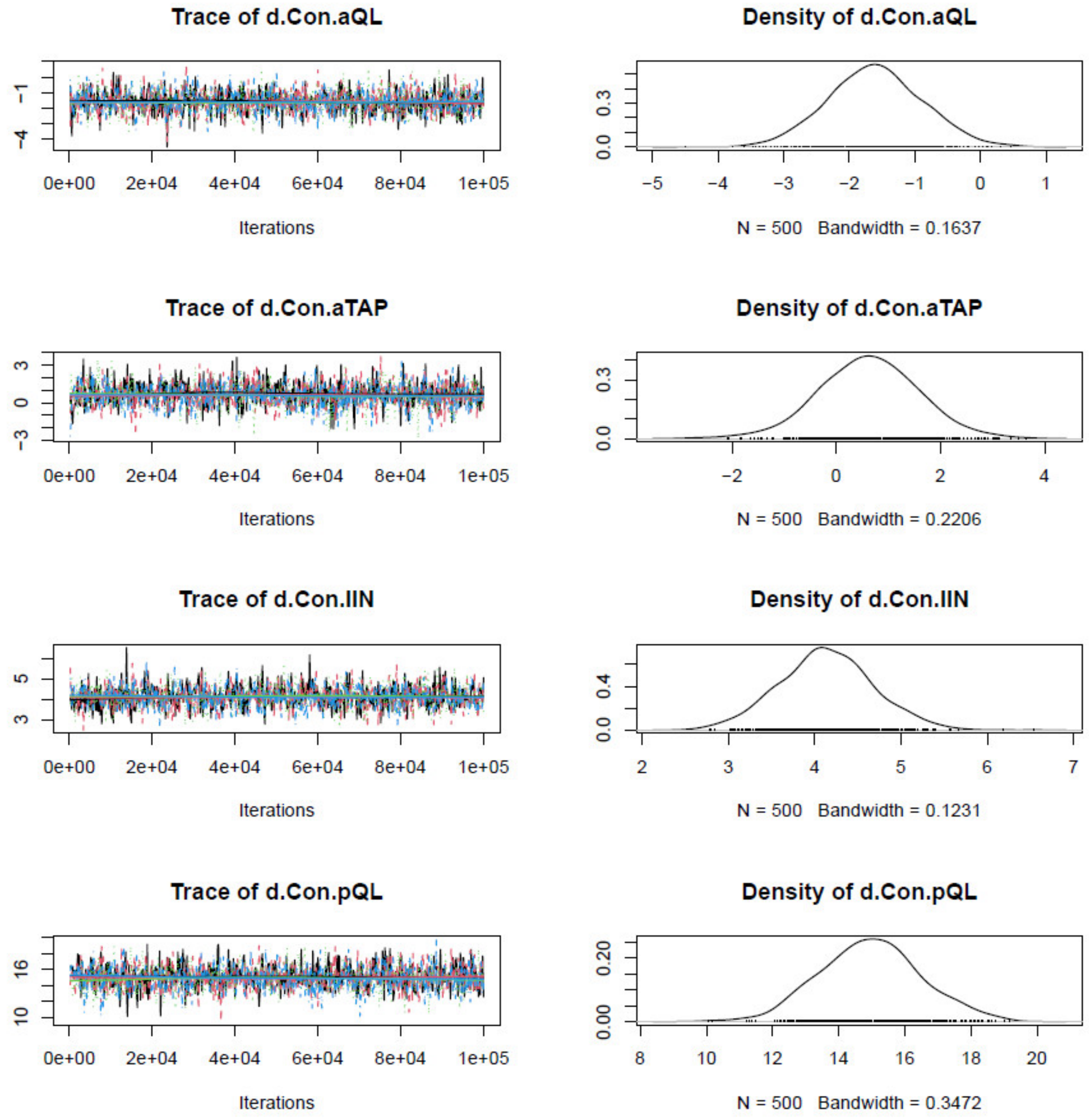

Figure S10. *Cont.*

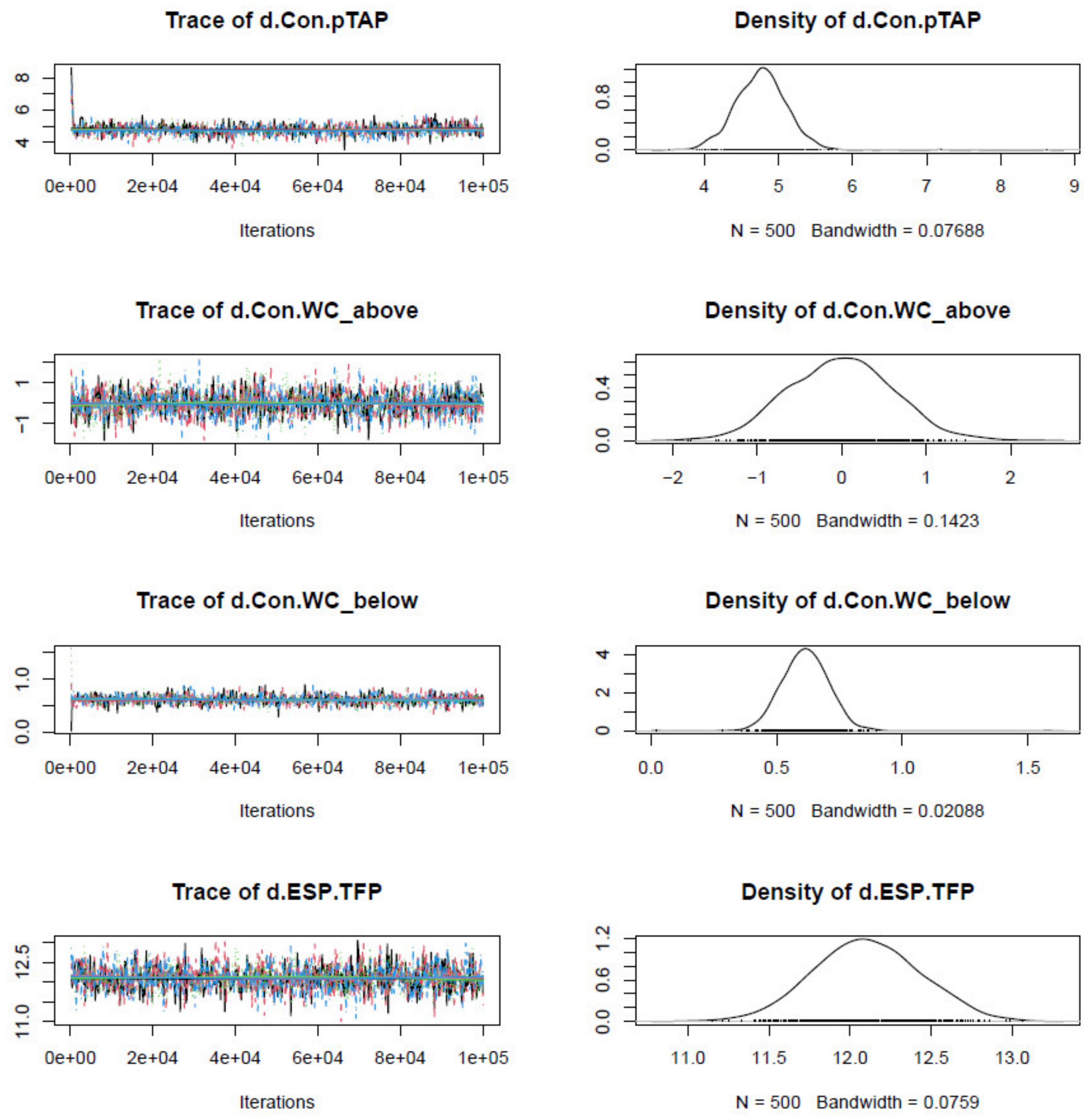

Figure S10. *Cont.*

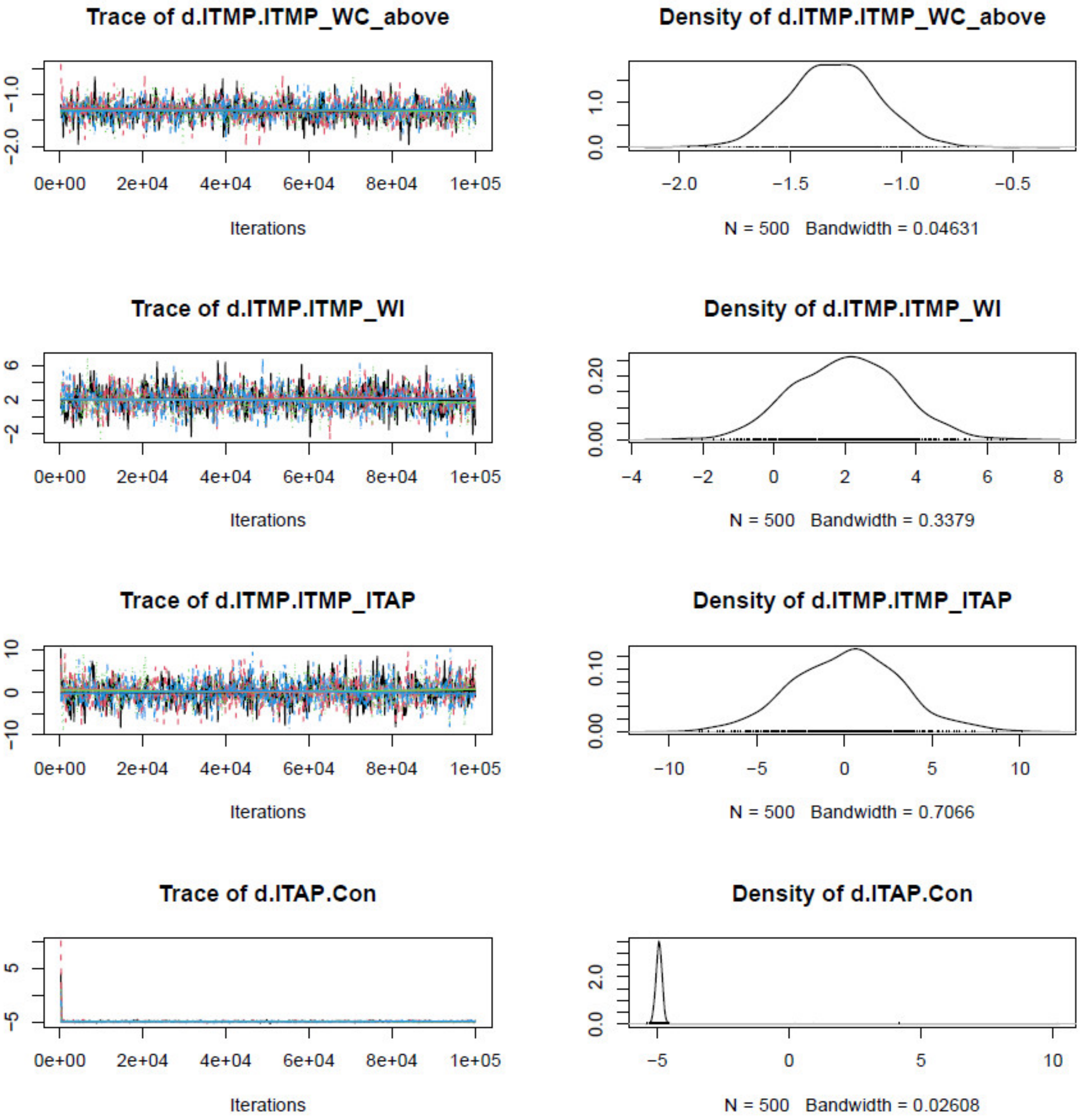

Figure S10. *Cont.*

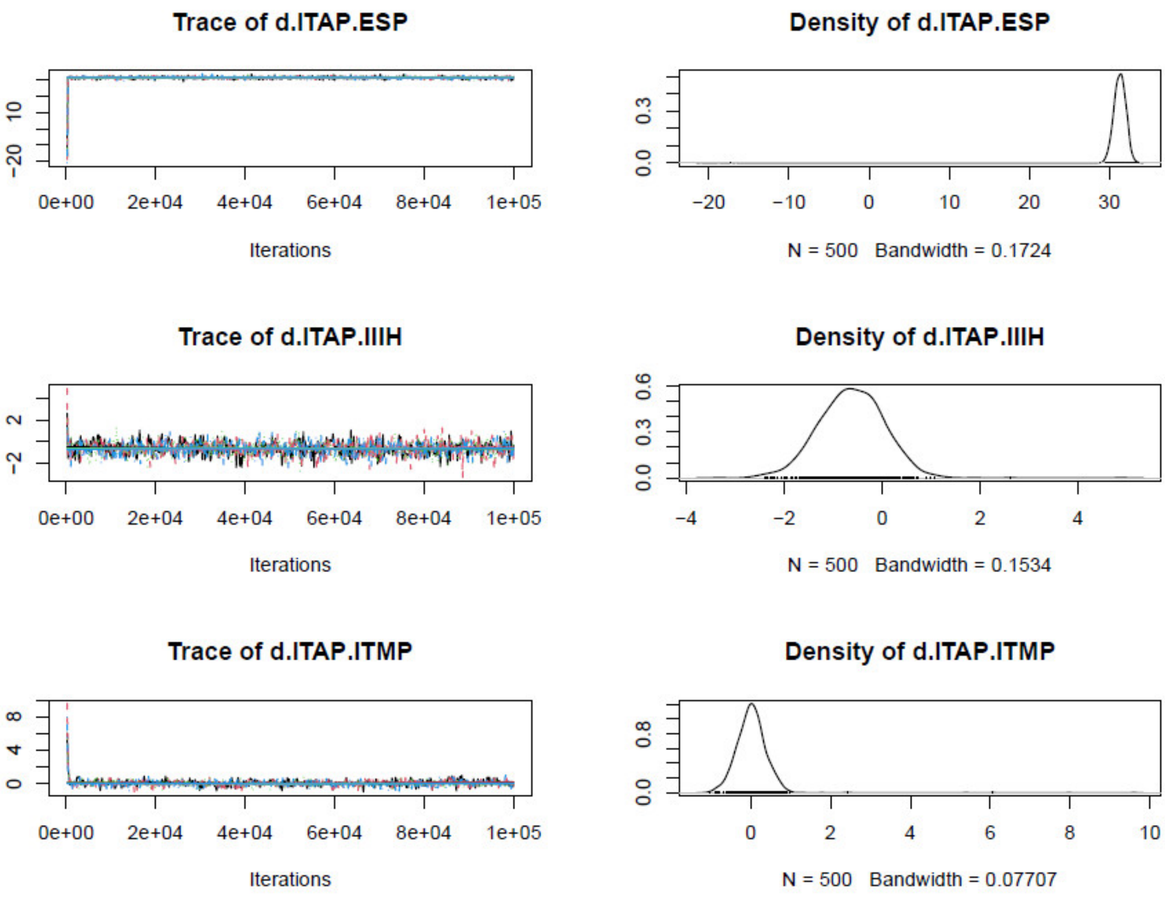

Figure S10. *Cont.*

(**B**) Trace plot and density plot in random effect model.

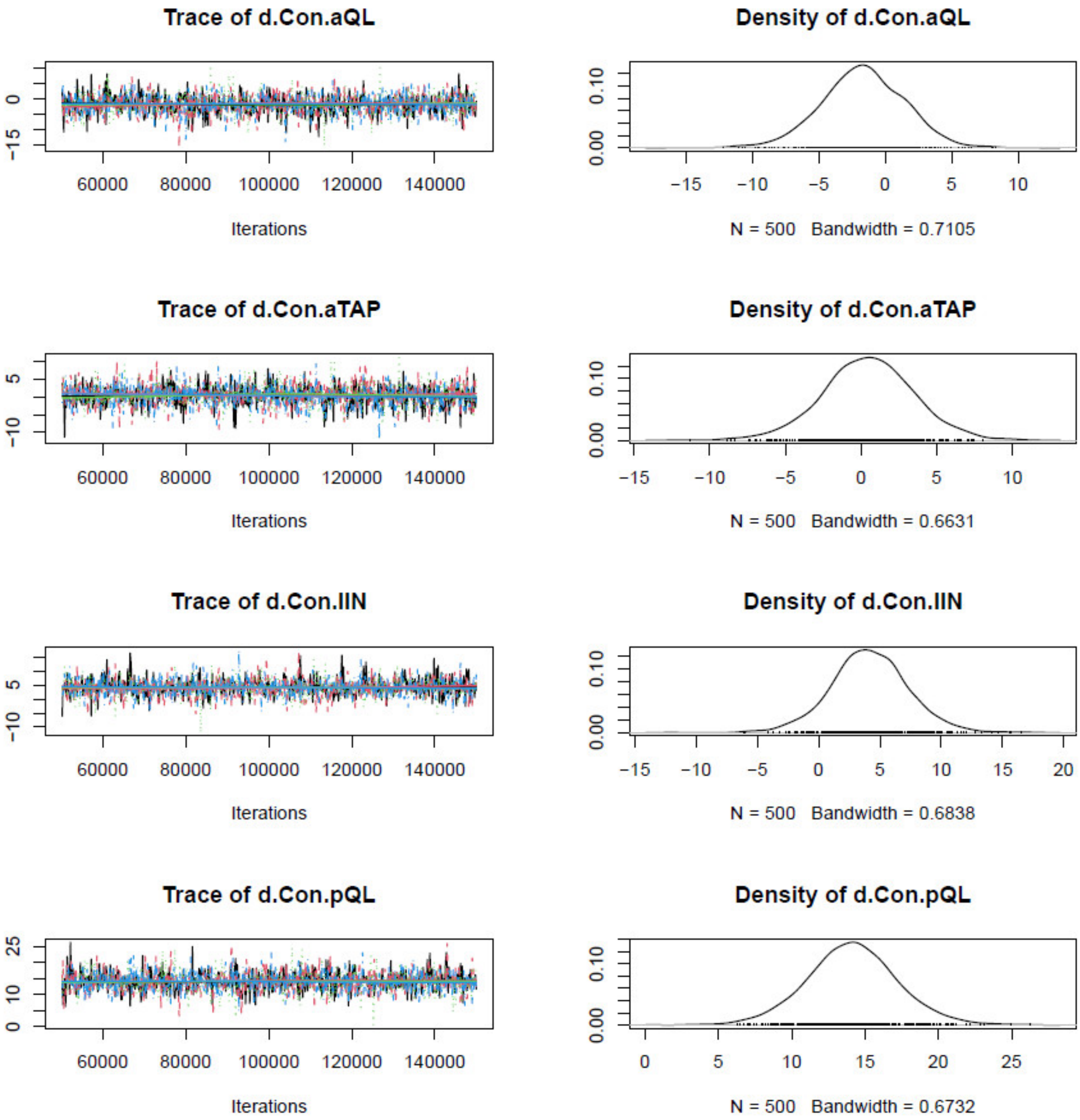

Figure S10. *Cont.*

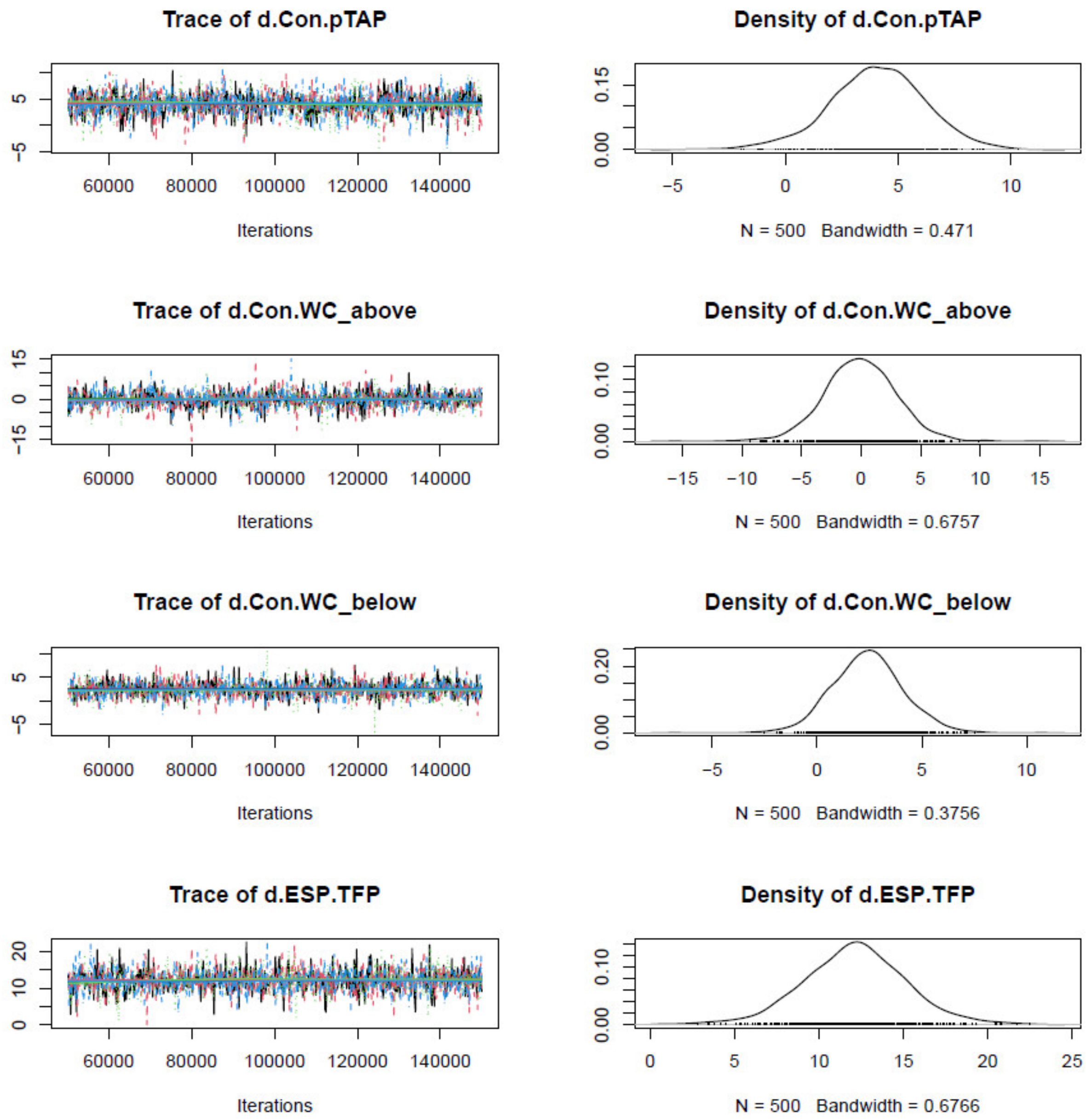

Figure S10. *Cont.*

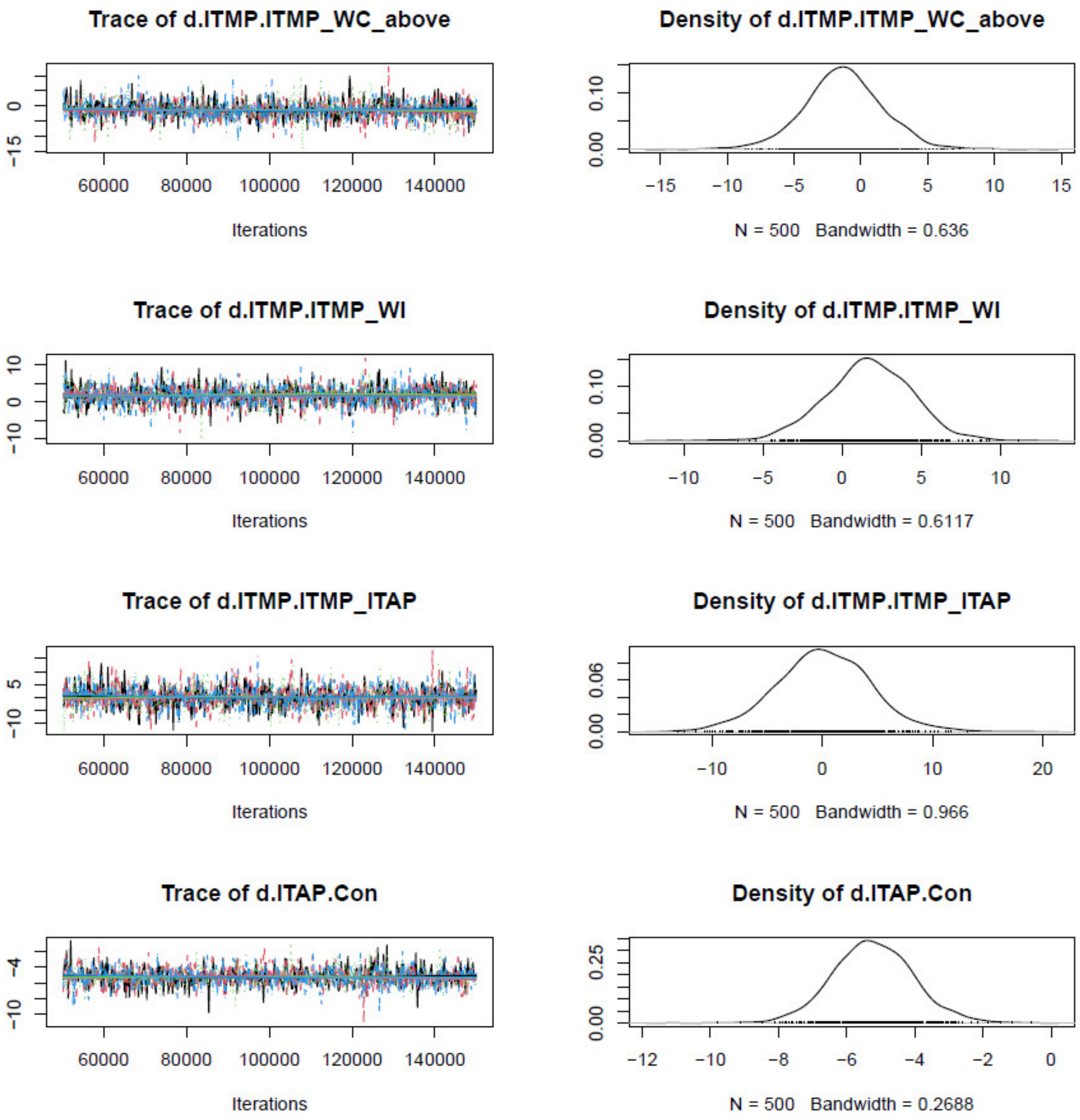

Figure S10. *Cont.*

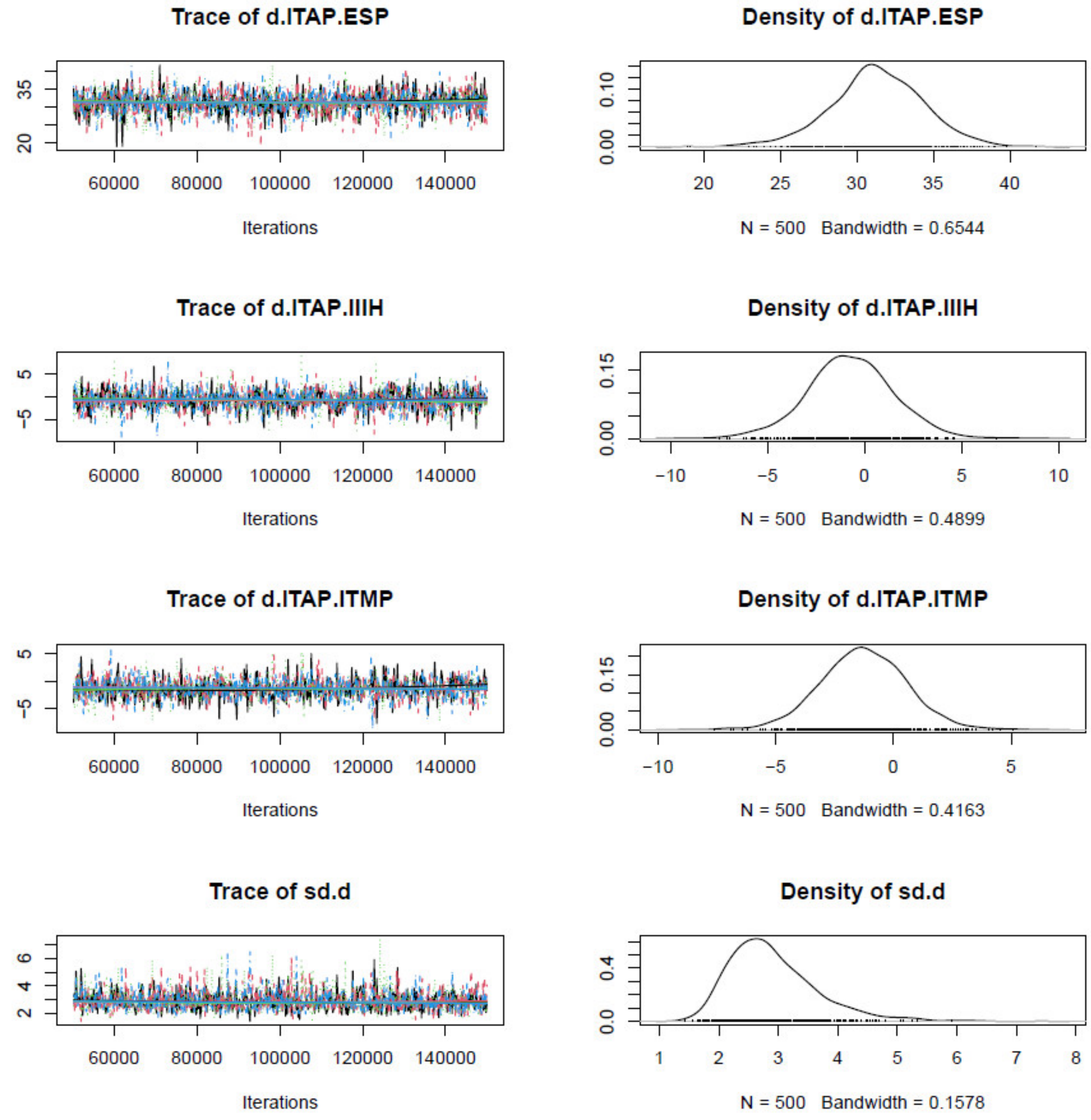

Figure S10. *Cont.*

(**C**) Gelman-Rubin-Brooks plot in fixed effect model.

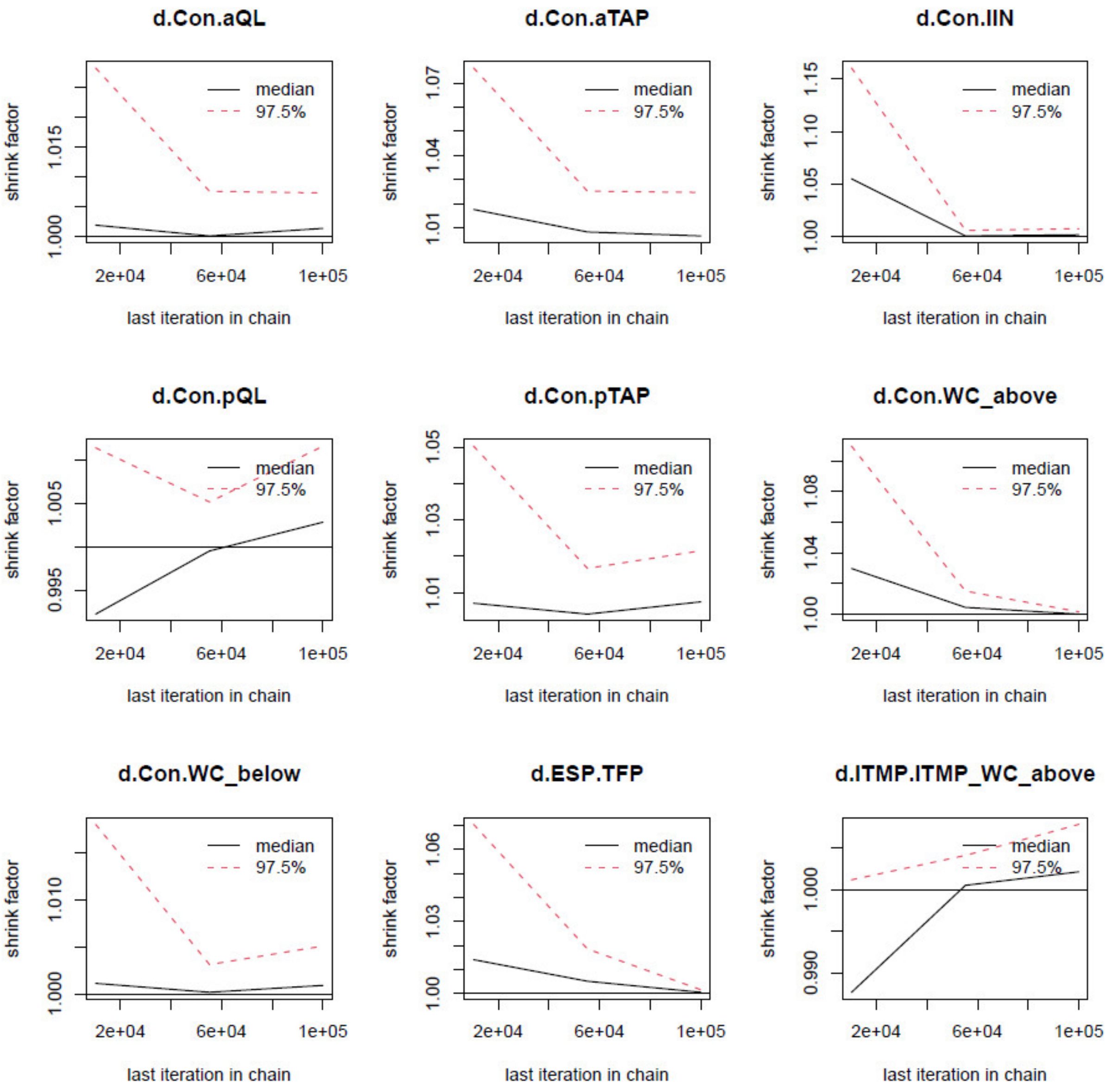

Figure S10. *Cont.*

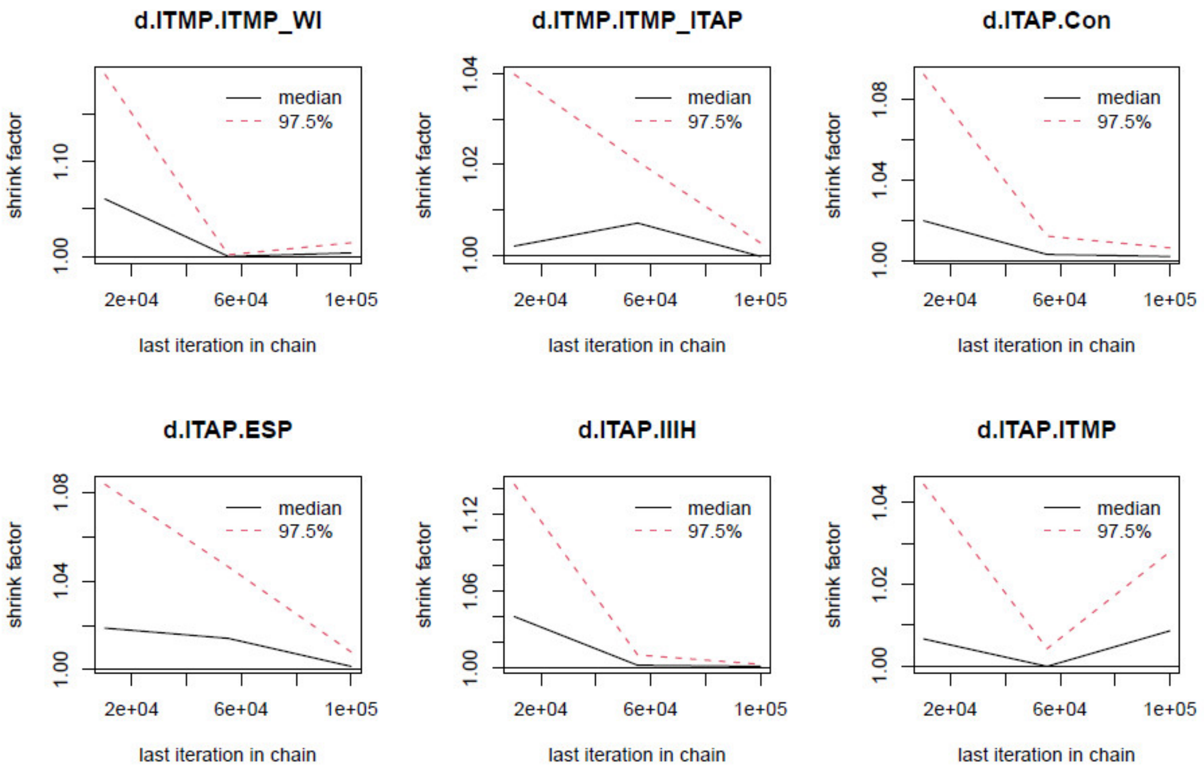

Figure S10. *Cont.*

(**D**) Gelman-Rubin-Brooks plot in random effect model.

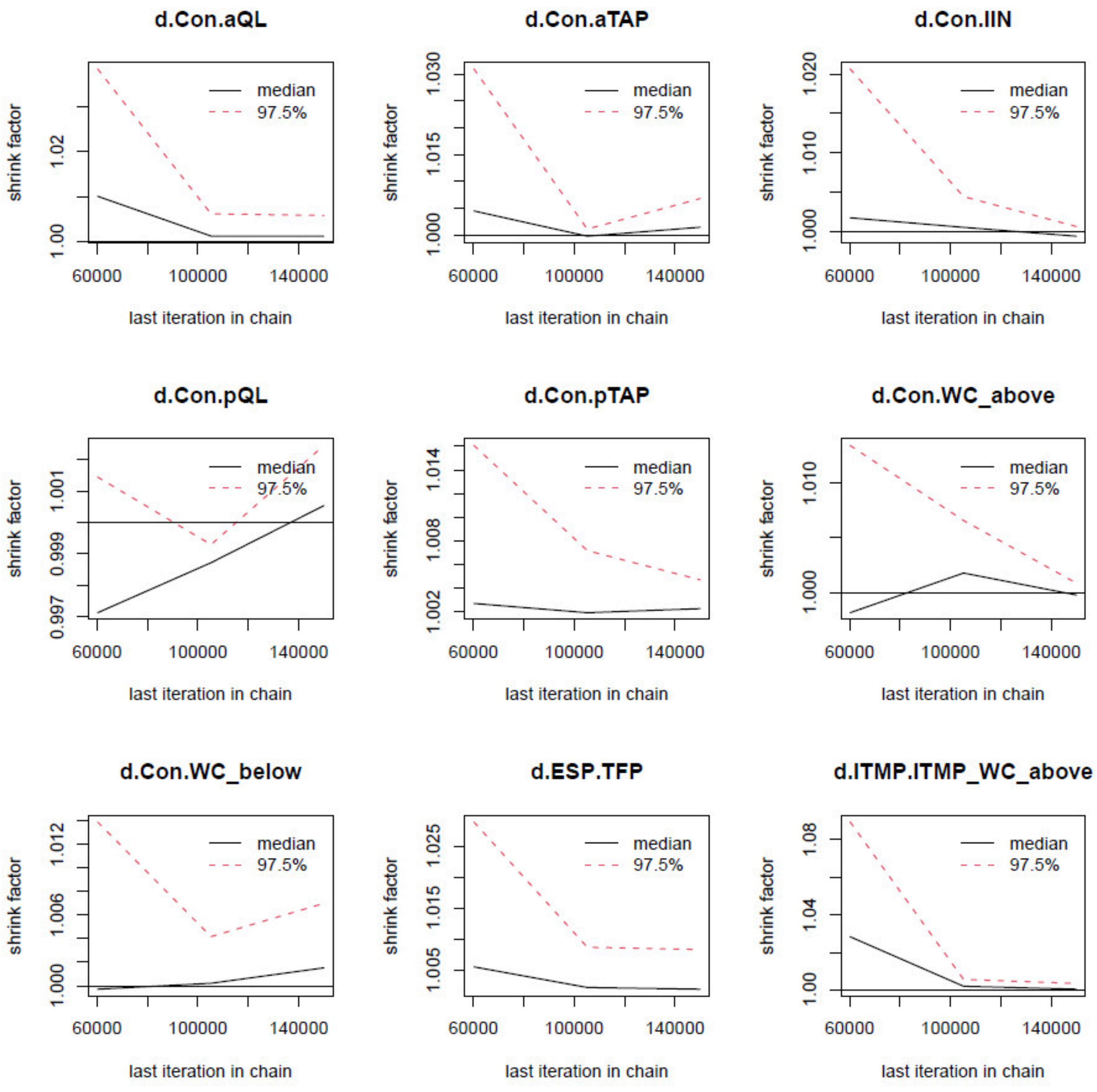

Figure S10. *Cont.*

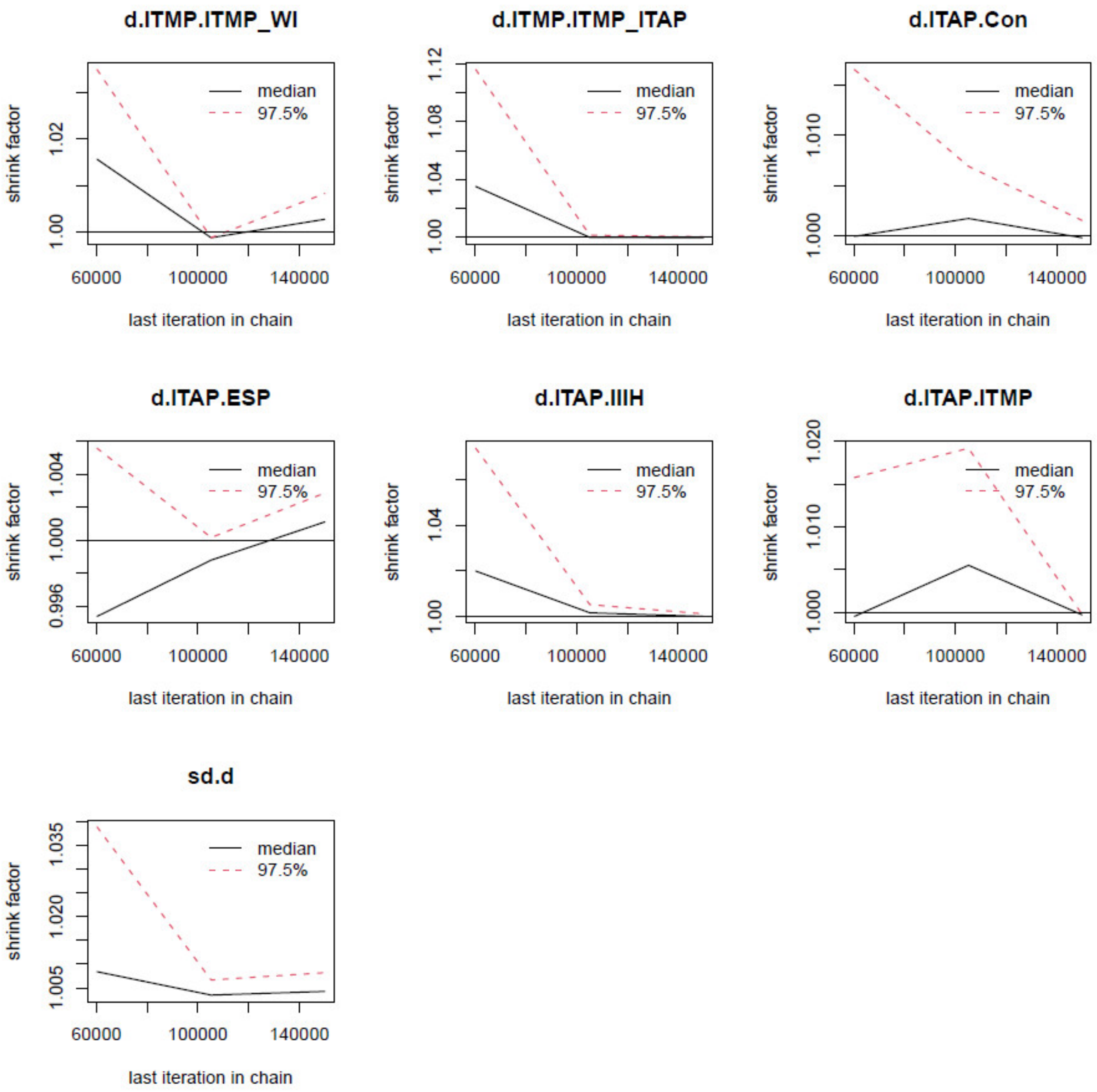

Figure S10. *Cont.*

(**E**) Forest plot comparing with control in random effect model.

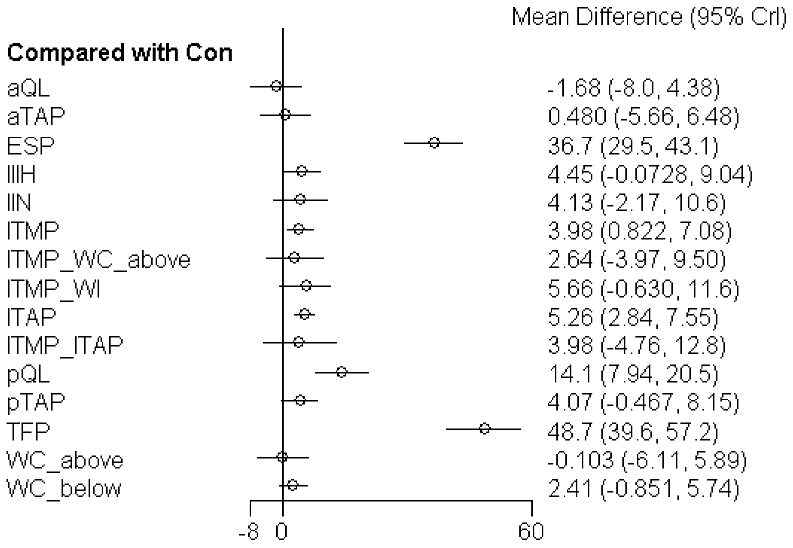

Figure S10. The time to first analgesic request. (**A**) Trace plot and density plot in fixed effect model; (**B**) Trace plot and density plot in random effect model; (**C**) Gelman-Rubin-Brooks plot in fixed effect model; (**D**) Gelman-Rubin-Brooks plot in random effect model; (**E**) Forest plot comparing with control in random effect model.

Table S1. Summary of risk of bias assessment (RoB 2).

ID	Author, Year	Randomization Process		Intended Intervention		Missing Outcome Data		Measurement of the Outcome		Selection of the Reported Result		Overall RoB
1	Aydin et al., 2020 [16]	low	random number sealed envelops	low	both blinded	low	no exclusion	low	blinded	low	predefined outcomes	low
2	Aydogmus et al., 2014 [17]	Some concerns	no statement for allocation concealment random code	Some concerns	different PNB, no information about trial context	low	no exclusion	Some concerns	no specific information	Some concerns	no information	Some concerns
3	Baaj et al., 2010 [18]	Some concerns	no specific information	low	both blinded	low	no exclusion	Some concerns	no specific information	Some concerns	no information	Some concerns
4	Bamigboye et al., 2008 [19]	low	random number sealed envelops	low	both blinded	low	no exclusion	low	blinded	Some concerns	no information	Some concerns
5	Barney et al., 2020 [20]	low	random number central allocation	low	both blinded	low	6/77 dropped, unrelated to the outcome	low	blinded	low	predefined outcomes	low
6	Belavy et al., 2009 [21]	low	block randomization table central allocation	low	both blinded	low	3/50 dropped, 1 in intervention doe to complication	low	blinded	low	predefined outcomes	low
7	Bell et al., 2002 [22]	low	computer−generated code central allocation	low	both blinded	low	11/90 dropout (6 in study and 5 in control group)	Some concerns	no specific information	Some concerns	no information	Some concerns
8	Bensghir et al., 2008 [23]	low	no statement for sequence generation envelope technique	low	both blinded	low	no exclusion	low	blinded	Some concerns	no information	Some concerns
9	Bessmertnyj et al., 2015 [24]	low	no statement for sequence generation random envelope	Some concerns	no specific information	Some concerns	no specific information	Some concerns	no block in control, may be influenced	Some concerns	no information	Some concerns
10	Blanco et al., 2015 [25]	low	random number sealed envelops	low	both blinded	low	2/50 excluded before receiving intervention	Some concerns	no specific information	low	predefined outcomes	Some concerns
11	Blanco et al., 2016 [26]	Some concerns	no statement for allocation concealment internet software	Some concerns	different PNB, no information about trial context	low	no exclusion	low	blinded	low	predefined outcomes	Some concerns
12	Bollag et al., 2012 [27]	low	computer−generated number central allocation	low	both blinded	low	9/90 excluded due to block failure	low	blinded	Some concerns	no information	Some concerns
13	Canakci et al., 2018 [28]	high	according to patient's preference	Some concerns	different procedure no information about trial context	low	4/84 excluded due to block failure	low	objective data/ self−reported	Some concerns	no information	high
14	Canovas et al., 2013 [29]	Some concerns	no statement for allocation concealment random code	Some concerns	different procedure no information about trial context	low	no exclusion	low	blinded	Some concerns	no information	Some concerns
15	Chandon et al., 2014 [30]	low	random number scratch card with block of 6	Some concerns	different PNB no information about trial context	high	10/39 and 5/41 dropped	low	blinded	low	predefined outcomes	high

16	Corsini et al., 2013 [31]	low	computerized randomization central allocation	low	both blinded	low	2/140 excluded	low	blinded	low	predefined outcomes	low
17	Costello et al., 2009 [32]	Some concerns	no statement for allocation concealment random number table	low	both blinded	low	4/100 excluded	low	blinded	Some concerns	no information	Some concerns
18	Demiraran et al., 2013 [33]	low	random number table sealed opaque envelops	low	both blinded	low	no exclusion	low	blinded	Some concerns	no information	Some concerns
19	Dereu et al., 2019 [34]	low	computerized randomization central allocation	low	both blinded	low	16/182 dropped, unrelated to true value	low	blinded	low	predefined outcomes	Some concerns
20	Ducarme et al., 2012 [35]	low	no statement for sequence generation sealed envelopes	low	both blinded	low	no exclusion	Some concerns	no specific information	Some concerns	no information	Some concerns
21	Eldaba et al., 2013 [36]	low	no statement for sequence generation central allocation	low	both blinded	low	no exclusion	low	blinded	Some concerns	no information	Some concerns
22	Eslamian et al., 2012 [37]	Some concerns	no statement for allocation concealment computer−generated list	low	both blinded	low	2/50 excluded due to surgical complications	Some concerns	no block in control, may be influenced	Some concerns	no information	Some concerns
23	Fakor et al., 2014 [38]	Some concerns	no statement for allocation concealment random block	Some concerns	no specific information	low	within pre−defined dropout range	Some concerns	no specific information	low	predefined outcomes	Some concerns
24	Fusco et al., 2016 [39]	low	computerized random block sealed envelopes	low	both blinded	low	no exclusion	low	blinded	low	predefined outcomes	low
25	Ganta et al., 1994 [40]	Some concerns	no specific information	Some concerns	different PNB no information about trial context	Some concerns	no specific statement	Some concerns	no block in control, may be influenced	Some concerns	no information	Some concerns
26	Gao et al., 2019 [41]	Some concerns	no specific information	Some concerns	no block in control group, no information about trial context	low	3/103 excluded due to loss of records	Some concerns	no block in control, may be influenced	Some concerns	no information	Some concerns
27	Givens et al., 2002 [42]	low	computer−generated randomization sealed packets	low	both blinded	Some concerns	no specific information	low	blinded	Some concerns	no information	Some concerns
28	Hansen et al., 2019 [43]	low	computer−generated blocks, sealed opque envelopes	low	both blinded	low	2/72 excluded due to surgical complications	low	blinded	low	predefined outcomes	low
29	Irwin et al., 2020 [44]	low	computer−generated number, numbered opaque envelops	low	both blinded	low	1/45 and 3/45 excluded due to protocol violation	Some concerns	no specific information	Some concerns	no information	Some concerns
30	Jadon et al., 2018 [45]	low	random number sealed	low	both blinded	low	3/70 and 2/69 (1 due to	some	no specific information	low	predefined outcomes	Some concerns

			opaque envelopes				complication) excluded	concer ns				
31	Jolly et al., 2015 [46]	low	random number list sealed envelope	some concer ns	no procedure in the control, no information about trial context	low	no exclusion	Some concer ns	no block in control, may be influenced	low	predefined outcomes	Some concerns
32	Kagwa et al., 2015 [47]	Some concer ns	no specific information	low	both blinded	some concer ns	no specific information	Some concer ns	no specific information	Some concer ns	no information	Some concerns
33	Kainu et al., 2012 [48]	Some concer ns	no statement for allocation concealment	Some concer ns	no specific information	low	6/66 excluded, unrelated reason with outcome	low	blinded	Some concer ns	no information	Some concerns
34	Kanazi et al., 2010 [49]	low	random number computer−gene rated table, sealed opaque envelopes	low	both blinded	low	within pre−defined dropout range	low	blinded	Some concer ns	no information	Some concerns
35	Kang et al., 2019 [50]	Some concer ns	random sequence no statement for allocation concealment	Some concer ns	blinding patients only no information about trial context	low	within pre−defined dropout range	Some concer ns	no specific information	low	predefined outcomes	Some concerns
36	Kessous et al., 2012 [51]	Some concer ns	random number no statement for allocation concealment	low	both blinded	low	within pre−defined dropout range	low	blinded	low	predefined outcomes	Some concerns
37	Kiran et al., 2017 [52]	low	computer−gene rated random table, closed envelopes	Some concer ns	different PNB no information about trial context	low	no exclusion	low	blinded	Some concer ns	no information	Some concerns
38	Klasen et al., 2016 [53]	Some concer ns	random table no statement for allocation concealment	Some concer ns	different PNB no information about trial context	low	within pre−defined dropout range	low	blinded	low	predefined outcomes	Some concerns
39	Krohg et al., 2018 [54]	low	blocks of 8 sealed opaque envelopes	low	both blinded	low	no exclusion	low	blinded	low	predefined outcomes	low
40	Kwikiriza et al., 2019 [55]	Some concer ns	random generator no statement for allocation concealment	low	both blinded	low	no exclusion	low	blinded	low	predefined outcomes	Some concerns
41	Lalmand et al., 2017 [56]	Some concer ns	random groups no statement for allocation concealment	low	both blinded	low	within pre−defined dropout range	low	blinded	low	predefined outcomes	Some concerns
42	Lavand'hom me et al., 2007 [57]	Some concer ns	random numbers no statement for allocation concealment	low	both blinded	low	no exclusion	low	blinded	Some concer ns	no information	Some concerns
43	Lee et al., 2013 [58]	Some concer ns	random single block no statement for allocation concealment	low	both blinded	low	1/50 excluded	low	blinded	Some concer ns	no information	Some concerns
44	Loane et al., 2012 [59]	low	computer−gene rated table, sealed envelopes	low	both blinded	low	3/69 excluded due to protocol violation	low	blinded	low	predefined outcomes	low

45	Lui et al., 2017 [60]	low	random block of 6 numbered sealed opaque envelope	Some concerns	blinded only in patient, no information about trial context	low	38/40 enrolled in one group	low	blinded	Some concerns	no information	Some concerns
46	Magnani et al., 2006 [61]	Some concerns	no specific information	Some concerns	no specific information	Some concerns	no specific information	Some concerns	no specific information	Some concerns	no information	Some concerns
47	Malawat et al., 2020 [62]	low	block randomization numbered sealed opaque envelopes	Some concerns	different PNB no information about trial context	low	early terminated, but no exclusion after intervention	low	blinded	low	predefined outcomes	Some concerns
48	Mankikar et al., 2016 [63]	low	random number table sealed envelopes	low	both blinded	low	no exclusion	low	blinded	Some concerns	no information	Some concerns
49	McKeen et al., 2014 [64]	low	random block table numbered sealed opaque envelopes	low	both blinded	low	within pre−defined dropout range	low	blinded	low	predefined outcomes	low
50	McMorrow et al., 2011 [65]	low	no statement for sequence generation central allocation	low	both blinded	low	no exclusion	low	blinded	Some concerns	no information	Some concerns
51	Mecklem et al., 1995 [66]	low	random number table central allocation	low	both blinded	Some concerns	no specific information	low	blinded	Some concerns	no information	Some concerns
52	Mieszkowski et al., 2018 [67]	Some concerns	random number table no statement for allocation concealment	Some concerns	no block in control group no information about trial context	low	2/60 excluded	Some concerns	no block in control, may be influenced	Some concerns	no information	Some concerns
53	Naghshineh et al., 2015 [68]	Some concerns	no specific information	Some concerns	no block in control group, no information about trial context	low	no exclusion	Some concerns	no block in control, may be influenced	Some concerns	no information	Some concerns
54	Niklasson et al., 2012 [69]	low	random block sealed opaque envelopes	low	both blinded	low	within pre−defined dropout range	low	blinded	low	predefined outcomes	low
55	O'Neill et al., 2012 [70]	low	computer−generated random number opaque envelopes	Some concerns	different PNB no information about trial context	low	within pre−defined dropout range	low	blinded	Some concerns	no information	Some concerns
56	Patel et al., 2017 [71]	low	random number generator sealed envelopes	low	both blinded	low	within pre−defined dropout range	low	blinded	low	predefined outcomes	low
57	Rackelboom et al., 2010 [72]	low	random number sealed opaque envelopes	low	both blinded	low	no exclusion	low	blinded	Some concerns	no information	Some concerns
58	Reinikainen et al., 2014 [73]	low	block randomization numbered sealed envelopes	low	both blinded	low	within pre−defined dropout range	low	blinded	low	predefined outcomes	low
59	Salama et al., 2020 [74]	low	random number sealed	Some concerns	no block in control group, no information	low	no exclusion	Some concerns	no specific information	low	predefined outcomes	Some concerns

			opaque envelopes		about trial context							
60	Sekhavat et al., 2011 [75]	low	random number sealed opaque envelopes	low	both blinded	low	within pre−defined dropout range	low	blinded	Some concer ns	no information	Some concerns
61	Serifsoy et al., 2020 [76]	low	no statement for sequence generation sealed envelopes	Some concer ns	no block in control group, no information about trial context	low	no exclusion	Some concer ns	no block in control, may be influenced	low	predefined outcomes	Some concerns
62	Shahin et al., 2010 [77]	low	random number sealed opaque envelopes	low	both blinded	low	within pre−defined dropout range	low	blinded	Some concer ns	no information	Some concerns
63	Singh et al., 2013 [78]	low	random number table sealed opaque envelopes	low	both blinded	low	within pre−defined dropout range	low	blinded	Some concer ns	no information	Some concerns
64	Srivastava et al., 2015 [79]	low	random number table sealed opaque envelopes	low	both blinded	low	within pre−defined dropout range	low	blinded	Some concer ns	no information	Some concerns
65	Staker et al., 2018 [80]	low	computer−generated sequence sealed envelopes	low	both blinded	low	no exclusion	low	blinded	Some concer ns	no information	Some concerns
66	Svirskiĭ et al., 2012 [81]	low	no statement for sequence generation envelope technique	Some concer ns	no block in control group no information about trial context	Some concer ns	no specific information	Some concer ns	no block in control, may be influenced	Some concer ns	no information	Some concerns
67	Pavy et al., 1994 [82]	Some concer ns	randomization table no statement for allocation concealment	low	both blinded	low	no exclusion	low	blinded	Some concer ns	no information	Some concerns
68	Tamura et al., 2019 [83]	Some concer ns	computer−generated, no statement for allocation concealment	low	both blinded	low	30/176 excluded, even among the group, unrelated to true value	low	blinded	low	predefined outcomes	Some concerns
69	Tan et al., 2012 [84]	low	random number table sealed opaque envelopes	Some concer ns	no block in control group, no information about trial context	low	no exclusion	Some concer ns	no block in control, may be influenced	Some concer ns	no information	Some concerns
70	Tawfik et al., 2017 [85]	low	random number table sealed opaque envelopes	Some concer ns	different PNB no information about trial context	low	within pre−defined dropout range	low	blinded	low	predefined outcomes	Some concerns
71	Telnes et al., 2015 [86]	low	no statement for sequence generation central allocation	Some concer ns	different PNB no information about trial context	low	within pre−defined dropout range	low	blinded	low	predefined outcomes	Some concerns
72	Triyasunant et al., 2015 [87]	low	block randomization sealed envelopes	Some concer ns	no block in control group, no information about trial context	low	no exclusion	low	blinded	low	predefined outcomes	Some concerns
73	Trotter et al., 1991 [88]	low	no statement for sequence	low	both blinded	some	no specific information	low	blinded	some	no information	some concerns

			generation central allocation			concerns				concerns		
74	Vallejo et al., 2012 [89]	Some concerns	candom table no statement for allocation concealment	low	both blinded	low	no exclusion	Some concerns	no specific information	Some concerns	no information	Some concerns
75	Wagner-Kovacec et al., 2018 [90]	low	no statement for sequence generation sealed envelopes	low	both blinded	low	no exclusion	low	blinded	low	predefined outcomes	low
76	Wolfson et al., 2012 [91]	low	random single block sequential sealed envelopes	low	both blinded	low	no exclusion	low	blinded	Some concerns	no information	Some concerns

Table S2. Network diagnostic for Bayesian analysis.

Outcome	Effect	Dbar	pD	DIC	PSRF	I^2(%)
Pain at rest 6 h after surgery	Fixed	4804.71	108.52	4913.23	1.07	97%
	Random	172.22	125.58	297.81	1.01	26%
Postoperative cumulative 24 h morphine equivalent consumption (mg)	Fixed	1624.74	463.05	2087.79	1.7	94%
	Random	95.22	87.89	183.11	1.01	3%
Pain at rest 24 h after surgery	Fixed	1058.75	180.82	1239.58	1.15	88%
	Random	131.65	124.09	255.74	1.02	3%
Dynamic pain at 6 h after surgery	Fixed	2380.54	83.94	2463.49	1.1	97%
	Random	83.11	82.75	165.86	1.02	1%
Dynamic pain 24 h after surgery	Fixed	1044.79	82.13	1126.93	1.02	91%
	Random	96.23	94.54	190.77	1.01	0.1%
The time to first analgesic request (hours)	Fixed	778.62	92.80	871.41	1.02	94%
	Random	50.65	49.56	100.21	1.01	1%

Dbar: posterior mean of the deviance, pD: adequate number of parameters, DIC: deviance information criterion, sum of Dbar and PD, PRSF: Potential Scale Reduction Factor.

Table S3. Network league tables for all the postcesarean analgesic strategies regarding. (**A**) pain at 6 h after surgery and (**B**) postoperative cumulative 24 h morphine equivalent consumption (mg).

(**A**)

**ITMP+II–aTAP**											
−4.60 (−10.62,1.42)	ITMP+IIIH										
−6.54 (−11.67,−1.41)	−1.94 (−5.77,1.89)	ITMP+lTAP									
−6.10 (−12.11,−0.09)	−1.50 (−6.45,3.45)	0.44 (−3.36,4.24)	ITMP+RS								
−6.64 (−12.79,−0.48)	−2.04 (−7.16,3.08)	−0.10 (−4.04,3.85)	−0.54 (−5.64,4.56)	ESP							
−6.76 (−12.75,−0.77)	−2.16 (−7.09,2.77)	−0.22 (−4.00,3.56)	−0.66 (−5.57,4.25)	−0.12 (−5.21,4.96)	ITMP+IPLA						
−7.36 (−13.11,−1.61)	−2.76 (−7.39,1.86)	−0.82 (−4.15,2.50)	−1.26 (−5.87,3.34)	−0.73 (−5.34,3.88)	−0.60 (−5.19,3.99)	ITMP+pQL					
−7.60 (−12.49,−2.70)	−3.00 (−6.51,0.51)	−1.06 (−2.59,0.46)	−1.50 (−4.98,1.98)	−0.96 (−4.69,2.76)	−0.84 (−4.30,2.62)	−0.24 (−3.25,2.78)	ITMP				
−7.52 (−13.51,−1.52)	−2.92 (−7.84,2.01)	−0.98 (−4.68,2.72)	−1.42 (−6.32,3.49)	−0.88 (−5.70,3.94)	−0.76 (−5.64,4.13)	−0.15 (−4.42,4.12)	0.08 (−3.37,3.54)	apQL			
−7.62 (−13.27,−1.97)	−3.02 (−7.52,1.48)	−1.08 (−4.19,2.03)	−1.52 (−6.00,2.96)	−0.98 (−5.34,3.38)	−0.86 (−5.32,3.60)	−0.26 (−4.06,3.55)	−0.02 (−2.84,2.80)	−0.10 (−3.37,3.16)	EDMP		
−7.83 (−13.11,−2.55)	−3.23 (−7.26,0.79)	−1.29 (−3.65,1.07)	−1.73 (−5.73,2.27)	−1.20 (−5.01,2.62)	−1.07 (−5.05,2.91)	−0.47 (−3.76,2.83)	−0.23 (−2.20,1.74)	−0.32 (−3.74,3.11)	−0.21 (−2.77,2.34)	WC_below	
−7.66 (−13.91,−1.42)	−3.07 (−8.29,2.16)	−1.13 (−5.21,2.96)	−1.57 (−6.77,3.64)	−1.03 (−6.10,4.05)	−0.91 (−6.10,4.29)	−0.30 (−4.99,4.38)	−0.07 (−3.94,3.81)	−0.15 (−5.03,4.73)	−0.05 (−4.48,4.39)	0.17 (−3.76,4.09)	slTAP

−8.14 (−13.22,−3.05)	−3.54 (−7.31,0.23)	−1.60 (−3.49,0.29)	−2.04 (−5.78,1.71)	−1.50 (−4.96,1.96)	−1.38 (−5.10,2.35)	−0.77 (−3.82,2.27)	−0.54 (−1.91,0.84)	−0.62 (−3.97,2.73)	−0.52 (−3.17,2.13)	−0.30 (−1.90,1.29)	−0.47 (−4.18,3.24)	lTAP												
−8.48 (−13.96,−3.00)	−3.88 (−8.17,0.41)	−1.94 (−4.73,0.85)	−2.38 (−6.65,1.89)	−1.84 (−5.97,2.28)	−1.72 (−5.97,2.53)	−1.12 (−4.72,2.48)	−0.88 (−3.35,1.59)	−0.96 (−4.81,2.88)	−0.86 (−4.13,2.41)	−0.65 (−3.19,1.89)	−0.82 (−4.98,3.35)	−0.34 (−2.58,1.90)	WC_above											
−8.58 (−13.81,−3.35)	−3.98 (−7.94,−0.02)	−2.04 (−4.32,0.24)	−2.48 (−6.42,1.46)	−1.94 (−5.81,1.93)	−1.82 (−5.73,2.10)	−1.22 (−4.26,1.83)	−0.98 (−2.81,0.85)	−1.06 (−4.24,2.11)	−0.96 (−3.57,1.65)	−0.75 (−2.81,1.32)	−0.91 (−4.86,3.03)	−0.44 (−2.17,1.28)	−0.10 (−2.65,2.45)	pQL										
−8.66 (−13.86,−3.47)	−4.06 (−7.98,−0.15)	−2.13 (−4.29,0.04)	−2.57 (−6.45,1.32)	−2.03 (−5.74,1.68)	−1.91 (−5.77,1.96)	−1.30 (−4.45,1.85)	−1.07 (−2.79,0.66)	−1.15 (−4.58,2.28)	−1.05 (−3.82,1.73)	−0.83 (−2.68,1.01)	−1.00 (−4.47,2.47)	−0.53 (−1.85,0.79)	−0.18 (−2.50,2.13)	−0.09 (−1.96,1.79)	WI									
−8.85 (−14.33,−3.38)	−4.25 (−8.54,0.03)	−2.31 (−5.20,0.58)	−2.75 (−7.01,1.51)	−2.22 (−6.68,2.25)	−2.09 (−6.34,2.15)	−1.49 (−5.38,2.40)	−1.25 (−3.71,1.20)	−1.34 (−5.57,2.90)	−1.23 (−4.97,2.51)	−1.02 (−4.17,2.13)	−1.19 (−5.77,3.40)	−0.72 (−3.53,2.10)	−0.37 (−3.86,3.11)	−0.27 (−3.34,2.79)	−0.19 (−3.19,2.82)	pTAP								
−8.84 (−14.45,−3.22)	−4.24 (−8.70,0.22)	−2.30 (−5.35,0.75)	−2.74 (−7.18,1.70)	−2.20 (−6.53,2.13)	−2.08 (−6.50,2.34)	−1.47 (−5.22,2.28)	−1.24 (−3.99,1.52)	−1.32 (−4.57,1.93)	−1.22 (−4.09,1.66)	−1.01 (−3.76,1.75)	−1.17 (−5.56,3.21)	−0.70 (−3.30,1.89)	−0.36 (−3.55,2.84)	−0.26 (−2.80,2.29)	−0.17 (−2.86,2.52)	0.02 (−3.67,3.70)	aQL							
−8.86 (−14.31,−3.40)	−4.26 (−8.52,0.00)	−2.32 (−5.06,0.42)	−2.76 (−6.99,1.48)	−2.22 (−6.28,1.84)	−2.10 (−6.31,2.12)	−1.49 (−5.08,2.09)	−1.26 (−3.67,1.16)	−1.34 (−5.18,2.49)	−1.24 (−4.49,2.02)	−1.03 (−3.53,1.48)	−1.19 (−5.35,2.97)	−0.72 (−2.83,1.39)	−0.38 (−3.26,2.51)	−0.28 (−2.82,2.26)	−0.19 (−2.49,2.10)	−0.00 (−3.45,3.44)	−0.02 (−3.21,3.17)	IIIH						
−8.97 (−14.97,−2.97)	−4.37 (−9.30,0.57)	−2.43 (−6.13,1.27)	−2.87 (−7.79,2.05)	−2.33 (−7.11,2.44)	−2.21 (−7.11,2.69)	−1.61 (−5.96,2.75)	−1.37 (−4.84,2.10)	−1.45 (−6.01,3.11)	−1.35 (−5.44,2.74)	−1.14 (−4.67,2.39)	−1.30 (−6.14,3.53)	−0.83 (−4.12,2.45)	−0.49 (−4.28,3.30)	−0.39 (−3.93,3.15)	−0.30 (−3.68,3.07)	−0.12 (−4.37,4.13)	−0.13 (−4.16,3.90)	−0.11 (−3.30,3.08)	aTAP					
−9.19 (−14.81,−3.57)	−4.59 (−9.06,−0.12)	−2.65 (−5.70,0.40)	−3.09 (−7.54,1.36)	−2.55 (−6.85,1.75)	−2.43 (−6.86,2.00)	−1.83 (−5.63,1.98)	−1.59 (−4.35,1.17)	−1.67 (−5.71,2.37)	−1.57 (−5.07,1.93)	−1.36 (−4.19,1.47)	−1.52 (−5.80,2.75)	−1.05 (−3.60,1.49)	−0.71 (−3.85,2.43)	−0.61 (−3.45,2.23)	−0.52 (−3.03,1.98)	−0.34 (−4.03,3.36)	−0.35 (−3.78,3.07)	−0.33 (−3.47,2.81)	−0.22 (−4.21,3.77)	IIN				
−9.78 (−15.43,−4.13)	−5.18 (−9.69,−0.67)	−3.24 (−6.35,−0.13)	−3.68 (−8.16,0.80)	−3.14 (−7.49,1.20)	−3.02 (−7.49,1.45)	−2.42 (−6.27,1.43)	−2.18 (−5.00,0.64)	−2.26 (−6.34,1.81)	−2.16 (−5.71,1.38)	−1.95 (−4.84,0.94)	−2.11 (−6.50,2.27)	−1.64 (−4.27,0.98)	−1.30 (−4.48,1.89)	−1.20 (−4.10,1.69)	−1.11 (−3.80,1.57)	−0.93 (−4.67,2.82)	−0.94 (−4.42,2.53)	−0.92 (−4.11,2.27)	−0.81 (−4.84,3.22)	−0.59 (−4.01,2.83)	TFP			
−10.10 (−16.11,−4.09)	−5.50 (−10.45,−0.55)	−3.56 (−7.37,0.25)	−4.00 (−7.48,−0.52)	−3.46 (−8.57,1.65)	−3.34 (−8.26,1.58)	−2.74 (−7.35,1.88)	−2.50 (−6.00,1.00)	−2.58 (−7.50,2.33)	−2.48 (−6.97,2.01)	−2.27 (−6.28,1.74)	−2.43 (−7.65,2.78)	−1.96 (−5.72,1.79)	−1.62 (−5.90,2.66)	−1.52 (−5.47,2.43)	−1.43 (−5.33,2.46)	−1.25 (−5.52,3.02)	−1.26 (−5.71,3.19)	−1.24 (−5.49,3.00)	−1.13 (−6.06,3.79)	−0.91 (−5.37,3.55)	−0.32 (−4.81,4.17)	RS		
−9.78 (−14.87,−4.69)	−5.18 (−8.96,−1.40)	−3.24 (−5.15,−1.33)	−3.68 (−7.44,0.08)	−3.14 (−6.73,0.45)	−3.02 (−6.75,0.71)	−2.42 (−5.39,0.55)	−2.18 (−3.59,−0.77)	−2.26 (−5.52,1.00)	−2.16 (−4.72,0.40)	−1.95 (−3.47,−0.42)	−2.11 (−5.75,1.52)	−1.64 (−2.58,−0.71)	−1.30 (−3.34,0.74)	−1.20 (−2.74,0.34)	−1.11 (−2.21,−0.02)	−0.93 (−3.76,1.90)	−0.94 (−3.40,1.52)	−0.92 (−2.97,1.12)	−0.81 (−4.01,2.39)	−0.59 (−2.98,1.80)	−0.00 (−2.45,2.45)	0.32 (−3.45,4.09)	Con	
−10.25 (−15.91,−4.59)	−5.65 (−10.17,−1.13)	−3.71 (−6.84,−0.58)	−4.15 (−8.65,0.35)	−3.61 (−7.97,0.75)	−3.49 (−7.97,0.99)	−2.89 (−6.75,0.98)	−2.65 (−5.50,0.20)	−2.73 (−6.83,1.36)	−2.63 (−6.19,0.93)	−2.42 (−5.33,0.49)	−2.59 (−6.98,1.81)	−2.11 (−4.76,0.53)	−1.77 (−4.98,1.43)	−1.67 (−4.59,1.25)	−1.59 (−4.29,1.12)	−1.40 (−5.16,2.36)	−1.41 (−4.90,2.08)	−1.39 (−4.60,1.82)	−1.28 (−5.33,2.76)	−1.06 (−4.50,2.38)	−0.47 (−3.95,3.01)	−0.15 (−4.66,4.36)	−0.47 (−2.95,2.01)	lQL

(B)

ITMP+II−aTAP												
−6.20 (−28.30,15.91)	lQL											
−7.37 (−28.81,14.06)	−1.18 (−22.71,20.36)	ITMP+RS										
−7.97 (−29.74,13.81)	−1.77 (−23.64,20.10)	−0.59 (−21.80,20.61)	ITMP+WI									
−8.72 (−30.15,12.72)	−2.52 (−24.05,19.01)	−1.34 (−22.19,19.51)	−0.75 (−21.95,20.45)	ITMP+IPLA								
−8.26 (−34.93,18.40)	−2.07 (−27.28,23.14)	−0.89 (−27.09,25.31)	−0.30 (−26.77,26.18)	0.45 (−25.74,26.65)	IIIH							
−10.71 (−26.27,4.85)	−4.51 (−20.21,11.18)	−3.34 (−18.08,11.41)	−2.74 (−17.98,12.49)	−1.99 (−16.73,12.74)	−2.45 (−24.10,19.21)	ITMP						
−12.00 (−29.17,5.17)	−5.81 (−19.81,8.20)	−4.63 (−21.06,11.81)	−4.03 (−20.91,12.84)	−3.28 (−19.71,13.14)	−3.74 (−24.85,17.37)	−1.29 (−8.55,5.97)	lTAP					
−11.62 (−30.87,7.63)	−5.42 (−24.79,13.94)	−4.25 (−22.85,14.35)	−3.65 (−22.64,15.34)	−2.90 (−21.50,15.69)	−3.36 (−27.80,21.08)	−0.91 (−12.25,10.43)	0.38 (−13.08,13.84)	ITMP+IIIH				
−13.19 (−37.41,11.03)	−7.00 (−29.68,15.68)	−5.82 (−29.52,17.89)	−5.22 (−29.24,18.79)	−4.48 (−28.18,19.22)	−4.93 (−31.51,21.65)	−2.48 (−21.04,16.08)	−1.19 (−19.14,16.76)	−1.57 (−23.32,20.18)	EDMP			
−12.82 (−35.73,10.09)	−6.62 (−29.63,16.38)	−5.44 (−27.81,16.92)	−4.85 (−27.54,17.84)	−4.10 (−26.46,18.26)	−4.55 (−31.97,22.86)	−2.11 (−18.92,14.71)	−0.82 (−19.13,17.50)	−1.20 (−21.48,19.09)	0.37 (−24.67,25.42)	ITMP+lTAP		
−14.59 (−38.81,9.63)	−8.40 (−31.08,14.29)	−7.22 (−30.93,16.49)	−6.62 (−30.64,17.39)	−5.88 (−29.58,17.83)	−6.33 (−32.91,20.25)	−3.88 (−22.45,14.68)	−2.59 (−20.55,15.37)	−2.97 (−24.72,18.78)	−1.40 (−16.16,13.36)	−1.77 (−26.82,23.28)	apQL	
−15.47 (−38.70,7.75)	−9.28 (−30.93,12.38)	−8.10 (−30.78,14.59)	−7.50 (−30.51,15.50)	−6.76 (−29.44,15.93)	−7.21 (−32.89,18.47)	−4.76 (−22.00,12.48)	−3.47 (−20.07,13.13)	−3.85 (−24.48,16.78)	−2.28 (−16.72,12.16)	−2.65 (−26.74,21.43)	−0.88 (−15.32,13.56)	aQL

−15.51 (−35.78,4.76)	−9.31 (−27.64,9.01)	−8.14 (−27.79,11.51)	−7.54 (−27.56,12.47)	−6.79 (−26.44,12.85)	−7.25 (−30.27,15.77)	−4.80 (−17.79,8.19)	−3.51 (−15.58,8.56)	−3.89 (−21.13,13.35)	−2.32 (−22.46,17.82)	−2.69 (−23.94,18.55)	−0.92 (−21.06,19.22)	−0.04 (−18.97,18.89)	TFP							
−15.41 (−32.88,2.05)	−9.22 (−24.52,6.09)	−8.04 (−24.78,8.71)	−7.45 (−24.62,9.73)	−6.70 (−23.43,10.04)	−7.15 (−28.23,13.93)	−4.70 (−12.64,3.24)	−3.41 (−9.92,3.09)	−3.79 (−17.63,10.05)	−2.22 (−20.12,15.68)	−2.59 (−21.19,16.00)	−0.82 (−18.72,17.08)	0.06 (−16.47,16.59)	0.10 (−11.91,12.11)	WI						
−16.84 (−35.59,1.91)	−10.64 (−29.51,8.22)	−9.47 (−27.54,8.61)	−8.87 (−27.35,9.61)	−8.12 (−26.20,9.95)	−8.58 (−32.62,15.47)	−6.13 (−16.59,4.33)	−4.84 (−17.57,7.89)	−5.22 (−20.64,10.21)	−3.65 (−24.95,17.66)	−4.02 (−23.83,15.78)	−2.25 (−23.56,19.06)	−1.37 (−21.53,18.80)	−1.33 (−18.00,15.34)	−1.43 (−14.56,11.70)	pTAP					
−17.37 (−38.81,4.07)	−11.18 (−32.72,10.36)	−10.00 (−24.75,4.75)	−9.41 (−30.61,11.80)	−8.66 (−29.51,12.19)	−9.11 (−35.31,17.09)	−6.66 (−21.41,8.09)	−5.37 (−21.82,11.07)	−5.75 (−24.36,12.85)	−4.18 (−27.89,19.53)	−4.56 (−26.93,17.81)	−2.78 (−26.49,20.93)	−1.90 (−24.59,20.79)	−1.86 (−21.52,17.79)	−1.96 (−18.71,14.79)	−0.53 (−18.62,17.55)	RS				
−18.73 (−36.26,−1.19)	−12.53 (−27.93,2.87)	−11.35 (−28.17,5.47)	−10.76 (−28.01,6.49)	−10.01 (−26.82,6.80)	−10.46 (−31.72,10.80)	−8.02 (−16.10,0.07)	−6.73 (−13.47,0.02)	−7.11 (−21.03,6.82)	−5.54 (−23.68,12.61)	−5.91 (−24.57,12.75)	−4.14 (−22.29,14.02)	−3.25 (−20.07,13.56)	−3.22 (−15.54,9.11)	−3.31 (−10.96,4.33)	−1.89 (−15.11,11.33)	−1.35 (−18.18,15.47)	WC_below			
−20.11 (−40.05,−0.17)	−13.91 (−31.91,4.08)	−12.74 (−32.05,6.57)	−12.14 (−31.83,7.54)	−11.39 (−30.70,7.91)	−11.85 (−34.59,10.89)	−9.40 (−21.87,3.07)	−8.11 (−19.64,3.42)	−8.49 (−25.34,8.36)	−6.92 (−21.36,7.52)	−7.29 (−28.23,13.64)	−5.52 (−19.96,8.92)	−4.64 (−18.08,8.80)	−4.60 (−19.32,10.12)	−4.70 (−16.15,6.76)	−3.27 (−19.55,13.00)	−2.74 (−22.05,16.58)	−1.38 (−13.19,10.42)	pQL		
−21.50 (−41.89,−1.11)	−15.30 (−33.75,3.14)	−14.12 (−33.90,5.65)	−13.53 (−33.67,6.61)	−12.78 (−32.55,6.99)	−13.24 (−36.36,9.89)	−10.79 (−23.97,2.39)	−9.50 (−21.76,2.77)	−9.88 (−27.26,7.51)	−8.31 (−28.58,11.97)	−8.68 (−30.05,12.69)	−6.91 (−27.19,13.38)	−6.03 (−25.11,13.06)	−5.99 (−21.31,9.33)	−6.09 (−18.30,6.13)	−4.66 (−21.48,12.17)	−4.12 (−23.91,15.66)	−2.77 (−15.29,9.75)	−1.39 (−16.28,13.51)	WC_above	
−27.26 (−44.47,−10.06)	−21.07 (−35.91,−6.22)	−19.89 (−36.36,−3.42)	−19.30 (−36.21,−2.39)	−18.55 (−35.01,−2.08)	−19.00 (−39.37,1.37)	−16.55 (−23.89,−9.21)	−15.26 (−20.80,−9.72)	−15.64 (−29.15,−2.14)	−14.07 (−31.14,3.00)	−14.45 (−32.79,3.90)	−12.67 (−29.75,4.40)	−11.79 (−27.43,3.85)	−11.75 (−22.47,−1.03)	−11.85 (−17.28,−6.42)	−10.42 (−23.20,2.35)	−9.89 (−26.37,6.59)	−8.54 (−14.62,−2.45)	−7.15 (−17.25,2.95)	−5.76 (−16.71,5.18)	Con

Estimates are presented as mean difference (95% confidence interval). Abbreviations: ITMP = intrathecal morphine; EDMP = epidural morphine; ESP = erector spinae plane block; TFP = transverse fascial plane block; AQL = anterior quadratus lumborum block; pQL = posterior QL; apQL = combined anterior and posterior quadratus lumborum block; lQL = lateral QL block; lTAP = lateral TAP block; pTAP = posterior TAP block; slTAP=combined subcostal and lateral TAP block; II−aTAP = combined ilioinguinal and anterior TAP block; IIIH = ilioinguinal−iliohypogastric block; IIN = ilioinguinal nerve block; RS=rectus sheath block; IPLA = intraperitoneal local anesthetics instillation; WC_below = wound continuous infusion below the fascia; WC_above = wound continuous infusion above the fascia; WI = wound infiltration; Con = control.
