# Supplementary material for: Postoperative Analgesic Effectiveness of Peripheral Nerve Blocks in Cesarean Delivery: A Systematic Review and Network Meta-Analysis
Source: J Pers Med. 2022 Apr 14;12(4):634. doi: 10.3390/jpm12040634 (PMC9033028; doi:10.3390/jpm12040634)
Supplement: Supplementary file 1 [file jpm-12-00634-s001.zip › jpm-1607529 - Supplementary Methods - Conversion.pdf]

Journal of *Personalized Medicine*

Supplementary Methods

S1. Materials and Methods

S1.1. Eligibility Criteria

S1.1.1. Inclusion Criteria

We included full reports of randomized controlled trials(RCTs) that investigated the postoperative analgesic effects of all kinds of peripheral nerve block (PNB) methods and intrathecal or epidural morphine in cesarean delivery.

The PICO-SD information was comprised as follows:

(1) Patients (P): all parturients receiving cesarean section performed under spinal, combined spinal-epidural (CSE), or general anesthesia.

(2) Intervention (I): PNB with or without intrathecal (as an adjuvant analgesic for spinal anesthesia) or epidural morphine (as an adjuvant analgesic for combined spinal-epidural (CSE) anesthesia) to improve post-cesarean delivery pain, which might be bolus infiltration or continuous infusion.

(3) Comparison (C): other PNB with or without intrathecal morphine (ITMP), other approaches of the same block technique with or without ITMP, active control (ITMP), or non-active control (sham block or saline block).

(4) Outcome measurements (O): The two co-primary outcomes of this network meta-analysis (NMA) were designated as (1) pain at rest 6 h after surgery and (2) postoperative cumulative 24 h morphine equivalent consumption. Secondary outcomes were as follows: (1) the time to first analgesic request (hours), (2) pain at rest 24 h after surgery, (3) dynamic pain 6 h after surgery, (4) dynamic pain 24 h after surgery.

(5) Study design (SD): full reports of RCTs.

We did include RCTs that were performed under not only spinal anesthesia but also CSE and general anesthesia. This is because our study is not for a comparison of intraoperative hemodynamic parameters but rather a comparison of postoperative analgesic effects of all kinds of analgesic strategies. Since the analgesic effect of spinal anesthesia performed with short-acting opioids gradually disappears around 6 h after the surgery [1], it is reasonable to compare these outcomes including PNBs in general anesthesia. Additionally, we designed the study to analyze the use of morphine, a long-acting opioid, both for spinal and epidural anesthesia as separate comparison factors.

On the other hand, the reason we chose pain severity at 6 h after surgery as a valuable primary outcome was to compare the worst pain after cesarean section. Kintu et al. reported that the worst pain after a cesarean section was experienced at 6 h because of spinal anesthesia wearing off without further analgesia around that time [1]. Second, 24 h interval was chosen in the three outcomes because the analgesic effect of PNB lasts for 12 to 24 hours, generally [2].

S1.1.2. Exclusion Criteria

Ineligible studies contained the following features:

(1) Matters relating to P: laparotomy surgeries with a lower abdominal transverse incision for other than cesarean sections.

(2) Matters relating to I or C: (1) studies that compared the postoperative analgesic effects with per os (PO) or intravenous medication, (2) studies that could not identify the anatomical approach even through full-text review, and (3) studies on PNB with an alternative drug (e.g., dexmedetomidine, ketorolac, etc.), not local anesthetics.

(3) Matters relating to O: (1) studies that compared analgesic effects for labor pain or surgical condition only and (2) studies that failed to report the outcomes of interest.

(4) Matters relating to SD: (1) different kinds of review articles, case reports, or case series, letters to the editor, commentaries, proceedings, laboratory sciences studies, and all other non-relevant studies, (2) dose–response studies that neither have a control group nor use other strategies, (3) dose-finding studies, which used up-and-down sequential method.

There were neither language limitations nor date restrictions in this study. Non-English articles were analyzed using an online translator or consulting with an expert in the language in which the article was written.

S1.2. Search Strategy and Study Selection

S1.2.1. Search Strategy

We searched MEDLINE, EMBASE, the Cochrane Central Register of Controlled Trials (CENTRAL), and Google Scholar using search terms related to PNB and ITMP for the management of post-cesarean delivery pain from inception until July 2021. The search parameters included a combination of free text, Medical Subject Headings, and EMTREE terms; search terms used for EMBASE and MEDLINE are presented in section S4 search terms. Two independent investigators (C.R. and Y.H.J.) performed the literature search.

The references were imported to EndNote software X 9.3 (Thompson Reuters, CA, USA), and duplicated articles were removed. Moreover, to ensure that we included all available articles, we identified additional relevant articles by scanning the reference lists of the original papers until there were no further relevant references. We also manually searched the reference lists of the related articles and meta-analyses to identify additional trials that matched the inclusion criteria of our study.

S1.2.2. Study Selection

Two independent investigators (C.W.B. and C.K.C.) screened the titles and abstracts of the retrieved articles, and duplicate articles were removed. To minimize data duplication due to multiple reporting, papers by the same author, organization, or country were compared. After this, for articles determined to be eligible based on the title and abstract, the full-text versions were retrieved. Potentially relevant articles found by at least one author were retrieved, and then the full text was evaluated. Any disagreements were discussed until a consensus was reached. In cases where an agreement could not be reached, the dispute was resolved with the help of another expert (H.K.). If the study period conducted by the same investigators overlapped, only the most recent study was included.

S1.3. Data Extraction and Management

S1.3.1. Data Extraction

Using standardized extraction, the following data were extracted independently by two independent investigators (C.R. and H.K.): (1) title, (2) name of the first author, (3) name of the journal, (4) year of publication, (5) country, (6) language, (7) primary anesthesia details and regimen (general versus neuraxial), (8) block technique and approach used, (9) number of subjects, (10) the kind and dose of drug used, (11) nature of primary and

secondary outcomes investigated, and (12) supplemental postoperative analgesia regimen. Data were primarily extracted from tables or results section of included studies. If data were incomplete, we tried to contact the study authors and requested additional information on their methodology and/or outcome data. If no response was obtained, missing information was calculated from the available data if possible. Data presented only in a graphical format were derived from the open source software Plot Digitizer (version 2.6.8; http://plotdigitizer.sourceforge.net, accessed on 1 December 2021).

The reference lists were divided into two halves. Then, two independent investigators each extracted data from each half of the reference list. Two data extraction forms were cross-checked to verify the accuracy and consistency of the extracted data.

The degree of agreement for study selection between the two independent investigators was computed using kappa statistics to measure the difference between the observed and expected agreements, i.e., whether they were random or by chance. Kappa values were interpreted as: (1) less than 0: less than chance agreement; (2) 0.01 to 0.20: slight agreement; (3) 0.21 to 0.40: fair agreement; (4) 0.41 to 0.60: moderate agreement; (5) 0.61 to 0.80: substantial agreement; (6) 0.8 to 0.99: almost perfect agreement [3].

S1.3.2. Data Management

The retrieved articles expressed pain severity using various forms of pain scales as follows: visual analog scale (VAS), numerical rating scale (NRS), numerical pain scale (NPS), verbal rating scale, etc. If various scales including VAS were used, we selected the VAS scale. For the four outcomes on postoperative pain severity at 6 and 24 h, all extracted postoperative pain data were converted to 0–10 point VAS scores (0 = none, 10 = worst pain imaginable). In the absence of the 6 h data, we analyzed them using the nearest one of the measured results between 2 h before and after. For postoperative cumulative 24 h morphine equivalent consumption, all data extracted on opioid consumption were converted to intravenous morphine equivalents (mg). Data on the time to first analgesic request are presented in hours.

If reported data were median (P_{25}–P_{75}), median (range), or mean (standard error of the mean), mean and standard deviations were calculated from these values [4]. When data were provided by mean and confidence interval (CI), these were statistically converted to a mean and standard deviation (SD) via the methods described by Wan et al. [5]. Nevertheless, if an SD value could not be obtained by the above methods, that value was excluded from the analysis.

S1.4. Quality Assessment

The quality of all included studies was independently assessed by two investigators (C.R. and H.K.), using version 2 of the Cochrane RoB tool for randomized trials. RoB 2 was evaluated by considering the following five potential sources of bias: (1) bias arising from the randomization process, (2) bias due to deviations from intended interventions, (3) bias due to missing outcome data, (4) bias in outcome measurements, and (5) bias in the selection of the reported results. We judged all five domains in each article according to a series of questions, "signaling questions," presented in the Cochrane RoB 2 to elicit information about features of the trial that are relevant to the risk of bias.

Thereafter, we evaluated the overall risk of biased judgment according to domain-level judgments. The methodology for each domain was assigned as "high risk of bias," "some concerns," or "low risk of bias" to reflect the risk of bias [13].

S1.5. Quality of the Evidence

Evidence grade was determined using the Grading of Recommendations Assessment, Development, and Evaluation (GRADE) system, which uses a sequential assessment of evidence quality, followed by an assessment of the risk–benefit balance and a subsequent judgment on the strength of the recommendations.

S1.6. Statistical Analysis

Ad hoc tables were designed to summarize data from the included studies and show their key characteristics and any important question related to the review objectives. After extracting the data, reviewers determined the feasibility of a meta-analysis.

A multiple treatment comparison NMA is a generalization method of meta-analysis that includes both direct and indirect comparisons of treatment. Before conducting the NMA, we determined whether a meta-analysis was possible. For this, we evaluated the similarity for direct comparison and transitivity assumptions for indirect comparison. The similarity assumption was checked for PICO-SD. Additionally, the transitivity assumption for the whole network was assessed by visually comparing the distribution of potential effect modifiers across comparisons such as demographics and the risk of bias (all risk versus removing "high risks of bias" for bias arising from the randomization process, and bias in outcome measurement).

Both frequentist and Bayesian NMA were conducted. A frequentist NMA was performed using Stata software (version 15; StataCorp LP, College Station, TX, USA) based on mvmeta with NMA graphical tools developed by Chaimani et al. [6].

Network plots linking all retrieved interventions including the control group were formed to indicate the kinds of postoperative analgesia strategies, the number of parturients under different strategies, and the level of pairwise comparisons. The nodes show postoperative analgesia strategies being compared, and the edges between the nodes show the eligible direct comparisons among those strategies. The nodes and edges were weighed according to the number of subjects who were randomized to that intervention and the inverse of the standard error of effect, respectively.

We evaluated the consistency assumption for the entire network using the design-by-treatment interaction model. We also evaluated each closed loop in the network in order to evaluate local inconsistencies between the direct and indirect effect estimates for the same comparison. For each loop, we estimated the inconsistency factor (IF) as the absolute difference between the direct and indirect estimates for each paired comparison in the loop [7].

Mean summary effects with CI were presented together with their predictive intervals (PrI) to facilitate interpretation of the results considering the magnitude of heterogeneity. PrIs provide an interval, which is expected to encompass the estimate of a future study.

A rankogram and cumulative ranking curve were drawn for each postoperative analgesia strategy. A rankogram is a plot of the probability of assuming each of the possible ranks. We used the surface under the cumulative ranking curve area (SUCRA) values to present the hierarchy of postoperative analgesia strategies for the primary and secondary outcomes. The SUCRA is a relative ranking measure that accounts for the uncertainty in the treatment order, that is, accounts both for the location and the variance of all relative treatment effects. SUCRA values range from 0% to 100%. A higher SUCRA value is regarded as a better result for individual interventions [8]. Comparison-adjusted funnel plots were used to assess the presence of small-study effects [9].

Additionally, to test the robustness of results of frequentist random NMA, we also conducted Bayesian NMA using fixed and random effects model and with Markov Chain Monte Carlo (MCMC) methods using R statistical package gemtc [10]. With a lack of understanding regarding pain after cesarean section, we used uninformative prior distributions as automatically provided by gemtc. MCMC simulations were run using four chains with different initial values for inferential 100,000 iterations after 50,000 burn-ins and thinning of 100.

Convergence of fixed and random models derived from MCMC simulations was assessed using trace and density plots, Gelman–Rubin–Books methods with potential scale reduction factor (PSRF) up to 1. The comparing the fit for fixed and random models was assessed using Dbar (posterior mean of the deviance), PD (adequate number of parame-

ters), and DIC (deviance information criterion, sum of Dbar and PD) statistics. Node-splitting models were used to evaluate consistency between direct and indirect comparisons. The MD and 95% credible interval (CrI) and rank probability were calculated. We also calculated SUCRA values from the Bayesian model and compared them with those in the frequentist model.

We also conducted a network meta-regression analysis to test the possible cause of heterogeneity.

S2. Results

S2.1. Study Selection

We initially identified 1164 unique citations from MEDLINE, EMBASE, the Cochrane Central Register of Controlled Trials, and Google Scholar databases. Additionally, we retrieved 10 more articles from the reference lists of related meta-analyses. A PRISMA flow chart of the study selection is shown in Figure 1. After the removal of duplicates (129 studies), we conducted extensive screening of the individual titles and abstracts of 1045 studies. In the first stage of study selection, the kappa value between the two investigators was 0.768. A total of 98 studies that met the predefined definitions of PICO-SD remained, whose eligibility we evaluated via full-text reviews. Among these, 22 additional studies were excluded for the reasons described in Figure 1. Excluded references are listed as follows:

(1) Undescribed approach:

20. Barney, E.Z.; Pedro, C.D.; Gamez, B.H.; Fuller, M.E.; Dominguez, J.E.; Habib, A.S. Ropivacaine and Ketorolac Wound Infusion for Post-Cesarean Delivery Analgesia: A Randomized Controlled Trial. *Obstet. Gynecol.* **2020**, *135*, 427–435.
96. Jarraya, A.; Zghal, J.; Abidi, S.; Smaoui, M.; Kolsi, K. Subarachnoid morphine versus TAP blocks for enhanced recovery after caesarean section delivery: A randomized controlled trial. *Anaesth. Crit. Care Pain Med.* **2016**, *35*, 391–393.
97. Kupiec, A.; Zwierzchowski, J.; Kowal-Janicka, J.; Goździk, W.; Fuchs, T.; Pomorski, M.; Zimmer, M.; Kübler, A. The analgesic efficiency of transversus abdominis plane (TAP) block after caesarean delivery. *Ginekol. Polska* **2018**, *89*, 421–424.
98. Sriramka, B.; Sahoo, N.; Panigrahi, S. Analgesic Efficacy of Ultrasound-guided Transversus Abdominis Plane Block following Caesarean Section. *Int. J. Perioper. Ultrasound Appl. Technol.* **2012**, *1*, 5–8.
99. Tuncer, S., Aysolmaz, G., Reisli R., Erol, A., Yalçin, N., Yosunkaya, A. The effects of the administration of subfacial levobupivacaine infusion with the ON-Q pain pump system on postoperative analgesia and tramadol consumption in cesarean operations. *Agri.* **2010**, *22*, 73–8.
100. Qian, H.; Zhang, Q.; Zhu, P.; Zhang, X.; Tian, L.; Feng, J.; Wu, Y.; Zhao, Z.; Luan, H. Ultrasound-guided transversus abdominis plane block using ropivacaine and dexmedetomidine in patients undergoing caesarian sections to relieve post-operative analgesia: A randomized controlled clinical trial. *Exp. Ther. Med.* **2020**, *20*, 1163–1168.
101. Buluc, H.; Ar, A.Y.; Turan, G.; Karadogan, F.; Sargin, M.A.; Akgun, N. The efficacy of transversus abdominis plane block for post-operative analgesia after the cesarean section performed under general anesthesia. *North. Clin. Istanb.* **2019**, *6*, 368–373.

(2) Same Block technique, different adjuvant:

102. Akkaya, A.; Yildiz, I.; Tekelioglu, U.Y.; Demirhan, A.; Bayir, H.; Ozlu, T.; Bilgi, M.; Kocoglu, H. Dexamethasone added to levobupivacaine in ultrasound-guided tranversus abdominis plain block increased the duration of postoperative analgesia after caesarean section: a randomized, double blind, controlled trial. *Eur. Rev. Med Pharmacol. Sci.* **2014**, *18*, 717–22.
103. Behdad, S.; Sekhavat, L.; Ayatollahi, V.; Meshkat, F.; Mortazavi, A. Comparison of postoperative analgesic effect of tramadol and bupivacaine subcutaneous infiltration in patients undergoing cesarean section. *Acta Clin. Croat.* **2013**, *52*.
104. Eslamian, L.; Kabiri-Nasab, M.; Agha-Husseini, M.; Azimaraghi, O.; Barzin, G.; Movafegh, A. Adding Sufentanil to TAP Block Hyperbaric Bupivacaine Decreases Post-Cesarean Delivery Morphine Consumption. *Acta MEDICA Iran.* **2016**, *54*.
105. Gupta, A.; Gupta, A.; Yadav, N. Effect of dexamethasone as an adjuvant to ropivacaine on duration and quality of analgesia in ultrasound-guided transversus abdominis plane block in patients undergoing lower segment cesarean section - A prospective, randomised, single-blinded study. *Indian J. Anaesth.* **2019**, *63*, 469–474.
106. Haliloglu, M.; Bilgen, S.; Menda, F.; Ozcan, P.; Ozbay, L.; Tatar, S.; Unal, D.O.; Koner, O. Analgesic efficacy of wound infiltration with tramadol after cesarean delivery under general anesthesia: Randomized trial. *J. Obstet. Gynaecol. Res.* **2016**, *42*, 816–821.
107. Ranjan, R.; John, R.; Ramachandran, T.; George, S.K. Analgesic efficacy of transverse abdominal plane block after elective cesarean delivery – Bupivacaine with fentanyl versus bupivacaine alone: A randomized, double-blind controlled clinical trial. *Anesthesia: Essays Res.* **2017**, *11*, 181–184.

108. Zachariah, S.K.; Joseph, B.; Abraham, S.P. The comparison of effects of fentanyl and dexmedetomidine as adjuvants to ropivacaine for ultrasound-guided transversus abdominis plane block for postoperative pain in cesarean section under spinal anesthesia –A randomized controlled trial. *J. Anaesthesiol. Clin. Pharmacol.* **2020**, *36*, 377–380.
109. Tharwat, A.A.; Yehia, A.H.; Wahba, K.A.; Ali, A.-E.G. Efficacy and safety of post-cesarean section incisional infiltration with lidocaine and epinephrine versus lidocaine alone in reducing postoperative pain: A randomized controlled double-blinded clinical trial. *J. Turk. Gynecol. Assoc.* **2016**, *17*, 1–5.
110. Katz, D.; Hamburger, J.; Gutman, D.; Wang, R.; Lin, H.; Marotta, M.; Zahn, J.; Beilin, Y. The Effect of Adding Subarachnoid Epinephrine to Hyperbaric Bupivacaine and Morphine for Repeat Cesarean Delivery: A Double-blind Prospective Randomized Control Trial. *Obstet. Anesthesia Dig.* **2019**, *39*, 51–52.
111. Onishi, Y.; Kato, R.; Okutomi, T.; Tabata, K.-I.; Amano, K.; Unno, N. Transversus abdominis plane block provides postoperative analgesic effects after cesarean section: Additional analgesia to epidural morphine alone. *J. Obstet. Gynaecol. Res.* **2013**, *39*, 1397–1405.

(3) Same Block technique, different concentration:

112. Ekmekçi, P.; Çağlar, G.S.; Yilmaz, H.; Kazbek, B.K.; Gursoy, A.Y.; Kiseli, M.; Tüzüner, F.; Yılmaz, H.; Gursoy, A.Y. Effects of different doses of tramadol added to levobupivacaine in continuous wound infusion for postoperative pain treatment following cesarean section. *J. Matern. Neonatal Med.* **2016**, *30*, 343–346.
113. Mohamed, A.Z.E.A. Assessment of the analgesic potency of ropivacaine 0.2% versus ropivacaine 0.5% in transversus abdominis plane block after cesarean delivery. *Egypt. J. Anaesth.* **2016**, *32*, 385–390.
114. Aly, M.; Ibrahim, A.; Farrag, W.; Abdelsalam, K.; Mohamed, H.; Tawfik, A. Pruritus after intrathecal morphine for cesarean delivery: incidence, severity and its relation to serum serotonin level. *Int. J. Obstet. Anesthesia* **2018**, *35*, 52–56.

(4) No eligible outcomes:

28. Canakci, E.; Gultekin, A.; Cebeci, Z.; Hanedan, B.; Kilinc, A. The Analgesic Efficacy of Transverse Abdominis Plane Block versus Epidural Block after Caesarean Delivery: Which One Is Effective? TAP Block? Epidural Block? *Pain Res. Manag.* **2018**, *2018*, 3562701.

Finally, we included 76 RCTs in this systematic review and the NMA [13-88]. In the second stage of study selection, the kappa value between the two investigators was 0.939.

S2.2. Study Characteristics

Finally, included studies were conducted in 34 countries, with the United States contributing the most (8 articles). Most of the articles were written in English, except for the following seven studies: three in French [11-13], two in Russian [14, 15], one in Spanish [16], and one in Persian [17] language. In papers published since 1991, we identified seven types of PNB methods: erector spinae plane (ESP) block, transverse fascial plane (TFP) block, quadratus lumborum (QL) block, ilioinguinal-iliohypogastric (II-IH) nerve block, TAP block, RS block, and surgical WI. Although there are several specific approach techniques for each block, only the following approach techniques were included in this study through data extraction: QL block (anterior, aQL; posterior, pQL; combined anterior and posterior, apQL; lateral approach, lQL), transversus abdominis plane (TAP) block (anterior, aTAP; lateral, lTAP; combined subcostal and lateral, slTAP; posterior approach, pTAP), continuous wound infusion (catheter insertion above or below the fascia, wound continuous infusion(WC)_above or WC_below, respectively). Consequently, we identified 28 different postoperative analgesia strategies (164 directly compared groups), except for the non-active control group (no intervention), either alone or in various combinations. Except for non-active controls (45 studies, 27.4%, 1835 patients), ITMP (active control, 24 studies, 14.6%, 891 patients), lTAP block (20 studies, 12.2%, 650 patients), and wound infiltration(WI) (15 studies, 9.1%, 662 patients) were compared the most. The included studies encompassed 6278 patients.

In all but one study, PNBs were performed immediately after the surgery in the operating room or post-anesthetic care unit. In one study, a study drug was injected subcutaneously in the line of the incision before starting the surgery [18].

The kind, concentration, and volume of each study drug used varied between studies (Table 1). At first, the types of local anesthetics used in the included studies are as follows: bupivacaine (0.125 to 0.5%), levobupivacaine (0.25 to 0.5%), ropivacaine (0.2 to 0.75%), and lidocaine (1 to 2%). The volume of local anesthetics injected ranged from 20 to 60 ml. The

dose of ITMP for spinal anesthesia ranged from 50 to 300 μg (mean with standard deviation: 133.8 ± 56.1 μg).

S2.3. Study Quality Assessment

The risk of bias assessment in included studies using the Cochrane RoB 2 is presented in the Table S1. When judging the overall risk of bias, only 13 studies had a low risk of bias in all domains. The main issues that judged the risk of bias for each domain were as follows:

First, considering bias arising from the randomization process, the most important criterion for judging was "allocation sequence concealment'. One article [19] that determined the kinds of intervention according to the preference of each patient was judged as high risk. Studies in which allocation sequence concealment was not performed determined the final risk by judging baseline imbalance. As a result, a total of 24 studies were judged as 'some concerns' in this domain.

Second, in the studies that did not perform injection or infusion of the same amount of 0.9% normal saline or sham block in the control group, the participants may be aware of their assigned intervention, and the investigators who conducted the procedures could not inevitably be blinded. However, in these cases, investigators do not always report whether deviations arose due to the trial context, so we judged them as 'no information' considering deviations from the intended intervention (signaling questions S1.3) [20]. Additionally, there is no case of analysis in the wrong group. As a result, a total of 27 studies were judged as 'some concerns' in this domain. In total, 14 studies compared different kinds of PNB methods. In 10 studies comparing the PNB method and control group (active or non-active controls), investigators did not perform any intervention on the control group, including sham block.

Third, we evaluated the 'risk of bias due to missing outcome data' through the results section and CONSORT flow diagrams in each article. In one study [21], the trial was terminated early in accordance with the recommendation of an independent pharmaco-vigilance committee because of major complications related to the procedure. In other studies, although there was no information about conducting sensitivity analyses, most of the excluded subjects were missed because of the reasons unrelated to the outcomes, true values.

Fourth, for the outcomes measuring the level of pain, outcome assessors are participants themselves, not observers. In 10 studies that did not perform any intervention on the control group, the participants must have been aware of their allocation. Then, the assessment of the outcome measuring level of pain could have been influenced by knowledge of the intervention received. Therefore, we judged them as 'some concerns' in the domain of risk of bias in the measurement of the outcome.

Finally, there was no information about pre-registration for clinical trials before enrollment in 44 studies, and therefore, we judged them as 'some concerns' in the domain of risk of bias in the selection of the reported result.

S2.4. Synthesis of Results

S2.4.1. Primary Outcomes

The results of primary outcomes are described in the main text.

S2.4.2. Secondary Outcomes

Pain at Rest 24 h after Surgery.

Although a total of 60 studies (4747 patients) measured the pain at rest 24 h after surgery, as one study was separated from the loops [22], we performed NMA excluding that study. Therefore, a total of 59 studies (4697 patients) were finally analyzed. The network plot of all eligible comparisons for this outcome is depicted in Figure 2C.

Although all 27 analgesic management strategies (nodes) were connected to the network, three comparisons (Control, ITMP, and lTAP) were more directly comparable than the other 24 nodes. There was no evidence of network inconsistency (χ^2 (16) = 59.65, $p <$ 0.001). There were 21 triangular loops and 3 quadratic loops closed in the network from the comparison of this outcome.

Six loops (Control/aTAP/II-IH [14], aQL/pQL/apQL [23], aQL/pQL/epidural morphine(EDMP) [23], aQL/apQL/EDMP [23], pQL/apQL/EDMP [23], RS/ITMP + RS/ITMP [24]) were formed only by multi-arm trials. Although almost loops showed no significance in the local inconsistency between the direct and indirect point estimates, four loops (pQL/WC_below/ITMP/EDMP, Control/pQL/WC_below/EDMP, Control/aQL/WC_below/EDMP, and pQL/ITMP + pQL/ITMP) showed significant inconsistency (Figure S1C).

Wound continuous infusion below the fascia (WC_below) showed a lower level of pain than control only in terms of a 95% confidence interval (Figure 3C).

The rankograms showed that slTAP and ITMP + II-aTAP had the lowest level of pain at rest 24 h after surgery (Figure S2C). The cumulative ranking plot was drawn, and the SUCRA probabilities of the different interventions for this endpoint were calculated (Figure S3C). The expected mean rankings and SUCRA values of each intervention are presented in Figure 4C. According to the SUCRA values, the pain at rest 24 h after surgery was lower in the order of the slTAP (83.6%), followed by ITMP + II-aTAP (82.1%), intraperitoneal local anesthetic instillation(IPLA) (80.1%), and WC_below (79.8%). The comparison-adjusted funnel plots showed that the funnel plots were symmetrical around the zero line, which suggested a less likely publication bias (Figure S4C).

The network diagnostics were tested using trace, density plot, and Gelman–Rubin–Brooks methods with PSRF and DIC (Figure S7A–D, Table S2). Thus, we analyzed data using a random-effects model.

Forest plot, node splitting plot, rankogram, and SUCRA values from the Bayesian model showed similar results to those from the frequentist model, which shows the robustness of our analysis (Figure S7E–G and Figure 4C).

Dynamic Pain at 6 h after Surgery.

Although a total of 38 studies (2923 patients) measured dynamic pain at 6 h after surgery, as one study was separated from the loops [22], we performed NMA excluding that study. Therefore, a total of 37 studies (2873 patients) measured dynamic pain severity at 6 h after surgery. The network plot of all eligible comparisons for this endpoint is depicted in Figure 2D.

The definitions of dynamic pain during active movement varied between studies as follows: sitting up from the lying position, mobilization, coughing, leg elevation, elevation of the head and shoulders from the pillow in the supine position, knee flexion, etc.

Although all 24 analgesic management strategies (nodes) were connected to the network, three comparisons (Control, ITMP, and lTAP) were more directly comparable than the other 21 nodes. There was no evidence of network inconsistency (χ^2 (12) = 70.30, $p <$ 0.001). There were 19 triangular loops and 1 quadratic loop closed in the network from the comparison of dynamic pain at 6 h after surgery.

Six loops (Control/aTAP/ II-IH [14], aQL/pQL/apQL [23], aQL/pQL/EDMP [23], aQL/apQL/EDMP [23], pQL/apQL/EDMP [23], and RS/ITMP + RS/ITMP [24]) were formed only by multi-arm trials. Although almost loops showed no significance in the local inconsistency between direct and indirect point estimates, 6 loops (lTAP/WI/ITMP, Control/WI/ITMP, Control/pQL/WC_below/EDMP, Control/pQL/ITMP + pQL, Control/ITMP + pQL/ITMP, and pQL/ITMP + pQL/ITMP) showed significant inconsistency (Figure S1D). WI showed a lower level of pain than control only in terms of 95% CI (Figure 3D).

The rankograms showed that WI and ESP had the lowest dynamic pain at 6 h after surgery (Figure S2D). The cumulative ranking plot was drawn, and the SUCRA probabil-

ities of the different interventions for this outcome were calculated (Figure S3D). The expected mean rankings and SUCRA values of each intervention are presented in Figure 4D. According to the SUCRA value, the dynamic pain at 6 h after surgery was lower in the order of WI (78.9%), followed by ESP (72.4%), slTAP (71.7%), and apQL (64.4%). The comparison-adjusted funnel plots showed that the funnel plots were symmetrical around the zero line, which suggested a less likely publication bias (Figure S4D).

The network diagnostics were tested using trace, density plot, and Gelman–Rubin–Brooks methods with PSRF and DIC (Figure S8A–D, Table S2). Thus, we analyzed data using a random-effects model.

Forest plot, node splitting plot, rankogram, and SUCRA values from the Bayesian model showed similar results to those from the frequentist model, which shows the robustness of our analysis (Figure S8E–G and Figure 4D).

Dynamic Pain 24 h after Surgery.

Although a total of 45 studies (3421 patients) measured dynamic pain at 24 h after surgery, as one study was separated from the loops [22], we performed NMA excluding that study. Therefore, a total of 44 studies (3371 patients) were analyzed. The network plot of all eligible comparisons for this endpoint is depicted in Figure 2E.

Although all 24 analgesic management strategies (nodes) were connected to the network, three comparisons (Control, ITMP, and lTAP) were more directly comparable than the other 21 nodes. There was no evidence of network inconsistency (χ^2 (12) = 35.96, $p <$ 0.001). There were 19 triangular loops and 1 quadratic loop closed in the network from the comparison of dynamic pain at 24 h after surgery.

Six loops (Control/aTAP/ II-IH [14], aQL/pQL/apQL [23], aQL/pQL/EDMP [23], aQL/apQL/EDMP [23], pQL/apQL/EDMP [23], and RS/ITMP + RS/ITMP [24]) were formed only by multi-arm trials. Although almost loops showed no significance in the local inconsistency between the direct and indirect point estimates, three loops (Control/pQL/WC_below/EDMP, pQL/ITMP + pQL/ITMP, Control/pQL/ITMP + pQL) showed significant inconsistency (Figure S1E).

EDMP, apQL, WC_below, and lTAP showed a lower level of pain than control in terms of 95% CI only (Figure 3E).

The rankograms showed that EDMP and apQL had the lowest dynamic pain at 24 h after surgery (Figure S2E). The cumulative ranking plot was drawn, and the SUCRA probabilities of the different interventions for dynamic pain at 24 h after surgery were calculated (Figure S3E). The expected mean rankings and SUCRA values of each intervention are presented in Figure 4E. According to the SUCRA value, the dynamic pain at 24 h after surgery was lower in the order of EDMP (96.7%), followed by apQL (89.2%), ESP (74.2%), and WC_below (71.3%). The comparison-adjusted funnel plots showed that the funnel plots were symmetrical around the zero line, which suggested a less likely publication bias (Figure S4E).

The network diagnostics were tested using trace, density plot, and Gelman–Rubin–Brooks methods with PSRF and DIC (Figure S9A–D, Table S2). Thus, we analyzed data using a random-effects model.

Forest plot, node splitting plot, rankogram, and SUCRA values from the Bayesian model showed similar results to those from the frequentist model, which shows the robustness of our analysis (Figure S9E–G and Figure 4E).

The Time to First Analgesic Request (Hours).

A total of 24 studies (1812 patients) reported the time to first analgesic request, and we analyzed those results. The network plot of all eligible comparisons for this endpoint is depicted in Figure 2F.

Although all 16 analgesic management strategies (nodes) were connected to the network, three comparisons (Control, lTAP, and ITMP) were more directly comparable than the other 13 nodes. There was no evidence of network inconsistency (χ^2 (5) = 3.11, p =

0.683). There were six triangular loops closed in the network from the comparison of the time to the first analgesic request. One loop (Control/aTAP/II-IH [14]) was formed only by a multi-arm trial. Although almost loops showed no significance in the local inconsistency between direct and indirect point estimates, one loop (Control/WC_below/ITMP) showed significant inconsistency (Figure S1F).

ESP showed a longer time to first analgesic request than control in terms of 95% CI [30.84,42.04] and PrI [19.95,52.94] at the same time. Additionally, ITMP, pTAP, II-IH, lTAP, ITMP + WI, TFP, and pQL showed longer times to first analgesic request than control only in terms of 95% CI (Figure 3F).

The rankograms showed that ESP had the longest time to first analgesic request (Figure S2F). The cumulative ranking plot was drawn, and the SUCRA probabilities of the different interventions for the time to the first analgesic request were calculated (Figure S3F). The expected mean rankings and SUCRA values of each intervention are presented in Figure 4F. According to the SUCRA value, the time to first analgesic request was longer in the order of the ESP (100.0%), followed by pQL (91.2%), TFP (87.5%), ITMP + WI (63.8%), and lTAP(62.9%). The comparison-adjusted funnel plots showed that the funnel plots were symmetrical around the zero line, which suggested a less likely publication bias (Figure S4F).

The network diagnostics were tested using trace, density plot, and Gelman–Rubin–Brooks methods with PSRF and DIC (Figure S10A–D, Table S2). Thus, we analyzed data using a random-effects model.

Forest plot, node splitting plot, rankogram, and SUCRA values from the Bayesian model showed similar results to those from the frequentist model, which shows the robustness of our analysis (Figure S10E–G, and Figure 4F).

S2.5. Quality of the Evidence

Six outcomes were evaluated using the GRADE system. The evidence quality was moderate for pain at rest 6 h after surgery, postoperative cumulative 24 h morphine equivalent consumption and the time to first analgesic request, and low for pain at rest 24 h after surgery, dynamic pain at 6 h after surgery, and dynamic pain 24 h after surgery. Study limitations for all outcomes were downgraded because 50% of included studies were graded as “some concerns” or “high” in risk of bias assessment. Inconsistencies for pain at rest 24 h after surgery, dynamic pain at 6 h after surgery, and dynamic pain 24 h after surgery were downgraded, as the overall inconsistency evaluated by the design-by-treatment interaction was statistically significant (Table S4).

S3. Discussion

First, the examples of studies showing that the analgesic effect differs according to the anatomical approach of PNBs are as follows: The subcostal approach for TAP block showed a larger area of spread of dye than the lateral approach in a cadaveric study [25]. A meta-analysis about analgesic effectiveness after TAP block for transverse lower abdominal incisions concluded that the posterior approach appears to produce more prolonged postoperative analgesia, compared with the lateral approach [26]. Transmuscular or anterior QL block (type 3) allows a wider spread of local anesthetic into the thoracic paravertebral space than lateral and posterior approaches, and thus it is expected to be effective on not only somatosensory pain caused by the surgical incision but also visceral pain [27, 28]. Finally, an RCT showed that continuous wound infusion of local anesthetics below the fascia provided significantly improved analgesic outcomes, compared with wound infusion above the fascia [22].

Second, why is the analgesic effect of this combined PNB so excellent? The ilioinguinal nerve arises from the L1 nerve root and lies between the internal and external oblique muscles. Then, it pierces the external oblique muscle superior and medial to anterior superior iliac spine (ASIS) to provide cutaneous sensation. Therefore, this block provides anterior abdominal wall analgesia, specifically in the dermatomal distribution supplied

by L1 where the Pfannenstiel incision lies [29]. On the other hand, a comparative study demonstrated that TAP block failed to provide the sensory blockade on L1 dermatome in above 50% of patients even with ultrasound [30] and suggested that a more anterior approach may provide more reliable coverage on the L1 dermatome where the incision line for cesarean section [31, 32]. The target of the anterior TAP block is the same fascial plane as that of the other approaches at the level of the deep circumflex iliac artery. Therefore, it is considered that II-aTAP block combines the concept of compartment fascial plane block used in TAP block as well as targeting specific PNB, the ilioinguinal nerve, even at the same needle insertion point. As a result, it is believed that the two individual PNB methods may have shown additional or synergistic analgesic effects on L1 dermatome. For this procedure, the needle is inserted 2 cm medial and 2 cm superior to the ASIS [29, 33]. Then, the local anesthetics are injected into the following two fascial planes with divided doses at a time: the first plane between the external and internal oblique muscles, and the second plane between the internal oblique and transversus abdominis muscle. Therefore, no additional risk is associated with this procedure, compared with other PNB methods.

S4. Search Terms

S4.1. Search Terms for Ovid-MEDLINE

1. randomized controlled trial.pt
2. randomized controlled trial$.mp
3. controlled clinical trial.pt
4. controlled clinical trial$.mp
5. random allocation.mp
6. exp double-blind method/
7. double-blind.mp
8. exp single-blind method/
9. single-blind.mp
10. or/1-9
11. clinical trial.pt
12. clinical trial$.mp
13. exp clinical trial/
14. (clin$ adj25 trial$).mp
15. ((singl$ or doubl$ or tripl$ or trebl$) adj25 (blind$ or mask$)).mp
16. random$.mp
17. exp research design/
18. research design.mp
19. or/11-18
20. 10 or 19
21. Case report.tw.
22. Letter.pt.
23. Historical article.pt.

24. Review.pt.
25. or/21-24
26. 20 not 25
27. Exp Cesarean Section/
28. Caesarean section.mp
29. Cesarean delivery.mp
30. Caesaerean delivery.mp.
31. C-section.mp
32. C section.mp
33. C-sections.mp
34. Abdominal delivery.mp
35. Abdominal deliveries.mp
36. Delivery, abdominal.mp
37. Or/27-36
38. 26 and 37
39. Exp Anesthesia, conduction/
40. Intrathecal$.mp
41. Subarachnoid.mp
42. Spinal.mp
43. Neuraxial.mp
44. epidural.mp
45. Or/39-44
46. Exp Analgesics, Opioid/
47. Morphine.mp
48. Fentanyl.mp
49. Sufentanil.mp
50. Alfentanil.mp
51. Nalbuphine.mp
52. Clonidine.mp
53. Dexmedetomidine.mp
54. Midazolam.mp
55. Or/46-54
56. 45 AND 55
57. Lumbar paravertebral.mp
58. Transversus abdominis plane.mp
59. TAP block.mp

60. Quadratus lumborum.mp
61. (Ilioinguinal adj25 iliohypogastric).mp
62. Erector spinae plane.mp
63. Rectus sheath.mp
64. Direct field.mp
65. Incisional block.mp
66. Wound infiltration.mp
67. Wound infusion.mp
68. Direct field block.mp
69. Continuous infiltration.mp
70. Or/56-69
71. 38 AND 70

S4.2. Search Terms for EMBASE

1. randomi?ed:ti AND controlled:ti AND trial$:ti
2. 'controlled clinical trial (topic)'/exp
3. controlled AND clinical AND trials
4. controlled clinical trial$.mp
5. 'randomization'/exp
6. 'random allocation'/exp
7. random allocation.mp.
8. double-blind.mp.
9. single-blind.mp.
10. #1 OR #2 OR #3 OR #4 OR #5 OR #6 OR #7 OR #8 OR #9
11. 'clinical trial (topic)'/exp
12. clinical AND trial$.mp.
13. random$.mp.
14. rct
15. #11 OR #12 OR #13 OR #14
16. #10 OR #15
17. 'case study'/exp
18. 'case report'/exp
19. 'abstract report'/exp
20. 'letter'/exp
21. #17 OR #18 OR #19 OR #20
22. #16 NOT #21
23. 'cesarean section'/exp

24. caesarean:ti,ab AND section:ti,ab
25. cesarean:ti,ab AND delivery:ti,ab
26. cesaerean:ti,ab AND delivery:ti,ab
27. C-section
28. abdominal:ti,ab AND delivery:ti,ab
29. #23 OR #24 OR #25 OR #26 OR #27 OR #28
30. #22 AND #29
31. 'spinal anesthesia'/exp
32. intrathecal$:ti,ab
33. Subarachnoid:ti,ab
34. Spinal:ti,ab
35. Neuraxial:ti,ab
36. epidural:ti,ab
37. #31 OR #32 OR #33 OR #34 OR #35 OR #36
38. 'opiate'/exp
39. Morphine:ti,ab
40. Fentanyl:ti,ab
41. Sufentanil:ti,ab
42. Alfentanil:ti,ab
43. Nalbuphine:ti,ab
44. Clonidine:ti,ab
45. Dexmedetomidine:ti,ab
46. Midazolam:ti,ab
47. #38 OR #39 OR #40 OR #41 OR #42 OR #43 OR #44 OR #45 OR #46
48. #37 AND #47
49. Lumbar AND paravertebral
50. Transversus AND abdominis AND plane
51. TAP AND block
52. Quadratus AND lumborum
53. Ilioinguinal AND iliohypogastric
54. Erector AND spinae AND plane
55. Rectus AND sheath
56. Direct AND field
57. Incisional AND block
58. Wound AND infiltration
59. Wound AND infusion

60. Direct AND field AND block
61. Continuous AND infiltration
62. #48 OR #49 OR #50 OR #51 OR #52 OR #53 OR #54 OR #55 OR #56 OR #57 OR #58
63. #30 AND #59

References

13. Sterne, J.A.C.; Savović, J.; Page, M.J.; Elbers, R.G.; Blencowe, N.S.; Boutron, I.; Cates, C.J.; Cheng, H.Y.; Corbett, M.S.; Eldridge, S.M.; et al. RoB 2: A revised tool for assessing risk of bias in randomised trials. *BMJ* **2019**, *366*, l4898.
14. Chaimani, A.; Higgins, J.P.; Mavridis, D.; Spyridonos, P.; Salanti, G. Graphical tools for network meta-analysis in STATA. *PLoS ONE* **2013**, *8*, e76654.
15. van Valkenhoef, G.; Lu, G.; de Brock, B.; Hillege, H.; Ades, A.E.; Welton, N.J. Automating network meta-analysis. *Res. Synth. Methods* **2012**, *3*, 285–299.
21. Belavy, D.; Cowlishaw, P.J.; Howes, M.; Phillips, F. Ultrasound-guided transversus abdominis plane block for analgesia after Caesarean delivery. *Br. J. Anaesth.* **2009**, *103*, 726–730.
23. Bensghir, M.; Elwali, A.; Miller, C.; Azendour, H.; Drissi, M.; Bakkali, H.; Belyamani, L.; Atmani, M.; Drissi Kamili, N. Effects of skin infiltration with ropivacaine 0.75% on postoperative pain after caesarean section. *Gynecol. Obstet. Fertil.* **2008**, *36*, 516–520.
24. Bessmertnyj, A.E.; Antipin, E.E.; Uvarov, D.N.; Sedyh, S.V.; Nedashkovsky, E.V. Comparison of the Effectiveness of Ilioinguinal-Iliohypogastric Blockade and Transversus Abdominis Plane Block for Analgesia after Cesarean Section. *Anesteziol. Reanimatol.* **2015**, *60*, 51–54.
29. Canovas, L.; Lopez, C.; Castro, M.; Rodriguez, A.B.; Perez, L. Contribution to post-caesarean analgesia of ultrasound-guided transversus abdominis plane block. *Rev. Esp. Anestesiol. Reanim.* **2013**, *60*, 124–128.
30. Chandon, M.; Bonnet, A.; Burg, Y.; Barnichon, C.; DesMesnards-Smaja, V.; Sitbon, B.; Foiret, C.; Dreyfus, J.F.; Rahmani, J.; Laloe, P.A.; et al. Ultrasound-guided Transversus Abdominis plane block versus continuous wound infusion for post-caesarean analgesia: A randomized trial. *PLoS ONE* **2014**, *9*, e103971.
31. Corsini, T.; Cuvillon, P.; Forgeot, A.; Chapelle, C.; Seffert, P.; Chauleur, C. Single-dose intraincisional levobupivacaine infiltration in caesarean postoperative analgesia: A placebo-controlled double-blind randomized trial. *Ann. Fr. Anesth. Reanim.* **2013**, *32*, 25–30.
35. Ducarme, G.; Sillou, S.; Wernet, A.; Davitian, C.; Poujade, O.; Ceccaldi, P.F.; Bougeois, B.; Luton, D. Single-shot ropivacaine wound infiltration during cesarean section for postoperative pain relief. *Gynecol. Obstet. Fertil.* **2012**, *40*, 10–13.
38. Fakor, F.; Farzi, F.; Abdollahzadeh, M.; Golrizan, F.; Kazemnejad, E. The Effect of Transversus Abdominis Plane (TAP) Block with Bupivacaine 25% on Post Cesarean Pain. *J. Guilan Univ. Med. Sci.* **2014**, *23*, 53–60.
50. Kang, W.; Lu, D.; Yang, X.; Zhou, Z.; Chen, X.; Chen, K.; Zhou, X.; Feng, X. Postoperative analgesic effects of various quadratus lumborum block approaches following cesarean section: A randomized controlled trial. *J. Pain Res.* **2019**, *12*, 2305–2312.
60. Lui, M.W.; Li, T.K.T.; Lui, F.; Ong, C.Y.T. A randomised, controlled trial of rectus sheath bupivacaine and intrathecal bupivacaine, without or with intrathecal morphine, vs. intrathecal bupivacaine and morphine after caesarean section. *Anaesthesia* **2017**, *72*, 1225–1229.
72. Rackelboom, T.; Le Strat, S.; Silvera, S.; Schmitz, T.; Bassot, A.; Goffinet, F.; Ozier, Y.; Beaussier, M.; Mignon, A. Improving Continuous Wound Infusion Effectiveness for Postoperative Analgesia After Cesarean Delivery. *Obstet. Gynecol.* **2010**, *116*, 893–900.
75. Sekhavat, L.; Behdad, S. Preoperative analgesia with local lidocaine for cesarean delivery pain relief. *J. Matern. Fetal Neonatal Med.* **2011**, *24*, 891–893.
80. Staker, J.J.; Liu, D.; Church, R.; Carlson, D.J.; Panahkhahi, M.; Lim, A.; LeCong, T. A triple-blind, placebo-controlled randomised trial of the ilioinguinal-transversus abdominis plane (I-TAP) nerve block for elective caesarean section. *Anaesthesia* **2018**, *73*, 594–602.
81. Svirskiĭ, D.A.; Antipin, E.E.; Uvarov, D.N.; Nedashkovskiĭ, E.V. Abdominal cross section space blockade as a component of the multimodal postoperative analgesia in patients after cesarean section: Blockade efficiency analysis. *Anesteziol. Reanimatol.* **2012**, *6*, 33–35.
95. Abdallah, F.W.; Laffey, J.G.; Halpern, S.H.; Brull, R. Duration of analgesic effectiveness after the posterior and lateral transversus abdominis plane block techniques for transverse lower abdominal incisions: A meta-analysis. *Br. J. Anaesth.* **2013**, *111*, 721–735.
115. Kintu, A.; Abdulla, S.; Lubikire, A.; Nabukenya, M.T.; Igaga, E.; Bulamba, F.; Semakula, D.; Olufolabi, A.J. Postoperative pain after cesarean section: assessment and management in a tertiary hospital in a low-income country. *BMC Heal. Serv. Res.* **2019**, *19*, 68.
116. Joshi, G.; Gandhi, K.; Shah, N.; Gadsden, J.; Corman, S.L. Peripheral nerve blocks in the management of postoperative pain: challenges and opportunities. *J. Clin. Anesthesia* **2016**, *35*, 524–529.
117. Viera, A.J.; Garrett, J.M. Understanding interobserver agreement: the kappa statistic. *Fam. Med.* **2005**, *37*, 360–3.

118. Hozo, S.P.; Djulbegovic, B.; Hozo, I. Estimating the mean and variance from the median, range, and the size of a sample. *BMC Med Res. Methodol.* **2005**, *5*, 13.
119. Wan, X.; Wang, W.; Liu, J.; Tong, T. Estimating the sample mean and standard deviation from the sample size, median, range and/or interquartile range. *BMC Med Res. Methodol.* **2014**, *14*, 1–13.
120. White, I.R.; Barrett, J.K.; Jackson, D.; Higgins, J.P.T. Consistency and inconsistency in network meta-analysis: model estimation using multivariate meta-regression. *Res. Synth. Methods* **2012**, *3*, 111–125.
121. Salanti, G.; Ades, A.; Ioannidis, J.P. Graphical methods and numerical summaries for presenting results from multiple-treatment meta-analysis: an overview and tutorial. *J. Clin. Epidemiology* **2011**, *64*, 163–171.
122. Riley, R.D.; Higgins, J.; Deeks, J. Interpretation of random effects meta-analyses. *BMJ* **2011**, *342*, d549, doi:10.1136/bmj.d549.
124. Milan, Z.; Tabor, D.; McConnell, P.; Pickering, J.; Kocarev, M.; Du Feu, F.; Barton, S. Three different approaches to Transversus abdominis planeblock: a cadaveric study. *Med. Glas. : Off. Publ. Med Assoc. Zenica-Doboj Canton, Bosnia Herzeg.* **2011**, *8*, 181–4.
125. Hansen, C.K.; Dam, M.; Bendtsen, T.F.; Børglum, J. Ultrasound-Guided Quadratus Lumborum Blocks. *A A Pr.* **2016**, *6*, 39.
126. Elsharkawy, H.; El-Boghdadly, K.; Barrington, M. Quadratus Lumborum Block. *Anesthesiol.* **2019**, *130*, 322–335.
127. Patel, S.; El Sharawi, N.; Sultan, P. Local anaesthetic techniques for post-caesarean delivery analgesia. *Int. J. Obstet. Anesthesia* **2019**, *40*, 62–77.
128. Lee, T.H.W.; Barrington, M.J.; Tran, T.M.N.; Wong, D.; Hebbard, P.D. Comparison of Extent of Sensory Block following Posterior and Subcostal Approaches to Ultrasound-Guided Transversus Abdominis Plane Block. *Anaesth. Intensiv. Care* **2010**, *38*, 452–460.
129. Moore, K.L.; Dalley, A.F.; Agur, A.M.R. *Clinically oriented* anatomy, 8th ed.; *Wolters Kluwer Health/Lippincott Williams & Wilkins*: Philadelphia, PA, USA, 2010.
